# Supplementary material for: Transcriptional profiling of identified neurons in leech
Source: BMC Genomics. 2021 Mar 25;22:215. doi: 10.1186/s12864-021-07526-0 (PMC7992859; doi:10.1186/s12864-021-07526-0)
Supplement: Supplementary file 1 — Additional file 1. [file 12864_2021_7526_MOESM1_ESM.pdf]

Supplementary Tables and Figures

Table S1: Primers used in this study

Common Name	F (*H. verbana*)	R (*H. verbana*)	F (*H. robusta*)	R (*H. robusta*)
**Inositol Triphosphate Receptor**	gagggagaatccgtgcaaata	gaagcactctcaccaacaaatc	ctaggcagtacctatgcatcac	atagcacccatccctcctta
**Collagen-alpha**	ccgtcagttccctggtttc	caagggtgactctggagaaatag	gtattgaaggtcctgtgggaat	agtggactgggaggatcaa
**Annelid hypothetical**	cggcgtgaacatctcatagaa	ctcctcccacaacatggaaa	n/a	n/a
**Protocadherin**	ccagtcacagggtcgaaatag	cccgctaaagtgatcgtgaa	tggctggaatctcaaacttact	cgggcgtcgatgttaaattattc
**HCN Channel**	tttccctgtaatgcctctttga	cggagtccagaggaggataa	cccatgctacaagacttccc	tcgtacgttgcataggataaaca
**Voltage-Gated Potassium Channel**	tactctccgctcaaaagtgc	taatacgactcactataggaagtccttaat gcttgcgc (with t7)	n/a	n/a
**Aromatic Amino Acid Decarboxylase**	gaaggcctttgtctcaggtaa	tgtctgaactcgggacaaataaa	atgatgaagtggctcggtaag	gttgacgtcaccttcgttagt
**Tryptophan Hydroxylase**	tgaccatcctggtttcaaaga	tcgataaaggtttcctgctctactgc	gatggtcacccgtggtttcc	tcgacaagttgaacagtttatcctgg

Fig. S1. Detection of Outliers in the RNASeq Dataset. RobustPCA of the original 14 samples (L). Samples shown with high score and orthogonal distances are outliers by this analysis. Right, MDS plot of the original 14 samples with the samples identified as outliers labeled.

Fig. S2. Expression patterns of each isolated cluster. Centroids of each cluster are shown in color, while the expression of each Trinity gene belonging to the cluster is shown in light grey.

Fig. S3. Expression of neuron-specific transcripts found in *Hirudo* in late-stage *Helobdella* embryos. ISH of stage 11 *Helobdella austinensis* embryos using probes specific to the *Helobdella* orthologs of six of the transcripts verified in figure 3. Probe localization is shown in blue/purple. For each probe, a light micrograph of a chain of 4 mid-body ganglia (left), and a single ganglion at higher magnification (right) are shown. Anterior-posterior (A-P) axis orientation is shown at left.

Fig. S4. Full Phylogenetic tree shown in Figure 5, with all members of each clade shown. Scale bar = the average number of substitutions per site along each branch.

Figure S1

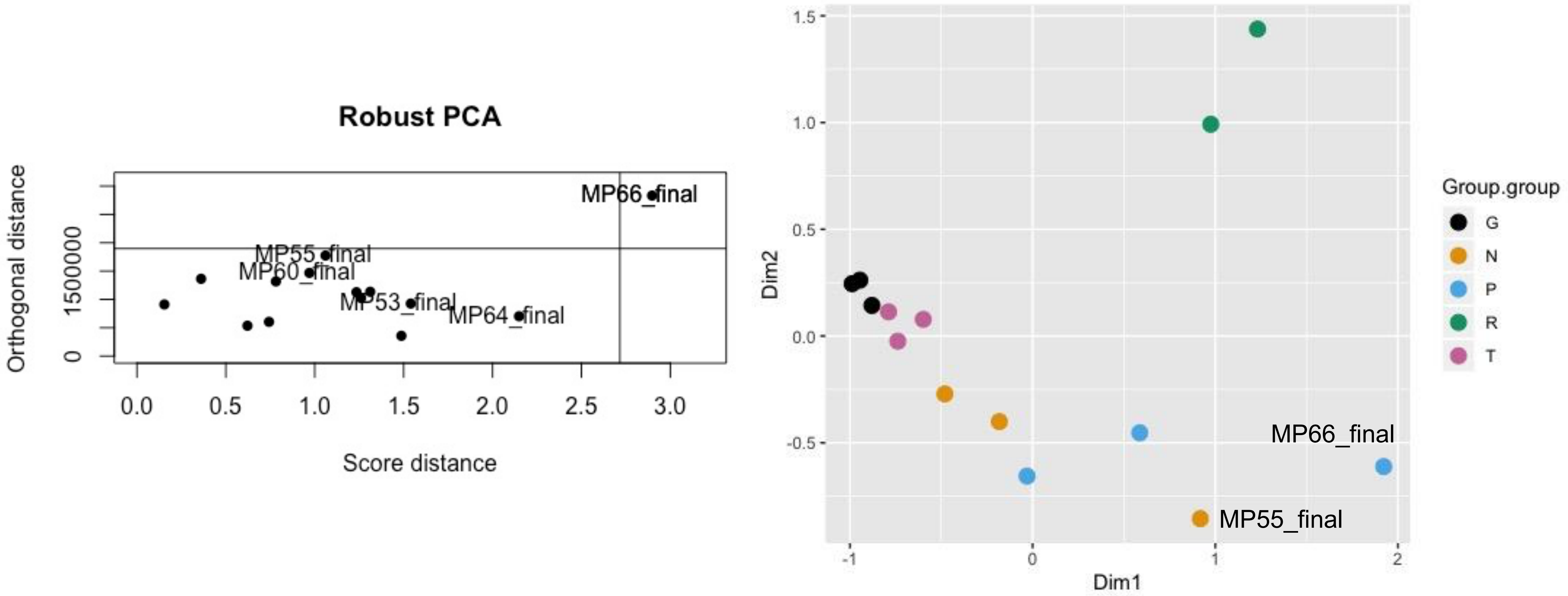

Robust PCA
Orthogonal distance
0
1500000
0.0
0.5
1.0
1.5
2.0
2.5
3.0
Score distance
MP66_final
MP55_final
MP60_final
MP53_final
MP64_final
1.5
1.0
0.5
0.0
-0.5
Dim2
-1
0
1
2
Dim1
MP66_final
MP55_final
Group.group
G
N
P
R
T

Figure S2

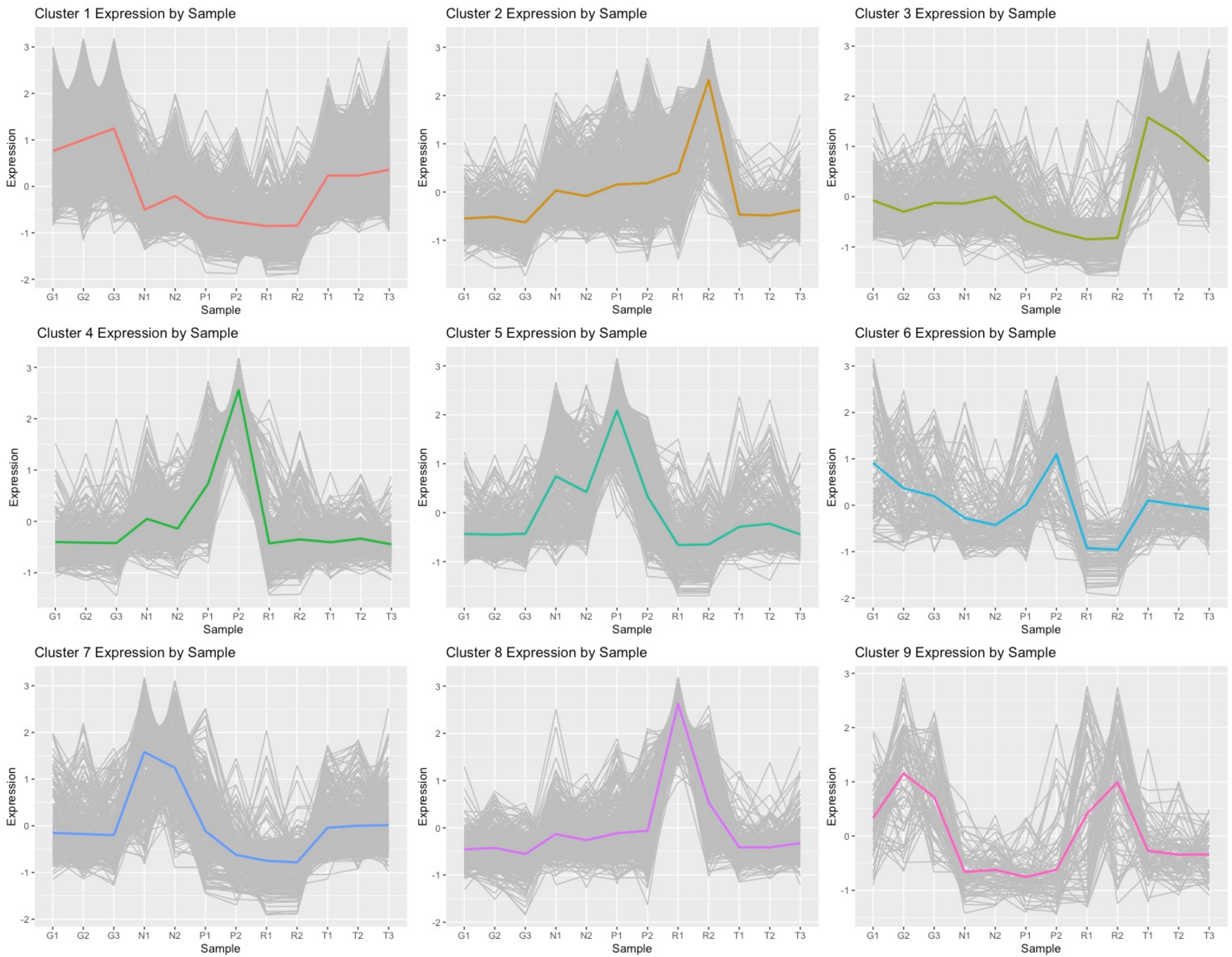

Cluster 1 Expression by Sample
Cluster 2 Expression by Sample
Cluster 3 Expression by Sample
Cluster 4 Expression by Sample
Cluster 5 Expression by Sample
Cluster 6 Expression by Sample
Cluster 7 Expression by Sample
Cluster 8 Expression by Sample
Cluster 9 Expression by Sample
Expression
Sample
G1 G2 G3 N1 N2 P1 P2 R1 R2 T1 T2 T3

Figure S3

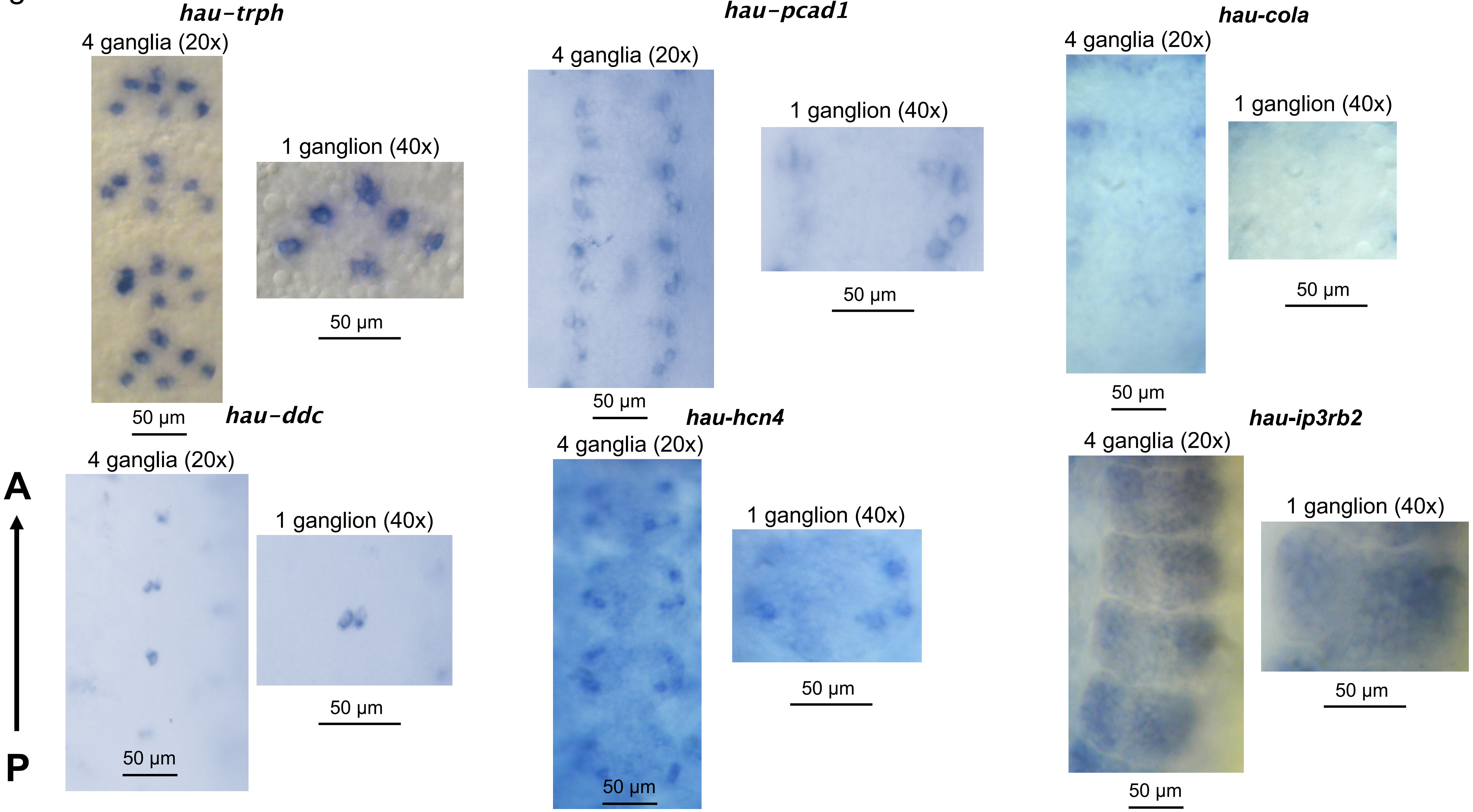
hau-trph
4 ganglia (20x)
1 ganglion (40x)
50 µm
50 µm
hau-pcad1
4 ganglia (20x)
1 ganglion (40x)
50 µm
50 µm
hau-cola
4 ganglia (20x)
1 ganglion (40x)
50 µm
50 µm
hau-ddc
4 ganglia (20x)
1 ganglion (40x)
50 µm
50 µm
A
P
hau-hcn4
4 ganglia (20x)
1 ganglion (40x)
50 µm
50 µm
hau-ip3rb2
4 ganglia (20x)
1 ganglion (40x)
50 µm
50 µm

Figure S4

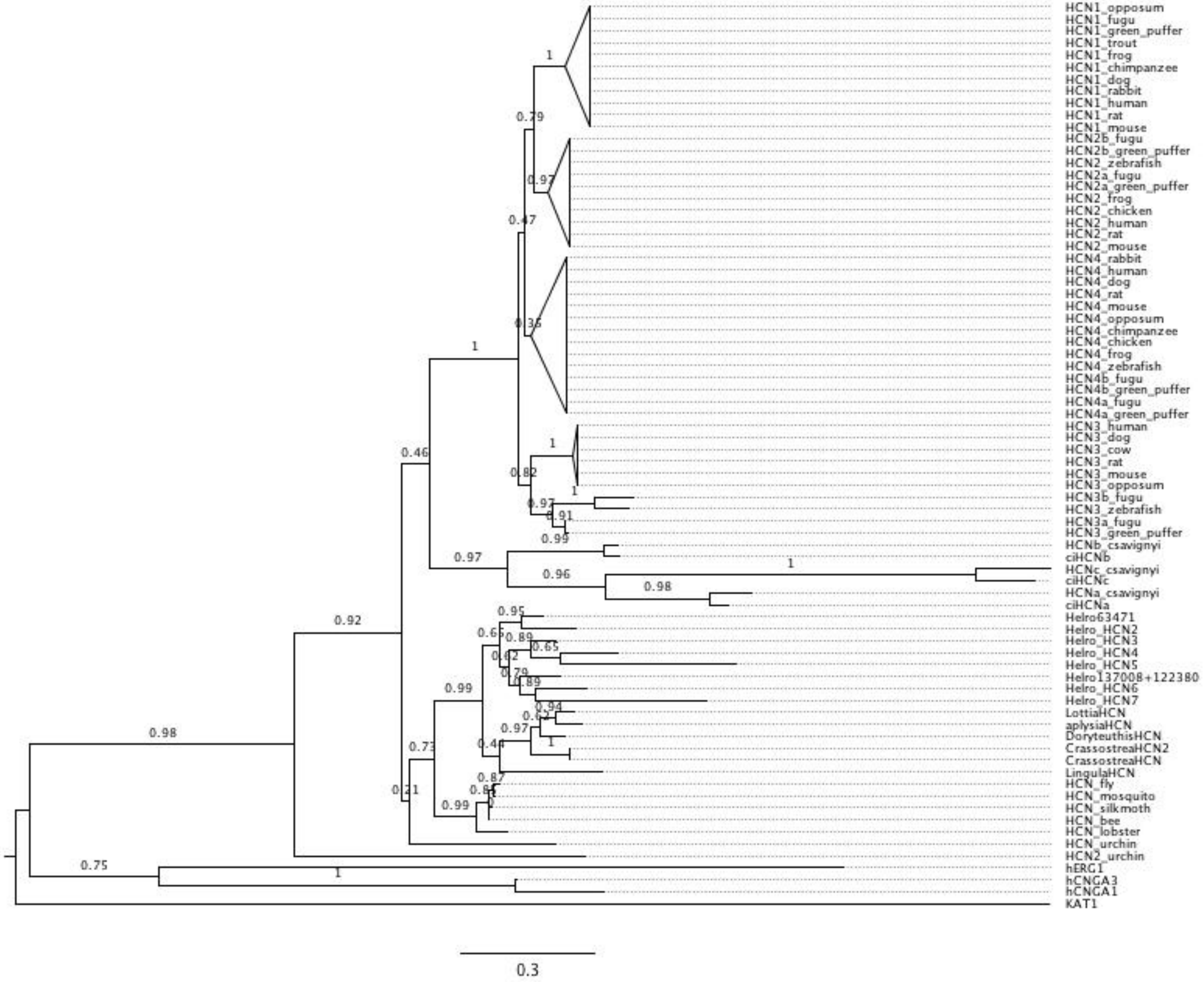
HCN1_opposum
HCN1_fugu
HCN1_green_puffer
HCN1_trout
HCN1_frog
HCN1_chimpanzee
HCN1_dog
HCN1_rabbit
HCN1_human
HCN1_rat
HCN1_mouse
HCN2b_fugu
HCN2b_green_puffer
HCN2_zebrafish
HCN2a_fugu
HCN2a_green_puffer
HCN2_frog
HCN2_chicken
HCN2_human
HCN2_rat
HCN2_mouse
HCN4_rabbit
HCN4_human
HCN4_dog
HCN4_rat
HCN4_mouse
HCN4_opposum
HCN4_chimpanzee
HCN4_chicken
HCN4_frog
HCN4_zebrafish
HCN4b_fugu
HCN4b_green_puffer
HCN4a_fugu
HCN4a_green_puffer
HCN3_human
HCN3_dog
HCN3_cow
HCN3_rat
HCN3_mouse
HCN3_opposum
HCN3b_fugu
HCN3_zebrafish
HCN3a_fugu
HCN3_green_puffer
HCNb_csavignyi
ciHCNb
HCNc_csavignyi
ciHCNc
HCNa_csavignyi
ciHCNa
Helro63471
Helro_HCN2
Helro_HCN3
Helro_HCN4
Helro_HCN5
Helro137008+122380
Helro_HCN6
Helro_HCN7
LottiaHCN
aplysiaHCN
DoryteuthisHCN
CrassostreaHCN2
CrassostreaHCN
LingulaHCN
HCN_fly
HCN_mosquito
HCN_silkmoth
HCN_bee
HCN_lobster
HCN_urchin
HCN2_urchin
hERG1
hCNGA3
hCNGA1
KAT1
0.3
