# Supplementary material for: Transcriptional profiling of identified neurons in leech
Source: BMC Genomics. 2021 Mar 25;22:215. doi: 10.1186/s12864-021-07526-0 (PMC7992859; doi:10.1186/s12864-021-07526-0)
Supplement: Supplementary file 7 — Additional file 7. [file 12864_2021_7526_MOESM7_ESM.docx]

**Supplementary File 6: Sequences used to generate the phylogenetic trees in this paper.**

**HCN (Figures 5 and S3):**

>Helobdella_HCN_98055

MAPQQFAPYTLRDHISSFFQVSDNKLAMKLFGNRTALMKEKQRQKEAGSWIIHPCSNFRFYWDLFMLVLLIANLIILPVIISFFHDDLSTKWIVFNSISDTVFLTDLVINFRTGIIADNFADEVILDPRKIAIRYLKTFFILDLVSSIPLDYIMLIFSQETAQSHFVHAGRALRIFRLLKLLSLLRLLRLSRLVRYVSQWQEFISVAGKFMRVFNLVCLMLLISHWNGCLQWLVPMLQEYPPNSWVALEELLNADWLEQYTWSLFKALSHMLCIGYGRYPPQSITDVWLTMVSMLIGATCYAMVVGHATTLIQSFDTSKRLYREKLKQVEEYTVYRKLPRNLRRRITTYYEHRYHGKMFDEKSILEELNECLKEEVINFNCRSLVASVPFFAQADPNFVSEIISKLEFEVYQPGDVIIREGTLGDKMYFIQEGVVDIYTGSGDRITTLFDGAFFGEICLLTNAKRVANVRAESYCNLFSLSVQHFNSVLKHYPVMRKTMEVVASERLEKIVKDGAVKVAEMPRIRSCVFEDVAADFNIVQETMKEKLVPENRKRLKTPTQSDEQSFHKSASECNFKRRSFHEKKPFTNIFDDKSYDNISINNINGLNNNNSNQHVNNDSNVFIEISKNAPVVKFDVAM

>Helro72757

MPRQYSSKQTLVTSAPTRQFSTPTTTHLRSNESVAECNINRETTTTIINSSVFSPDSTLLSNNDGECPQQQQQQSSQHESQQDVQKTGSYLKDQIISFFQVSDNKLALKLFGNRNALMKEKMRQKAVGNWIIHPCSSFRFYWDLFMLTLLLANLIILPVIISFFGDDLKTHWIVFNSVSDTVFLTDLLFNFRTGIIADNYADEVILEPRLIAKHYLKTFFLLDLISSLPLDYIIILLTPDTNTTQFYHAGWALRMVRLVKLLSLLRLLRLSRLVRYVSQWEEFLKQFLTVAEKFMRIFNLVSLMLLIGHWNGCLQWLVPMLQDFPSDSWAALEELQNADWSEQYTWALFKALSHMLCIGYGRYPPQSTTDVWLTMVSMLTGATCYAMFVGHATTLIQSFDTSKRMYREKFKQVEEYMAYRKLPRNLRQRITYYYEHRYQGKMFDEKNILAELNECLKEEIINYNCRALVASVPFFTHADVNFVSEVITKLEFEVFQPGDFIIKEGTMGTKMYFIQEGVVDIITKEGQIATSLSDGSYFGEICLLANTKRVASVRAETYCNLFSLSSTHFNSVLEHYPVMRSTMESVAAQRLNKIGRDPYIFLRRKQESDGTGFDVGRFYLKFQQRFLYITIGNKIAILCLLCPCYVRMKKL

>Helro77257

MKGSFSFKELSKSGALSTSTSYLKEQITSVFQPSDNKLAMKLFGNKMALKKEKERQEAVGKWVIHPCSNFRFYWDFIMLIFLIANLISLPVTITFFHDDLPNTWITFNAVSDTIFLMDLIINFRTGYITNTTTNYFTGIIADNFADEIILEPHHIAVHYLKTWFLLDFLSSIPLDHVIVLFTPDAEGISQIIHAGRALRFFRLVKLLSLLKLLRLSRLVRYVSQWEEVSLFISVAGKFIRIFNLISFILLLGHWNGCLQWLVPMLQDFPSNSWPALEELQNVHWLEQYTWSLFKALSHMLCIGFGRFPPQSSTDVWLTMVSMLTGATCYALFVGHSTTIIQSFDTAKRLYREKFKQVEEYMMYRKTPKHLRQKITDYYQHRYRGKMFDEKSILSEVNECLREDIINYNCRSLVAAVPFFTHADPNFVTDVVTKLEYEVYQPGDYVIKESTIGTKMYFIQEGIVDIVTKDGDVATSLSDGSYFGEICLLTNSRRCASVRCETYCNLFSLSVENFNSVLDHYPLMRRTLESVAAERLNKIGSSSSSFSSVFV

>Capitella63272

LDLISSIPLDYIILLFSPRHNFNILPFSFAGRALRIFRLVKLLSLLRLLRLSRLVRYVSQWEEFLTVASK

FMRILNLVALMLLLGHWNGCLQWLISLRCNFEHYFDSLISCGMVIRAHWSEQYTWALFKAMSHMLCIGYG

KFPPQSTTDVWLTMISMLTGATCYALFVGHATTLIQSFDTSKRLYREKFKQVEEYMMYRKLPRNLRQRIT

DYYEHRYQGKMFDEDSILGELNECLREEIVNYNCRALVASVPFFTHADPNFVSEIITQLKYEVFQPGDYV

IKEGTIGTKMYFIQEGIVDIVTKNGEVATSLSDGSYFGEICLLTNAKRVASVRVETYCNLFSLSVEHFNS

VLEHYPLMRRTMESVAAERLNKIGKNPNMV

>CrassostreaHCN

MSSLRNPSGHPLINISEYDRPVSYSSDEESSNPTSPYHQARNQQGALSDRSKESENEELLSENESEFQLHVEHVATLSPKPTRSDSVKLKRQSNGRQYRKNSQPIGSATPPKPARRSASLKLKRMHLDVNNQSKERTMPCLPKCSNRPTNPSITITIESDDDSIYSDYLNPDVNYKNEINRVHFVGDETSLYGTPREELLPPTGDTASNDLYNNKSSPTSFLKEQIISFFQPSDNKLAMKLFGNKNSLQREKMRHKRVGNWVIHPCSNFRFYWDLFMLVLLLANLIILPVAISFFNNDLSEEWIAFNCISDTVFFLDIIINFRTGIILNDFADEIILDPKLIAKHYVKSWFFLDLISTIPMDYIFLWWDAEADFYQMVHAGRALRILRLAKLLSLLRLLRLSRLVRYVQQWEEFLAVAGKFMRIFNLICLMLLLGHWNGCLQFLVPMLEDFPRDCWVSIEELKNASWWTQYTWALFKALSHMLCIGYGRFPPQNMTDTWLTILSMLSGATCYALFLGHTTTIIQSFDTSRRLYNEKFKQVEEYMIYRKLPRKLRQKISEYYEHRYQGKMFDEDNILDELNEVLRQEVINHNCRALVKSVPFFTNADPQFVSAVISKLEFEVFQPGDYVIREGTMGNKMYFIQEGIVDIITKDNEIATSLSDGSYFGEICLLTNAKRVASVRAETYVNLYSLSVQHFNSVLERYPLMRRTMESVAAERLTKIGKNPSIVSSRADLEEDQKLVNEIVMESTPVVTSASEDEDKDSDDSSESSKSSRRKKKFKFDFTGKLQKITEERKSKSRESLHKDSLDMPNGKMEKLRKMPSGSNLFGLRVGFAGFSDRKRSESVGANLNSIEHHSGSGQSGQSYLPRRASFLGTKFFKPKEPKKEKKERTRSTGGNDSPSSDGKGGVIQFLTVPLQADLRHKLKGKKSKSEHSVSQKEPPTNMHSQDIIEESPLNVQSGSDSIPETVVHQTDRAKECVYQIEDIDESSPSSHL

>CrassostreaHCN2

MPCLPKCSNRPTNPSITITIESDDDSIYSDYLNPDVNYKNEINRVHFVGDETSLYGTPREELLPPTGDTASNDLYNNKSSPTSFLKEQIISFFQPSDNKLAMKLFGNKNSLQREKMRHKRVGNWVIHPCSNFRFYWDLFMLVLLLANLIILPVAISFFNNDLSEEWIAFNCISDTVFFLDIIINFRTGIILNDFADEIILDPKLIAKHYVKSWFFLDLISTIPMDYIFLWWDAEADFYQMVHAGRALRILRLAKLLSLLRLLRLSRLVRYVQQWEEFLAVAGKFMRIFNLICLMLLLGHWNGCLQFLVPMLEDFPRDCWVSIEELKNASWWTQYTWALFKALSHMLCIGYGRFPPQNMTDTWLTILSMLSGATCYALFLGHTTTIIQSFDTSRRLYNEKFKQVEEYMIYRKLPRKLRQKISEYYEHRYQGKMFDEDNILDELNEVLRQEVINHNCRALVKSVPFFTNADPQFVSAVISKLEFEVFQPGDYVIREGTMGNKMYFIQEGIVDIITKDNEIATSLSDGSYFGEICLLTNAKRVASVRAETYVNLYSLSVQHFNSVLERYPLMRRTMESVAAERLTKIGKNPSIVSSRADLEEDQKLVNEIVMESTPVVTSASEDEDKDSDDSSESSKSSRRKKKFKFDFTGKLQKITEERKSKSRESLHKDSLDMPNGKMEKLRKMPSGSNLFGLRVGFAGFSDRKRSESVGANLNSIEHHSGSGQSGQSYLPRRASFLGTKFFKPKEPKKEKKERTRSTGGNDSPSSDGKGGVIQFLTVPLQADLRHKLKGKKSKSEHSVSQKEPPTNMHSQDIIEESPLNVQSGSDSIPETVVHQTDRAKECVYQIEDIDESSPSSHL

>LottiaHCN

MPCLQSGTANPSITITLDSDSDSVYSDYLSPEINYKNDVRVQFIGDDTSLYGTPKEELLPPSQETNNAVEIKPSPTSYLKDQILYFFQPSDNKLAMKLFGNKNALLKEKMRHKRVGNWVIHPCSNFRFYWDLVMLVLLIGNLIILPVAISFFNDDLSTHWIVFNCISDTVFFLDIIINFRTGVILNDFADEIILDPKLIAKHYMKTWFFLDLLSSIPMDYIFLMWDAEADFSQLFHAGRALRMLRLVKLLSLLRLLRLSRLVRYVQQWEEVCFLAIAGKFMRIFNLICLMFLLGHWNGCLQFLIPMLQDFPKDCWVSIEELQDAHWAEQYTWSLFKALSHMLCIGYGRFPPQNMSDTWLTILSMLSGATCYALFLAHTTTLIQSFDTSRRLYNEKFKQVEEYMIYRKLPRSLRQRITDYYEHRYQGKMFDEETIHRELNECLRQEVINHNCRALVASVPFFTNADPEFVSEVVSKLEFEVYQNGDYIIKEGTIGTKMYFIQEGIVDIITSDNEVATSLSDGSYFGEICLLTNARRVASVRAETYVNLYSLSVHHFNAVLDRYPVMRRTMESVAAERLTKIGKNPSIVSSRADLEEDQKLVNEIV

>LingulaHCN

MASTPQRQLSQDSYKESTGSPCSGRTAYTTASSAPTSKSETVFGSVTDSSCNTPGSVATTTKVTLEFDESCEVTEETTIPLTTEPNTLQQLPDRNESKSKTLLQRLPSLRLNRFGSGKSKNKSPAINLINDEEQGSESPPVSVKEKTKSEPFFIGDDASLYGTPKEELLPKSDTRSNTPSTTNFLKDQILTFFQPSDNRLAMKLFGNKSALIKERQRQRATGTWIIHPCSNFRFYWDLFMLLMLIANLIILPVAISFFNDDLSIHWVIFNCASDTVFLLDIFINFRTGVLDADYADEIILEPKKIAISYIKSWFFLDLISSLPLDYIFLMFDPNTPDVNQLMQAGRALRILRFAKLLSLLRLLRLSRLVRYVSQWEEFLSVAGVVVRIFNLICLMLLLGHWNGCLQYLVPMLQEFPPDCWVAIEELTKADWKEQYTWALFKALSHMLCIGYGRWPPQNVTDVTLTIISMLTGATCYALFVGHATTLIQSFDTGRRLFREKYKQVEEYMSYRRLPRDLRGKIADFYEHKYRGKMFDEDAILNDLSETLRESVVNHNCRALVANVPFFTNADPHFVTEVISKLKYEVCQPKDFIIKEGTRGDKMYFIQEGIVDIITKDGEVATSLSDGSYFGEICLLTNAKRVASVYAETYCNLFSLSVEDFNKILEHYPLMRRTLESVAAQRLSKIGKDANMMSTVQEEDVKAMNQIIQESASLRSDTGSNSPDEKRRESKKYRSSEFLEVDERPHKQKKKRLSSIFKAPFLLEFKTKKDRSDTTCGEVHEEETSDIDDVDDEYDGSDGHSNSKCKGTSGGTSPLLSVPQGTLSTYHSVPSFYSPSTDKVNGQVAYVNVDVEEEEETADATANVAEKESLV

>aplysiaHCN

MGQECVAGEAGGAASKASSTTSYLKDQILNFFQPSDNKLAMKLFGNKNALIKEKMRHKRVGNWVIHPCSNFRFYWDLFMLVLLIANLIILPVAISFFNDDLSTHWIVFNCISDTVFFLDIVINFRTGIILNDFADEIILDPKLIAKQYMKTWFFLDLLSSVPMDYIFLMWDAEADFNQLFHAGRALRMLRLAKLLSLLRLLRLSRLVRYVQQWEEFLAIAGKFMRIFNLICLMFLLGHWNGCLQFLVPLIQDFPKDCWVSIEGLQEAHWAEQYTWALFKALSHMLCIGYGRFPPQNMSDTWLTILSMLSGATCYALFLAHTTTLIQSFDTSRRLYNEKFKQVEEYMVYRKLPRSLRQRITDYYEHRYQGKMFDEETILSELNECLKHEVVNHNCRSLVASVPFFTNADPAFVSEVVSKLKFEVYQPGDYIIREGTMGTKMFFIQEGIVDIITSDGEVATSLSDGSYFGEICLLTNARRVASVRAETYACLYSLAVEHFTAVLERYPVMRRTMESVAAERLTKIGKNPSIVSSRADLEEDQKMVNEIVMESTPIPTSASEDEDRDSDESSDGSKQKKKTAFKFDFSTKLHKISEEKKNKSPKEHSKERDLLEFGETKHHRLSQGPDP

>DoryteuthisHCN

MPCLPQNNPNPSITITIDSDDESVYSDYLSPEINYKSDARVQFIGDDTSLYGTPKEELLPCREENVTVENTKSSPTTFLKDQIYSFFQPSDNKLAMKLFGNKNALYKEKMRHKRVGNWVIHPCSNFRFYWDLFMLVLLIANLIILPVAISFFNDDLSTHWIVFNCISDTVFFLDIIINFRTGVITNDFADEIILDPKLIAKHYIRTWFFLDLISSIPMDYIFLMWDTEANFSQLFHAGTNARMDASQYWQGRALRMLRLAKLLSLLRLLRLSRLVRYVQQWEEFLALAGKFMRIFNLICLMFLLGHWSGCLQFLVPMLQDFPQDCWVSIEELQKAHWAEQYTWALFKALSHMLCIGYGRFPPQNMTDTWLTILSMLSGATCYALFLGHTTTLIQSFDTSRRLYNEKFKQVEEYMVCRKLPRTLRQRISDYYEHRFQGKMFDEDTILGELNECLRQEVINHNCRSLVASVPFFTNADPQFVSEVVSRLKYEVFQPGDYIIREGTIGTKMYFIQEGIVDIITKEGEVATSLSDGSYFGEICLLTNAKRVASVRSETYVNLYSLSVEHFNAVLDNYPVMRRTMESVAAERLSKIGKNPSIVSSRADLEEDQKLVNEIVMESTPIPTSASEDEDRESDDSYETKPKKKFKFDFPLTLQKITEERKSKSKENLARMAGEDGDDYRPSNIFSRLPKVHSGSNLFGLRVPQMPDRKRTGSCGDVLGIINETSKDMKEMKDEKLSPETKAKDDKEKDDKEEKEKDKAKEKEERDKAKEKEDKDKDKEKDHHHHHEKEKEKEKHHHHHHHSHKDEKDKSRERKPILKQTSAEDKKEHVSADEDSSNEKRKPGYLEKKFKSADSSGSSREHKARKYRERKAPSLDERSVTFETRPSMQYLKIPGESIPSLDKTKLKSSDSSKEHRPRSRSVDIKGKTLDQKSEKKEEDKEKEKEKEKDDKEKEKEKDR

>HCN_fly

MPALSAVAARQQQLLLNGSLKGKGQSQSQGQSRQTLPGHRASVRSESGSGSSHTIPATGKSPPVPHSLAAKISSSASGSKNCNLLSASSNSCHKLNAHAQGSGAGSGSGSGSGSGPPGHSHYAAASPKSSVSSNGHLNKYCLTDLTRRKAEFNRQLSAPTDYTHHSSSNGSQQEGSSEANEGHEPVGESTITVASAGVSYPHPYSYPYHYAHHASSATAPANLKASLQLHSFGSHHPCPYPARPTSTSCTNSFNRRHIRRHKGKLGDRLLSGDSEESVRCSYCSVLNVNDNDLRISFENTCTDSLVTAFDDEALLICDQGTEMVHFDDVSLYGTPKEEPMPNIPIVSEKVSANFLKSQLQSWFQPTDNRLAMKLFGSRKALVKERIRQKTSGHWVIHPCSSFRFYWDLCMLLLLVANLIILPVAISFFNDDLSTRWIAFNCLSDTIFLIDIVVNFRTGIMQQDNAEQVILDPKLIAKHYLRTWFFLDLISSIPLDYIFLIFNQIMKLQDFSDSFQILHAGRALRILRLAKLLSLVRLLRLSRLVRYVSQWEEVYFLNMASVFMRIFNLICMMLLIGHWSGCLQFLVPMLQGFPSNSWVSINELQESYWLEQYSWALFKAMSHMLCIGYGRFPPQSLTDMWLTMLSMISGATCYALFLGHATNLIQSLDSSRRQYREKVKQVEEYMAYRKLPRDMRQRITEYFEHRYQGKFFDEELILGELSEKLREDVINYNCRSLVASVPFFANADSNFVSDVVTKLKYEVFQPGDIIIKEGTIGTKMYFIQEGVVDIVMANGEVATSLSDGSYFGEICLLTNARRVASVRAETYCNLFSLSVDHFNCVLDQYPLMRKTMETVAAERLNKIGKNPNIMHQKDEQLSNPESNTITAVVNALAAEADDCKDDDMDLKENLLHGSESSIAEPVQTIREGLPRPRSGEFRALFEGNTP

>HCN_mosquito

MDPCALSVRQYPAPQSAGTSRLFSAASSDSIRFHSNLSNQNDTDLRVSIDNTCTDSLVTALDDEALLITDYMNDMAKSKHFDDVSLYGTPKEEPLPNIPPSIEKPSSNFLKNQLQAWFQPTDNRLAMKLFGSKKALVKERIRQKTAGHWVIHPCSSFRFYWDLCMLLLLVANLIILPVAISFFNDDLSTRWIAFNCLSDTIFLVDIVVNFRTGIMQQDNAEQVILDPKLIAKHYLKTWFFLDLISSIPLDYIFLIFNQDFSDSFQLLHAGRALRILRLAKLLSLVRLLRLSRLVRYVSQWEEVYFLNMASVFMRIFNLICMMLLIGHWSGCLQFLVPMLQGFPSNSWVAINELQESYWLEQYSWALFKAMSHMLCIGYGRFPPQSLTDMWLTMLSMISGATCYALFLGHATNLIQSLDSSRRQYREKVKQVEEYMAYRKLPRDMRQRITEYFEHRYQGKFFDEECILGELSEKLREDVINYNCRSLVASVPFFANADSNFVSDVVTKLRYEVFQPGDIIIKEGTIGSKMYFIQEGIVDIVMANGEVATSLSDGSYFGEICLLTNARRVASVRAETYCNLFSLSVDHFNAVLDQYPLMRKTMETVAAERLNKIGKNPNIMAQKEESESGNTETNQISAVVNALAAEADNVDNLSDGNGSMKKGTSQSSLNELNQSLKMSLPRPKSGEFRALFEG

>HCN_bee

MNYKGSGKVHFGVDDVSLYGTPKEEPGPGLPGQEVKQSFLKNQLQALFQPTDNKLAMKLFGSKKALMKERIRQKAAGHWVIHPCSSFRFYWDLCMLLLLVANLIILPVAISFFNDDLSTRWIAFNCLSDTIFLIDIVVNFRTGIMQQDNAEQVILDPKLIAKHYLRTWFFLDLISSIPLDYIFLIFNQFQDFSESFQILHAGRALRILRLAKLLSLVRLLRLSRLVRYVSQWEEVYFLNMASVFMRIFNLICMMLLIGHWSGCLQFLVPMLQGFPSNSWVAINELQDSFWLEQYSWALFKAMSHMLCIGYGRFPPQSLTDMWLTMLSMISGATCYALFLGHATNLIQSLDSSRRQYREKVKQVEEYMAYRKLPREMRQRITEYFEHRYQGKFFDEELILGELSEKLREDVINYNCRSLVASVPFFANADSNFVSDVVTKLRYEVFQPGDIIIKEGTIGSKMYFIQEGIVDIVMANGEVATSLSDGSYFGEICLLTNARRVASVRAETYCNLFSLSVDHFNAVLDQYPLMRRTMESVAAERLNKIGKNPNLVAHREEDLGSESKTINAVVNALAEQAAHASASEESVHSMELRTLPCLLPRPKSENNFASQELSREGRRIFHKSDTFHKDSYQ

>HCN_silkmoth

MDNTNTCCTDSLVTALDDETLLLGDADMSLKQGSGTGKVHFGGLDDVSLYGTPVEPAPPATDAKQGFLRNQLQALFQPTDNKLAMKLFGSKKALMKERIRQKAAGHWVIHPCSSFRFYWDLCMLLLLVANLIILPVAISFFNDDLSTRWIAFNCLSDTIFLIDIVVNFRTGIMQQDNAEQVILDPKLIAKHYLRTWFFLDLISSIPLDYIFLIFNQDFSESFQILHAGRALRILRLAKLLSLVRLLRLSRLVRYVSQWEEVYFLNMASVFMRIFNLICMMLLIGHWSGCLQFLVPMLQGFPPNSWVAINELQEAYWLEQYSWALFKAMSHMLCIGYGRFPPQSLTDMWLTMLSMISGATCYALFLGHATNLIQSLDSSRRQYREKVKQVEEYMAYRKLPREMRQRITEYFEHRYQGKFFDEELILGELSEKLREDVINYNCRSLVASVPFFANADSNFVSDVVTKLRYEVFQPGDIIIKEGTIGSKMYFIQEGIVDIVMANGEVATSLSDGSYFGEICLLTNARRVASVRAETYCNLFSLSVDHFNAVLDQYPLMRRTMESVAAERLNKIGKNPNLVTHREDDTTSEGNTINAVVNALAAEAEHVSLSDDSVARLSERSLGLALQPLQAASCRMAGVALPGLGVAAALPRPKSEHDFSAAAPLASAAAFHKSDAGIAP

>HCN_lobster

MNYRDVSKVHFGGDDVSLYGTPKEELGPGQLCVGAAGAPPGVEPKPSFLKNQLQALFQPTDNRLAMKLFGSKKALMKERIRQKAAGHWIIHPCSNFRFYWDLCMLFLLVANLIILPVAISFFNDDLSTRWIAFNCLSDTIFLIDIVVNFRTGIKQQDNSEQVILDPKLIARHYLKTWFLLDLISSVPLDYIFLIFNKDFNESFQILQAGRALRILRLAKLLSLVRLLRLSRLVRYVSQWEEVYVSSFLNMASVFMRIFNLISMMLLIGHWSGCLQFLVPMLQGFPSNSWVAINELQSSHWLEQYSWAFFKAMSHMLCIGYGSFPPQNLTDLWLTMISMISGATCYALFIGHATNLIQSLDSSRRQYRERLKQVEEYMAYRKLPRELRTRITEYFEHRYQGKFFDEEMILGELSEKLREDVINFNCRALVASVPFFANADARFVTDVVTKLRYEVYQPGDIIIKEGTIGNKMYFIQEGIVDIVMSNGEVATSLSDGSYFGEICLLTNARRTASVRAETYCNLFSLSVEHFNTVLDSYPLMRRTMESVAAERLNKIGKNPSIVSNREDLTNDCKTVNAIVNALASVASTEQCDGGTSSEESMMGHDGSSMKGGGGGGGGRHHHHHHHHHNLLDLGSIGKALAKGHLPRPKSENNFALSLDTPSPLNRNRPSFHKSDTFHKALRSNSRAPPPMH

>HCN_urchin

MDNKETNGELEQSDEADPSGQNLDDGETDSKQEENLINVSPPKTPPGPPPPLKNGGRGQKPPKIPICHQNGKLPKEVEWTEDRGEDRKDSLTLQSKLDHGAYTDEKQDLLTYLDRHGINSPVKLTPDETGGSSALDILGIIEERDTGALGSDPSSTMQAMAKPVGFLQRQLWTVLQPSDNRLSMKLFGSKKGLQKEKYRLRKAGVLIIHPCSHFRFYWDLLMLCLIMANVILLPVVITFFHNKDMSTGWLIFNCFSDTFFILDLICNFRTGIMNPKSAEQVILNPRQIAYHYLRSWFIIDLVSSIPMDYIFLLAGGQNRHFLEVSRALKILRFAKLLSLLRLLRLSRLMRFVSQWEQAFNVANAVIRICNLVCMMLLIGHWNGCLQYLVPMLQEYPDQSWVAINGLEHAHWWEQYTWALFKALSHMLCIGYGKFPPQSITDVWLTIVSMVSGATCFALFIGHATNLIQSMDSSSRQYREKLKQVEEYMQYRKLPSHLRNKILDYYEYRYRGKMFDERHIFREVSESIRQDVANYNCRDLVASVPFFVGADSNFVTRVVTLLEFEVFQPADYVIQEGTFGDRMFFIQQGIVDIIMSDGVIATSLSDGSYFGEICLLTRERRVASVKCETYCTLFSLSVQHFNQVLDEFPAMRKTMEEIAVRRLTRIGKESSKLKSRLESPTIRDTAPLFPIPPDTPSFVTDIEKNRFFGDDTDDVHIRTRVDVERGSHENVIAIMDGSLSDLRMENEIQARKSSSGKRRKFQQQTTEL

>HCN2_urchin

MSTAMANKVMPDTQGEFVKVKRPDRLSKISALRKDFDVKKAAVLKEKPKERRASLPSAMTDKSPGHRDSLVVDLGGAGRRNSMPTKESLFLASGSDFVKKIYRSEKALKDEQERQQNIRRFVIHPFSNFRWYWDLFMVFLLLITLVLLPVNVTFFSDDITMYWIINCISDTLFMLDITLNFFTGVIENAEDTVTLDRTKEEFSSLRGWFFLDLISSFPFDYIYLFLGQVDFTSHTALALRILRLTKVLSLLRLLRLSRLLRYIHRPEELLNVETAVIRIVHLVFVMLLLMHWNGCIQFLVPFFQTFPPDCWVVINGLENAGKLEQYSWSFFKAICHMISIGFGRFPPMNVTEMWMTTFSIMLGATFYALFIGTMSTLLLAVDASGRLYNERLNQVKEYLRYRKVPMNTQRRVLSYYEHRYQRKYFNEKTILGEQSHPIRREILQHHFNNFITKVNFLNEADPDFAYDVIEKLSFEVFLEGDVIIKAGSLGGAMYFIEHGTVEVLVDDRIVNRLSDGDHFGEISLLIDERRVASIIAATTCDVFCLSREDFHKVLKDYPEMGARMAEIAQERLNTIDSSLDTVDEEADGDEEEATANNNNNHPAKNDKKVKKNDLATPQNKDHIQKINDWLKENFAQRI

>HCN1_trout

MEDKSNSFSSNKEGEKADGNNVFQRQDSIQKNNMGSQNMKGGDHGNSVGFKGDREEALVGFDDIDGSGNRHGFMQRQFGAMMQPGVNKFSLRMFGSQKAVEKEQERVQTAGYWIIHPYSDFRFYWDLVMLVMMMGNLIIIPVGITFFSEQTTTTWLIFNVASDTIFLVDLVMNFRTGIVNEESSEIILDPKVIKMNYLKSWFVVDFLSSIPVDYIFLIVEKGFDSEVYKTARALRIVRFTKILSLLRLLRLSRLIRYIHQWEEIFHMTYDLASAVVRIFNLIGMMLLLCHWDGCLQFLVPLLQDFPQDCWVSLNGMVNDSWGKQYSYALFKAMSHMLCIGYGARAPVSMSDLWITMLSMIVGATCYAMFVGHATALIQSLDSSRRQYQEKYKQVEQYMSFHKLPADMRQKIHDYYEHRYQGKIFDEDNILSELNDPLKEEIVNFNCRKLVATMPLFANADPNFVTGMLSKLKFEVFQPNDYIIREGTVGKKMYFIQHGVASVITKLNKEMKLTDGSYFGEICLLTKGRRTASVRADTYCRLFSLSVDHFNEVLEEYPMMRRAFETVAIDRLDRIGKKNSLLLQKFQKDLNAGVFNTQENEILKQIIRQDREMVMMVDRKQSVTGMSVTGMSVTGMNTTPISGNSIINSPAQPPYTTALGNNQFQQSATSLTYSASAVTAPSSAATARILPASAQGVYPVPSVIHGNLNSSSPVPQTPLSLHQQGSIMSPVSFTTAVCSPPVQTPGLAGRSFQYGSPTASQLSLIQQPLPTALPPQQPLPQPQQPGGAAASSATQQPQQQQQVPSPQRSDSLHKASHALQSGSLSRDVRHLSASQPSLPHDTSLGPRAHPAASGDSLASIAPPVAAVQGMGIQSGLRTTVPQRVNLFRQMSSGALPPVRAVSSAAQHRDSTGSRRDSRRDSTLSSTETEQDKMRFASNL

>HCN1_rabbit

MATASSPPRRPRRARGLEDAEGPRRQYGFMQRQFTSMLQPGVNKFSLRMFGSQKAVEKEQERVKTAGFWIIHPYSDFRFYWDLIMLIMMVGNLVIIPVGITFFTEQTTTPWIIFNVASDTVFLLDLIMNFRTGTVNEDSSEIILDPKVIKMNYLKSWFVVDFISSIPVDYIFLIVEKGMDSEVYKTARALRIVRFTKILSLLRLLRLSRLIRYIHQWEEIFHMTYDLASAVVRIFNLIGMMLLLCHWDGCLQFLVPLLQDFPPDCWVSLNEMVNDSWGKQYSYALFKAMSHMLCIGYGAQAPVSMSDLWITMLSMIVGATCYAMFVGHATALIQSLDSSRRQYQEKYKQVEQYMSFHKLPADMRQKIHDYYEHRYQGKIFDEENILNELNDPLREEIVNFNCRKLVATMPLFANADPNFVTAMLSKLRFEVFQPGDYIIREGAVGKKMYFIQHGVAGVITKSSKEMKLTDGSYFGEICLLTKGRRTASVRADTYCRLYSLSVDNFNEVLEEYPMMRRAFETVAIDRLDRIGKKNSILLQKFQKDLNTGVFNNQENEILKQIVKHDREMVQAIAPISYPQMTALNSTSSTATPTSRMRTQSPPVYTATSLSHSNLHSPSPSTQTPQPSAILSPCSYTTAVCSPPVQSPLATRTFHYASPTASQLSLMPQQQQQPQAPQTQPQQPPQQPQTPGSATPKNEVHRSTQALPNTSLTREVRPLSASQPSLPHEVSTLISRPHPTVGESLASIPQPVAAVHSAGLQAAGRSTVPQRVTLFRQMSSGAIPPNRGVPPAPPPPAAPLQREASSVLNTDPEAEKPRFASNL

>HCN4_rabbit

MDKLPPSMRKRLYSLPQQVGAKAWIMDEEEDAEEEGAGGRQDPRRRSIRLRPLPSPSPSPSAAAAAAGGAESRGAALGGAADGEGPARGAAKSSTNGDCRRFRGSLASLGSRGGGGGGGSTGGGSHGHLHDSAEERRLIAEGDASPGEDRTPPGLAAEPERPGAPAPPAASPPQVPSSCGEQRPADAAVKVEGGAAAGDQILPEAEARLGQAGFMQRQFGAMLQPGVNKFSLRMFGSQKAVEREQERVKSAGFWIIHPYSDFRFYWDLTMLLLMVGNLIIIPVGITFFKDENTTPWIVFNVVSDTFFLIDLVLNFRTGIVVEDNTDIILDPRRIKMKYLKSWFVVDFVSSIPVDYIFLIVETRIDSEVYKTARALRIVRFTKILSLLRLLRLSRLIRYIHQWEEIFHMTYDLASAVVRIVNLIGMMLLLCHWDGCLQFLVPMLQDFPDDCWVSLNNMVNNSWGKQYSYALFKAMSHMLCIGYGRQAPMGMSDVWLTMLSMIVGATCYAMFIGHATALIQSLDSSRRQYQEKYKQVEQYMSFHKLPPDTRQRIHDYYEHRYQGKMFDEESILGELSEPLREEIINFNCRKLVASMPLFANADPNFVTSMLTKLRFEVFQPGDYIIREGTIGKKMYFIQHGVVSVLTKGNKETKLADGSYFGEICLLTRGRRTASVRADTYCRLYSLSVDNFNEVLEEYPMMRRAFETVALDRLDRIGKKNSILLHKVQHDLSSGVSNYQENAIVQRIVQHDREMAHCARRAQATTPVAPAIWTPLIQAPLQAAAATTSVAIALTHHPRLPAAIFRPPPGPTTLGSLGAGQTPRHLRRLQSLAPSAPSPASPASSPSQPDTPSSASLHVQPLPGCSTPAGLGSLLPTAGSPPAPTPPTTAGAAGFSHFHRALGGSLSSSDSPLLTPMQSAARSPQQPPPPPGAPAGLGLLEHFLPPPARSPTSSPGQLGQPPGELSPGLGSGPPGTPETPPRQPERLPFAAGASAGASPVAFSPRGGPSPPGHSPGTPRTFPSAPPRASGSHGSLLLPPASSPPPPPPPPAPQRRATPPLAPGRLSQDLKLISASQPALPQDGAQTLRRASPHSSSGESVAALPPFPRAPGRPPGAGPGQHVTLTLPRKASSGSLPPPLSLFGPRAAPAGGPRLTAAPQREPGAKSEPVRSKLPSNL

>HCN4_human

MDKLPPSMRKRLYSLPQQVGAKAWIMDEEEDAEEEGAGGRQDPSRRSIRLRPLPSPSPSAAAGGTESRSSALGAADSEGPARGAGKSSTNGDCRRFRGSLASLGSRGGGSGGTGSGSSHGHLHDSAEERRLIAEGDASPGEDRTPPGLAAEPERPGASAQPAASPPPPQQPPQPASASCEQPSVDTAIKVEGGAAAGDQILPEAEVRLGQAGFMQRQFGAMLQPGVNKFSLRMFGSQKAVEREQERVKSAGFWIIHPYSDFRFYWDLTMLLLMVGNLIIIPVGITFFKDENTTPWIVFNVVSDTFFLIDLVLNFRTGIVVEDNTEIILDPQRIKMKYLKSWFMVDFISSIPVDYIFLIVETRIDSEVYKTARALRIVRFTKILSLLRLLRLSRLIRYIHQWEEIFHMTYDLASAVVRIVNLIGMMLLLCHWDGCLQFLVPMLQDFPDDCWVSINNMVNNSWGKQYSYALFKAMSHMLCIGYGRQAPVGMSDVWLTMLSMIVGATCYAMFIGHATALIQSLDSSRRQYQEKYKQVEQYMSFHKLPPDTRQRIHDYYEHRYQGKMFDEESILGELSEPLREEIINFNCRKLVASMPLFANADPNFVTSMLTKLRFEVFQPGDYIIREGTIGKKMYFIQHGVVSVLTKGNKETKLADGSYFGEICLLTRGRRTASVRADTYCRLYSLSVDNFNEVLEEYPMMRRAFETVALDRLDRIGKKNSILLHKVQHDLNSGVFNYQENEIIQQIVQHDREMAHCAHRVQAAASATPTPTPVIWTPLIQAPLQAAAATTSVAIALTHHPRLPAAIFRPPPGSGLGNLGAGQTPRHLKRLQSLIPSALGSASPASSPSQVDTPSSSSFHIQQLAGFSAPAGLSPLLPSSSSSPPPGACGSPSAPTPSAGVAATTIAGFGHFHKALGGSLSSSDSPLLTPLQPGARSPQAAQPSPAPPGARGGLGLPEHFLPPPPSSRSPSSSPGQLGQPPGELSLGLATGPLSTPETPPRQPEPPSLVAGASGGASPVGFTPRGGLSPPGHSPGPPRTFPSAPPRASGSHGSLLLPPASSPPPPQVPQRRGTPPLTPGRLTQDLKLISASQPALPQDGAQTLRRASPHSSGESMAAFPLFPRAGGGSGGSGSSGGLGPPGRPYGAIPGQHVTLPRKTSSGSLPPPLSLFGARATSSGGPPLTAGPQREPGARPEPVRSKLPSNL

>HCN2_human

MDARGGGGRPGESPGATPAPGPPPPPPPAPPQQQPPPPPPPAPPPGPGPAPPQHPPRAEALPPEAADEGGPRGRLRSRDSSCGRPGTPGAASTAKGSPNGECGRGEPQCSPAGPEGPARGPKVSFSCRGAASGPAPGPGPAEEAGSEEAGPAGEPRGSQASFMQRQFGALLQPGVNKFSLRMFGSQKAVEREQERVKSAGAWIIHPYSDFRFYWDFTMLLFMVGNLIIIPVGITFFKDETTAPWIVFNVVSDTFFLMDLVLNFRTGIVIEDNTEIILDPEKIKKKYLRTWFVVDFVSSIPVDYIFLIVEKGIDSEVYKTARALRIVRFTKILSLLRLLRLSRLIRYIHQWEEIFHMTYDLASAVMRICNLISMMLLLCHWDGCLQFLVPMLQDFPRNCWVSINGMVNHSWSELYSFALFKAMSHMLCIGYGRQAPESMTDIWLTMLSMIVGATCYAMFIGHATALIQSLDSSRRQYQEKYKQVEQYMSFHKLPADFRQKIHDYYEHRYQGKMFDEDSILGELNGPLREEIVNFNCRKLVASMPLFANADPNFVTAMLTKLKFEVFQPGDYIIREGTIGKKMYFIQHGVVSVLTKGNKEMKLSDGSYFGEICLLTRGRRTASVRADTYCRLYSLSVDNFNEVLEEYPMMRRAFETVAIDRLDRIGKKNSILLHKVQHDLNSGVFNNQENAIIQEIVKYDREMVQQAELGQRVGLFPPPPPPPQVTSAIATLQQAAAMSFCPQVARPLVGPLALGSPRLVRRPPPGPAPAAASPGPPPPASPPGAPASPRAPRTSPYGGLPAAPLAGPALPARRLSRASRPLSASQPSLPHGAPGPAASTRPASSSTPRLRPTPAARAAAPSPDRRDSASPGAAGGLDPQDSARSRLSSNL

>HCN1_human

MEGGGKPNSSSNSRDDGNSVFPAKASATGAGPAAAEKRLGTPPGGGGAGAKEHGNSVCFKVDGGGGGGGGGGGGEEPAGGFEDAEGPRRQYGFMQRQFTSMLQPGVNKFSLRMFGSQKAVEKEQERVKTAGFWIIHPYSDFRFYWDLIMLIMMVGNLVIIPVGITFFTEQTTTPWIIFNVASDTVFLLDLIMNFRTGTVNEDSSEIILDPKVIKMNYLKSWFVVDFISSIPVDYIFLIVEKGMDSEVYKTARALRIVRFTKILSLLRLLRLSRLIRYIHQWEEIFHMTYDLASAVVRIFNLIGMMLLLCHWDGCLQFLVPLLQDFPPDCWVSLNEMVNDSWGKQYSYALFKAMSHMLCIGYGAQAPVSMSDLWITMLSMIVGATCYAMFVGHATALIQSLDSSRRQYQEKYKQVEQYMSFHKLPADMRQKIHDYYEHRYQGKIFDEENILNELNDPLREEIVNFNCRKLVATMPLFANADPNFVTAMLSKLRFEVFQPGDYIIREGAVGKKMYFIQHGVAGVITKSSKEMKLTDGSYFGEICLLTKGRRTASVRADTYCRLYSLSVDNFNEVLEEYPMMRRAFETVAIDRLDRIGKKNSILLQKFQKDLNTGVFNNQENEILKQIVKHDREMVQAIAPINYPQMTTLNSTSSTTTPTSRMRTQSPPVYTATSLSHSNLHSPSPSTQTPQPSAILSPCSYTTAVCSPPVQSPLAARTFHYASPTASQLSLMQQQPQQQVQQSQPPQTQPQQPSPQPQTPGSSTPKNEVHKSTQALHNTNLTREVRPFSAWQPSLPHEVSTLISRPHPTVGESLASIPQPVTAVPGTGLQAGGRSTVPQRVTLFRQMSSGAIPPNRGVPPAPPPPAAALPRESSSVLNTDPDAEKPRFASNL

>HCN3_human

MEAEQRPAAGASEGATPGLEAVPPVAPPPATAASGPIPKSGPEPKRRHLGTLLQPTVNKFSLRVFGSHKAVEIEQERVKSAGAWIIHPYSDFRFYWDLIMLLLMVGNLIVLPVGITFFKEENSPPWIVFNVLSDTFFLLDLVLNFRTGIVVEEGAEILLAPRAIRTRYLRTWFLVDLISSIPVDYIFLVVELEPRLDAEVYKTARALRIVRFTKILSLLRLLRLSRLIRYIHQWEEIFHMTYDLASAVVRIFNLIGMMLLLCHWDGCLQFLVPMLQDFPPDCWVSINHMVNHSWGRQYSHALFKAMSHMLCIGYGQQAPVGMPDVWLTMLSMIVGATCYAMFIGHATALIQSLDSSRRQYQEKYKQVEQYMSFHKLPADTRQRIHEYYEHRYQGKMFDEESILGELSEPLREEIINFTCRGLVAHMPLFAHADPSFVTAVLTKLRFEVFQPGDLVVREGSVGRKMYFIQHGLLSVLARGARDTRLTDGSYFGEICLLTRGRRTASVRADTYCRLYSLSVDHFNAVLEEFPMMRRAFETVAMDRLLRIGKKNSILQRKRSEPSPGSSGGIMEQHLVQHDRDMARGVRGRAPSTGAQLSGKPVLWEPLVHAPLQAAAVTSNVAIALTHQRGPLPLSPDSPATLLARSAWRSAGSPASPLVPVRAGPWASTSRLPAPPARTLHASLSRAGRSQVSLLGPPPGGGGRRLGPRGRPLSASQPSLPQRATGDGSPGRKGSGSERLPPSGLLAKPPRTAQPPRPPVPEPATPRGLQLSANM

>HCN4_mouse

MDKLPPSMRKRLYSLPQQVGAKAWIMDEEEDGEEEGAGGRQDPSRRSIRLRPLPSPSPSVAAGCSESRGAALGATESEGPGRSAGKSSTNGDCRRFRGSLASLGSRGGGSGGAGGGSSLGHLHDSAEERRLIAAEGDASPGEDRTPPGLATEPERPATAAQPAASPPPQQPPQPASASCEQPSADTAIKVEGGAAAIDHILPEAEVRLGQSGFMQRQFGAMLQPGVNKFSLRMFGSQKAVEREQERVKSAGFWIIHPYSDFRFYWDLTMLLLMVGNLIIIPVGITFFKDENTTPWIVFNVVSDTFFLIDLVLNFRTGIVVEDNTEIILDPQRIKMKYLKSWFVVDFISSIPVEYIFLIVETRIDSEVYKTARAVRIVRFTKILSLLRLLRLSRLIRYIHQWEEIFHMTYDLASAVVRIVNLIGMMLLLCHWDGCLQFLVPMLQDFPHDCWVSINGMVNNSWGKQYSYALFKAMSHMLCIGYGRQAPVGMSDVWLTMLSMIVGATCYAMFIGHATALIQSLDSSRRQYQEKYKQVEQYMSFHKLPPDTRQRIHDYYEHRYQGKMFDEESILGELSEPLREEIINFNCRKLVASMPLFANADPNFVTSMLTKLRFEVFQPGDYIIREGTIGKKMYFIQHGVVSVLTKGNKETRLADGSYFGEICLLTRGRRTASVRADTYCRLYSLSVDNFNEVLEEYPMMRRAFETVALDRLDRIGKKNSILLHKVQHDLNSGVFNYQENEIIQQIVRHDREMAHCAHRVQAAASATPTPTPVIWTPLIQAPLQAAAATTSVAIALTHHPRLPAAIFRPPPGPGLGNLGAGQTPRHPRRLQSLIPSALGSASPASSPSQVDTPSSSSFHIQQLAGFSAPPGLSPLLPSSSSSPPPGACGSPPAPTPSTSTAAAASTTGFGHFHKALGGSLSSSDSPLLTPLQPGARSPQAAQPPPPLPGARGGLGLLEHFLPPPPSSRSPSSSPGQLGQPPGELSLGLAAGPSSTPETPPRPERPSFMAGASGGASPVAFTPRGGLSPPGHSPGPPRTFPSAPPRASGSHGSLLLPPASSPPPPQVPQRRGTPPLTPGRLTQDLKLISASQPALPQDGAQTLRRASPHSSGESVAAFSLYPRAGGGSGSSGGLGPPGRPYGAIPGQHVTLPRKTSSGSLPPPLSLFGARAASSGGPPLTTAAPQREPGARSEPVRSKLPSNL

>HCN2_mouse

MDARGGGGRPGDSPGTTPAPGPPPPPPPPAPPQPQPPPAPPPNPTTPSHPESADEPGPRARLCSRDSACTPGAAKGGANGECGRGEPQCSPEGPARGPKVSFSCRGAASGPSAAEEAGSEEAGPAGEPRGSQASFLQRQFGALLQPGVNKFSLRMFGSQKAVEREQERVKSAGAWIIHPYSDFRFYWDFTMLLFMVGNLIIIPVGITFFKDETTAPWIVFNVVSDTFFLMDLVLNFRTGIVIEDNTEIILDPEKIKKKYLRTWFVVDFVSSIPVDYIFLIVEKGIDSEVYKTARALRIVRFTKILSLLRLLRLSRLIRYIHQWEEIFHMTYDLASAVMRICNLISMMLLLCHWDGCLQFLVPMLQDFPSDCWVSINNMVNHSWSELYSFALFKAMSHMLCIGYGRQAPESMTDIWLTMLSMIVGATCYAMFIGHATALIQSLDSSRRQYQEKYKQVEQYMSFHKLPADFRQKIHDYYEHRYQGKMFDEDSILGELNGPLREEIVNFNCRKLVASMPLFANADPNFVTAMLTKLKFEVFQPGDYIIREGTIGKKMYFIQHGVVSVLTKGNKEMKLSDGSYFGEICLLTRGRRTASVRADTYCRLYSLSVDNFNEVLEEYPMMRRAFETVAIDRLDRIGKKNSILLHKVQHDLSSGVFNNQENAIIQEIVKYDREMVQQAELGQRVGLFPPPPPPQVTSAIATLQQAVAMSFCPQVARPLVGPLALGSPRLVRRAPPGPLPPAASPGPPAASPPAAPSSPRAPRTSPYGVPGSPATRVGPALPARRLSRASRPLSASQPSLPHGVPAPSPAASARPASSSTPRLGPAPTARTAAPSPDRRDSASPGAASGLDPLDSARSRLSSNL

>HCN1_mouse

MEGGGKPNSASNSRDDGNSVFPSKAPATGPVAADKRLGTPPGGGAAGKEHGNSVCFKVDGGGGEEPAGSFEDAEGPRRQYGFMQRQFTSMLQPGVNKFSLRMFGSQKAVEKEQERVKTAGFWIIHPYSDFRFYWDLIMLIMMVGNLVIIPVGITFFTEQTTTPWIIFNVASDTVFLLDLIMNFRTGTVNEDSSEIILDPKVIKMNYLKSWFVVDFISSIPVDYIFLIVEKGMDSEVYKTARALRIVRFTKILSLLRLLRLSRLIRYIHQWEEIFHMTYDLASAVVRIFNLIGMMLLLCHWDGCLQFLVPLLQDFPPDCWVSLNEMVNDSWGKQYSYALFKAMSHMLCIGYGAQAPVSMSDLWITMLSMIVGATCYAMFVGHATALIQSLDSSRRQYQEKYKQVEQYMSFHKLPADMRQKIHDYYEHRYQGKIFDEENILSELNDPLREEIVNFNCRKLVATMPLFANADPNFVTAMLSKLRFEVFQPGDYIIREGAVGKKMYFIQHGVAGVITKSSKEMKLTDGSYFGEICLLTKGRRTASVRADTYCRLYSLSVDNFNEVLEEYPMMRRAFETVAIDRLDRIGKKNSILLQKFQKDLNTGVFNNQENEILKQIVKHDREMVQAIPPINYPQMTALNCTSSTTTPTSRMRTQSPPVYTATSLSHSNLHSPSPSTQTPQPSAILSPCSYTTAVCSPPIQSPLATRTFHYASPTASQLSLMQQPQQQLPQSQVQQTQTQTQQQQQQQQQQQQQQQQQQQQQQQQQQQQQQQQQQQQQPQTPGSSTPKNEVHKSTQALHNTNLTKEVRPLSASQPSLPHEVSTLISRPHPTVGESLASIPQPVAAVHSTGLQAGSRSTVPQRVTLFRQMSSGAIPPNRGVPPAPPPPAAVQRESPSVLNTDPDAEKPRFASNL

>HCN3_mouse

MEEEARPAAGAGEAATPARETPPAAPAQARAASGGVPESAPEPKRRQLGTLLQPTVNKFSLRVFGSHKAVEIEQERVKSAGAWIIHPYSDFRFYWDLIMLLLMVGNLIVLPVGITFFKEENSPPWIVFNVLSDTFFLLDLVLNFRTGIVVEEGAEILLAPRAIRTRYLRTWFLVDLISSIPVDYIFLVVELEPRLDAEVYKTARALRIVRFTKILSLLRLLRLSRLIRYIHQWEEIFHMTYDLASAVVRIFNLIGMMLLLCHWDGCLQFLVPMLQDFPSDCWVSMNRMVNHSWGRQYSHALFKAMSHMLCIGYGQQAPVGMPDVWLTMLSMIVGATCYAMFIGHATALIQSLDSSRRQYQEKYKQVEQYMSFHKLPADTRQRIHEYYEHRYQGKMFDEESILGELSEPLREEIINFTCRGLVAHMPLFAHADPSFVTAVLTKLRFEVFQPGDLVVREGSVGRKMYFIQHGLLSVLARGARDTRLTDGSYFGEICLLTRGRRTASVRADTYCRLYSLSVDHFNAVLEEFPMMRRAFETVAMDRLRRIGKKNSILQRKRSEPSPGSSGGVMEQHLVQHDRDMARGVRGLAPGTGARLSGKPVLWEPLVHAPLQAAAVTSNVAIALTHQRGPLPLSPDSPATLLARSARRSAGSPASPLVPVRAGPLLARGPWASTSRLPAPPARTLHASLSRTGRSQVSLLGPPPGGGARRLGPRGRPLSASQPSLPQRATGDGSPRRKGSGSERLPPSGLLAKPPGTVQPPRSSVPEPVTPRGPQISANM

>HCN4_rat

MDKLPPSMRKRLYSLPQQVGAKAWIMDEEEDGEEEGAGGLQDPSRRSIRLRPLPSPSPSVAAGCSESRGAALGAADSEGPGRSAGKSSTNGDCRRFRGSLASLGSRGGGSGGAGGGSSLGHLHDSAEERRLIAAEGDASPGEDRTPPGLATEPERPGAAAQPAASPPPQQPPQPASASCEQPSADTAIKVEGGAAASDQILPEAEVRLGQSGFMQRQFGAMLQPGVNKFSLRMFGSQKAVEREQERVKSAGFWIIHPYSDFRFYWDLTMLLLMVGNLIIIPVGITFFKDENTTPWIVFNVVSDTFFLIDLVLNFRTGIVVEDNTEIILDPQRIKMKYLKSWFVVDFISSIPVDYIFLIVETRIDSEVYKTARALRIVRFTKILSLLRLLRLSRLIRYIHQWEEIFHMTYDLASAVVRIVNLIGMMLLLCHWDGCLQFLVPMLQDFPHDCWVSINGMVNNSWGKQYSYALFKAMSHMLCIGYGRQAPVGMSDVWLTMLSMIVGATCYAMFIGHATALIQSLDSSRRQYQEKYKQVEQYMSFHKLPPDTRQRIHDYYEHRYQGKMFDEESILGELSEPLREEIINFNCRKLVASMPLFANADPNFVTSMLTKLRFEVFQPGDYIIREGTIGKKMYFIQHGVVSVLTKGNKETKLADGSYFGEICLLTRGRRTASVRADTYCRLYSLSVDNFNEVLEEYPMMRRAFETVALDRLDRIGKKNSILLHKVQHDLNSGVFNYQENEIIQQIVRHDREMAHCAHRVQAAASATPTPTPVIWTPLIQAPLQAAAATTSVAIALTHHPRLPAAIFRPPPGPGLGNLGAGQTPRHPRRLQSLIPSALGSASPASSPSQVDTPSSSSFHIQQLAGFSAPPGLSPLLPSSSSSPPPGACSSPPAPTPSTSTAATTTGFGHFHKALGGSLSSSDSPLLTPLQPGARSPQAAQPPPPLPGARGGLGLLEHFLPPPPSSRSPSSSPGQLGQPPGELSPGLAAGPPSTPETPPRPERPSFMAGASGGASPVAFTPRGGLSPPGHSPGPPRTFPSAPPRASGSHGSLLLPPASSPPPPQVPQRRGTPPLTPGRLTQDLKLISASQPALPQDGAQTLRRASPHSSGESMAAFSLYPRAGGGSGSSGGLGPPGRPYGAIPGQHVTLPRKTSSGSLPPPLSLFGARAASSGGPPLTAAPQREPGARSEPVRSKLPSNL

>HCN2_rat

MDARGGGGRPGDSPGATPAPGPPPPPPPPAPPQPQPPPAPPPNPTTPSHPESADEPGPRSRLCSRDSSCTPGAAKGGANGECGRGEPQCSPEGPARGPKVSFSCRGAASGPAAAEEAGSEEAGPAGEPRGSQASFLQRQFGALLQPGVNKFSLRMFGSQKAVEREQERVKSAGAWIIHPYSDFRFYWDFTMLLFMVGNLIIIPVGITFFKDETTAPWIVFNVVSDTFFLMDLVLNFRTGIVIEDNTEIILDPEKIKKKYLRTWFVVDFVSSIPVDYIFLIVEKGIDSEVYKTARALRIVRFTKILSLLRLLRLSRLIRYIHQWEEIFHMTYDLASAVMRICNLISMMLLLCHWDGCLQFLVPMLQDFPSDCWVSINNMVNHSWSELYSFALFKAMSHMLCIGYGRQAPESMTDIWLTMLSMIVGATCYAMFIGHATALIQSLDSSRRQYQEKYKQVEQYMSFHKLPADFRQKIHDYYEHRYQGKMFDEDSILGELNGPLREEIVNFNCRKLVASMPLFANADPNFVTAMLTKLKFEVFQPGDYIIREGTIGKKMYFIQHGVVSVLTKGNKEMKLSDGSYFGEICLLTRGRRTASVRADTYCRLYSLSVDNFNEVLEEYPMMRRAFETVAIDRLDRIGKKNSILLHKVQHDLSSGVFNNQENAIIQEIVKYDREMVQQAELGQRVGLFPPPPPPQVTSAIATLQQAVAMSFCPQVARPLVGPLALGSPRLVRRAPPGPLPPAASPGPPAASPPAAPSSPRAPRTSPYGVPGSPATRVGPALPARRLSRASRPLSASQPSLPHGAPAPSPAASARPASSSTPRLGPAPTTRTAAPSPDRRDSASPGAASGLDPLDSARSRLSSNL

>HCN1_rat

MEGGGKPNSASNSRDDGNSVYPSKAPATGPAAADKRLGTPPGGGAAGKEHGNSVCFKVDGGGGEEPAGSFEDAEGPRRQYGFMQRQFTSMLQPGVNKFSLRMFGSQKAVEKEQERVKTAGFWIIHPYSDFRFYWDLIMLIMMVGNLVIIPVGITFFTEQTTTPWIIFNVASDTVFLLDLIMNFRTGTVNEDSSEIILDPKVIKMNYLKSWFVVDFISSIPVDYIFLIVEKGMDSEVYKTARALRIVRFTKILSLLRLLRLSRLIRYIHQWEEIFHMTYDLASAVVRIFNLIGMMLLLCHWDGCLQFLVPLLQDFPPDCWVSLNEMVNDSWGKQYSYALFKAMSHMLCIGYGAQAPVSMSDLWITMLSMIVGATCYAMFVGHATALIQSLDSSRRQYQEKYKQVEQYMSFHKLPADMRQKIHDYYEHRYQGKIFDEENILSELNDPLREEIVNFNCRKLVATMPLFANADPNFVTAMLSKLRFEVFQPGDYIIREGAVGKKMYFIQHGVAGVITKSSKEMKLTDGSYFGEICLLTKGRRTASVRADTYCRLYSLSVDNFNEVLEEYPMMRRAFETVAIDRLDRIGKKNSILLQKFQKDLNTGVFNNQENEILKQIVKHDREMVQAIPPINYPQMTALNCTSSTTTPTSRMRTQSPPVYTATSLSHSNLHSPSPSTQTPQPSAILSPCSYTTAVCSPPIQSPLATRTFHYASPTASQLSLMQQPQPQLQQSQVQQTQTQTQQQQQQQQPQPQPQQPQQQQQQQQQQQQQQQQQQQQQPQTPGSSTPKNEVHKSTQALHNTHLTREVRPLSASQPSLPHEVSTMISRPHPTVGESLASIPQPVATVHSTGLQAGSRSTVPQRVTLFRQMSSGAIPPNRGVPPAPPPPAAVQRESPSVLNKDPDAEKPRFASNL

>HCN3_rat

MEEEARPAVGDGEAATPARETPPAAPAQARAASGGVPESAPEPKRRQLGTLLQPTVNKFSLRVFGSHKAVEIEQERVKSAGAWIIHPYSDFRFYWDLIMLLLMVGNLIVLPVGITFFKEENSPPWIVFNVLSDTFFLLDLVLNFRTGIVVEEGAEILLAPRAIRTRYLRTWFLVDLISSIPVDYIFLVVELEPRLDAEVYKTARALRIVRFTKILSLLRLLRLSRLIRYMHQWEEIFHMTYDLASAVVRIFNLIGMMLLLCHWDGCLQFLVPMLQDFPSDCWVSMNRMVNHSWGRQYSHALFKAMSHMLCIGYGQQAPVGMPDVWLTMLSMIVGATCYAMFIGHATALIQSLDSSRRQYQEKYKQVEQYMSFHKLPADTRQRIHEYYEHRYQGKMFDEESILGELSEPLREEIINFTCRGLVAHMPLFAHADPSFVTAVLTKLRFEVFQPGDLVVREGSVGRKMYFIQHGLLSVLARGARDTRLTDGSYFGEICLLTRGRRTASVRADTYCRLYSLSVDHFNAVLEEFPMMRRAFETVAMDRLRRIGKKNSILQRKRSEPSPGSSSGGVMEQHLVQHDRDMARGIRGLAPGTGARLSGKPVLWEPLVHAPLQAAAVTSNVAIALTHQRGPLPLSPDSPATLLARSARRSAGSPASPLVPVRAGPLLARGPWASTSRLPAPPARTLHASLSRTGRSQVSLLGPPPGGGGRRLGPRGRPLSASQPSLPQRATGDGSPRRKGSGSERLPPSGLLAKPPGTVQPSRSSVPEPVTPRGPQISANM

>HCN3_cow

MDVSLMDQSDGRPRGIQEEQPAPTSVAVLYGTAPFYSFLKEISSKPPEQGSPTVALVVNAGLPRNLATCQCRLDRCNLPVLSASIDERWLYGQDFVFILVFILSVHSVTGGVALASGPVPGSRPGPEPKRRQLGTLLQPTVNKFSLRVFGSHKAVEIEQERVKSAGAWIIHPYSDFRFYWDLIMLLLMVGNLIVLPVGITFFKEENSPPWIVFNVLSDTFFLMDLVLNFRTGIVVEEGAEILLAPRAIRTRYLRTWFLVDLISSIPVDYIFLVVELEPRLDAEVYKTARALRIVRFTKILSLLRLLRLSRLIRYIHQWEEIFHMTYDLASAVVRIFNLIGMMLLLCHWDGCLQFLVPMLQDFPPDCWVSISHMVNHSWGRQYSHALFKAMSHMLCIGYGQQAPVGMPDVWLTMLSMIVGATCYAMFIGHATALIQSLDSSRRQYQEKYKQVEQYMSFHKLPADTRQRIHEYYEHRYQGKMFDEESILGELSEPLREEIINFTCRGLVAHMPLFAHADPSFVTAVLTKLRFEVFQPGDLVVREGSVGRKMYFIQHGLLSVLARGARDTRLTDGSYFGEICLLTRGRRTASVRADTYCRLYSLSVDHFNAVLEEFPMMRRAFETVAMDRLRRIGKKNSILQRKRSEPSPSSSGGVMEQHLVQHDRDMARGVRGLAPSTGAQLSGKPVLWEPLVHAPLQAAAVTSNVAIALTHQRGPLPLSPDSPVTLLARSARRSAGAGSPASPLVPVRAGPLARGPWASTSRLPAPPARTLHASLSRAGRSQVSLLGPPPGGGGRRLGPRGRPLSASQPSLPQRAAGDGSPGRKGSGSERLPPSGLLTKPPGTAQLPRLPVPEPATPRGPQLSANM

>HCN4_chimpanzee

MDKLPPSMRKRLYSLPQQVGAKAWIMDEEEDAEEEGAGGRQDPSRRSIRLRPLPSPSPSAAAGGTESRSSALGAADSEGPARGAGKSSTNGDCRRFRGSLASLGSRGGGSGGTGSGSSHGHLHDSAEERRLIAEGDASPGEDRTPPGLAAEPERPGASAQPAASPPPPQQPAQPASASCEQPSVDTAIKVEGGAAAGDQILPEAEVRLGQAGFMQRQFGAMLQPGVNKFSLRMFGSQKAVEREQERVKSAGFWIIHPYSDFRFYWDLTMLLLMVGNLIIIPVGITFFKDENTTPWIVFNVVSDTFFLIDLVLNFRTGIVVEDNTEIILDPQRIKMKYLKSWFMVDFISSIPVDYIFLIVETRIDSEVYKTARALRIVRFTKILSLLRLLRLSRLIRYIHQWEEIFHMTYDLASAVVRIVNLIGMMLLLCHWDGCLQFLVPMLQDFPDDCWVSINNMVNNSWGKQYSYALFKAMSHMLCIGYGRQAPVGMSDVWLTMLSMIVGATCYAMFIGHATALIQSLDSSRRQYQEKYQVEQYMSFEIINFNCRKLVASMPLFANADPNFVTSMLTKLRFEVFQPGDYIIREGTIGKKMYFIQHGVVSVLTKGNKETKLADGSYFGEICLLTRGRRTASVRADTYCRLYSLSVDNFNEVLEEYPMMRRAFETVALDRLDRIGKKNSILLHKVQHDLNSGVFNYQENEIIQQIVQHDREMAHCAHRVQAAASATPTPTPVIWTPLIQAPLQAAAATTSVAIALTHHPRLPAAIFRPPPGSGLGNLGAGQTPRHLKRLQSLIPSALGSASPASSPSQVDTPSSSSFHIQQLAGFSAPAGLSPLLPSSSSSPPPGACGSPSAPTPSAGAAATTIAGFGHFHKALGGSLSSSDSPLLTPLQPGARSPQAAQPPPAPPGARGGLGLPEHFLPPPPSSRSPSSSPGQLGQPPGELSLGLATGPLSTPETPPRQPEPPSLVAGASGGASPVGFTPRGGLSPPGHSPGPPRTFPSAPPRASGSHGSLLLPPASSPPPPQVPQRRGTPPLTPGRLTQDLKLISASQPALPQDGAQTLRRASPHSSGESMAAFQLFPRAGGGSGGSGSSGGLGPPGRPYGAIPGQHVTLPRKTSSGSLPPPLSLFGARATSSGGTPLTAGPQREPGARPEPVRSKLPSNL

>HCN1_chimpanzee

MEGGGKPNSSSNSRDDGNSVFPAKASATGAGPAAAEKRLGTPPGGGGAGAKEHGNSVCFKVDGGGGGGEEPAGGFEDAEGPRRQYGFMQRQFTSMLQPGVNKFSLRMFGSQKAVEKEQERVKTAGFWIIHPYSDFRFYWDLIMLIMMVGNLVIIPVGITFFTEQTTTPWIIFNVASDTVFLLDLIMNFRTGTVNEDSSEIILDPKVIKMNYLKSWFVVDFISSIPVDYIFLIVEKGMDSEVYKTARALRIVRFTKILSLLRLLRLSRLIRYIHQWEEIFHMTYDLASAVVRIFNLIGMMLLLCHWDGCLQFLVPLLQDFPPDCWVSLNEMVNDSWGKQYSYALFEAMSHMLCIGYGAQAPVSMSDLWITMLSMIVGATCYAMFVGHATALIQSLDSSRRQYQEKYKQVEQYMSFHKLPADMRQKIHDYYEHRYQGKIFDEENILNELNDPLREEIVNFNCRKLVATMPLFANADPNFVTAMLSKLRFEVFQPGDYIIREGAVGKKMYFIQHGVAGVITKSSKEMKLTDGSYFGEICLLTKGRRTASVRADTYCRLYSLSVDNFNEVLEEYPMMRRAFETVAIDRLDRIGKKNSILLQKFQKDLNTGVFNNQENEILKQIVKHDREMVQAIAPINYPQMTTLNSTSSTTTPTSRMRTQSPPVYTATSLSHSNLHSPSPSTQTPQPSAILSPCSYTTAVCSPPVQSPLAARTFHYASPTASQLSLMQQQPQQQVQQSQPPQTQPQQPSPQPQTPGSSPDAEKPRFASNL

>HCN1_dog

MEAGGKPNSASNSRDDGSSAFPGKAPATGAGPAAAEKRLGTPPGGGGAGAKELGNSVCFKVDGGGAGEEPAGGFEDAEGPRRQYGFMQRQFTSMLQPGVNKFSLRMFGSQKAVEKEQERVKTAGFWIIHPYSDFRFYWDLIMLIMMVGNLVIIPVGITFFTEQTTTPWIIFNVASDTVFLLDLIMNFRTGTVNEDSSEIILDPKVIKMNYLKSWFVVDFISSIPVDYIFLIVEKGMDSEVYKTARALRIVRFTKILSLLRLLRLSRLIRYIHQWEEIFHMTYDLASAVVRIFNLIGMMLLLCHWDGCLQFLVPLLQDFPPDCWVSLNEMVNDSWGKQYSYALFKAMSHMLCIGYGAQAPVSMSDLWITMLSMIVGATCYAMFVGHATALIQSLDSSRRQYQEKYKQVEQYMSFHKLPADMRQKIHDYYEHRYQGKIFDEENILNELNDPLREEIVNFNCRKLVATMPLFANADPNFVTAMLSKLRFEVFQPGDYIIREGAVGKKMYFIQHGVAGVITKSSKEMKLTDGSYFGEICLLTKGRRTASVRADTYCRLYSLSVDNFNEVLEEYPMMRRAFETVAIDRLDRIGKKNSILLQKFQKDLNTGVFNNQENEILKQIVKHDREMVQAIAPINYPQMTALNSTSSTATPTCRARTQSPPVYSAPSLSHGNLHSPSPGTQTPQPSAVLSPCSYTTAVCSPPVQSPLAARTFHYASPTASQLSLAQQPQPAQPPQPPQPAQPAQPPQPQPPQPQPQPPQPPQPPQPPQTPGGGSTPRNDVHRSTQALHNASLTREVRPLSASQPSLPHEVATLMSRPHPTVGESLASIPQPVTAVHGPGLQAGGRGTVPQRVTLFRQMSSGAIPPNRGAPPAPPPPAAALPRESSSVLTTDPEAEKPRFASNL

>HCN3_dog

MDAEQRQTTGADERATPGLEAAPPAAPAPATAASGPPPGPGSGPEPKRRQLGTLLQPTVNKFSLRVFGSHKAVEIEQERVKSAGAWIIHPYSDFRFYWDLIMLLLMVGNLIVLPVGITFFKEENSPPWIVFNVLSDTFFLLDLVLNFRTGIVVEEGAEILLAPRAIRSRYLRTWFLVDLISSIPVDYIFLVVELEPRLDAEVYKTARALRIVRFTKILSLLRLLRLSRLIRYIHQWEEIFHMTYDLASAVVRIFNLIGMMLLLCHWDGCLQFLVPMLQDFPPDCWVSINHMVNHSWGRQYSHALFKAMSHMLCIGYGQQAPVGMPDVWLTMLSMIVGATCYAMFIGHATALIQSLDSSRRQYQEKYKQVEQYMSFHKLPADTRQRIHEYYEHRYQGKMFDEESILGELSEPLREEIINFTCRGLVAHMPLFAHADPSFVTAVLTKLRFEVFQPGDLVVREGSVGRKMYFIQHGLLSVLARGARDTRLTDGSYFGEICLLTRGRRTASVRADTYCRLYSLSVDHFNAVLEEFPMMRRAFETVAMDRLRRIGKKNSVLQRKRSEPSPGSSGGTMEQHLVQHDRDMARGVRGLAPDTGARLSGKPVLWEPLVHAPLQAAAVTSSVAIALTHQRGPLPLSPDSPVTLLARSARRSAGSPASPLVPVRAGPLLARGPWASTSRLPVPPARTLHASLSRAGRSQVSLLGPPPGGGGRRLGLRGRPLSASQPSLPQRAAGDGSPGCKGLGSERLSSSGLLAKLPGTAQPPRPSVPEPAAPRGPQLSANM

>HCN4_dog

MDEEEDAEEDGAGGRRDPSRRSIRLRPLPSPSPSPAPSPAPSAADGAGKASSNGDCRRFRGSLASLGSRGGGTGGIFQEIEASLNHADFMQRQFGAMLQAGVNKLSQRMFGSQKAAELEQERVKSAGFWIIHPYSDFRFYWDLTMLLLMVGNLIIIPVGITFFKDENTTPWIVFNVVSDTFFLIDLVLNFRTGIVVEDNTEIILDPQRIKMKYLKSWFVVDFISSIPVDYIFLIVETRIDSEVYKTARALRIVRFTKILSLLRLLRLSRLIRYIHQWEEIFHMTYDLASAVVRIVNLIGMMLLLCHWDGCLQFLVPMLQDFPDDCWVSINNMVNNSWGKQYSYALFKAMSHMLCIGYGRQAPVGMSDVWLTMLSMIVGATCYAMFIGHATALIQSLDSSRRQYQEKYKQVEQYMSFHKLPPDTRQRIHDYYEHRYQGKMFDEESILGELSEPLREEIINFNCRKLVASMPLFANADPNFVTSMLTKLRFEVFQPGDYIIREGTIGKKMYFIQHGVVSVLTKGNKETKLADGSYFGEICLLTRGRRTASVRADTYCRLYSLSVDNFNEVLEEYPMMRRAFETVALDRLDRIGKKNSILLHKVQHDLNSGVFNYQENEIIQQIVQHDREMAHCAHRVQAAAAAAAAAPPAPVIWTPLIQAPLQAAAATTSVALALTHHPRLPAAVF

>HCN4_opposum

MDKLPPSMRKRLYSLPQQVGPKAWIMDEEEDEEESDKDTRRKSIRLRPLPSPSAAAGGGEPRSAAPGIMETEVGSRSGGGGSTAKSSTNGDCRRFRGSLSSLTSRGGGGGGGGSGGGGGGGTSSSGGGGGSSHLRDSAEEKRLIGEGDASPCEDKSPPGLDGEPEPPESLGQSPMPSCSAEQPSGSSATCIKVEGGAGGDQIVPDDEARLGQAGFMQRQFGAMLQPGVNKFSLRMFGSQKAVEREQERVKSAGFWIIHPYSDFRFYWDLTMLLLMVGNLIIIPVGITFFKDENTTPWIVFNVVSDTFFLIDLVLNFRTGIVVEDNTEIILDPQRIKMKYLKSWFVVDFISSIPVDYIFLIVETRIDSEFYKTARALRIVRFTKILSLLRLLRLSRLIRYIHQWEEIFHMTYDLASAVVRIVNLIGMMLLLCHWDGCLQFLVPMLQDFPGDCWVSLNRMVNDSWGKQYSYALFKAMSHMLCIGYGKQAPVGMSDVWLTMLSMIVGATCYAMFIGHATALIQSLDSSRRQYQEKYKQVEQYMSFHKLPPDTRQRIHDYYEHRYQGKMFDEESILGELSEPLREEIINFNCRKLVASMPLFANADPNFVTSMLTKLRFEVFQPGDYIIREGTIGKKMYFIQHGVVSVLTKGNKETKLADGSYFGEICLLTRGRRTASVRADTYCRLYSLSVDNFNEVLEEYPMMRRAFETVALDRLDRIGKKNSILLHKVQHDLNSGVFNYQENEIIQQIVQHDREMAHCAHHVQSAAVSTPTPVIWTPLIQAPLQAAAATTSVAIALTHHPRLPAAIFRPPTGAGSGLGSLGAGQTPRHLKRLHSLIPSALGSPASTPSQLDTPSSSSFHIQQLAGFSAPAGLGPFSSASSPPAGPGPPPPGACGSPLETASAAPTTPAATGFGHFHKASGGSLSSSDSPLLTPLPSRTRSPPGTQVQPLAGAAGGLGLLEHFLPPPGSSGSPSSSPSQLVQPPGASSPVSVPPSALGMSPMQPERLPFAMGASGGVSSAAFTPRGTQDLKLISASQPALPQDGAQTLLRGSPHSSGESVAAFPPFRGGGGGFGPLGRPFGTGPTQRVTLPRQTSSGSLPPPPPLFGAEAASPGGPPLTAAPRGRESTGLVGAPEPPVRSKLPSNL

>HCN3_opposum

MDTKQRPAAAAAGAGDEGTPGLETASPPAPSPATAAPGPSPAPGPESGPGPMRRQLGALLQPAVNKFSLRVFGSHKAVEIEQERVKSAGAWIIHPYSDFRFYWDLIMLLLMVGNLIVLPVGITFFKEENSPPWIVFNVLSDTFFLLDLVLNFRTGIVVEEGAEILLAPQAIRTRYLRTWFLVDLISSIPVDYIFLVVELEPRLDAEVYKTARALRIVRFTKILSLLRLLRLSRLIRYIHQWEEIFHMTYDLASAVVRIFNLIGMMLLLCHWDGCLQFLVPMLQDFPPDCWVSINHMVNHSWGRQYSHALFKAMSHMLCIGYGQQAPQGMPDVWLTMLSMIVGATCYAMFIGHATALIQSLDSSRRQYQEKYKQVEQYMSFHKLPADTRQRIHEYYEHRYQGKMFDEESILGELSEPLREEIINFTCRGLVAHMPLFAHADPSFVTAVLTKLRFEVFQPGDLVVREGSVGRKMYFIQHGLLSVLTRGARDTRLTDGSYFGEICLLTRGRRTASVRADTYCRLYSLSVDHFNAVLEEFPMMRRAFETVAMDRLHRIGKKNSLLQRKRSEPSPGSGGSGVMEQHLVQHDRDMARGVRGLAPGTGARLSGKPVLWEPLVHAPLQAAAVTSNVAIALTHHRAPLPLSPDSPAALLARSARRSAGSPASPLVPARGLPLLARGPWASTSRLPAPAARTLHASLSRAGRSQVSLLGPPPGGGGRRLGPRGRPLSASQPSLPHRAAGEGSPGRKGSGGERLPASGLLTRLPGTAQPLRPPASDPGTSRGPQLPANM

>HCN1_opposum

MDGAGKPNSSSGSRDDGNSVFPVKPPSSVGGAAGAVAEKNLGSPPGGGGGGSTKEHGNSVCFKVDGGSGGSTGAGGGEEPVVGFEDAEGPRRQYGFMQRQFTSMLQPGVNKFSLRMFGSQKAVEKEQERVKTAGFWIIHPYSDFRFYWDLIMLIMMVGNLVIIPVGITFFTEQTTTPWIIFNVASDTVFLLDLIMNFRTGTVNEDSSEIILDPKIIKMNYLKSWFVVDFISSIPVDYIFLIVEKGMDSEVYKTARALRIVRFTKILSLLRLLRLSRLIRYIHQWEEIFHMTYDLASAVVRIFNLIGMMLLLCHWDGCLQFLVPLLQDFPSDCWVSLNKMVNDSWGKQYSYALFKAMSHMLCIGYGAQAPVSMSDLWITMLSMIVGATCYAMFVGHATALIQSLDSSRRQYQEKYKQVEQYMSFHKLPADMRQKIHDYYEHRYQGKIFDEENILSELNDPLREEIVNFNCRKLVATMPLFANADPNFVTAMLSKLRFEVFQPGDYIIREGAVGKKMYFIQHGVAGVITKSSKEMKLTDGSYFGEICLLTKGRRTASVRADTYCRLYSLSVDNFNEVLEEYPMMRRAFETVAIDRLDRIGKKNSILLQKFQKDLNTGVFNNQENEILKQIVKHDREMVQAIAPMNFPQMQALNSGTSTTTPASRMRTQSPPVYTATSLSHSNLHSPSPSTQTPQQAVILSPCSYTTAVCSPPVQSPLAARTFQYASPTASQLSLMQQHPPPAQPPQQPQPQQPQQPPQPLQQQQQPQQQQQQQQQPQQPQPQPQQQQQPQQQPQQQQQPQTPTISSQKNEVHRSTQALHNTNLTREVRPLSASQPSLPHEISTLISRPHPTVGESLASIPQPVSAVHGPGIQAGARGSVSQRITLFRQMSSGAIPPNRGAVPAPPPTAATPQRDSSTVLSTDQEGDKPRFASNL

>HCN4_chicken

MDKLPPSMRKRLYSLPQQIGPKASIMDEEEDSDKDTRRKSIRLKPLPSPSAGSTRALGGDPGRGEDPGLVETEASSKGAKTSTNGDCRRFKGSLSSLTSRHLHDAAEEKRLIGGEGEPASPGEDKSPSGSGETQGLPVRPPPASPPEPQQQPLRACSSTSIKVEGGGGCDQITPDEEQRLGQAGFMQRQFGAMLQPGVNKFSLRMFGSQKAVEREQERVKSAGFWIIHPYSDFRFYWDLTMLLLMVGNLIIIPVGITFFKDENTTPWIVFNVVSDTFFLIDLVLNFRTGIVVEDNTEIILDPQRIKMKYLKSWFVVDFISSIPVDYIFLIVETRIDSEVYKTARALRIVRFTKILSLLRLLRLSRLIRYIHQWEEIFHMTYDLASAVVRIVNLIGMMLLLCHWDGCLQFLVPMLQDFPDDCWVSLNRMVYKQVEQYMSFHKLPADMRQRIHDYYEHRYQGKMFDEESILGELSEPLREEIINFNCRKLVASMPLFANADPNFVTSMLTKLRFEVFQPGDYIIREGTIGKKMYFIQHGVVSVLTKGNKETKLADGSYFGEICLLTRGRRTASVRADTYCRLYSLSVDNFNEVLEEYPMMRRAFETVALDRLDRIGKKNSILLHKVQHDLNSGVFNYQENEIIQQIVQHDREMAHCAHNVQAAAAAAAAAASTPTPVIWTPLIQAPLQAAAATTSVAIALTHHPRLPTAIFRPPVSVLGSLGQQSSQTPRQLKRLQSLIPSTGPSAVGSPSSTPSQLHTPGAETPSSSSYHIQQLAGYSAAAGLGQFQVGSPPAGSGQQGLSGASSAGLSQLQQAPSGSPLGTAQPLQQQQLQRQPSLSSSGFGHFQQAAASSPSTSLTQLSSNSPPSLLNQFQPATRPLQGGQLQQLSGSGTLGGINHFQPPPSSNSPSSSLSQLAQASGGPSSGLCQTQPSALGSLTGTIAQLHQERSPFASVSPLQQSGVASPCYTPSGLSPPTQSPVATRTFQCGPLGASGSHGSLLLPQTASPPLQILQSKSTPPVPPGRLNQDIKLISASQPSLPQELAQTLSQSSHSSRESVSSFSPFPGGGTGLLGKPCXSIPGRVTLPHQMSSGSLPHPL

>HCN2_chicken

MGAVGAPPGLRTRGLRQRGELRSRCGPVARPRPGPPARPRPAPRRGGGYRSGTARVPLGRRWPRRQRTMRSGGEAAAEEEAAEGAKRGGGAAAGRARGRGGKGSPNGECRRGETPRSPSAEQPREPKVSFSCGGGGGGGGGGSASPGGAKAAEEGDDAGEEVRGSQASFMQRQFGAMLQPGVNKFSLRMFGSQKAVEREQERVKSAGAWIIHPYSDFRFYWDFTMLLFMVGNLIIIPVGITFFKEETTAPWIVFNVVSDTFFLMDLVLNFRTGIVIEDNTEIILDPERIKKKYLKTWFVVDFVSSIPVDYVFLIVEKGIDSEVYKTARALRIVRFTKILSLLRLLRLSRLIRYIHQWEENDSWSELYSFALFKSMSHMLCIGYGKQAPESMTDIWLTMLSMIVGATCYAMFIGHATALIQSLDSSRRHYQEKIVNFNCRKLVASMPLFANADPNFVTAMLTKLKFEVFQPGDYIIREGTIGKKMYFIQHGVVSILTKGNKEMKLSDGSYFGEICLLTRGRRTASVRADTYCRLYSLSVDNFNEVLEEYPMMRRAFETVAIDRLDRIGKKNSILLHKVQHDLNSGVFNNQENEIIQEIVKYDREMVQQAELQQHTAMYSPVQPQVTSAIATLQQAVAMSFCPQMASPLVGSMALGSPRMMRRLQYAQAVPSPFAVSPVLLQQSPPQQPQPPVPHANPSPSQDQAQPASASAFAAASPPSQSPLASRTFAYGGAKGQLGSQLSLSQQQTPGSPPRLAVHKSTQALPTSSFSQDSRPLSASQPSLPHGLAAGSTQSPPASVHESSASIGGGPAATAVSPGSGPPAGLRGAVSSRGPLSHPAPTLQQDSAAARKDSVGSMPDTDPAKSRLSSNL

>HCN2_frog

MDGRGGGGCSAAGMRGVEVAAAAAEEQLPRGVGRRGTRSPCSSAGTPGTGKGSPNGECRRELSETPPREHKVSFSTEPPRGSSPGKEPEDGDGGEDETRSGFMQRQLSAMLQPGVNKFSLRMFGSQKAVEREQERVKSAGAWIIHPYSDFRFYWDFTMLLFMVGNLIIIPVGITFFKDETTTPWIVFNVVSDTFFLMDLVLNFRTGIVIEDNTEIILDPQKIKRKYLRTWFVVDFVSSIPVDYIFLIVEKGIDSEVYKTARALRIVRFTKILSLLRLLRLSRLIRYIHQWEEIFHMTYDLASAVMRIFNLIAMMLLLCHWDGCLQFLVPMLQDFPTNCWVSINKMVNDSWSELYSFALFKAMSHMLCIGYGRQAPESMSDIWLTMLSMIVGATCYAMFIGHATALIQSLDSSRRQYQEKYKQVEQYMSFHKLPADFRQRLHDYYEHRYQGKMFDEDSILGELNEPLREEIVNFNCRKLVASMPLFANADPSFVTAMLTKLKFEVFQPTDYIIREGTIGKKMYFIQHGVVSVLTKGNKEVKLSDGSYFGEICLLTRGRRTASVRADTYCRLYSLSVDHFNEVLEEYPMMRRAFETVAIDRLDRIGKKNSILMHKVQHDLNSGVFNNKENEIIQEIVKYDREMVHQAELNQQLAGGMYATCNPPSPQPQVTSAIATLQQAVAMSFCPPMASPLVGSPRIMRRFQYAQGGPSAFSLSPALFQPPLQQMQHYPPPQVSRQPSSVVPSPPSQSPRVSQNFAYPTKGQTGSQLSLTQSGTSASPSPHRLSVHKSTHSLHSGTLTRESRPLSASQPSLPQGASQLRSPPAPPLASGGLSSQLGKGMPGSQAGPRVPVPARLALSHHLSSSSSVGGVSDSAGGASLGGRKDSVVSAPDTDPIRGRLSANL

>HCN1_frog

MESKFNSSTNSSRDDGNNVLQGKAEKSLTASTSSVKEHGNSVCFKTDAADDPVVGFEDAEGPSRPHGFMQRQFGSLMQPGVNKFSLRMFGSQKAVEKEQERVKTAGVWIIHPYSDFRFYWDLIMLIMMVGNLVIIPVGITFFTEQTTTPWIIFNVASDTVFLFDLFMNFRTGIVIEDNSEIILDPKVIKMNYLKSWFVVDFISSIPVDYIFLIVEKGMDSEVYKTARALRIVRFTKILSLLRLLRLSRLIRYIHQWEEIFHMTYDLASAVVRIFNLIGMMLLLCHWDGCLQFLVPLLQENDSWGKQYSYALFKAMSHMLCIGYGARAPVSMSDLWITMLSMIVGATCYAMFVGHATALIQSLDSSRRQYQEKYKQVEQYMSFHKLPSDMRQKIHDYYEHRYQGKIFDEDNILNELNDPLREEIVNFNCRKLVATMPLFANADPNFVTAMLSKLKFEVFQPGDYIIREGAVGKKMYFIQHGVAGVITKSSKEMKLTDGSYFGEICLLTKGRRTASVRAETYCRLYSLSVENFNEVLEEYPMMRRAFETVAIDRLDRIGKKNSILQQRFQKDLNTGIFNNQESEILKQMLTQDREMVEGHPSVSYQQLPALNSSTSSSLRMRTRSPPVYNANSLSHGNLQSPTPSPQTPQQAVVFSPSSFTSAACSPSVQSPLAGRTFQYSSLTASQLSLIQQQQQASGASQIHEVHKSAQALANTNLTREVRPLSASQPSLPHEISTLGSKPHPTVGESLASLPQPISNVQAAGTQSGSRSNIPPRVALFRQMSSGALPPARVGATTQPSPAPPTKDSSTVSSTDTDTDKLKYASNL

>HCN4_frog

MDRLHPSMRKRLYSLPQHIGFKASIMDEAECEERDTWRKSIKLKPLPSPSALRVLDPRHGAKETHIVMETEVGKAQRTSSNGDCRRFTGSLSSITSCGRHLHDADPSEGRRLISDGDISPTEECSPTLDPAVGESNIHLSQNTQTSSQCQGAEQLSSPGKLDGREQTLSGGEDEQGSFMHRQFGALLQPGVNKFSLRMFGSQKAVEREQDRVKSAGFWIIHPYSDFRFYWDLTMLLLMVGNLIIIPVGITFFKEENTPAWIVFNVVSDTFFLVDLVLNFRTGIVLEDNTEIILEPQRIKIRYLKSWFLVDFVSSIPVDYIFLIVETRIDSEVYKTARALRIVRFAKVLSLLRLLRLSRLIRYIHQWEENASWGKQYSYALFKAMSHMLCIGYGQQAPVGMGDVWLTMLSMIVGATCYAMFIGHATALIQSLDSSRRQYQEKYKQVEQYMSFHKLPADLRQRIHDYYEHRYQGKMFDEESILGELSEPLREEIINFNCRKLVASMPLFANADPNFVTSMLTKLRFEVFQPGDYLIREGTVGKKMFFIQHGVVSILSKGSKETKLSDGSYFGEICLLTRGRRTASVRADTYCRLYSLSVDHFNEVLEEYPMMRRAFESVALDRLDRIGKKNSLLLHKVQHDLRSGVLNYHENEIIQQIVQHDREMAQGPRCSQTEEESTSLPATPVIWTPLVQAPLRAAAATTSVAIPLTHQRIPATIFRPAPSALGVTARGLRQAQRTALPTHLIGIEGAVSQPSSFHVHQLAGFSAAACVSPAPPPSVPPLQRHIVKHLSTYSPPAEPLQLVTGEANSSISLSPELSQSDQQFRPLSSGQCQYPVSHSLDTHLPGASEPQWVLPPPPHKADSPGKATAVLSPGPVRTAPIGGSSRSLLLPEITALSNLPNSCTALPLNRLRQDGRPISVSQPSLPQAPASGSPNFPQQFIPDFSSLPRSEKSNIGHVTLPRKGPSVLGLGVQSICGEDLSHTKLPSNL

>HCN1_green_puffer

MEDKTGSFSSGKEEKADGNNVFQRQDSIQKNNTGSQNTKEHSNSVGFKGEREEAMVGFDDLEGASRQHGFMQRQFGAMMQPGVNKFSLRMFGSQKAVEKEQERVQTAGYWIIHPYSDFRFYWDLIMLIMMMGNLIIIPVGITFFSDQNTTTWLVFNVASDTIFLVDLVMNFRTGIVNEESSEIILDPKVIKMNYLKSWFVVDFLSSIPVDYIFLIVEKGFDSEVYKTARALRIVRFTKILSLLRLLRLSRLIRYIHQWEEIFHMTYDLASAVVRIFNLIGMMLLLCHWDGCLQYLVPLLQDFPPDCWVSLNGMVNVSWGKQYSYALFKAMSHMLCIGYGARAPVSMSDLWITMLSMIVGATCYAMFVGHATALIQSLDSSRRQYQEKYKQVEQYMSFHKLPADMRQKIHDYYEHRYQGKIFDEDNILSELNDPLKEEIVNFNCRKLVATMPLFANADPNFVTGMLSKLKFEVFQPNDYIIREGTVGKKMYFIQHGVASVITKSNKEMKLTDGSYFGEICLLTKGRRTASVRADTYCRLFSLSVDHFNEVLEEYPMMRRAFETVAIDRLDRIGKKNSLLLQKFQKDLNAGVFNTQENEMLKQIIRQDREMVMMVDRKQSVTGMNSTPASGNSIINSPAQPLFPAAFGFSQLQQASAPMTYSASAIANASAARMLPVAAAAAAAAQAGYPPPSLSHSSLNSSLTNPQGALQQAAIMSPCSFTAAMCSPPVQTPLANRSFQYGSRTASQLSLIQQPTGQPTLPTQQPPASATASAPQQLQQQLQQQQVSPQQQQVPSPQRGDIPKGAQALQSGSLSRDVRHLSASQPSLPHEASLGPRAHPASGDSLASIAPPTAAAIQGMSLQGGIRTTVPQRVNLFRQMSSGALPPVRSAAAAAAAAAASSVSSSSTQQREAAGSRRDSVLSSTETEQDKLRFSSNL

>HCN4b_green_puffer

MDRLHSSMRKRLYSLPQHIGHKASIMSEGEDADKDARRKSIRMKPLPSPSPTSAGSCKGIGETKSGESGIMETDIGRPMKTSSNGDCRRFRGSLSSITSRHVHDSNSAEERRLITEGDVTPSEESPPGAIGDGPPEQGAQGASGGGGGGGGGGQKEPPDQQPGFVKLDGIDQILPDDERLYQAGFMHRQFGAMLQPGVNKFSLRMFGSEKAVEREQERVKSAGFWIIHPYSDFRFYWDLTMLLLMVGNLIIIPVGITFFKDEHTPPWIVFNVVSDTFFLMDLVLNFRTGIVKEDNTEIILDPQQIKIKYLRSWFVVDFISSIPVDYIFLIVETRIDSDFYKTARALRIVRFTKILSLLRLLRLSRLIRYIHQWEEIFHMTYDLASAMVRIVNLIGMMLLLCHWDGCLQFLVPMLQDFPADCWVSKNKMVNDTWGQQYSYALFKAMSHMLCIGYGMYPPVGMTDVWLTILSMIVGATCYAMFVGHATALIQSLDSSRRQYQEKYKQVEQYMSFHKLPADMRQRIHDYYEHRYQGKMFDEESILGELNEPLREEIINFNCRKLVASMPLFANADPNFVTSMLTKLRFEVFQPGDYIIREGTIGKKMYFIQHGVVSVLTKGNKETKLSDGSYFGEICLLTRGRRTASVRADTYCRLYSLSVDNFNEVLEEYPMMRRAFETVALDRLDRIGKKNSILQHKVQHDLSSGVLNYQENEIIQQIVQHDRDMAHCAHLMQNVPPQTPPSPTPVIWAPLIQAPLQAAAATTSVAIALTHHPHLPHTLFRPPVSVLGSLKDPSSRMKKIHTFALSTAPGSATDSPSSTPSKTLTEVDMPLLTSFQAQDMPSGTLVTGSSKEGSGIEDHHRPNLSDSGLGGGGLSAFPFEQFSSTGSPSSSIAQLHSQPKRQQSFRSGTPPLSNLFHQGSTGGSTGSGLSGGACGTTGWSSGGAVGESVEGATGGDSSWVGEDFTTGTVDVNPGINFGLGMGSVGSIGEISGESIGRISEGLMGAAFGRGSGASSGASSASGGASCGPLADVARSLKGTARGSFGTSSGGSVGSSGETLAVVTTEGSLGRASGKTKSVLSRGLTIGSTGESHGDSMGLNSGGPFGGASVGTLVSHLGGGSSDSGGGMTLGHSSRAAGGSHIGLQQCGLGANSGGGTTGHIISSRCQSPIFTSSSSFMPGQVPHCHPPSKTPQVSSLQQFAGGGRTTGGLNLLHSPPQGSPSTCRRQHSQQPPEGSPSSLGHFSLTGLPSGILPHQMSIDRSALASLAQYGSANASPCLTPVAPSPTIQSPVAGRTFHYSDPPNTTVSNSSLPMAHSSFPSQLPSQSPQTGRDSPLGRFSEDLKLLSSSHPSLPQEVAQALGHHAPFSSVETGYYPSPFSSPTLVPKPCTPVPGRMTPPIQTSPGHRQQTQSQRASPALPLQRGSSVNPTDLETQTVRSKLPSNL

>HCN4a_green_puffer

MERLQSSMRKRLYSLPQNIGPKPVAVDEGDHGDKDTKRKSIRLKHLSSPSPASSCRSIEETRSDDLDRDVPVKSNLNGDCRFFRGSLSSITGRHPPGSDPAEQQRLIPAADVGVSGSFEEGPAAQQRAACGLGGTSAFDQPAFIKLEGAEQILPEDERLYQAGFMHRQFGAMLQPGVNKFSLRMLGSERAVEHERERVKSAGFWIIHPYSDFRFYWDLTMLLLMVGNLIIIPVGITFFKDEHTPPWIVFNVVSDTFFLMDLVLNFRTGIVKEDNTEIILDPQKIKIKYLKSWFMVDFISSIPVDYIFLIVETRINSDFYKTARALRIVRFTKILSLLRLLRLSRLIRYIHQWEEVFHMTYDLASAMVRIMNLIGMMLLLCHWDGCLQFLVPMLQDFPPDCWVTRNKMVNDTWGQQYSYALFKAMSHMLCIGYGLYPPIGMADVWLTILSMIVGATCFAMFVGHATALIQSLDSCRRQYQEKYKQVEQYMSFHKLPADMRQRIHEYYEHRYQGKMFDEESILEELNEPLREEIINFNCRKLVASMPLFANADPNFVTSMLTKLRFEVFQPGDYIIREGTIGKKMYFIQHGVVSVLTKSSKDTKLSDGSYFGEICLLTRGRRTASVRADNYCRLYSLSVDNFNEVLEEYPMMRRAFETVALDRLDRIGKRNSVLQHKVQHHQSSGTLNLQESEIIQRIVQHDRDMAQCTQLIQSSLNPSPVPPSPTPIIWTPLVQAPLQAAAATTPLALALAHQTQLPPILLHHPVVSGSLKDPPGHPKKVYVGVNSCGSGPGSPTCSSKVRAALETPTLASLRTHQLSAGAVSPTPVSQPIFTSSLQPLGLPLLHPPQSLHAGMASVFPMGHTSVSYACATQPSSQPFHGAMTTPPSSVGLLQQGVLGRLVSRVPAPSTISPLISSSVGPVLNHLQRTSQPSMQPWMSSGLNLPHSPLICSTLPSSGCTQPGLPAAAGATGSLAQHSLAGLQSAFLPQVLPEYSALASLAQYGSADASPCYTPPVHSPSLQSPVKGRTGQQTELPCTMDSQSPVITAASWPSGMSCTPTERAETTGDIFTQEIKTISGSHTSIHQDRPLATSGSSQGKSSPSGSFFSSVDISEPCKPLPGQATPFSWTQSGPETQIKAAKFLKAEPRESNQTDL

>HCN2a_green_puffer

MKCNKNGECRRDSGTGSLRSIISKQEKQSGGSGRAEDGGSAKRGACNSTSASMDGSVTPGGSLTGGQADGVNPSTAVSSGVPDKKDSKVSFSNAPSRKASSSLAQTQGQGQGQGQQPRNSVTFQKPEDGPPIVPAEDAETRGSQASFMQRQFSSMLQPGVNKFSLRMYGSQKAVEREQERVKSAGNWIIHPYSDFRFYWDFTMLLFMVGNLIIIPVGITFFKDETTTPWIIFNVVSDTFFLMDLVLNFRTGIIIEDNSDIILDPKTIKKKYLKTWFIVDFISSIPVDYIFLIVEKGIDSEVYKTARALRIVRFTKILSLLRLLRLSRLIRYIHQWEEIFHMTYDLASAVMRIINLIGMMLLLCHWDGCLQFLVPMLQDFPSDCWVSLNKMENDTWSELYSFAVFKAMSHMLCIGYGRQAPESLSDIWLTMLSMIVGATCYAVFIGHATALIQSLDSSRRQYQEKYKQVEQYMSFHKLPADFRQKIHDYYEHRYQGKMFDEESILGELSEPLREEIVNFNCRKLVASMPLFANAEPNFVTAMLTKLRFEVFQPHDYIIREGTIGKKMYFIQHGVCSVITKGTLAMKLSDGSYFGEICLLTRGRRTASVRAETYCRLYSLSVDHFNEVLEEYPMMRRAFETVAIDRLNRIGKKNSILMHKVQHDLNSGVFNNQENEMIQEIVKYDREMVKLVDLQRPRAMSMTPSIGGIFAPPGQPPTGSAIATLQQAVAMSFCPQMASPLVGPGTLQSPRMVRRFQVMQNLSSPVSMSPLQSQPPQAFGGAFGSAISSPPVQSPLAATGRTFQYMASTGPSGSQLSLVQHHAPSPTQTQQRPASHKSTHSLHAGSLSQDTRALSASQPSLPQEGTPPAASAAPSPPASTHTSNNSIGPPQPPHPNLPGPSGAGRPAGAQQRVAFASTPPPGGPAGTGVAAAVGSGPPDSGAPKKDSIVSLPELDSARSRLSSNL

>HCN3_green_puffer

MDGVAGGVGTPSSVGGSGGDSLPRRNGESKRRSKGSLPSPGYRLSQASLEGERASFGADNTSLGGRGRRLSIMSSSTRDGLPFRTAGTPTTPVPLPPPPPSSSSATAHPPQRSVGFASSRAALASTSSTGTGVMVVATGLETTTTTACNTTAAGTPEMVGQSGLGGFGMGLDGEDYSTSNQSTFIQRQFGAMLQPGVNKFSLRMFGSHKAVAQEQERLKSAGAWIIHPYSDFRFYWDLLMLMLMMGNLIILPVGITFFRDENTPSWIIFNVVSDTLFMVDLVLNFRTGIIKEDNTEILLDPRAIRQKYLKTWFLVDFVSSIPVDYIFLMVDSLDSEVYRTARALRIVRFTKILSLLRLLRLSRLIRYIHQWEEIFHMTYDLASAMVRIVNLIGMMLLLCHWDGCLQFLVPMLQDFPPDCWVSKNLMVNDTWGVQYSYALFKAMSHMLCIGYGAQAPEGMTDVWLTMLSMIVGATCYAMFIGHATALIQSLDSSRRQYQEKYKQVEQYMSFHKLPADVRQKIHEYYEHGFQGKMFDEENILGELSEPLKEEIVSFNCRSLVANMPLFANADPNFVTAVLTKLRFEVFQPSDFIIREGTVGRKMYFIQHGRVSVLTRGNKETKLSDGSYFGEICLLTRGRRTASVRADTYCRLYSLSVDSFNEVLEEHPMMRRAFETVAVDRLDRIGRKNSMHLRKSSQGSLGSCDSILVQQIVKHDSMPAMQDAIAAAAAAGRGGVAGGSGTVSPRPRPVIWAPLVHAPLQTAAATSNVAIALMHQQQQQQHQFQQLQQQHALGGAFFLPSPLISPSPSSSFPLSPPRAPVLQPLRPSVSSLIGMMAMGGIGGLSPRGAFPASPSTMGPPSGLTSPPVAKTPPMQACSVPAPVQQGRTLHNAPRLPADHSAVAAGPLGGPPTPPFHKIPPTNTAGPASLSDGNAPAAAQQGAKEALLRGGGGSQGLPALGRLTKEARLLSASQPTLPHRSWAGVQPHPPLHRKASGGNLLATPFLAGQLARGGSTDVLTCNPPAQLATNTPFHAQAHTQATVPIQSTTTHTHSGPHSGPVPAPTPAHVSPAAPLSCSSPPRHSPLSSCTPSPSPTRLSPIPAPSQPPCPKRIPMAAPCS

>HCN2b_green_puffer

MICNKNGDCRRDPTSSGSLSSLISRQERQADGVSAPAAEDSEAAAMDGSVTSAGSLSQAPGAEVRTTAAAEKKDSRVSFSRAPSGKGQTPGQQARTSVTFSKAEDGSQIGPDDGESRGGQTYMQRQFSSMLQPGVNKFSLRMFGSQKAVEKEQERVKSAGNWIIHPYSDFRFYWDFTMLMFMVANLIIIPVGITFFKDETTTPWIIFNVVSDTFFLMDLVLNFRTGIVFEDNTEIILDPDKIKKKYLKTWFVVDFVSSIPVDYIFLIVEKGIDSEVYKTARALRIVRFTKILSLLRLLRLSRLIRYIHQWEEIFHMTYDLASAVMRIINLIGMMLLLCHWDGCLQFLVPMLQDFPSDCWVSLNKMVNDSWSELYSFALFKAMSHMLCIGYGRQAPESMSDIWLTMLSMIVGATCYAMFIGHATALIQSLDSSRRQYQEKYKQVEQYMSFHKLPADFRQKIHDYYEHRYQGKMFDEESILEELNEPLREEIVNFNCRKLVASMPLFANADPNFVTAMLTKLRFEVFQPKDYIVREGTIGKKMFFIQHGVVSILTKGNISMKLMDGSYFGEICLLTRGRRTASVQAETYCRLYSLSVDNFNEVLEEYPMMRRAFETVAIDRLDRIGKKNSILMHKVQHDLNSGVFNNRENEMIQEIVKYDREMVQQVDVPRPRAMSLTPPLLEGMPPPPGCTQNSAIASLQQAVAMSFCPPKMLRRLQANEGVSSPASSSPLQPQLLPSGAFTPMLCSPPVQSPLAFGGRSFQYGSSGLAGPTSGSQLSLTQQHLPSPAHRPSTHKSTHSLPMGSLSQDARALSASQPSLPHEGAQLHGPSPPQSTRVSSTSIGPPQMPSGHTGIRNAVAHRVVLPHQMSVGAFPLGSLPGSAHSFGAGAGLVVGGAGTVLKDSGLPKKDSIASIPETDRIRVRSRLSSNL

>HCN2_zebrafish

MDEAEDGDEDHKETRRGDILDSSSKMKTVAPGGGAASSRISNNKDFASCGRSTESAALLGHGDASRSGAHSDGEDATGMRCVINGDCRRDDSVCSVLSKLEKQTGGGFPASACHSSTSSMDGSVTPAAAAAPPADKKDSRVSFSSAAPAHGPSPSNPAGNSVSFSKSEDGQITAEDGEARDNQTYMQRQFSAMLQPGVNKFSLRMFGSQKAVEKEQERVKSAGNWIIHPYSDFRFYWDFTMLMFMVGNLIIIPVGITFFKEETTTPWIIFNVVSDTFFLMDLVLNFRTGIVYEDNTEIILDPEKIKKKYLKTWFVVDFVSSIPVDYIFLIVEKGIDSEVYKTARALRIVRFTKILSLLRLLRLSRLIRYIHQWEEIFHMTYDLASAVMRIINLIGMMLLLCHWDGCLQFLVPMLQDFPSDCWVSLNKMVNDSWSELYSFALFKAMSHMLCIGYGRQAPESMSDIWLTMLSMIVGATCYAMFIGHATALIQSLDSSRRQYQEKYKQVEQYMSFHKLPADFRQKIHDYYEHRYQGKMFDEESILEELNEPLREEIVNFNCRKLVASMPLFANADPNFVTAMLTKLRFEVFQPGDYIIREGTIGKKMYFIQHGVVSVLTKGNIGMKLSDGSYFGEICLLTRGRRTASVRADTYCRLYSLSVDNFNEVLEEYPMMRRAFETVAIDRLDRIGKKNSILMHKVQHDLNSGVFNNRENEMIQEIVKYDREMVKLVKQGDMQRPRAMSMTPSTHGSMFGPSSQPSTSSAIATLQQAVAMSFCPQMGNPLMGSGSVQSPRMVRRFQVVQTQTPPGSQYSTLARASALASTPVHSPLATTARTFQYGSSPPGAPSGSQLSLVQQLVPSPTHRPAVHRSTLTQDARALSASQPSLRPDMIQPVAPSPPQSTRVSSTSIGPPQNLPGPTGLRGSIPPRMALTHQMSVGAFPPASLPQMRPSLDSGLPKKDSISSLPETEQHHIRSRLSSNL

>HCN4_zebrafish

MHRVHSSMRKRLYSLPHQFGHKGAMTRDGEDSDKDTRRKSVKMKHFPSPSSTGSSKGVCEAKSGDAETDVGRPFRTNVNGDCRRFRGSLSSLTSRHAAPDADLAEQRRLIGDASPEDDGPSEQPGPGESPGAKGGRGDGAQQAPSEEQSTFVKLEGIDQILPDDERMYQAGFMHRQFGAMLQPGVNKFSLRMFGSEKAVEHEQERVKSAGFWIIHPYSDFRFYWDLIMLLLMVGNLIIIPVGITFFKDEHTPAWIVFNVVSDTFFLVDLVLNFRTGIVKEDSTEIILDPQQIKVRYLRSWFAVDFLSSIPVDYIFLIVETRIDSDFYKTARALRIVRFTKILSLLRLLRLSRLIRYIHQWEEIFHMTYDLASAMVRIVNLIGMMLLLCHWDGCLQFLVPMLQDFPSDCWVAKNKMVNDTWGQQYSYALFKAMSHMLCIGYGMYPPVGMTDVWLTILSMIVGATCYAMFVGHATALIQSLDSSRRQYQEKYKQVEQYMSFHKLPADMRQRIHDYYEHRYQGKMFDEESILGELNEPLREEIISFNCRKLVATMPLFANADPNFVTSMLTKLRFEVFQPGDYIIREGTVGKKMYFIQHGVLTVLTKGSKETKISDGSYFGEICLLTRGRRTASVRADTYCRLYSLSVDHFNEVLEEYPMMRRAFETVALDRLDRIGKRNSVLQHKVQRDLNSGVLNYQENEIIQQIVQHDRDMAHCAHLMQTPPPPPHPAPTPSSHTPVIWAPLIQAPLQAAAATTSVAIALTRHPHLPHTLFRPPVPRLGSLKDQPGYFKRFPTALSGADTFGGSSSTSSQFSSCIETPILDTLRAERSSTSTPPPSSSSLPSTITTTVTTTTSSITESSVYHHRPIISHGSKDFSVAQLHSQPQLSQASFSSIAASLPGFFQQGAAGGEAVHLAVPPASTLTPLIAHSVSPILTQLHATHSNPQPTKPIQDLNKAGRTAPQSPVTESPVHSKSILEQVQSPTSVQSQHPQIGIQPDVLSQLAQEPSALASLAQYGSGNASPCSTPSAWSPTCQSPTAEKMRMSAHSQPSSISGSQTSLLTPQTLFPPPPQPRERGGSLVDLPSQDLSTLSMSLPIPGLAEKSRAVGRGPQPPDLSHHRGSTSGYSPLPSPPPLVNPFSSIPGHVVLSRQTSKLSITQTGSSGGSPAHGSSDRSTPGTPSLHPKLPSNL

>HCN3_zebrafish

MDGDGQPPASNGDSKLRSRGGFSLTGWRSSSKTLLRGEAEDVSRRLLSIVNHNQERSDSGASPTSKSLEVTAGLDGTTATTTTVTAATPPLPHKAMPESSGAPVASEAEDGPFVDQSTFLQRQFSSMLQPGVNKFSLRMFGSAKGVAAEQDRVKSFGVWIIHPYSDFRFYWDLVMLCLMMGNLVILPWGITFFEDQNTLPWITFNVASDTLFLADLVFNFRTGIMEGDNSEIILDPQIISRRYLRGWFLVDFISSIPVDYIFLIVDIESRLESAEVYRTARALRIVRFTKILSLLRLLRLSRLIRYIHQWEEIFHMTYDLASAVVRIVNLIGMMLLLCHWDGCMQFMVPMLQDFPSTCWVSKNNMVNSTWDVQYSYALFMAMSHMLCIGYGAQAPEGPTDVWLTMISMIIGATCYAMFLGNATNLVQSLDASHRQYQEKYKQVEQYMSFHKLPADMRQRIHDYYEHRFQGKMFDEENILEELSDPLKEEIVNYNCRGLVANMPLFANADPHFVTVLLSKLRFEVFQPGDVIIREGTLGRKMYFIQHGCVSVITSDNKEKKLNDGCYFGEICLLTRGRRTASVRADTYCRLYSLSVDSFNEVLEENPLMRRAFENVAVNRLGRES

>HCN1_fugu

MEDKSNSFSSGKEEKADGNNVFQRQDSIQKNNTGSQNTKEHSNSVSFKGEREEAMVGFDDLEGASRQHGFMQRQFGAMMQPGVNKFSLRMFGSQKAVEKEQERVQTAGYWIIHPYSDFRFYWDLIMLIMMMGNLIIIPVGITFFSDQNTTTWLVFNVASDTIFLVDLVMNFRTGIVNEESSEIILDPKVIKMNYLKSWFVVDFLSSIPVDYIFLIVEKGFDSEVYKTARALRIVRFTKILSLLRLLRLSRLIRYIHQWEEIFHMTYDLASAVVRIFNLIGMMLLLCHWDGCLQYLVPLLQDFPPDCWVSLNGMVNVSWGKQYSYALFKAMSHMLCIGYGARAPVSMSDLWITMLSMIVGATCYAMFVGHATALIQSLDSSRRQYQEKYKQVEQYMSFHKLPADMRQKIHDYYEHRYQGKIFDEDNILSELNDPLKEEIVNFNCRKLVATMPLFANADPNFVTGMLSKLKFEVFQPNDYIIREGTVGKKMYFIQHGVASVITKSNKEMKLTDGSYFGEICLLTKGRRTASVRADTYCRLFSLSVDHFNEVLEEYPMMRRAFETVAIDRLDRIGKKNSLLLQKFQKDLNAGVFNTQENEMLKQIIRQDREMVMMVDRKQSVTGMNSTPASGNSIINSPAQPLFPTAFGFSQLQQSSAPMAYSASAIANASAARMLPVAAAAAAAAQAGYPPPSLSHSNLNSSLTNPQGVLQQGAIMSPCSFTAAMCSPPVQTPLANRSFQYGSRTASQLSLIQQPIGQPTLPLQQQLAQQPPAAAAASVPQQQQVPSPQRGDIPKGAQALQSGSLSRDVRHLSASQPSLPHDTSLGPRAHPASGDSLASIVPPTAAAIQGMSLQGGIRTTVPQRVNLFRQMSSGALPPVRSAAAAAAAAAAASSSSAQHRESAGSRRDSILSSTETEQDKMRFSSNL

>HCN2a_fugu

MKCNKNGECRRDSGTGSLRSIISKQEKQSGGSGRAEDGGSAKRGARNSTSASMDGSITPGGSLTGGQGGESVNPSVTPVSSTVPDKKDSKVSFSNAPSRKASSSLAQTQTQQPRNSVTFQKPEDGPPIVPAEDAEARGSQASFMQRQFSSMLQPGVNKFSLRMYGSQKAVEREQERVKSAGNWIIHPYSDFRFYWDFTMLLFMVGNLIIIPVGITFFKEETTTPWIIFNVVSDTFFLMDLVLNFRTGIIIEDNSDIILDPKTIKKKYLKTWFIVDFISSIPVDYIFLIVEKGIDSEVYKTARALRIVRFTKILSLLRLLRLSRLIRYIHQWEEIFHMTYDLASAVMRIINLIGMMLLLCHWDGCLQFLVPMLQDFPSDCWVSLNKMENDTWSELYSFAVFKAMSHMLCIGYGRQAPESLSDIWLTMLSMIVGATCYAVFIGHATALIQSLDSSRRQYQEKYKQVEQYMSFHKLPADFRQKIHDYYEHRYQGKMFDEESILGELSEPLREEIVNFNCRKLVASMPLFANAEPNFVTAMLTKLRFEVFQPHDYIIREGTIGKKMYFIQHGVCSVITKGTLAMKLSDGSYFGEICLLTRGRRTASVRAETYCRLYSLSVDHFNEVLEEYPMMRRAFETVAIDRLNRIGKKNSILMHKVQHDLNSGVFNNQENEMIQEIVKYDREMVKLVDLQRPRAMSMTPSIGGIFAPPGQPHTGSAIATLQQAVAMSFCPQMASPLVGPGTLQSPRMVRRFQVMQNLSSPVSMSPLQSQPPQAFGGAFGSAISSPPVQSPLTATGRTFQYMASSGPSGSQLSLVQHHVPSPTQTQQRPASHKSTHSLHAGSLSQDTRALSASQPSLPQEGTPQAASAAPSPPASTHTSNNSIGPPQPPHPNLPGPSGVGRAAGGQQRVAFASTPPPGGPTGMGVAAAVGSGPPDSGAPKKDSIVSLPELDSARSRLSSNL

>HCN2b_fugu

MICNKNGDCRRDPTSSGSLSSLISRQERRADGVTTPSAEDGTAAVMDGSVTSAGSLSQGPGAEVKTTATSEKKDSRVSFSNAPAGKGQTLSQHSKNSRISFSKAEDGSQIGPDDGESRGNQTYMQRQFSSMLQPGVNKFSLRMFGSQKAVEKEQERVKSAGNWIIHPYSDFRFYWDFTMLMFMVANLIIIPVGITFFKDETTTPWIIFNVISDTFFLMDLVLNFRTGIVFDDNTEIILDPDKIKKKYLKTWFVVDFVSSIPVDYIFLIVEKGIDSEVYKTARALRIVRFTKILSLLRLLRLSRLIRYIHQWEEIFHMTYDLASAVMRIFNLIGMMLLLCHWDGCLQFLVPMLQDFPSDCWVSLNKMVNDSWSELYSFALFKAMSHMLCIGYGRQAPESMSDIWLTMLSMIVGATCYAMFIGHATALIQSLDSSRRQYQEKYKQVEQYMSFHKLPADFRQKIHDYYEHRYQGKMFDEESILEELNEPLREEIVNFNCRKLVASMPLFANADPNFVTAMLTKLRFEVFQPKDYIVREGTIGKKMFFIQHGVVSILTKGNISMKLMDGSYFG

>HCN3a_fugu

MDGVAGGVGTPSSVGGSGGDSLPRRTGEAKRRSKGSLPSPGYRLSQASLEGERASFGADTVSLGGRGRRLSIMSSSTRDGLPFRTAGTPTTPVPLPPPPPSSSSSATAHPPQRSVGFASSRAALASTSSTGTGVMVVATGLETTTTTACNTTAAGSPEMVGLSGLGGFGVGLDGEDYSTSNQSTFIQRQFGAMLQPGVNKFSLRMFGSHKAVAQEQERLKSAGAWIIHPYSDFRFYWDLLMLMLMMGNLIILPVGITFFRDENTASWIIFNVVSDTLFMVDLVLNFRTGIIKEDNTEILLDPRAIRQKYLKTWFLVDFVSSIPVDYIFLMVDSLDSEVYRTARALRIVRFTKILSLLRLLRLSRLIRYIHQWEEIFHMTYDLASAMVRIVNLIGMMLLLCHWDGCLQFLVPMLQDFPPDCWVSKNLMVNDTWGVQYSYALFKAMSHMLCIGYGAQAPEGMTDVWLTMLSMIVGATCYAMFIGHATALIQSLDSSRRQYQEKYKQVEQYMSFHKLPADVRQKIHEYYEHRFQGKMFDEENILGELSEPLKEEIVSFNCRSLVANMPLFANADPNFVTAVLTKLRFEVFQPSDFIIREGTVGRKMYFIQHGRVSVLTRGNKETKLSDGSYFGEICLLTRGRRTASVRADTYCRLYSLSVDSFNEVLEEHPMMRRAFETVAVDRLDRIGRKNSMLLRKSSQSLGSCDSILVQQIVKHDSMPAMQDAIAAAAAGRGGVTGGSGTVSPRPRPVIWAPLVHAPLQTAAATSNVAIALMHQQQQQQQQFQQLQQHNALGGAFFLPSPLISPSPSASFPLSPPRAPVLQPLRPSVSSLIGMMAMGGLGGLSPRGAFPASPSTMGPPGGVTSPPIAKTPPMQACSVPPPVQQGRTLHNAPRLPADHSAVTTGSLGGSPSPPFHKIPTTLSTASASQPEGNVQVTGQQGAKEALLRGGSSSQGLPALGRLTKEARLLSASQPTLPHRSWAGVQPHPPLHRKASGGNLLATPFLAGQLARGGSSDMLTSNPPTQLVTNTQFNAQRYTPATVPVQPATTYAQSGPHTGSVPTPAPAHISPAAPLSSSSPPKQTPLSSCTPSPSPTRLSPTPGPSQPPCPIRIPMPPPCS

>HCN3b_fugu

MTSPCQSAEGRRGINESPAHKPETPRYRSWSSFRFSRWRSSSQRSTPPATPSPKTDKRGDVTKTLFSLVKTHNDDYTADPDGLLEGDVAHEEGNNGAHQDHSSYFHWHFGSMLQPGVNKFSLRMFGSHKGVAAEQERVKSFGVWIIHPYSDFRFYWDIVMLVLMMSNLVILPWGITFFEDQNTLPWITFNVLSDTLFLMDLVLNFRTGIPGEDSHIILDPKEIRMHYLRTWFLVDFVSSIPVDYIFLIVDLESRLDSTDLYRTTRALRIVRFTKILSLLRLLRLSRLIRYIHQWEEIFHMTYDLASAVVRIVNLIGMMLLLCHWDGCLTFMVPMLQDFPPDCWVSKNNMVNSTWHTQYSYSLFMAMSHMLCIGYGAHPPEGISDVWLTMVSMVVGATCYAMFLGHAANLVQSLDASHRQYQEKYKQVEQYMSFHKLPADVRQRIHDYYEQRFQGKMFDEDSILGELSDPLKEEIVSYNCRGLVANMPLFANTDPHFVTVILTKLRFEVFQPGDLIIREGTLGRKMYFIQHGAVTVIPRGTKEITLNDGAYFGEICLLTQGRRTATVRADTYCRLYSLSVDNFNEVLEEHPLMRRAFESVAVDRLGRIS

>HCN4a_fugu

MERFQSSMRKRLYSLPQDIGQKPFVVDEGDSGDKDTKRKSIRLKHLSSLSPASSCRSIEEATVKTNLNGDCRFFRGSISSITGRHPPGSDPAEQQRLIPGADVGVSESFEEGPAAQQRATCGLSGATGQASAFDQSGFIKLEGTEQIIPEDDRLYQAGFMHRQFGAMLQPGVNKFSLRMLGSERAVEHERERVKSAGFWIIHPYSDFRFYWDLTMLLLMVGNLIIIPVGITFFKDEHTPPWIVFNVVSDTFFLMDLVLNFRTGIVKEDNTEIILDPQKIKIKYLKSWFMVDFISSIPVDYIFLIVETRINSDFYKTARALRIVRFTKILSLLRLLRLSRLIRYIHQWEEVFHMTYDLASAMVRIMNLIGMMLLLCHWDGCLQFLVPMLQDFPPDCWVTRNKMVNDTWGQQYSYALFKAMSHMLCIGYGLYPPIGMADVWLTILSMIVGATCFAMFVGHATALIQSLDSCRRQYQEKYKQVEQYMSFHKLPADMRQRIHEYYEHRYQGKMFDEESILEELNEPLREEIINFNCRKLVASMPLFANADPNFVTSMLTKLRFEVFQPGDYIIREGTIGKKMYFIQHGVVSVLTKSSKDTKLSDGSYFGEICLLTRGRRTASVRADNYCRLYSLSVDNFNEVLEEYPMMRRAFETVALDRLDRIG

>HCN4b_fugu

MDRLHSSMRKRLYSLPQHIGHKASIMSEGEDADKDTRRKSIRMKPLPSPTSAGSCKGIGETKSGESGIMETDIGRPMKTSSNGDCRRFRGSLSSITSRHVHESDSAEERRLITEGDVTPSEESPLGAIGDGPPEQGAQGAGGGGGTQKDSPDQQPGFVKLDGIDQILPDDERLYQAGFMHRQFGAMLQPGVNKFSLRMFGSEKAVEREQERVKSAGFWIIHPYSDFRFYWDLTMLLLMVGNLIIIPVGITFFKDEHTPPWIVFNVVSDTFFLMDLVLNFRTGIVKEDNTEIILDPQQIKIKYLRSWFVVDFISSIPVDYIFLIVETRIDSDFYKTARALRIVRFTKILSLLRLLRLSRLIRYIHQWEEIFHMTYDLASAMVRIVNLIGMMLLLCHWDGCLQFLVPMLQDFPADCWVSKNKMVNDTWGQQYSYALFKAMSHMLCIGYGMYPPVGMTDVWLTILSMIVGATCYAMFVGHATALIQSLDSSRRQYQEKYKQVEQYMSFHKLPADMRQRIHDYYEHRYQGKMFDEESILGELNEPLREEIINFNCRKLVASMPLFANADPNFVTSMLTKLRFEVFQPGDYIIREGTIGKKMYFIQHGVVSVLSKGNKETKLSDGSYFGEICLLTRGRRTASVRADTYCRLYSLSVDNFNEVLEEYPMMRRAFETVALDRLDRIGKKNSILQHKVQHDLSSGVLNYQENEIIQQIVQHDRDMAHCAHLMQNVPPQTPPSPTPVIWAPLIQAPLQAAAATTSVAIALTHHPHLPHTLFRPPVSVLGSLKDPSSRMKKLHTFAPSPAPGSAGDSPASTPSKLHTGVDMPLLASFRAQHMPSGTLITGSSKQGSSSEDQQQPNTSNSGLGGGGLSAFPFEQFSSSGSPFSSTAQLHPQPKHQQPFRSETPPLSNLFHQGSAGGSTGSGLSGGAGGVFGTTGWSSGGAVGDAVEGATGGDTSWAGDDFTIGTADLNPGSHFGMGIGSRGSVGEISGESIGRISEGLMGAAFGRLGCGASGASGLATCGPIGDVARSLQGTNRGSTGTSSGGSVGSSGETLAAVTAEGSLGRVSGRSKGVLSRGMTIGSTGESHGSVLGDSVGLTSGGPFGRASVGTLVSHMSVGSSSSGGGVPLGHPSRAAGGSHIGLSTCVVGVSSSGVTTGHVTSSRCQSPIFTSSSSFAPSQVPHSQPSAKPPQVSPLQQFAGGGRTTGDLSLLHSPPQGSPSTRRRFYSQQPAEGSPSSLSHFNLTGLSSGILPHQMSIERSALASLAQYGSTNASPCLTPVAPSPTIQSPVAGRTFHYSDPPSITGSNSSLPMPHSSFPSQLPSQSPKTGRDSPLGRFSEDLKLLSSSHPSLPQEVVPALGHHAPCSSVETGYYPSPFSSPTLVPKPCSPMPGRMTPPIQTSPGHRQQTQSPRVSPALPLQRGSSAYSTDLETQTVRSKLPSNL

>ciHCNa

MPPKRKVGSKPNESKNDPTPEVPQIFLATESSSDVPQFSVQVDDHDVEASTNNLTNNMVIASEPTVSYLAMNMDPGLVRRKSQQFEANIKAEEEEHVREALEKERKQVEKLVSSRSVTQGSISSAMHLHPDKSSFRRGVTGSMSIPMNKMSLKLYGSEKAVIEEQERLKKAGSWIIHPYSNFRFIWDSFTLVLLLVNIILIPVIISFWKDDDSAWLPFKAVSDTWFLTDIIINFRTGIVIDGPDSEVILDPKQIRMMYLKTWFTIDLVSTFPFDLVFTIIDGASSMAETGLKALSLLRFAKILALLRLLRLSRFIRYMKQWEEIFNFQYEMALAFARIFNLIMLMLFICHFNGCLQYMVPMFLDFPEKTWVRDRNLHLSNVTIWERYSWSVFKSTSHMLCIGYGLFPPQGLVDVWVTYVSMCSGAMCFALFIGNATSLIQSMDASKRAYKEKYMQVKEYMQFRKLPSGLRHRISDYYENRFQGKMFDEERILTELNHNLRDEVIQYNCKDLVDQVPFFNEADPSFVAAMLGKLNFEVFLNDEVIVKEGTEGKKMYFINRGTVTIKSAQHKIEQSLSDGCYFGEIALLQQNLRRVASVTAETYCYLYSLSVDDFNEVLKEFPRQRAKLQHVAQARLKDNTAQDSFTSTCSIIPLDGPRERSQPGSSNLSEITEESSGMLDDYNKSAKKK

>ciHCNb

MSVPVTKFSLRMYGSQKAVEQEQKRQESAGSFVIHPYSNFRFYWDFFTLILLLISMIIIPVAITFFNDEMRTDSGWIAFNLCLDFWFLSDIIMNFHTGIIVEYGDGDVVLDLPTIRSRYLRSWFVIDLISTLPVDYLLQLTSGSSASASASRAMKLVRFAKIISLLRLLRISRLIRYVHQWEHIVGMQYDLAVAAVRIFNLVCLMLLIGHWNGCLQFLIPMLHNYPADSWIVIDKLVGKPWAEQYSWSMFKAMSHMLCIGYGQQPPKNLMDLWMTMLSMVSGAVCFAMFIGHATALIQSMDSSKRQYKEKYMQVKEYMRFRRLPKTLRSKVYEYYENRFQGKMFDENGILSELSTNLREEVVNFNCRHLVASVPFFSNAESDFVTEVVQKLKYEVFQPKDVIVREGEIGKKMYFIQHGLVEVKNSHRSEPIKQLSDGSYFGEICLLINDRRVASVEAVSYCSTYSLHVNDFNYLLSEHPVMRKTLERVAAARLSSLGRHNPVLEEKVKESTNGYSKSNEIAEEIVRHDTQLLTEKAAYLGSSCNRLNFRERTSSNASCNRLSVRERASHCNESPSRRIKTSVSLGTDMSHFASGLFPSSSKRQRHRTANKEEEITDIEMTNDIEKCDDILEVTCKEPESPRSKSPRPFRKPKFLQNLTLQRSKLYESTIKGKDSVIESFLNNVTRPRSKQQCLSSNGCSSPHISTARRSAATEERSFVEPTITIVNVEQSNDENDNCDANGTSHNKKIVLSKADVQCTDPLNNVLKLPDLNNTPKQSDNKTCLLGERCPKDIESRCEHMIDEII

>ciHCNc

MSTTEWINEFVPITSTTDSIGSQSEHIRRFSVAGLMKFPRKSINSIKERLSKRKGPSTKSQSISDTALLRAPNARTSRTAKGTSSLASTKSAVSVATRAVPFTKWSRKLYGSEKAVQDEHLRVIEAGGFIIHPFSNFRFTWDLLSALLIIANIIIIPMDLAFSGDRREVASMAFKLISDAWFLIDIILNFRTGISVIGTDSTIIELDPAKIRNRYLKGWFAIDFIASFPMDFILTYVVGQAPGKSHKAISLLRVGKGLSLIRVARIPRLIRGLHQWEEVFNLQYDMAVSLLRLAYLIFIIFLVCHWMGCLQYMVPMYYGFPEDTWVRMRGLDNPNITWWEAYSWSLFKSTSHMLCIGYSEVIPIGLIDLWMTMLSQIVGAILFAVFIGNAINLMEEMDASKNAYKMKLSQITEYLAFRRIPVKLRRKILDYFDIRYTGRLFDEEKILKELSPGLRRDIIWHNCADLIHSVPLFEDVSDGFIEDLGTKMVFTVYTADAEVVSEGEKAFHMFFILRGELVIEASDGSFMREISDGMHFGETCLLNQELRRAASVKAATNVHMYTLHRHDYQQVALRHPDDKRRIDTRFAKLRERSSQYDFIQKCQSNFKI

>HCNa_csavignyi

MSSKRKVGSKNDDENKGNEIQTPKIFIASESTDNFADVKIKHDYDVEAPAAAETTTLNMNTSPEPAVSYLAMNMDPGLVRKKSKQFEENMKIQDELKTKEALEKERIKVEKLVSAQSVGQQSISSNMYLHPDKSAFRRGVTGSMSIPMNKMSLKLYGSEKAVMEEQERLQKAGNWIIHPYSNFRFIWDSFTLVLLLVNIILIPVAISFWKEDDSSWMPFKAVSDLWFLTDIIFNFRTGIVIDGPDSEVILDPKQIRKMYLKTWFTIDLVSTFPFDLLFTIIEGATPMAESGLKALSLLRLAKILALLRLLRLSRFIRYVKQWEEIFNFQYEMALAFARIANLMMLMMFICHFNGCLQYMVPMFFQFPEKTWVRDRNLHLDNVTIWERYSWSVFKSTSHMLCIGYGLFPPQGLIDLWVTYMSMATGALCFALFIGNATSLIQSMDASKRAYKEKYMQVKEYMQFRKLPGNLRLRISDYYENRFQGKMFDEERILQELNHNLREEVINYNCKDLVDQVPFFNEADPSFVSAMLSKLVFEVFLIDETIVKEGTKGRKMYFINRGTVTIKSEQHNIEQNLSDGCYFGEIALLQQNLKRVASVIADTYCYLYSLSVDDFNEVLKEFPRQRAKLAHVAKARMKDNKAQDSFTSTCAIVPLDGPSNRTSNGYLDEITEESSAMLDDYNNVKKS

>HCNb_csavignyi

MSVPVTKFSLRMYGSKKAVEQEQKRQQSEGSYVIHPYSNFRFYWDFFTLILLLISMLIIPVAITFFNDEMRTDRGWIAFNLALDFWFLTDIIMNFHTGIIVEYGDGDVILDLPTIRSHYLRSWFAVDLISTLPVDYLLQLTSGSSASASAASRAMKLVRFAKIISLLRLLRISRLIRYVHQWEQIVGMQYDLAVAAVRIFNLVGLMLLIGHWNGCLQFLIPMLHNFPADSWIVIDDLVGRPWAEQYSWSVFKAMSHMLCIGYGQKPPKNLMDLWMTMLSMVSGAVCFAMFIGHATALIQSMDSSKRQYKEKYMQVKEYMRFRRLPKPLRSKVYEYYENRFQGKMFDELGILNELSNNLREEVVNFNCRHLVASVPFFSNAEPDFVTEIVQKLKYEVFQPKDVIVREGTIGKKMYFIQHGLVEVKNSQRSDPIKQLSDGSYFGEICLLINDRRVASVEAVSYCSTYSLHADDFNYLLNEHPVMRTTLERVAAARLSSLGHRNPVLDEKVKESTNQDSKSDEMAKEIVRHDTKVYIEKSVSLGSEMSDSNTGKAPKAGSKFSTRRVSGNKDHCNGISEVTCEDDTARSHTPRPFRRPKFLHNLAMQRAKLTESTIKGKESVIESFSNNVLKPRGKQPFGALNGSNTLKSTTARAHPTTPERSFTEPRITVVNVELENEENGDFTGVEDTDQNKTIIVSKADVLCSESNNCITETPVENKSRERVGSATCLLDECLAEDIESRCEHMIDEII

>HCNc_csavignyi

MFRVSEKMSAISWMNDVAPTSSDNGSLSSHEHMRRFSIAALMKLPRKSINSIKERLSKKKGHPSKRQSVSDTALLRTPNSRSSRTARMTSSLASTKSSAVSVATRTIPFTKWSRKLYGSKKAIEDEHERIINAGSFVIHPFSNFRFTWDVLSAVLILLNIIIIPMDLAFSGDRQEVASMAFKLISDVWFLIDIILNFRTGISVIGTDTMLIELDPAKIRKRYLKGWFAIDFIASFPIDFILTFLVGYSPGKSHKAIGLIRVGKAFSLIRVARIPRLLRGLHQWEEAFNLQYDMAVTMIRFIYLMFISIMICHWNGCIQYMVPMYYDFPDDTWVKIRNLDGPNTTWFEAYSWSFFKAVSQMICIGYSEVIPVGMIDLWTTMLSQCTGAIYFAFFIGNTINLMEEMDASKNAYKLKISQIQEYLSFRRLPLKLRRRIMDYFDLRYSGRIFDEEQILSELSPGLRRDIIWHNCADLIQSVPLFDDVSDDFVADLGSVMVYTVYSSDTEVVREGEMAFHMFFILRGELVISASDGSFLREISDGMHFGETCLLNEELRRAASVKAATNVHMYTLHRHDYQEVASRHLEDKEKIDTRFAKLRERTSQYDFIQKSSVKQ

>hCNGA1

MKNNIINTQQSFVTMPNVIVPDIEKEIRRMENGACSSFSEDDDSASTSEESENENPHARGSFSYKSLRKGGPSQREQYLPGAIALFNVNNSSNKDQEPEEKKKKKKEKKSKSDDKNENKNDPEKKKKKKDKEKKKKEEKSKDKKEEEKKEVVVIDPSGNTYYNWLFCITLPVMYNWTMVIARACFDELQSDYLEYWLILDYVSDIVYLIDMFVRTRTGYLEQGLLVKEELKLINKYKSNLQFKLDVLSLIPTDLLYFKLGWNYPEIRLNRLLRFSRMFEFFQRTETRTNYPNIFRISNLVMYIVIIIHWNACVFYSISKAIGFGNDTWVYPDINDPEFGRLARKYVYSLYWSTLTLTTIGETPPPVRDSEYVFVVVDFLIGVLIFATIVGNIGSMISNMNAARAEFQARIDAIKQYMHFRNVSKDMEKRVIKWFDYLWTNKKTVDEKEVLKYLPDKLRAEIAINVHLDTLKKVRIFADCEAGLLVELVLKLQPQVYSPGDYICKKGDIGREMYIIKEGKLAVVADDGVTQFVVLSDGSYFGEISILNIKGSKAGNRRTANIKSIGYSDLFCLSKDDLMEALTEYPDAKTMLEEKGKQILMKDGLLDLNIANAGSDPKDLEEKVTRMEGSVDLLQTRFARILAEYESMQQKLKQRLTKVEKFLKPLIDTEFSSIEGPGAESGPIDST

>hCNGA3

MAKINTQYSHPSRTHLKVKTSDRDLNRAENGLSRAHSSSEETSSVLQPGIAMETRGLADSGQGSFTGQGIARLSRLIFLLRRWAARHVHHQDQGPDSFPDRFRGAELKEVSSQESNAQANVGSQEPADRGRSAWPLAKCNTNTSNNTEEEKKTKKKDAIVVDPSSNLYYRWLTAIALPVFYNWYLLICRACFDELQSEYLMLWLVLDYSADVLYVLDVLVRARTGFLEQGLMVSDTNRLWQHYKTTTQFKLDVLSLVPTDLAYLKVGTNYPEVRFNRLLKFSRLFEFFDRTETRTNYPNMFRIGNLVLYILIIIHWNACIYFAISKFIGFGTDSWVYPNISIPEHGRLSRKYIYSLYWSTLTLTTIGETPPPVKDEEYLFVVVDFLVGVLIFATIVGNVGSMISNMNASRAEFQAKIDSIKQYMQFRKVTKDLETRVIRWFDYLWANKKTVDEKEVLKSLPDKLKAEIAINVHLDTLKKVRIFQDCEAGLLVELVLKLRPTVFSPGDYICKKGDIGKEMYIINEGKLAVVADDGVTQFVVLSDGSYFGEISILNIKGSKSGNRRTANIRSIGYSDLFCLSKDDLMEALTEYPEAKKALEEKGRQILMKDNLIDEELARAGADPKDLEEKVEQLGSSLDTLQTRFARLLAEYNATQMKMKQRLSQLESQVKGGGDKPLADGEVPGDATKTEDKQQ

>hERG1

MPVRRGHVAPQNTFLDTIIRKFEGQSRKFIIANARVENCAVIYCNDGFCELCGYSRAEVMQRPCTCDFLHGPRTQRRAAAQIAQALLGAEERKVEIAFYRKDGSCFLCLVDVVPVKNEDGAVIMFILNFEVVMEKDMVGSPAHDTNHRGPPTSWLAPGRAKTFRLKLPALLALTARESSVRSGGAGGAGAPGAVVVDVDLTPAAPSSESLALDEVTAMDNHVAGLGPAEERRALVGPGSPPRSAPGQLPSPRAHSLNPDASGSSCSLARTRSRESCASVRRASSADDIEAMRAGVLPPPPRHASTGAMHPLRSGLLNSTSDSDLVRYRTISKIPQITLNFVDLKGDPFLASPTSDREIIAPKIKERTHNVTEKVTQVLSLGADVLPEYKLQAPRIHRWTILHYSPFKAVWDWLILLLVIYTAVFTPYSAAFLLKETEEGPPATECGYACQPLAVVDLIVDIMFIVDILINFRTTYVNANEEVVSHPGRIAVHYFKGWFLIDMVAAIPFDLLIFGSGSEELIGLLKTARLLRLVRVARKLDRYSEYGAAVLFLLMCTFALIAHWLACIWYAIGNMEQPHMDSRIGWLHNLGDQIGKPYNSSGLGGPSIKDKYVTALYFTFSSLTSVGFGNVSPNTNSEKIFSICVMLIGSLMYASIFGNVSAIIQRLYSGTARYHTQMLRVREFIRFHQIPNPLRQRLEEYFQHAWSYTNGIDMNAVLKGFPECLQADICLHLNRSLLQHCKPFRGATKGCLRALAMKFKTTHAPPGDTLVHAGDLLTALYFISRGSIEILRGDVVVAILGKNDIFGEPLNLYARPGKSNGDVRALTYCDLHKIHRDDLLEVLDMYPEFSDHFWSSLEITFNLRDTNMIPGSPGSTELEGGFSRQRKRKLSFRRRTDKDTEQPGEVSALGPGRAGAGPSSRGRPGGPWGESPSSGPSSPESSEDEGPGRSSSPLRLVPFSSPRPPGEPPGGEPLMEDCEKSSDTCNPLSGAFSGVSNIFSFWGDSRGRQYQELPRCPAPTPSLLNIPLSSPGRRPRGDVESRLDALQRQLNRLETRLSADMATVLQLLQRQMTLVPPAYSAVTTPGPGPTSTSPLLPVSPLPTLTLDSLSQVSQFMACEELPPGAPELPQEGPTRRLSLPGQLGALTSQPLHRHGSDPGS

>KAT1

MSISWTRNFFERFCVEEYNIDTIKQSSFLSADLLPSLGARINQSTKLRKHIISPFNPRYRAWEMWLVLLVIYSAWICPFQFAFITYKKDAIFIIDNIVNGFFAIDIILTFFVAYLDSHSYLLVDSPKKIAIRYLSTWFAFDVCSTAPFQPLSLLFNYNGSELGFRILSMLRLWRLRRVSSLFARLEKDIRFNYFWIRCTKLISVTLFAIHCAGCFNYLIADRYPNPRKTWIGAVYPNFKEASLWNRYVTALYWSITTLTTTGYGDFHAENPREMLFDIFFMMFNLGLTAYLIGNMTNLVVHWTSRTRTFRDSVRAASEFASRNQLPHDIQDQMLSHICLKFKTEGLKQQETLNNLPKAIRSSIANYLFFPIVHNIYLFQGVSRNFLFQLVSDIDAEYFPPKEDIILQNEAPTDLYILVSGAVDFTVYVDGHDQFQGKAVIGETFGEVGVLYYRPQPFTVRTTELSQILRISRTSLMSAMHAHADDGRVIMNNLFMKLRGQQSIAIDDSNTSGHENRDFKSMGWEEWRDSRKDGYGLDVTNPTSDTALMDAIHKEDTEMVKKILKEQKIERAKVERSSSETAGRSYANDSSKKDPYCSSSNQIIKPCKREEKRVTIHMMSESKNGKLILLPSSIEELLRLASEKFGGCNFTKITNADNAEIDDLDVIWDGDHLYFSSN

**IP3 Receptors (Figure 6):**

>Helro_71321_71772_190044

CYKILNALYTLGTHSSTFVQRHRSALGECVASFASTFPVAFLEPKLNKFNKNSIVFGIEEDRIATHSLEAKEVLDEVAINVLPLEKIVSEIEGLAESGGKYNEAPHVIEVTLPMLCSQVQVTTVTADLMNNVLGNVLKLVKNNIGTLNAPWMNKIASRTQPIIVNATTAMLKDQILPVAIKIREQARNLEKEEEEYLAEVRRMQHKKDGGEIESEVPEKYQILVRDMYAFYPLLIKYVDLHRSNWLKCPNSDAEHLYLCVADVFNLGTKSHIFKKEEQNFVFSNEIDNMALIRPSQDKHSASGATMVDGGKMDQPKTKGRKRVPKKMEVSASLNVACLKRLLPIGLNMFGGKEQELVQQAKQKLVDHSQNPEHVEGLHRDIEADVEEFLRVNFHTEEKAKEDDDAHQWQNALYKKMSHKNVDSNKQLTTDHVIARMLAMAKVLHGLHLVEHPVTDVETQWKKAVSLQRKRAVMACFRMVPLYNLSRHRAINLFLRSYKEQWLDSEPPGHSRLVEDITSTNEKAVAEEATSESSPLTQLITAFTRAATIEQSNALETDFLYISCHGEEEEEEAEAVEGEVEGETDDGGAGASLQEQEMERQKLLFEQSRLSDRGAAEMVLMYISASKGQWNDMLISTIDLGISILMGGNTGVQKKMLDHLKEKKDVGVFTSIAGLMQQCCVLDLDAFERYNKAEGLGVTEGTAQVKNLHDAEHTCKLFRLSKLLEGAGKESFIRAISVASQVFNSISEYIQGPCQGNQLALAHSRLWDAVGGFLFIFAHMQDKLSRDPEQFELLREFMTLQKDMMIMLLSMLEGNVMNGPIGKQMVDTLVESSANFELILKFFDIFLKMKDLTRSEGFMEIDVNNDGIVTPKEFRRALELQKIYTEEEIEYVMMCIDANQDGKVDFNEFTGRFHGPAKDIGFNVAVLLTNLSEHIPNDPRLERFLEKAQCVLNYFEPYLGRIEIMGSASKIERVYFEIKQDHIDQWEKPQIKESKRTFLHNIVNEEGDKGKLECFVNFCEETIFEMQHANSISAEEQALMLAQYASNRAAGEGQRTFAFLYILFYFFFSILGNFNYFFYASHLLDVAVCFKTLGTILQSVTHNGKQLVLTVMLLSIIVYLYTVIAFNFFRKFYVQEEDDEVDYKCHDMLSCYVFHLYVGVRAGGGIGDEINPPDGDPYEVYRIFFDITFFFFVIVILLAIIQGLIIDAFGELRDQLEQVKSDLESSCFICGMNKEYFDKQPHGFETHVQNEHNFANYLFFLMHLINKPDTEYTGQESYVWQLYQQRCWDFFPVGECFRSQYESELLK

>Helro_162842_162843_162844_162846

LHIIVVTITKNLLTMTQSLIGRNMLIEQVLRLFHFLIKFGYYSPDDIKNLLKPLINLLNGKNDKPFLPDAEKGSYSKESHKTVLAFKTKDRFEANINAEAIVNAKFQALEVLDLLFTFQFNTRLECFIAKFKTAELTTINKKSKFQSALCPLLEESFDAFDQSKSSIAIQKKAVKELKNMFDFTAYFDSEQITSILMDLTYYKYDKMVNKALALMNKFYSAKTKLFKSAVQAMVLITSDSCHVHREIARTMPILRRFSKAKLNEDQVKSLGAVLDRYTEYAAFFFVILQLQSSKMTIIPGRLKKMMSESKRLEQEELDLRCLQLLRTIIHNEERKLPEDWEENPSDNKKQMAVIRNLQVELNDMGAVITALPHLAKPSDRMAKEVLAFLCIMLFNANEKIQSSLLQYFLHTREESFFAAIRNRMHLSALAIKEKRSLLAQHRARAKEVMAQSKSMLTTMAIEKKTLQEIQAVDLALKKDRIATRMNGVADNLYNEQLPDKLTAMGDENLLVGCGADEIEKMFLQKEKKQLKSKQDRKKFSNNLKKDGKLKQMSSSLSPQSDLYKDTVIVNIDDNHQEDDQQDLDNIQNDGMDDEDEDDLSNLDQKNGGCIGVVLRVLARMCDGQHAGLQGNQLNRSVMLNSKVVDYINFILRAAEFSGCSLNKILSLKMSIGNLVLAMIEENTPESLIVAKVVAFYIFILFLVLLNLALTVLKEVKDTMDKEAVYRCMTQCYESCLSDRTKLDSLKGNLSMKAIQAAKDLPKFAKRQDDDSRTPELKEKLDEVGFVYYLILARIYDIDPKLGNNVNLTEMQQESFNHYRKNTMSIEILKDSNLQKINFRNVLRNEIKEKLTWNVDRSSPSNKIRDLMKWSKDILIDIYYQRKILSNPLATFLTKNWLLWNRLVIILSLILNIFMLATWNASMSLADLPGSKNLTDLPDELYKVHVRNHPHPHCSLPTSAPAKRQPKSCLKLEEYSTVIYSLGGVHCFFSFLVLLTYFLSNHPTLPSFSAIKQKFAKCGWKKKKKKGTDSEEEEEKEHESKLEVKFLSFTTLYYLVFLGMSVAGIVFQEGKGSSWIKSLCMKFCVCVMYGYFFAFHLLNIVNNNQLLRGVIQAVTLNGKSLLWVAVLGLVVFYLYGLVSFALMRTSFDPENELYCSTLWQCTVSVVHYGLVGDIFSKVVSHPAENTFYRFGFLVLFHMSFFIFITTIGLNIIFGIIVDTFSELRNLKWTAERDMIDTCFICSRSSYDFEHHGQDLTDAETVTACKAVKQN

>Helro_176697_176698_176700

EKPKHVPCPQAIQFQICIQNRYKLNKKYRKLLQTSADEVESLELKTLKAQAKFAADAENDDNVAEQKRQHGKRLKHMFTDKFIHISTTTTSRRDKNNMLVHLLAYNGKHAQIKILPRYKVKTEGESVQIFDQIIFESVKSPGQFFHVSAPWKIDNFSVGSELNLGVQPAGFTVVKCYRHSDEQKEYLRVHEYMIAMAFLIALQGGSVIRLFHKELEAFVVAEGLFDDEITEDVHLRIREVDQLVPKSLYPTTSGNTFWQVETETSLLNGDVIRWEQQIRFRHMTTRQYLCITTDRRVTLTFDSKDPRTVFRVHPVIKEVDEIIFDSYARIEHVITGYWLHAMRDEEYVRRQLRQTENESGESLKSLRWDAASLRKISASGEKVYHDAYTLQVVEPANLSAFNYAAGMVPFLLNHILDRNQGKILNAKKTFKITQALEELKNFMIVNGVAMKKRQKLMRNLRLIDLLVRALQFPLQGAQDQTHLTKIFKGAYDVLYTYMIGNSRKNALYFAKYIDFFQTQITVKVGDIGLNVAKMIAELIRDNRKIVDRISQEQIELFVGLLKINKNYRYLDLLNVLCVCNGVAITNNQTYITEHWLRRDKVTEMSIEVLKDDCLQKINFRVKNKNVLREEVKEKMTWNVDRSSPSNKIRDMMKWSKDILKDIYYQRKILSNPFATFFTKQWVLWNQIVILLSVAINILMLVTWNAHTSLEDVEITKNATSVPNELYEPLPRIELEQYDVIILALGGVHNFFCGLVLLFLCMSIMGTIFRGYFFAFHLLNIVNNNQLLRGVIQAVTQNGKSLLWVAVLGFVVFYLYGLVSFALLRSSFDPDNDLYCATLWQCTVTVIRYGLVGQIFDKLNSHPAENTFTKFGLLVIFHLSFFIFITTIGLNIIFGIIVDTFSELRDLKGFDHHINYEHDIWAYIFFFIHLHDTKQCDYTSLELYIYRLLSQEKFDFFPLNRALSLSVMDEGSTDSKIDDLLKYVVTMVDRQREEDAKTKREEERQKQLRWREENRSALLQIGRSQTDPGNEGGQAFGGTLPRRRRKQLEKLQHYSHDYPLYDAKEATSDDHLNHDDAIDRHSDGGYRYGSGGRGGGDDEGGGDSRSISPSGRFRTREPSTWLPEESVDSLDIDYNNESEHSLHHHKHHHHEGMEKGVEMDDRLASSQVGVTTFESRASSIIAPNDYQEDYDGQALLPLSLEKMTSRRSDTAASCRSDLDRMSPIDAVKRKERKFGKSGGPSGSGSGGRKSSVSSRSAGSSINRDIGNVDGDDEAAILDDPFEVRSERGGGRRGGGGGGRGGEVDASHTKRVGNNVEIINLSNLNPRLEQVRRLHTAFKIEKAFESLQTSIM

>Helro_194357_194356

HQNLDLFLVPGLLQAETCRAIYQDNVQLCNEVVTNNLYGSMNLVEMMKSERERMDPSSPLTYHIHLVQLLAACTEGKNVYTEIKCHSFLPLDDIVKVVTNEDCIAEVKSAYINFLNHCYIDTEVEMKEIYNSTHMWSLFDDFLLDMATSQQPMFIKLLQGAYRVANCKWLSARNRLLAIPPELEFQLIAFFNRTHNLMKHSRAWISSAGKYRSEVAPPSQDCRNIIEGLQDIVALLEDQLKPLIQSELSVLVDVLHQPEALFLPGTEAQRKCQSGGFISKLIQHAGKLLEEKEEKLCLKLLQTLKDMMTLDSEENKCDQLRSKLLQCYYGKQQGHHKSSKFNNSAMMTSSPSLSSSSSSSSSSSLMGQGQDYDKGSMMISRADMTLHDVQCYLDSQGTTDLVIDLIIKNYSNKSFQEAIELGIALLDGGNGVIQKSFFERLTYEKNSEKFFKVLYDRIRDAHTEIKANLSVTAADSITTGKGGDVPEEDSNKRKAKLIPGKFTSELQDQLEDAATVISKAYTHLQHIRHLEKGSDGGGGSSGGAASGGGGVGGGLLGGAGSRPQAATASTSSAVADDLQDESAGGLGGAGDEKKKLPLEVALMQPILRFLQLLCENHNLELQNFLRDQECFKRLIRENNEDTDDEDFLDCICGSTSGGLGLLGLYINKTNVGLVNQTLQTLTEYCQGPCHENQDNASKLLLAIMESSRENEYAERILYSMNPKQLVDVIHLAYEQFESPDDLLNKKTGSVDEGNDEDFKSRSREVGHNIYILAHQLSKHNKELAEMLRPTEPTPPTTVITSPLTSSSASMATAQTEMCDSALDYYSSHTAQIEIVRPDRAIEQIVFPIPEICEYLTSETKHKIYLTAERDDQGSKVMDFFERFDDLFSEMQWQKKLRANFLLFWMSSHMTQWGKASFYLAILINVLVAVYYPFTSTPKDIVYREETLLNVIRSVTKNGRSILLTAVLAVILIYLFSIVGFTFFRDDFLIEIDPLEKGKTTLNTTLSRRSLNDDDYCLPGSTDCNLIANNKSASNNYTNNKNIITNDGGDNNNEDDDDDDDDDDDGKERHCDSLIMCIVTTLNEGLRNGGGIGDVLRKPSSNEPLFFPRVIYDLLFFFIVIIITLNLIFGVIIDTFADLRSEKQQKEEILKNSCFICGLERAAFDNKTVSFEEHCKQEHNMWHYLYFVILVKVKDPTEFTGPESYVHAMIKEKNLDWFPRMRAMSLSTNENETEQNDITNLKIQLVKTHGIIAQLSNQLAELKEQMNEQRKQKHRPGIISGSTGIPQPL

>Helro_123029_83805

LYNYPQLVTASLKLIFRHFSQKQETISAIKQVQLLITYSDSKNYKQIKCDLEELRLLVEKSELWVTKKKSTNKVLCNNSSIGHHQHPGGHEGQGSSQGHTAPRSSQGQRPVQSRSSRAYNQRLLKNLGVHEAMIELLQIQYDKKNDRSMNEIIQLVHIFIQNFCYRNPANQNLIHQNFNLFLSPGILECETACFIYKDNANLCANLSQSTVQHYITCIEKYGRKSEYLKFLQIIVRSETYVYHRKAQDMIMTGLMDSSEEVLHFYTDSSSIQSLVENMKSQIGPLDPRSSLAYHVDLVNLLACCTEGKNVFTEIRCHSLLSLQCLLDKQGAIKLVTDLIMHNYSTHTFFETVQLGVALLQGGNESCQNSFYAYIENRNCSGKFLNVLQERMKEAEMETKTKVSLITNNIVSGKSSIGFYSISHDNDGINLSQDLATTTTANSSNNNNINSNNRLSFTERELVAIRQSFDECKLVDSMKVMMTILKFLQLLCENHNSKLQNFLRTQPNNKVTCNLVGQTVNFLDSLCGGTAVSLGLLGVFVNRSNVSLVRQTIRTIVKFCHGPCFENQYEVAMRESNLLDTASTLLTDEIQPLAETDFMAVLKLKNSTCNLLLSLIESRHDDVIENCILNYFAPAKLIETIKKLYDLSNAGSSSSTTNSSSGFDSRNSSNSSSSRGSPTTSSSSSPSSPSSSSSSSSSAMEAIEYYDSWTGSIEVVKSDRSMEKVIFPIPDICGYLTSSTKHTVQVTVEKDERGSKVPEFFKRHEMMMKEMIWQKKLREKWLLSWFSRKLTFWKMTSFYLTVLLNVLVAFCFPFSDYNFDIDYRVSYAIWSLIPLLLVQSAFRKNFKPLLLVAVIVVRMVLSLGVINTFKTFGILNIANSLACMVSSLGNNGVLLKRRDEMLKDASFIHHSIHLSFGLAGICLHEFFYSLLLLDIVYREETLMNVIRSVTKNGRSILLTAILAVILIYLFSIVGFVFFRNDFLSDVDTTTTTATTTTTTTAIATTTTTTTTSTTTSTTTSTTITATTSVTSALSGAYVHCDSLLMCIVTVLNEGLRNGGGIGDVLRKPSYQEPLFYARIIYDLLFFFIIIIITLNLIFGVIIDTFADLRSEKQQKEEVLKNSCFICGLERQSFDNKVTSFEDHCAEEHNMWNYFYFIVHLKVKNPLEHCGIERYVHELVKEKNLDWVPHLRAMSLSEGEDEEKMNIDELESIRNQIVDINSLTAQMANQIQELKEQMI

>Hsapiens_IP3Rt1

SERDRMDENSPLMYHIHLVELLAVCTEGKNVYTEIKCNSLLPLDDIVRVVTHEDCIPEVKIAYINFLNHCYVDTEVEMKEIYTSNHMWKLFENFLVDICRACNNTSDRKHADSILEKYVTEIVMSIVTTFFSSPFSDQSTTLQTRQPVFVQLLQGVFRVYHCNWLMPSQKASVESCIRVLSDVAKSRAIAIPVDLDSQVNNLFLKSHSIVQKTAMNWRLSARNAARRDSVLAASRDYRNIIERLQDIVSALEDRLRPLVQAELSVLVDVLHRPELLFPENTDARRKCESGGFICKLIKHTKQLLEENEEKLCIKVLQTLREMMTKDRGYGEKGEALRQVLVNRYYGNVRPSGRRESLTSFGNGPLSAGGPGKPGGGGGGSGSSSMSRGEMSLAEVQCHLDKEGASNLVIDLIMNASSDRVFHESILLAIALLEGGNTTIQHSFFCRLTEDKKSEKFFKVFYDRMKVAQQEIKATVTVNTSDLGNKKKDDEVDRDAPSRKKAKEPTTQITEEVRDQLLEASAATRKAFTTFRREADPDDHYQPGEGTQATADKAKDDLEMSAVITIMQPILRFLQLLCENHNRDLQNFLRCQNNKTNYNLVCETLQFLDCICGSTTGGLGLLGLYINEKNVALINQTLESLTEYCQGPCHENQNCIATHESNGIDIITALILNDINPLGKKRMDLVLELKNNASKLLLAIMESRHDSENAERILYNMRPKELVEVIKKAYMQGEVEFEDGENGEDGAASPRNVGHNIYILAHQLARHNKELQSMLKPGGQVDGDEALEFYAKHTAQIEIVRLDRTMEQIVFPVPSICEFLTKESKLRIYYTTERDEQGSKINDFFLRSEDLFNEMNWQKKLRAQPVLYWCARNMSFWSSISFNLAVLMNLLVAFFYPFKGVRGGTLEPHWSGLLWTAMLISLAIVIALPKPHGIRALIASTILRLIFSVGLQPTLFLLGAFNVCNKIIFLMSFVGNCGTFTRGYRAMVLDVEFLYHLLYLVICAMGLFVHEFFYSLLLFDLVYREETLLNVIKSVTRNGRSIILTAVLALILVYLFSIVGYLFFKDDFILEVDRLPNETAVPETGESLASEFLFSDVCRVESGENCSSPAPREELVPAEETEQDKEHTCETLLMCIVTVLSHGLRSGGGVGDVLRKPSKEEPLFAARVIYDLLFFFMVIIIVLNLIFGVIIDTFADLRSEKQKKEEILKTTCFICGLERDKFDNKTVTFEEHIKEEHNMWHYLCFIVLVKVKDSTEYTGPESYVAEMIKERNLDWFPRMRAMSLVSSDSEGEQNELRNLQEKLESTMKLVTNLSGQLSELKDQMTEQRKQKQRIGLLGHPPHMNVNPQQPA

>Hsap_IP3Rt3

PLMYHISLVDLLAACAEGKNVYTEIKCTSLLPLEDVVSVVTHEDCITEVKMAYVNFVNHCYVDTEVEMKEIYTSNHIWTLFENFTLDMARVCSKREKRVADPTLEKYVLSVVLDTINAFFSSPFSENSTSLQTHQTIVVQLLQSTTRLLECPWLQQQHKGSVEACIRTLAMVAKGRAILLPMDLDAHISSMLSSGASCAAAAQRNASSYKATTRAFPRVTPTANQWDYKNIIEKLQDIITALEERLKPLVQAELSVLVDVLHWPELLFLEGSEAYQRCESGGFLSKLIQHTKDLMESEEKLCIKVLRTLQQMLLKKTKYGDRGNQLRKMLLQNYLQNRKSTSRGDLPDPIGTGLDPDWSAIAATQCRLDKEGATKLVCDLITSTKNEKIFQESIGLAIHLLDGGNTEIQKSFHNLMMSDKKSERFFKVLHDRMKRAQQETKSTVAVNMNDLGSQPHEDREPVDPTTKGRVASFSIPGSSSRYSLGPSLRRGHEVSERVQSSEMGTSVLIMQPILRFLQLLCENHNRDLQNFLRCQNNKTNYNLVCETLQFLDIMCGSTTGGLGLLGLYINEDNVGLVIQTLETLTEYCQGPCHENQTCIVTHESNGIDIITALILNDISPLCKYRMDLVLQLKDNASKLLLALMESRHDSENAERILISLRPQELVDVIKKAYLQEEERENSEVSPREVGHNIYILALQLSRHNKQLQHLLKPVKRIQEEEAEGISSMLSLNNKQLSQMLKSSAPAQEEEEDPLAYYENHTSQIEIVRQDRSMEQIVFPVPGICQFLTEETKHRLFTTTEQDEQGSKVSDFFDQSSFLHNEMEWQRKLRSMPLIYWFSRRMTLWGSISFNLAVFINIIIAFFYPYMEGASTGVLDSPLISLLFWILICFSIAALFTKRYSIRPLIVALILRSIYYLGIGPTLNILGALNLTNKIVFVVSFVGNRGTFIRGYKAMVMDMEFLYHVGYILTSVLGLFAHELFYSILLFDLIYREETLFNVIKSVTRNGRSILLTALLALILVYLFSIVGFLFLKDDFILEVDRLPNNHSTASPLGMPHGAAAFVDTCSGDKMDCVSGLSVPEVLEEDRELDSTERACDTLLMCIVTVMNHGLRNGGGVGDILRKPSKDESLFPARVVYDLLFFFIVIIIVLNLIFGVIIDTFADLRSEKQKKEEILKTTCFICGLERDKFDNKTVSFEEHIKLEHNMWNYLYFIVLVRVKNKTDYTGPESYVAQMIKNKNLDWFPRMRAMSLVSNEGEGEQNEIRILQDKLNSTMKLVSHLTAQLNELKEQMTEQRKRRQRLGFVDVQNCISR

>Hsap_IP3Rt2

SERDRGDESGPLAYHITLVELLAACTEGKNVYTEIKCNSLLPLDDIVRVVTHDDCIPEVKIAYVNFVNHCYVDTEVEMKEIYTSNHIWKLFENFLVDMARVCNTTTDRKHADIFLEKCVTESIMNIVSGFFNSPFSDNSTSLQTHQPVFIQLLQSAFRIYNCTWPNPAQKASVESCIRTLAEVAKNRGIAIPVDLDSQVNTLFMKSHSNMVQRAAMGWRLSARSGPRFKEALGGPAWDYRNIIEKLQDVVASLEHQFSPMMQAEFSVLVDVLYSPELLFPEGSDARIRCGAFMSKLINHTKKLMEKEEKLCIKILQTLREMLEKKDSFVEEGNTLRKILLNRYFKGDYSIGVNGHLSGAYSKTAQVGGSFSGQDSDKMGISMSDIQCLLDKEGASELVIDVIVNTKNDRIFSEGIFLGIALLEGGNTQTQYSFYQQLHEQKKSEKFFKVLYDRMKAAQKEIRSTVTVNTIDLGNKKRDDDNELMTSGPRMRVRDSTLHLKEGMKGQLTEASSATSKAYCVYRREMDPEIDIMCTGPEAGNTEEKSAEEVTMSPAIAIMQPILRFLQLLCENHNRELQNFLRNQNNKTNYNLVCETLQFLDCICGSTTGGLGLLGLYINEKNVALVNQNLESLTEYCQGPCHENQTCIATHESNGIDIIIALILNDINPLGKYRMDLVLQLKNNASKLLLAIMESRHDSENAERILFNMRPRELVDVMKNAYNQGLECDHGDDEGGDDGVSPKDVGHNIYILAHQLARHNKLLQQMLKPGSDPDEGDEALKYYANHTAQIEIVRHDRTMEQIVFPVPNICEYLTRESKCRVFNTTERDEQGSKVNDFFQQTEDLYNEMKWQKKIRNNPALFWFSRHISLWGSISFNLAVFINLAVALFYPFGDDGDEGTLSPLFSVLLWIAVAICTSMLFFFSKPVGIRPFLVSIMLRSIYTIGLGPTLILLGAANLCNKIVFLVSFVGNRGTFTRGYRAVILDMAFLYHVAYVLVCMLGLFVHEFFYSFLLFDLVYREETLLNVIKSVTRNGRSIILTAVLALILVYLFSIIGFLFLKDDFTMEVDRLKNRTPVTGSHQVPTMTLTTMMEACAKENCSPTIPASNTADEEYEDGIERTCDTLLMCIVTVLNQGLRNGGGVGDVLRRPSKDEPLFAARVVYDLLFYFIVIIIVLNLIFGVIIDTFADLRSEKQKKEEILKTTCFICGLERDKFDNKTVSFEEHIKSEHNMWHYLYFIVLVKVKDPTEYTGPESYVAQMIVEKNLDWFPRMRAMSLVSNEGDSEQNEIRSLQEKLESTMSLVKQLSGQLAELKEQMTEQRKNKQRLGFLGSNTPHVNHHMPPH

>Mmus_IP3Rt2

SERARGDESGPLAYHITLVELLAACTEGKNVYTEIKCNSLLPLDDIVRVVTHDDCIPEVKIAYVNFVNHCYVDTEVEMKEIYTSNHIWKLFENFLVDMARVCNTTTDRKHADTFLERCVTESVMNIVSGFFNSPFSDNSTSLQTHQPVFIQLLQSAFRIYNCTWPNPAQKASVESCIRALAEVAKNRGIAIPVDLDSQVNTLFMKNHSSTVQRAAMGWRLSARSGPRFKEALGGPAWDYRNIIEKLQDVVASLEQQFSPMMQAEFSVLVDVLYSPELLFPEGSDARIRCGAFMSKLINHTKKLMEKEEKLCIKILQTLREMLEKKDSFMEEGSTLRRILLNRYFKGDHSISVNGPLSGAYAKTAQVGGSFSGQDSDKKGISMSDIQCLLDKEGASELVIDVIVNTKNDRIFSEGILLGIALLEGGNTQTQYSFYQQLHEQKKSEKFFKVLYDRMKAAQKEIRSTVTVNTIDLGSKKREEDSDVMALGPRMRVRDSSLHLREGMKGQLTEASSATSKAYCVYRREMDPEIDTMCPGQEAGSAEEKSAEEVTMSPAITIMRPILRFLQLLCENHNRELQNFLRNQNNKTNYNLVCETLQFLDCICGSTTGGLGLLGLYINERNVALVNQTLESLTEYCQGPCHENQTCIATHESNGIDIIIALILNDINPLGKYRMDLVLQLKNNASKLLLAIMESRHDSENAERILFNMRPRELVDVMKNAYNQGLECDHGDEEGGDDGVSPKDVGHNIYILAHQLARHNKLLQQMLKPGSDPEEGDEALKYYANHTAQIEIVRHDRTMEQIVFPVPNICEFLTRESKYRVFNTTERDEQGSKVNDFFQQTEDLYNEMKWQKKIRNNPALFWFSRHISLWGSISFNLAVFINLAVALFYPFGDDGDEGTLSPMFSVLLWVAVAICTSMLFFFSKPVGIRPFLVSVMLRSIYTIGLGPTLILLGAANLCNKIVFLVSFVGNRGTFTRGYRAVILDMAFLYHVAYVLVCMLGLFVHEFFYSFLLFDLVYREETLLNVIKSVTRNGRSIILTAVLALILVYLFSIIGFLFLKDDFTMEVDRLKNRTPVTGNHGVPTMTLSSMMETCQKENCSPTIPSSNTAGEEGEDGIERTCDTLLMCIVTVLNQGLRNGGGVGDVLRRPSKDEPLFAARVVYDLLFFFIVIIIVLNLIFGVIIDTFADLRSEKQKKEEILKTTCFICGLERDKFDNKTVSFEEHIKSEHNMWHYLYFIVLVKVKDPTEYTGPESYVAQMITEKNLDWFPRMRAMSLVSNEGDSEQNEIRNLQEKLESTMSLVKQLSGQLAELKEQMTEQRKNKQRLGFLGSNTPHVNHHMPPH

>Mmus_IP3Rt1

QSTTLQTRQPVFVQLLQGVFRVYHCNWLMPSQKASVESCIRVLSDVAKSRAIAIPVDLDSQVNNLFLKSHNIVQKTALNWRLSARNAARRDSVLAASRDYRNIIERLQDIVSALEDRLRPLVQAELSVLVDVLHRPELLFPENTDARRKCESGGFICKLIKHTKQLLEENEEKLCIKVLQTLREMMTKDRGYGEKQISIDESENAELPQAPEAENSTEQELEPSPPLRQLEDHKRGEALRQILVNRYYGNIRPSGRRESLTSFGNGPLSPGGPSKPGGGGGGPGSSSTSRGEMSLAEVQCHLDKEGASNLVIDLIMNASSDRVFHESILLAIALLEGGNTTIQHSFFCRLTEDKKSEKFFKVFYDRMKVAQQEIKATVTVNTSDLGNKKKDDEVDRDAPSRKKAKEPTTQITEEVRDQLLEASAATRKAFTTFRREADPDDHYQSGEGTQATTDKAKDDLEMSAVITIMQPILRFLQLLCENHNRDLQNFLRCQNNKTNYNLVCETLQFLDCICGSTTGGLGLLGLYINEKNVALINQTLESLTEYCQGPCHENQNCIATHESNGIDIITALILNDINPLGKKRMDLVLELKNNASKLLLAIMESRHDSENAERILYNMRPKELVEVIKKAYMQGEVEFEDGENGEDGAASPRNVGHNIYILAHQLARHNKELQTMLKPGGQVDGDEALEFYAKHTAQIEIVRLDRTMEQIVFPVPSICEFLTKESKLRIYYTTERDEQGSKINDFFLRSEDLFNEMNWQKKLRAQPVLYWCARNMSFWSSISFNLAVLMNLLVAFFYPFKGVRGGTLEPHWSGLLWTAMLISLAIVIALPKPHGIRALIASTILRLIFSVGLQPTLFLLGAFNVCNKIIFLMSFVGNCGTFTRGYRAMVLDVEFLYHLLYLLICAMGLFVHEFFYSLLLFDLVYREETLLNVIKSVTRNGRSIILTAVLALILVYLFSIVGYLFFKDDFILEVDRLPNETAVPETGESLANDFLYSDVCRVETGENCTSPAPKEELLPAEETEQDKEHTCETLLMCIVTVLSHGLRSGGGVGDVLRKPSKEEPLFAARVIYDLLFFFMVIIIVLNLIFGVIIDTFADLRSEKQKKEEILKTTCFICGLERDKFDNKTVTFEEHIKEEHNMWHYLCFIVLVKVKDSTEYTGPESYVAEMIRERNLDWFPRMRAMSLVSSDSEGEQNELRNLQEKLESTMKLVTNLSGQLSELKDQMTEQRKQKQRIGLLGHPPHMNVNPQQPA

>Mmus_IP3Rt3

PLMYHISLVDLLAACAEGKNVYTEIKCTSLLPLEDVVTVVTHEDCITEVKMAYVNFVNHCYVDTEVEMKEIYTSNHIWTLFENFTLDMALVCNKREKRLSDPTLEKYVLTVVLDTISAFFSSPFSENSTSLQTHQTIVVQLLQSTTRLLECPWLQQQHKGSVEACVRTLAMVAKSRAILLPMDLDAHMSALLSSGGSCSAAAQRSAANYKTATRTFPRVIPTANQWDYKNIIEKLQDIIMALEERLKPLVQAELSVLVDMLHWPELLFPEGSEAYQRCESGGFLSKLIRHTKGLMESEEKLCVKVLRTLQQMLLKKSKFGDRGNQLRKMLLQNYLQNRKSGARGELTDPTGSGLDQDWSAIAATQCRLDKEGATKLVCDLITSTKNEKIFQESIGLAIRLLDGGNTEIQKSFYNLMTSDKKSERFFKVLHDRMKRAQQETKSTVAVNMSDLGSQPREDREPADPATKGRVSSFSMPSSSRYLLGLGLHRGHDMSERAQNNEMGTSVLIMRPILRFLQLLCENHNRDLQNFLRCQNNKTNYNLVCETLQFLDIMCGSTTGGLGLLGLYINEDNVGLVIQTLETLTEYCQGPCHENQTCIVTHESNGIDIITALILNDISPLCKYRMDLVLQLKDNASKLLLALMESRHDSENAERILISLRPQELVDVIKKAYLQEEERENSEVSPREVGHNIYILALQLSRHNKQLQHLLKPVRRIQEEEAEGISSMLSLNNKQLSQMLKSSAPAQEEEEDPLAYYENHTSQIEIVRQDRSMEQIVFPVPAICQFLTEETKHRLFTTTEQDEQGSKVSDFFDQSSFLHNEMEWQRRLRSMPLIYWFSRRMTLWGSISFNLAVFINIIIAFFYPYVEGASTGVLGSPLISLLFWILICFSIAALFTKRYSVRPLIVALILRSIYYLGIGPTLNILGALNLTNKIVFVVSFVGNRGTFIRGYKAMVMDMEFLYHVGYILTSVLGLFAHELFYSILLFDLIYREETLFNVIKSVTRNGRSILLTALLALILVYLFSIVGFLFLKDDFILEVDRLPGNHSRASPLGMPHGAATFMGTCSGDKMDCVSEVSVPEILEEDEEPDSTERACDTLLMCIVTVMNHGLRNGGGVGDILRKPSKDESLFPARVVYDLLFFFIVIIIVLNLIFGVIIDTFADLRSEKQKKEEILKTTCFICGLERDKFDNKTVSFEEHIKLEHNMWNYLYFIVLVRVKNKTDYTGPESYVAQMIKNKNLDWFPRMRAMSLVSGEGEGEQNEIRILQEKLGSTMKLVSHLTSQLNELKEQMTEQRKRRQRLGFVDVQNCMSR

>Mmus_RyaR1

RMIDDLSKAGEQEEEEEEVEEKKPDPLHQLVLHFSRTALTEKSKLDEDYLYMAYADIMAKSCHLEEGGENGEEGGEEEEVEVSFEEKEMEKQRLLYQQSRLHNRGAAEMVLQMISACKGETGAMVSSTLKLGISILNGGNAEVQQKMLDYLKDKKEVGFFQSIQALMQTCSVLDLNAFERQNKAEGLGMVNEDGTVINRQNGEKVMADDEFTQDLFRFLQLLCEGHNNDFQNYLRTQTGNTTTINIIICTVDYLLRLQESISDFYWYYSGKDVIEEQGKRNFSKAMSVAKQVFNSLTEYIQGPCTGNQQSLAHSRLWDAVVGFLHVFAHMMMKLAQDSSQIELLKELLDLQKDMVVMLLSLLEGNVVNGMIARQMVDMLVESSSNVEMILKFFDMFLKLKDIVGSEAFQDYVTDPRGLISKKDFQKAMDSQKQFTGPEIQFLLSCSEADENEMINCEEFANRFQEPARDIGFNVAVLLTNLSEHVPHDPRLRNFLELAESILEYFRPYLGRIEIMGASRRIERIYFEISETNRAQWEMPQVKESKRQFIFDVVNEGGESEKMEMFVSFCEDTIFEMQIAAQISEPEGEPEEDEDEGAEEAEEGAAGSDGSGSAAAAGVWVWLAATAGRTLRGLSYRSLRRRVRRLRRLTAREAATAVAALLWALVTRAGGAGAGAAAGALRLLWGSLFGGGLVDSAKKVTVTELLAGMPDPTGDEVHGQQPSGAGSDAEGEGEGEGEGDAADGAGDEEAAADQAGTGGADGAVAVADGSPFRPEGAGGLGDMGDTTPVEPPTPEGSPILKRKLGVDGEEEEPPPEPEPEPEPEPEKADTENGEKEVPEPPPEPPKKTPPPPPPKKEEAGGAGLEEFWGELEVQRVKFLNYLSRNFYTLRFLALFLAFAINFILLFYKVSDSPPGEDDIEGSGAGDMSGAGSGDGSGWGSRAGEEVEGDEDENMVYYFLEESTGYMEPALRCLSLLHTLVAFLCIIGYNCLKVPLVIFKREKELARKLEFDGLYITEQPEDDDVKGQWDRLVLNTPSFPSNYWDKFVKRKVLDKHGDIFGRERIAELLGMDLASLEITAHNERKPDPPPGLLTWIMSIDVKYQIWKFGVIFTDNSFLYLGWYMVMSLLGHYNNFFFAAHLLDIAMGVKTLRTILSSVTHNGKQLVMTVGLLAVVVYLYTVVAFNFFRKFYNKSEDEDEPDMKCDDMMTCYLFHMYVGVRAGGGIGDEIEDPAGDEYELYRVVFDITFFFFVIVILLAIIQGLIIDAFGELRDQQEQVKEDMETKCFICGIGSDYFDTTPHGFETHTLEEHNLANYMFFLMYLINKDETEHTGQESYVWKMYQERCWDFFPAGDCFRKQYEDQLS

>Mmus_RyaR2

TALTEKCKLEEDFLYMAYADIMAKSCHDEEDDDGEEEVKSFEEKEMEKQKLLYQQARLHDRGAAEMVLQTISASKGETGPMVAATLKLGIAILNGGNSTVQQKMLDYLKEKKDVGFFQSLAGLMQSCSVLDLNAFERQNKAEGLGMVTEEGSGEKVLQDDEFTCDLFRFLQLLCEGHNSDFQNYLRTQTGNNTTVNIIISTVDYLLRVQESISDFYWYYSGKDIIDEQGQRNFSKAIQVAKQVFNTLTEYIQGPCTGNQQSLAHSRLWDAVVGFLHVFAHMQMKLSQDSSQIELLKELMDLQKDMVVMLLSMLEGNVVNGTIGKQMVDMLVESSNNVEMILKFFDMFLKLKDLTSSDTFKEYDPDGKGVISKRDFHKAMESHKHYTQSETEFLLSCAETDENETLDYEEFVKRFHEPAKDIGFNVAVLLTNLSEHMPNDTRLQTFLELAESVLNYFQPFLGRIEIMGSAKRIERVYFEISESSRTQWEKPQVKESKRQFIFDVVNEGGEKEKMELFVNFCEDTIFEMQLAAQISESDLNERLANKEESEKERPEEQAPRMGFFSLLTIQSALFALRYNVLTLVRMLSLKSLKKQMKRMKKMTVKDMVLAFFSSYWSVFVTLLHFVASVCRGFFRIVSSLLLGGSLVEGAKKIKVAELLANMPDPTQDEVRGDEEEGERKPLESALPSEDLTDLKELTEESDLLSDIFGLDLKREGGQYKLIPHNPNAGLSDLMTNPVPVPEVQEKFQEQKAKEEKEEKEETKSEPEKAEGEDGEKEEKAKDEKSKQKLRQLHTHRYGEPEVPESAFWKKIIAYQQKLLNYFARNFYNMRMLALFVAFAINFILLFYKVSTSSVVEGKELPTRTSSDTAKVTNSLDSSPHRIIAVHYVLEESSGYMEPTLRILAILHTIISFFCIIGYYCLKVPLVIFKREKEVARKLEFDGLYITEQPSEDDIKGQWDRLVINTQSFPNNYWDKFVKRKVMDKYGEFYGRDRISELLGMDKAALDFSDAREKKKPKKDSSLSAVLNSIDVKYQMWKLGVVFTDNSFLYLAWYMTMSVLGHYNNFFFAAHLLDIAMGFKTLRTILSSVTHNGKQLVLTVGLLAVVVYLYTVVAFNFFRKFYNKSEDGDTPDMKCDDMLTCYMFHMYVGVRAGGGIGDEIEDPAGDEYEIYRIIFDITFFFFVIVILLAIIQGLIIDAFGELRDQQEQVKEDMETKCFICGIGNDYFDTVPHGFETHTLQEHNLANYLFFLMYLINKDETEHTGQESYVWKMYQERCWEFFPAGDCFRKQYEDQLN

>Mmus_RyaR3

EDEEEEETERQPDPLHQIILHFSRNALTERSKLEDDPLYTSYSSMMAKSCQSGEDEEEEEDKEKTFEEKEMEKQKTLYQQARLHERGAAEMVLQMISASKGEMSPMVVETLKLGIAILNGGNAGVQQKMLDYLKEKKDAGFFQSLSGLMQSCSVLDLNAFERQNKAEGLGMVTEEGTLIVRERGEKVLQNDEFTQDLFRFLQLLCEGHNSDFQNFLRTQMGNTTTVNIIISTVDYLLRLQESISDFYWYYSGKDIIDESGQHNFSKALAVTKQIFNSLTEYIQGPCIGNQQSLAHSRLWDAVVGFLHVFANMQMKLSQDSSQIELLKELLDLLQDMVVMLLSLLEGNVVNGTIGKQMVDTLVESSTNVEMILKFFDMFLKLKDLTSSDTFKEYDPDGKGIISRKEFQKAMEGLKQYTQSEIDFLLSCTEADENDMFNYVDFVERFHEPAKDIGFNVAVLLTNLSEHMPNDSRLKSLLDPAESVLNYFEPYLGRIEIMGGAKKIERVYFEISESSRTQWEKPQVKESKRQFIFDVVNEGGEQEKMELFVNFCEDTIFEMQLASQISESDSTDRPEEEEEEDEDSAYSIETEGEEEEKSFESASAFTMACVSVKRNVTKFLKRATLKNLRKQYRNVKKMSAKELVKVFFSFFWMLFVGLFQLLFTIFGGIFQILWNTVFGGGLVEGAKNIRVTKILGDMPDPTQFGIHDDVIETDRAEVTEPGVTTELVHFVKGEAGDTDIMSDLFGIHSKKEGGLKQGPEVGLGDLSEIIGKDEPPTLESTVRKKRKAQAAEMKAVHEAEGKAESEKADMEDREKEDKIKEEGQTDYLWADVTVKKTRRRGQKAEKPEAFMANFFKGLEIYQTKLLHYLARNFYNLRFLALFVAFAINFILLFYKVTEEPLEEETEDVANLWNSFNDDDEEEAMVFFVLQESTGYMAPTLRALAIVHTIISLVCVVGYYCLKVPLVVFKREKEIARKLEFDGLYITEQPSEDDIKGQWDRLVINTPSFPNNYWDKFVKRKVINKYGDLYGAERIAELLGLDKNALDFSPVEEAKAEAASLVSWLSSIDMKYHIWKLGVVFTDNSFLYLAWYTTMSVLGHYNNFFFAAHLLDIAMGFKTLRTILSSVTHNGKQLVLTVGLLAVVVYLYTVVAFNFFRKFYNKSEDDDEPDMKCDDMMTCYLFHMYVGVRAGGGIGDEIEDPAGDPYEMYRIVFDITFFFFVIVILLAIIQGLIIDAFGELRDQQEQVREDMETKCFICGIGNDYFDTTPHGFETHTLQEHNLANYLFFLMYLINKDETEHTGQESYVWKMYQERCWDFFPAGDCFRKQYEDQLG

>Hsap_RyaR1

MIDDLSKAGEQEEEEEEVEEKKPDPLHQLVLHFSRTALTEKSKLDEDYLYMAYADIMAKSCHLEEGGENGEAEEEVEVSFEEKQMEKQRLLYQQARLHTRGAAEMVLQMISACKGETGAMVSSTLKLGISILNGGNAEVQQKMLDYLKDKKEVGFFQSIQALMQTCSVLDLNAFERQNKAEGLGMVNEDGTVINRQNGEKVMADDEFTQDLFRFLQLLCEGHNNDFQNYLRTQTGNTTTINIIICTVDYLLRLQESISDFYWYYSGKDVIEEQGKRNFSKAMSVAKQVFNSLTEYIQGPCTGNQQSLAHSRLWDAVVGFLHVFAHMMMKLAQDSSQIELLKELLDLQKDMVVMLLSLLEGNVVNGMIARQMVDMLVESSSNVEMILKFFDMFLKLKDIVGSEAFQDYVTDPRGLISKKDFQKAMDSQKQFSGPEIQFLLSCSEADENEMINCEEFANRFQEPARDIGFNVAVLLTNLSEHVPHDPRLHNFLELAESILEYFRPYLGRIEIMGASRRIERIYFEISETNRAQWEMPQVKESKRQFIFDVVNEGGEAEKMELFVSFCEDTIFEMQIAAQISEPEGEPETDEDEGAGAAEAGAEGAEEGAAGLEGTAATAAAGATARVVAAAGRALRGLSYRSLRRRVRRLRRLTAREAATAVAALLWAAVTRAGAAGAGAAAGALGLLWGSLFGGGLVEGAKKVTVTELLAGMPDPTSDEVHGEQPAGPGGDADGEGASEGAGDAAEGAGDEEEAVHEAGPGGADGAVAVTDGGPFRPEGAGGLGDMGDTTPAEPPTPEGSPILKRKLGVDGVEEELPPEPEPEPEPELEPEKADAENGEKEEVPEPTPEPPKKQAPPSPPPKKEEAGGEFWGELEVQRVKFLNYLSRNFYTLRFLALFLAFAINFILLFYKVSDSPPGEDDMEGSAAGDVSGAGSGGSSGWGLGAGEEAEGDEDENMVYYFLEESTGYMEPALRCLSLLHTLVAFLCIIGYNCLKVPLVIFKREKELARKLEFDGLYITEQPEDDDVKGQWDRLVLNTPSFPSNYWDKFVKRKVLDKHGDIYGRERIAELLGMDLATLEITAHNERKPNPPPGLLTWLMSIDVKYQIWKFGVIFTDNSFLYLGWYMVMSLLGHYNNFFFAAHLLDIAMGVKTLRTILSSVTHNGKQLVMTVGLLAVVVYLYTVVAFNFFRKFYNKSEDEDEPDMKCDDMMTCYLFHMYVGVRAGGGIGDEIEDPAGDEYELYRVVFDITFFFFVIVILLAIIQGLIIDAFGELRDQQEQVKEDMETKCFICGIGSDYFDTTPHGFETHTLEEHNLANYMFFLMYLINKDETEHTGQESYVWKMYQERCWDFFPAGDCFRKQYEDQLS

>Hsap_RyaR2

RTALTEKCKLEEDFLYMAYADIMAKSCHDEEDDDGEEEVKSFEEKEMEKQKLLYQQARLHDRGAAEMVLQTISASKGETGPMVAATLKLGIAILNGGNSTVQQKMLDYLKEKKDVGFFQSLAGLMQSCSVLDLNAFERQNKAEGLGMVTEEGSGEKVLQDDEFTCDLFRFLQLLCEGHNSDFQNYLRTQTGNNTTVNIIISTVDYLLRVQESISDFYWYYSGKDVIDEQGQRNFSKAIQVAKQVFNTLTEYIQGPCTGNQQSLAHSRLWDAVVGFLHVFAHMQMKLSQDSSQIELLKELMDLQKDMVVMLLSMLEGNVVNGTIGKQMVDMLVESSNNVEMILKFFDMFLKLKDLTSSDTFKEYDPDGKGVISKRDFHKAMESHKHYTQSETEFLLSCAETDENETLDYEEFVKRFHEPAKDIGFNVAVLLTNLSEHMPNDTRLQTFLELAESVLNYFQPFLGRIEIMGSAKRIERVYFEISESSRTQWEKPQVKESKRQFIFDVVNEGGEKEKMELFVNFCEDTIFEMQLAAQISESDLNERSANKEESEKERPEEQGPRMAFFSILTVRSALFALRYNILTLMRMLSLKSLKKQMKKVKKMTVKDMVTAFFSSYWSIFMTLLHFVASVFRGFFRIICSLLLGGSLVEGAKKIKVAELLANMPDPTQDEVRGDGEEGERKPLEAALPSEDLTDLKELTEESDLLSDIFGLDLKREGGQYKLIPHNPNAGLSDLMSNPVPMPEVQEKFQEQKAKEEEKEEKEETKSEPEKAEGEDGEKEEKAKEDKGKQKLRQLHTHRYGEPEVPESAFWKKIIAYQQKLLNYFARNFYNMRMLALFVAFAINFILLFYKVSTSSVVEGKELPTRSSSENAKVTSLDSSSHRIIAVHYVLEESSGYMEPTLRILAILHTVISFFCIIGYYCLKVPLVIFKREKEVARKLEFDGLYITEQPSEDDIKGQWDRLVINTQSFPNNYWDKFVKRKVMDKYGEFYGRDRISELLGMDKAALDFSDAREKKKPKKDSSLSAVLNSIDVKYQMWKLGVVFTDNSFLYLAWYMTMSVLGHYNNFFFAAHLLDIAMGFKTLRTILSSVTHNGKQLVLTVGLLAVVVYLYTVVAFNFFRKFYNKSEDGDTPDMKCDDMLTCYMFHMYVGVRAGGGIGDEIEDPAGDEYEIYRIIFDITFFFFVIVILLAIIQGLIIDAFGELRDQQEQVKEDMETKCFICGIGNDYFDTVPHGFETHTLQEHNLANYLFFLMYLINKDETEHTGQESYVWKMYQERCWEFFPAGDCFRKQYEDQLN

>Hsap_RyaR3

KVEEEEEEETEKQPDPLHQIILYFSRNALTERSKLEDDPLYTSYSSMMAKSCQSGEDEEEDEDKEKTFEEKEMEKQKTLYQQARLHERGAAEMVLQMISASKGEMSPMVVETLKLGIAILNGGNAGVQQKMLDYLKEKKDAGFFQSLSGLMQSCSVLDLNAFERQNKAEGLGMVTEEGTLIVRERGEKVLQNDEFTRDLFRFLQLLCEGHNSDFQNFLRTQMGNTTTVNVIISTVDYLLRLQESISDFYWYYSGKDIIDESGQHNFSKALAVTKQIFNSLTEYIQGPCIGNQQSLAHSRLWDAVVGFLHVFANMQMKLSQDSSQIELLKELLDLLQDMVVMLLSLLEGNVVNGTIGKQMVDTLVESSTNVEMILKFFDMFLKLKDLTSSDTFKEYDPDGKGIISKKEFQKAMEGQKQYTQSEIDFLLSCAEADENDMFNYVDFVDRFHEPAKDIGFNVAVLLTNLSEHMPNDSRLKCLLDPAESVLNYFEPYLGRIEIMGGAKKIERVYFEISESSRTQWEKPQVKESKRQFIFDVVNEGGEQEKMELFVNFCEDTIFEMQLASQISESDSADRPEEEEEDEDSSYVLEIAGEEEEDGSLEPASAFAMACASVKRNVTDFLKRATLKNLRKQYRNVKKMTAKELVKVLFSFFWMLFVGLFQLLFTILGGIFQILWSTVFGGGLVEGAKNIRVTKILGDMPDPTQFGIHDDTMEAERAEVMEPGITTELVHFIKGEKGDTDIMSDLFGLHPKKEGSLKHGPEVGLGDLSEIIGKDEPPTLESTVQKKRKAQAAEMKAANEAEGKVESEKADMEDGEKEDKDKEEEQAEYLWTEVTKKKKRRCGQKVEKPEAFTANFFKGLEIYQTKLLHYLARNFYNLRFLALFVAFAINFILLFYKVTEEPLEEETEDVANLWNSFNDEEEEEAMVFFVLQESTGYMAPTLRALAIIHTIISLVCVVGYYCLKVPLVVFKREKEIARKLEFDGLYITEQPSEDDIKGQWDRLVINTPSFPNNYWDKFVKRKVINKYGDLYGAERIAELLGLDKNALDFSPVEETKAEAASLVSWLSSIDMKYHIWKLGVVFTDNSFLYLAWYTTMSVLGHYNNFFFAAHLLDIAMGFKTLRTILSSVTHNGKQLVLTVGLLAVVVYLYTVVAFNFFRKFYNKSEDDDEPDMKCDDMMTCYLFHMYVGVRAGGGIGDEIEDPAGDPYEMYRIVFDITFFFFVIVILLAIIQGLIIDAFGELRDQQEQVREDMETKCFICGIGNDYFDTTPHGFETHTLQEHNLANYLFFLMYLINKDETEHTGQESYVWKMYQERCWDFFPAGDCFRKQYEDQLG

>Dmel_IP3R

LDDIVTIICHPLCMPEVKEAYVDFLNHCYIDTEVEMKEIYASGHMWSLFEKSFLVDINQLITNPAAASNKTLQAYVLNGVTNLLGSFFASPFSDQSAIVQSRQLIFVQLLQAAHRITQCRWLSLGDRFNVENCIRTLTESAKMRSIALPPELEQQVATMSSKTAMLTRQTTKWLLASKQPKYEAQQAASLMRWDRSIIEGLQDIVSLLEDQLKPVVEAELSLLVDILYRSELLFPAGTEARKRCESGGFIRKLIKHTEKLLEEKEERMCVKVLRTLREMMAIDVNYGEKGDALRQTLLLRYFQTKSTPRLPEDEVPLLAAPLMDPAKQNHLVTHGPGAKYLQRAGKTLHEMQNHLDREGASDLVVELVIKSVHSPNIFVEAVELGIALLEGGNPIIQKGMFQKFLSDDLNQAFFKVFFEKMKDAQQEIKSTVTVNTTDIAAKAHEHKQDTNLELDKIARKHGLKSNGVVITEELKRELHNAGLATARAYGNARNIHSGEESSAISVNSPLEDILAEKLEKHKDSRDQRNQLSNKVLVMQPILRFLQLLCENHNPDMQNLLRNQNNKTNNNLVSETLMFLDCICGSTTGGLGLLGLYINEHNVALINQTLEALTEYCQGPCHENQNCIATHESNGLDIITALILNNINPLGENRMDLVLELKNNASKLLLAIMESRGDSENAERILYNMNPKQLVEVACKAYHQEELIDEQDDGDEPDAGSDDDDATVSPREVGHNIYILCHQLAQHNKELAGLLKASEDPQSASFDAKTSQALMYYATHTAQIEIVRNDRTLEQIVFPIPEICEYLTTDTKIKILNTAERDDQGSKVADFFDKAEEMFNEMKWQKKLRSQPLLFWISSYMSLWSNILFNCVVVINMIVAFFYPFDNTVPELSSHISLLFWIITIFSLVIVLALPRESGIRTFIGSVILRFIFLLGPESTLCLLGVVTVTLKSVHIVSIMGNKGTLEKQLIKIITDFQLLYHCIYIAFCFCGLIFHPFFYSLLLFDVVYREETLVNVIRSVTRNGRSIVLTAVLALILVYLFSIIGYMFFKDDFLVSVDFEEQDNAPPPSVPLTLSVPVSGDSCSAPDDLGNCQAAKEVAPPSAGGGEVKERSCDSLVMCIVTTLNQGLRNGGGIGDILRAPSSKEGLFVARVIYDLLFFFIVIIIVLNLIFGVIIDTFADLRSEKQQKEAILKTTCFICSLNRSAFDNKTVSFEEHIKSEHNMWHYLYFIVLVKVKDPTEFTGPESYVYAMVKAGILEWFPRLRAMSLAAVDADGEQIELRSMQAQLLDTQLLIKNLSTQLHELKDHMTEQRKQKQRLGLLNTTANSLLPFQ

>Dmel_RyaR

FEDSEKSKKEGEETDSKPDPLTQLVTTFCRGAMTERSGALQEDLLYMSYAQIAAKSTGKEEEEGGDEEGGEGGEEGEGTSIHEQEMEKQKLLFHQARLSNRGVAEMVLLHISASKGIPSEMVMTTLNLGIAILRGGNIDIQMGMLNHLKEKKDVGFFTSIAGLMNSCSVLDLDAFERNTKAEGLGVGSEGAAGEKNMHDAEFTCALFRFIQLTCEGHNLEWQNYLRTQAGNTTTVNVVICTVDYLLRLQESIMDFYWHYSSKEIIDPAGKANFFKAIEVASQVFNTLTEVIQGPCTLNQQALAHSRLWDAVGGFLFLFSHMQDKLSKHSSQVDLLKELLNLQKDMITMMLSMLEGNVVNGTIGKQMVDTLVESASNVELILKYFDMFLKLADLIESPSFHEVDMKNEGWVTPKDFREKMEQSKNYTPEEMDFLLACCERNHEGKIDYRAFVEHFHEPSKEIGFNLAVLLTNLSEHMPNEPRLARFLETAGSVLNYFEPFLGRIEILGSSKRIERVYFEIKDSNIEQWEKPQIRESKRAFFYSIVTEGGDKEKLEAFVNFCEDAIFEMTHASGLMATDDGGGNVKRDTAYSSYMSEEEEERAARDPIRRTITAVKEGLKFGVHMLSPANIKHQIGVMQTKSIPELIVGFFKIIFYIFYYTGYAHFCVVRYIFGILLNLMRGPAPEQEEEPVVEEETFGRALPPLPLEEPPGTVQAFGLDINKEENGMYKVVVHESPANSSMEEGGESSPEDGAAASGELVEGEPHQEPISIVDLLGGEAAKKAAQERQEAQKAQEAAMASIEAEAKKSSSAPQETPAVHQIDFSQYTHRAVSFLARNFYNLKYVALVLAFSINFMLLFYKVTSFTEEADSSAEEELILGSGSGGGADITGSGFGGSGDGGSGDGEMEDEIPELVHVDEDFFYMEHVLRIAACLHSLVSLAMLIAYYHLKVPLAIFKREKEIARRLEFEGLFIAEQPEDDDFKSHWDKLVISAKSFPVNYWDKFVKKKVRQKYSETYDFDSISNLLGMEKSTFAAQESEETGIFKYIMNIDWRYQVWKAGVTFTDNAFLYSLWYFSFSVMGNFNNFFFAAHLLDVAVGFKTLRTILQSVTHNGKQLVLTVMLLTIIVYIYTVIAFNFFRKFYIQEEDEEVDKKCHDMLTCFVFHLYKGVRAGGGIGDEIGDPDGDDYEVYRIIFDITFFFFVIIILLAIIQGLIIDAFGELRDQLESVKDNMESNCFICGMGKDFFDIVPHGFDTHVQKEHNLANYMFFLMHLINKPDTEYTGQETYVWNMYQQRSWDFFPVGDCFRKQYEDELSGGGGGG

>Drer_RyaR1

KGETGVMVSSTLKLGISILNGGNSDVQQRMLDYLKDKKDVGFFLSLQALMQTCSVLDLNAFERQNKAEGLGMVSEEGTKEKVMADDEFTCDLFRFLQLLCEGHNNDFQNYLRTQTGSTTTINIIICTVDFLLRLQESISDFYWYYSGKDIIDEPGKRNFSKAMTVAKQVFNSLTEYIQGPCTGNQQSLAHSRLWDAVVGFLHVFAHMMMKLAQDSSQIGLLKELLDLQKDMVVMLLSLLEGNVVNGTIARQMVDMLVESSSNVEMILKFFDMFLKLKDIVASDAFRDYVTDPRGLISKKDFSKAMDSQKQYTPSEIQFLLSCSEADENEMINFEEFANRFQEPAKDIGFNIAVLLTNLSEHVPHDTRLQNFLEQAESVLNYFRPFLGRIEIMGASKRIERIYFEISEANRNQWEMPQVRESKRQFIFDVVNEGGESEKMELFVNFCEDTIFEMNIASQISEQEEEVIEDEDEEEEEESGGGGGGEGEGEEGNGEEAPPESSSAFADFIKSIVNFLSLFTFRNLRRRYRKIRKMTIKDMIVGLVMFLYTVLFGILHFIYSVCKGFFLLIWNTLFGGGLVEGAKKMTLTEILTNMPDPTQDEVHGDLPPEPGARKVQEAGGAGEGEEGAGGGEEEAEEGEEDEDGRAGFGPHGGLGDMGETEPEEPPTPEGSPLLKRKLVSEEGGEGEVVVEEEKVEEEPAPEPEKADAENGEKEKEAEPETEDKQEEEQAEIAKAKAKAKKGKKEEEVGFELWNELEIQRIKFMNYLSRNFYNLRFLALFIAFALNFILLFYKVSDRLPGEEEEFEGSAMFEGSGAFEGSAVFEGSAVFEGSGVFEGSGEAEGSGMEENGEEDEENGFVYYFLEESTGYMEPAMAFFSILHTIISFLCIIGYNCLKVPLVIFKREKELARKLEFDGLYVTEQPEDDDIKGQWDRLVLNTPSFPNNYWDKFVKRKVLEKYGDIYGRERIAELLGMDLASLDVSAMTHEKKRQPDPSMFTWLTSIDIKYQIWKFGVVFTDNTFLYLVWYTVMSLLGHFNNFFFACHLLDIAMGVKTLRTILSSVTHNGKQLLMTVGLLAVVVYLYTVVAFNFFRKFYNMSEDEDEPDMKCDDMMTCYLFHMYVGVRAGGGIGDEIEDPAGDEYELYRVVFDITFFFFVIVILLAIIQGLIIDAFGELRDQQEQVREDMETKCFICGIGSDYFDSTPHGFETHTMEEHNLANYMFFLMYLINKDETEHTGQESYVWKMYQERCWDFFPAGDCFRKQYEDQLG

>Drer_RyaR2

YEKSWIETEEHYFEDKLIEDLAKPGVEEPPEEDEMTKNIDPLHQLIQLFSRTALTEKCKLEEDHLYMAYADIMAKSCHDEEEEDEVKSFEEKEMEKQKLLYQQARLHDRGAAEMVLQTISASKGEMGPMVASTLKLGIAILNGGNSTVQQKMLDYLKDKRDVGFFQSLAGLMQSCSVLDLNAFERQNKAEGLGMVTEEGSGEKVMQDDEFTCDLFRFLQLLCEGHNSDFQNYLRTQTGNNTTVNIIISTVDYLLRVQESISDFYWYYSGKDVIDEQGQRNFSKAINVAKQVFNTLTEYIQGPCTGNQQSLAHSRLWDAVVGFLHVFAHMQMKLSQDSSQIELLKELMDLQKDMVVMLLSMLEGNVVNGTIGKQMVDMLVESSNNVEMILKFFDMFLKLKDLTSSDAFKEYDPDGKGVISKRDFHKAMESHKHYTQSETEFLLSCAETDENELLDYEEFVERFHEPAKDIGFNVAVLLTNLSEHMPHDSRLQTFLELAESVLNYFQPYLGRIEIMGSAKRIERVYFEISESSRTQWEKPQVKESKRQFIFDVVNEGGEKEKMELFVNFCEDTIFEMQLAARMSDAGERSASKEDSEREKPEEETPEMGFFSITTVRTALFALRYNVMLLMKVLSMKSLKKQVKKAKKMTVKDMVTTLVSFYWSVLLGLLHFAFSVARGFFRIIYNSFLGGSLVEGAKTMKVSELLANMPDPTQDEVRGEMEDGEKKPVDKTSPEEDLADLTVATDETDLLSDIFGLDLKREGGQYKLMPHNPNASLTDLLNTPVPPPPTPSPELRRRHHTKGSSSSTDNNDAAEETKSVPEKAEGENVEKAQKKKEEAVKRKVRQHYEPDLQESAFWKKIIAYQRKLLNYFARNFYNMRMLALFVAFAINFILLFYKVSTSSAVTEGKEVLFSASSDTSVQWDPLSGSSSRPVTVHFVLEESSGYMEPTLRILAILHTVISFFCIIGYYCLKVPLVIFKREKEVARKLEFDGLYITEQPSEDDIKGQWDRLVINTQSFPNNYWDKFVKRKVMDKYGEFYGHDRISELLGMDKAALDFSDAHEKKKPKRDSSLAAVLNSVDVKYQIWKLGVVFTDNSFLYLAWYMSMSILGHYNNFFFAAHLLDIAMGFKTLRTILSSVTHNGKQLVLTVGLLAVVVYLYTVVAFNFFRKFYNKSEDGESPDMKCDDMLTCYMFHMYVGVRAGGGIGDQIEDPAGDEYEIYRIIFDITFFFFVIVILLAIIQGLIIDAFGELRDQQEQVKEDMETKCFICGIGNDYFDTVPHGFETHTLQEHNLANYLFFFMYLINKDETEHTGQESYVWKMYQERCWEFFPAGDCFRKQYEDQLN

>Drer_RyaR3

AKTPMTVVEEEEEEEAEVQPDPLHQLILHFSHNALTERSFLEEDPLYIAYADMMAKSCAENEEEEEEEEGKEKTFEEKEMEKQKILYQQARLHARGAAEMVLQMISASKGRLGPMVTGTLKLGISVLNGGNVLVQQKMLDYLKEKRDAGFFKSLSALMMSCSILDLNAFERQNKAESLGMVTEEGSISVKGRGSKVLQNDDFTKDLFRFLQLLCEGHNNDFQNFLRTQTGNTTTVNIIISTVDYLLRLQESISDFYWYYSGKDVMDEAGQHNFSKALSVAKQIFNSLTEYIQGPCIGNQQSLAHSRLWDAVVGFLHVFANMQMKLSQDSSQITLLKELMDLQKDMVVMMLSLLEGNVVNGTIGKQMVDTLVESSSNVEMILKFFDMFLKLKDLTSSDNFKEYDPDCKGMISKRDFQKSMESQKQYTQSEIEFLLSCVEADENDMFNYSDFVERFHEPAKDIGFNVAVLLTNLSEHMPHDSRLSTFLDLAESVLSYFEPYLGRIEIMGGAKRIERVYFEISESSRTQWEKPQVKESKRQFIFDVVNEGGESEKMEMFVNFCEDTIFEMQLASQISEPDPTERPEEEEEEELHSLLEGLSEEEDASLESASAFTAACASVKKSMSNFRQMLTFKSMKKQMKKFRKLTIREMITGFFSFFWMLFTGFFKGIFGIIFGFFHIIWSSMFGGGLVEGAKNMKVTDILGNMPDPTQFGIHGDVLEAEKAEISEMVVSADMVQMTGAGGGEKLETDVIMDLLNPAVKKEGKHGAEPGLGDVCEILTETSAPVEKKKSQKVAAEKPESEPEKADTENQEKEDKTKEEAPEEPAAEKPAPKVKRGSSKDESPAFMASFFAGLEVYKTKMLNYLARNFYNLRFLALFVAFAINFILLFYKVTGDFDDEEEEEGSWGGEEEEADEDEGGMEYFILQESTGYMAPTLTFLAVLHTVISLLCVFGYYCLKVPLVVFKREKEIARKLEFDGLYITEQPSDDDIKGQWDRLVIATPSFPNNYWDKFVKRKVINKYGDLYGAERIAELLGLDKSALDFDPTEEAVVKEASLLSWLSSIDTKYHVWKMGVVMTDNSFLYLIWYTTMSILGHYNNFFFAAHLLDIAMGFKTLRTILSSVTHNGKQLVLTVGLLAVVVYLYTVVAFNFFRKFYNKSEDEDAPDMKCDDMMTCYLFHMYVGVRAGGGIGDEIEDPAGDPYELYRILFDITFFFFVIVILLAIIQGLIIDAFGELRDQQEQVKEDMETKCFICGIGNDYFDRTPHGFETHTLQEHNLANYLFFLMYLINKDETEHTGQESYVWKMYQERCWDFFPAGDCFRKQYEDQLG

>Drer_IP3Rt3

LAACAEGKNVYTEIKCTSLLPLEEMVKVITHEDCITEVKIAYVNFVNHCYVDTEVEMKEIYTSNHIWKLFENFTVDMARVCSNREKRMSDPVLEKYVIQVVLDTVTAFFSSPFSENSTSTEAHHTTVKQLLQSTMRLLDCPWLQPQQKVQVESCIRTLAVTTKSRSIPLPVELEAHVNMMLSHSNSLTLSRSSHSNKSMSRLTRPAAPTNPWDYKNIIEKLQDIINTLEERVMPLVNAELSVLVDVLHQPELLFLEGTDARSRCESGGFISKLIQHTKALMNSDEKLCIKVLRTLQEMLIRELDFDEKGFALRKVLLQNYLYNNKKNIKAELVEHAGGAEGERDWLTVAALQCRLDKEGGTKLFTDLITSTKNEKIFQESIQLAICLLEGGNTEIQNSFYKLMMGDNKSEKFFKVLNDRMKNAQLDIKSTVSVNVGEMSNKAKDDKDLETGGVSSFGQPEPQPEQQEVETEMGPSVTIMKPILRFLQLLCENHNQDLQNFLRIQNNKTNYNLVSETLQFLDIMCGSTTGGLGLLGLYINENNVELITQTLETLTEYCQGPCQENQTCIVSHECNGIDIITALILNDISPLCRYRMELVLQLKDNASKLLLALMESRHDSENAERILFNLRPRELVELIKKAYLQEGECKEGEVSPREVGHNIYILALQLARHNKVLLTLLKPVKKIKEEEEESISSMLNLNKQQEEEKEDPLEHYDRQTAQIEIVREDRSMEQIVFPVHPICEFLTEESKFRVFTTTEQDEQGSKVTNFFEQTSFLHNEMEWQKKLRSMPVLYWFSRRMSLWGTISFNLAVFINLIIALFYPHDSGHSGSIDSSLLLMGFWCFAGLAVLGLLFKRYGFQSLTVAITLRCIYHFGIGPTLLLLGALNLINKIVYLVSFVGNNGTFIMGYKAMVMDVEFLYHVAYVLTSSLGLFVHEFFYSILLFDLIYREETLFNVIKSVTRNGRSILLTAVLAIILVYLFSIVGFLFLRNDFIMEVDHLASPAPGDTESFMSSCSSDGIDCTEETGLLAPAEDEDNTERACDTLLMCIITVLNHGLRNGGGVGDVLRKPSKNEPLFPARVVYDLLFYFIVIIIVLNLIFGVIIDTFADLRSEKQKKEEVLKTTCFICGLERDKFDNKTVSFEEHIKLEHNIWNYLYFIVLIREKNKTDYTGPESYVALMIKNKNLDWFPRMQAMSLVVTDGDGEQNEMRNLQDRLSSTMKVVTQLTSQLTELKEQMTEQRKRRQRMGFVDVQSGSNPGMPVPPSPAGGNQVYKA

>Drer_IP3Rt1

RERLDESSALRYHIHLVELLAVCTEGKNVYTEIKCNSLLPLDDIVRVVTHEDCIPEVKMAYVNFLNHCYVDTEVEMKEIYTSNHMWKLFDDFLVDVCRVCNNTSDRKHADTVLERYVTETIMSIVTTFFSSPFSDQSTSLQTRQPVFVQLLQGVFRVYHCNWLLPGQKGNVEACIKVLSDVAKGRAIAIPVDLDCQVNNLFMKSNNIVQKTALSWRLSVRNATRRDSVLTTSRDYRNIIERLQDIVSALEERLRPLVQAELSVLVDVLHRPELLFPEHTEAHRKCESGGFICKLIKHTKQLLEENEERLCIKVLQTLREMMTKDRGYGEKLLGYDDEMDVTEVVDISLPPKQQEDRQRGEALRQLLVNRYYGFRSGGRRESLTSFGNSTLTPVGPIKSQPGGLSRAEMSLMEVQCHLDREGASDLVIDLIMNTTSDRVFHESILLAIALLEGGNTIIQHSFFKRLTEDKNSEKFFRVFYDRMKVAQVEIKATVTVNTSDLGNKRRDDNTPDKDTPQRRRGKDSGVVVTDDAREQLLEASAATKKAFGSYRRDADPEETFGTADGDKGGGDKGTEQGEMSPVILIMQPILRFLQLLCENHNRDLQNFLRCQNNKNNYNLVCETLQFLDCICGSTTGGLGLLGLYINQHNVALINQTVESLTEYCQGPCHDNQNCIATHESNGIDIIIALILNDINPLGRKRMDLVLELKNNASKLLLAIMESRHDSENAERILYNMRPKELVEVMKMAYQQGEAEFEDEEQENGEDHAASPRNVGHNIYILAHQLSRHNKELQILLKPGGEDQALEYYTKHTSQIEIVRQDRTMEQIVFPVPNICSFLTNESKLRVYYGTERDEQGSKINDFFLHADDLFNEMRWQKKLRAQPVLYWCSRNMSFWSNVSFNLAVLINLLVAFFYPLDGVSESQLEPSLSLLLWVCLLGSLGFVLMSPRPNAVRVLVISTVLQLGFSVGLQHMLTLLGAFNVCNKIVFMLSFVGNRGSFTRGYRVMVMDREFLFHLLYLLICILGLFGHVFFYSLLLFDLVNREETLLNVIKSVTRNGRSIVLTAVLGLILVYLFSIVGYMFFKDDFILEVNRISNGTLEEGVTLPAGFCDARNGSCLSDDVDEDDVERACDSLWMCMITVLSHGLRSGGGVGDVLRKPSKEERLFAARVVYDLLFFFLVIIIVLNLIFGVIIDTFADLRSEKQRKEEILKTTCFICGLERDKFDNKTVTFEEHIKEEHNLWHYLFFIVLVRVKDSTEYTGPESYVAQMIKEHNLDWFPRMRAMSLVSSDGEGEQNELRSLQEKLESTMRLVSNLTNQLTELKEQMTEQRKHKQRLGLLGNPAHLNINPQQPA

>Drer_IP3Rt2

LMYHITLVELLAACTEGKNVDTELKCNSLLPLDDIVNVVTYDGCTPEVKIAYVNFVNHCYVDTEVEMKEIYTSDHIWNLFDSFLVDMATVSIGPTDRKNADTSLEKYVTDTIMAIVKGFFGSQFSVSNSKLQAHQATFIKLLQSACRLYNCTWLNSLQKTNIEGCIKTLADVAKSRAIPIPVDLDGQVNALSLKTHTSMVQRAVKDWRLSARGRPRKEPFGAHDYKIIEKLQGVVSYLEEQFNPRVQAEFSVLVDVLHSPEMLFPESAQLRTEDGAFLSKLIKHTKKLMEKEEKLCIKILQTLREMLDRREMFDEKGNALRKSLLAHYFGNHTKALEYSSSNSSSSGGGSPGQDSDKPGTSLADIQCVLDNVGASELVVDLIVSTKNDRIFEESILLGIALLRGGNTQIQNSFHNQLHKQKKSEKFFRVFYERMEQAQKEIRSSVSVNMFELSCRKREEESEGSGIRHKKVRDSLHLREEMRGQLKDASSVTSKAYSTFRREWDPDIEAVGTTGDATEEVVEEIQMSQAITIMKPILRFLQLLCENHNRDLQNFLRDQNNKTNYNLVCETLQFLDCICGSTTGGLGLLGLYINERNVYLVIQTLETITEYCQGPCHENQSCIARHESNGIDIIIALIVNPIDPLGKNRLDLVLELKNNASKLLLAIMESRQDSENAEKILYKMRPNELVDVIKEAYVQGEEECEIDDETLDLIRSKDVGHNIYILAHQLAKHRKNLQQSLKPGPDPTIDGEAALTYYTNHTAQIEIVRHDRTMEQIVFPVPNICEYLTEESKVRVFTTTERDDQGSKVNDFFQQFDDLYNEMRWQKKIRNNAPLFWVSKHISLWGSISFNMAVLINLAVALFYPFGDDGDEGVLPPFLSVLLWGAVVVSTFTLFILPPRGGILAFLVTVIFRSIYTLGLGPTLLLLGAANLLNKIVFLVSFVGNQGTFTRGYKAVIMDVFFLLHVSYVIVCMLGLFVHEFFYSILLFDLVRREETLLNVMRSVTKNGRSIFLTAVLAIILVYLFSIVGFLFFKDDFLMEVDHLPLKRPNVNGKSMSCVSADCAESMFSRNHVEEEDEDGSERVCDTLLMCIITVLNHGLRNGGGIADVLRKLSKEEPMFPARVVYDLLFFFIVIIIVLNLIFGVIIDTFADLRSEKQRKEEILKTTCFICGLERDKFDNKTVSFEEHIKSEHNMWHYLYFLVLVKVKDPTEYTGPESYVAQMIKEKNLDWFPRMRAMSLVSSEGESEQNEIRSLQERLDTTVSLVAQLSSQLSELKEQMTEQRKNKQRLGFLGPPQMNHQIAPAL

>Cele_itr1

PMDHIVRVVTAKQCLVEVKTVYLQLLLHCYIDTDAEMKDAYKTEYVDHILNNLLEDIRSLRVEKLTGAETATLEHYICHTVTEVLIKFFEAPYSALQQAKVDVHHHKKTFSEVLLELTYLEKGKLRGSKSSRNWYRVAECIKRLTKWAEEHNITLPATLAGPQMSGQTSVRQKWQQAASSAKWIGIGKMSQDSQSDYDSDSSQGPCQISRLNRQNTLNPGHRLYGTSNSMTEHTSANVVTCYHMMIGEFKFYLHPLHAAEGSVLVEVLHTPELLFPEGSALRDQCARGGVVAKLIQHCKTLMQNKQDNLCARVLQTLCKMCDCTKQQLTHQGQQLRQLLLQRYFGHHNNHHPPLDRQQSKIGEVIEAVKEKKEETWSQERDLYAIQCKLNDAGASDLVTDIIIMEPSREIFLKAIHLARALLHEGNDKVQHSFYMRMKQKDIHEPFFKAILTRIQTAQNRLKSDMMSCSDSKPKVSLSATVSRRSSTVLTPLIDAGDTGFNGALFEVPQQVRHPSISEMSQLSNDLTHSIPDLAPYQDEEKSTDALPPEVALVEPILRVLQLLCENHNSLLQNFLRKQSDRTNHNLVSETLSFLDTVCGSTKGSLGVFGEIGEHNFSLITQTLATLTEFCQGPCHENQNTMAMQENGLNIIISLVLNEIKPLADDHMELALEIKSQASKLLLAIMESRHDGENANRVLRNMANMSGGPKQLVHAIKQAYEMTNSNHHMLKSISRDLFRQAEDDLKKKSGPQITVNTVTLPEINVDASGIVSIHTEKNISSSLDDKFNDDDIPSVDPREVGHNIYILAHQLAIHDGELEIWLDGSDEKKDDLTREALNYYKERTAQIEIVRRDRTLERVVFPINDICSYLTKDTKDYVYNNTERDNQGSKVTEFFDEWETMYHEMIWQRKLQDRKWLSWCAFRLPLWTRLSFHFAFIVNALVARYYPLPEHSNSSISLGNLYSWFAVFSSFLLAHYLRHDKIYLHKTSLLILASLCFLLLSSIGVTLTLYIFGILQLVNKIVHVVAFVSNKGLEDRPIAEILACRNLHYLLVYLFICILGLLVHPMIYCILLFDIIFTEETLQNVIASVTRNYQSIVWTGLLALILLYFFSILGFLYFRHDFYLEVDPVENDSSATISSGIPSETCPSEGCPGLQPSEKDDNDDEKKVKSCETLWMCILQTGYQGLRNGGGIGDVLRNPAPWEDMFIWRVAYDMTFFVVLIVIVLNLIFGVIIDTFGDLRAEKNEKEQILKNNCFICGLDRSRFDNRSVTFETHRETEHNIWHYLYYIVMLQIKDETEFTGPESYVAQCVKDRNLDWFPRMQALSLQDSELDTDQSEVKQMKDQLLQMMTLMREIISQNEESRAFMEQFQPR

>Cele_RyaR

EKETNPDPLKQLIRCFQRAATSEETAASAIHEDSLYIRFADVMAKSIHIEEEDGEDGEEGEIDQAAKEEQSQALRGEQAVLASRGAAIMCLMYLSASGGEPNEMVAQTLQLGIHLLSGGNVEIQKMLIEYLQLKKDVRFFTSMAGLMNKCSVLNLEMFERQIKAEGLGMGAELASGDNQNLNDADFTCSLFRFLQLTCEGHNLEFQNYLRTQPGHTTSVNLINCTVDYLLRLQESVMDFYWHYSSKEVIDEGGKEYFLRAIQVCSQVFNTLTESIQGPCVGNQMTLANSRLWDAINGFFFLFAHMMEKLYKNSTQLELLREFLNLQKDMIVLMLSMLEGNVLNGSIGKQMVDALVESQPSVEKILKFSDMFLKLKDLTTSQAFQDFDTNQDGWISPKEFQRAMESQKMYTVEDITYLMMCTDVNNDGKVDYMEFTERFHNPARDIGFNLAVLLVNLKEHITNDPRLEKIIEKAQTLLEYFDPFLGRIEIMGSSKRVEKIYFEIQESWLEQWGKQQIRDSKNSFLFNVLQDDGGDQGKLEAFINFCEDTIFEMQHAAAISSGDSDTKMERAIKQRDYFLQQTSADHISETFKSGYNYGISAASALSPQNISTTMRNVSSSVRQMSWTQLLYAVIILFIRAGLAIGWAGYLLLMTIFRFGYFLTTSSEEEEAARQEKEQAKMNNEHPSFNPPIIQEFHHSHVGVTAFGVGMNADHLNVNSLPDFVPPERPETPETVLEEEKPLNQETSPPTSPTSPASKAPSIYESIGAPQMVQLQSEADFQQGQYEPKIAESNSTKSRGSILNMLARNFKTIEKITLYLAFFINVILLFHRVDISHAENAEAASEGDDDEDALESIFITGMQFPYVEYEITGWMLAQILYWISVLHLSTSFALLVSFYQLKIPLITFKREKEIARKLMFDGCWITEEDSEELGIVDTFMWYLDRIVVSAKSFPMMYWDKFVRRKTRSKFKDQVDEETLTSILGEEKMSTDSSYDYRYSCWLWIGVILTNGQFLYRVGYLLCSACGVFLSPFFYAFHLIDVVLSFPMLKAILQSVTHNLQQLILTIMMTLVVVYLYTVIAFNFFRKFYVQEGEEGEEPDRKCHNMLTCFIYHFYAGVRAGGGIGDELESPYGDDLEYPRMFYDISFFFFVIIILLAIMQGLIIDAFGELRDQQESATEKLESSCFICDIGKETFDRMPRGFEIHTTKEHNFANYLFFLQHLVNKDETEYTGQETYVREKYDNRDWDFFPVGECFVKQYEDQLLQS

>Ctel_159249

EGNSEKSVDVAIERILAMAKVLYGLHLVEHPTGSMEMQWKKAVSAQRKRAVMACFRMIPISKLPRQGSCTCFFMHRAINLFIKSYREIWLKSQLAGHGCLIEDVTGPPEDEVAAITSAAEDGSEEKAEDPHPLSQLITAFSRSATTQNSQVLEADFLYISYAKIMSQSCHGTDDDDDDGGEGEEGDDAGASLQEQEMEKQKLLYEQNRLADRGAAEMVLMYISASKGQWSDMVNATIDLGISILRGGNVDVQKRMLEHLKEKKDVGFFTSVAGLMQQCSVLDLDAFERCNKAEGLGMTEGSSNQVKNLQDADTTQQLFRFLQLLCEGHNLEFQNYLRTQAGNTTTVNIIICTVDYLLRIQESMMDFYWHYSGRDVIDPPGKESFFRAINVAAQIFCSITEYIQGPCAGNQLALAHSRLWDAVGGFLYIFAHMQDKLSKDPEQLELLREFMKLQKEMMIMLLSMLEGNVVNGPIGKQMVDTLVESSANFEMILKFFDIFLKMKDLTSSEAFMEFDTNKDGVISHKEFRRAMESQKMYTPEEIEYIMMCVDANQDGKVDFNEFTDRFHNPAKDIGFNVAVLLTNLSEHIPNDPRLDRFIERARSVLDYFEPYLGRIEIMGGAGRIERVYFEIKQSHIDQWEKPQIKESKRAFLHSVVAEEGDKEKLEAFVNFCEDTVFEMQHATSISAEEQSLQMASYAAARPGTETKGGVVESIKWGMGHVKDAMWTGLSALHPSNIREKYHVVRAMTWKERAKGFLRLNTSTAYGVVWLIFMLIWLFLKFIYIMMRGEPRTEPKQTKQPSKISAATERDKDPSGGIPGLMLDPTANAPKPPLDKKPDLDLGDKGALNEPVGMITQTETNAEPMDESEVIGEDMETDISVEMDEPPIELKDKGSDFVQFLLSMCARNFYKFKLIALAMAFSINILLLFFKVDSASAELAAGAEGEDGADAGDGDGDGYEFISMADNVTYLHPLLRIIALLHSLVAFAMMVAYNYLKVPLVVFKREKEIARSLEFDGMWVAEQPSDDDIRGHWDKLVISTRSFPYKYWDKFVKKKVRNKYSEQYDYDQISTLLGMEKADGFNASLGPKEVTSILPSFMTEIDWQYQFWKSGIILTDQSFLYIVWYFSFSVLGNYNPFFFAAHLLDVAICFKTLGTILQSVTHNGKQLVLTVLLTSIVVYLYTVIAFNFFRKFYVKDEDGDVDYKCHDMLTCYVYHLYVGVRAGGGIGDEIESPDGDPYEGYRILFDITFFFFVIIILLAIIQGLIIDAFGELRDQLEQVKEDLESSCFICGIGKDYFDKMPHGFETHVKNEHNFANYMFFLMHLINKPDTEYTGQESYVWELYQERSWDFFPVGDSFRKQYEDELGTS

>Ctel_222687

DQSSPLTYHINLVQLLACCTEGKNVYTEIKCHSLLPLDDIVRVVTHPDCIPEVKNAYINFLNHCYIDTEVEMKEIYTSNHMWTLFENFLVDMAMVCNATHDRKHADRAMEDYVTITVMNVISTFFNSPFSDQSTTTRQPVFVRLLQGAFRTSHSTWLSGQQKFHVENCIKTLSDIAKSRSIAIPVDLDSQVNTLFNKSQLVMKHTQHWIKAGKSRRDSTMQINRDYRTIIEGLQDIVSLLEDQLKPLVQAELSVLVDVLHRPELLFPLNTEARHKCESGGFISKLINHTERLLEEKEEKLCIKVLQTLKEMMTVDLEYGEKGEALRQGLLTRYYGSQHPRVAGRKEGPGPPAVQQAGGHGVNSSPGSRLLSRAEMTLHEVQCHLDREGASDLVVDLIVNNHSSRIFLETVELGIALLEGGNSAIQRSLYNRLLSDKNSERFFKVFYDRMREAQSEIKATVSVNTSESLAQKSQEEEQDSGRKKCQCVTCPDYQLCFVLTAKSTPLSPELKEQLDDAAINTSKALTHAKAASHPDDGQVVVTDELLAEKKEKEEKKISAEIAVMQPILRFLQLLCENHNRDLQNYLRNQSNKTNYNLVCETLQFLDCICGSTTGGLGLLGLYINEHNVALINQTLESLTEYCQGPCHENQNAIASHESNGIDIIIALILNDINPLGKYRMDLVLVLKNNASKLLLAIMESRHDSENAERILYNMSPKQLVDVARQAYQQAENIDEEGEEEEEEEEDEEEKTNPKDLSKHNKDLAELLKPPPDIPMDPALEYYEKHTAQIEIVRQDRTMEQIVFPIPEICEYLTPDTKSRVYQTTERDDQGSKVSDFFERTEDMFSEMKWQKKLRANAILFWLCSHMTLWKSISFNFAVLINILVAAFYPFSPTIIEIDPHLSGLVWTAMFVSLAIVITLPRPLGLRTFIVAAILRLILSVGLECTLMLLGALNVCNGVLLAVSIMGNSGTFTKALRLILMDKEFLYMMGYLITSMLSLCLHEFFYSILLLDIVYREETLLNVIRSVTKNGRSILLTAMLAVILIYLFSIVGFIFFKDDFVMEVDALDAIGINKDVVAAAATSLSGAAVMSGTCTKDAKDCNVTEEQARVIPDKEAVADLVAVEEEEEEEERERFCDSLIMCIVTSLNEGLRNGGGIGDVLRKPSRTEPLFVARVVYDLLFFFIVIIIVLNLIFGVIIDTFADLRSEKQQKEEILKNTCFICGLDRSSFDNKSVSFEEHCAFDHSMWHYLFFIVLIKVKDPTEFTGPESYVYAMIKEKNLEWFPRMRAMSLAADDGEGEQNELRNLQTQLESTNKLVQTLSSQLSELKDQMTEQRKQKQRIGLLSNPSMPIMPSGATQAL

>Ctel_203606

HDKKMWKVITGLGEELNKITEFSKDNSETVKQLLKKSPSKSQNLVDRIMDDPDVLNSILSEKGNGEWKTIHGSLHYLLDAVVPFLQVFFHNYYQPDRENFPDEPDELDKLANCFVEFIKVVGPLISKQQQMKGIVGCMNVLLSASTLPVEVMEEFQEKYGGATSVQDIRSDARKKYEEYYDQEEELNAQLNIFSINYSTVYGGSNDLKTQLKFNSDKEYTDLGGDEELPLGEEFQEHVACFINPSASNIKAKYQMAGKLVEQLAISAANAQMNEKERVNQEVLDIKCLQLLRAIIHNEVVKLPDDWENDLKANRKQLKRIIDSQNALNDHMALLKVLPHLSKTGDDIVREVLAFLAAMLFGGNEDVQRSLIDYFLGTREENFFFALKNRMQLSALATKERRSLIAQHQAKVEEAIEQAKALRKALKTGQMAANEIKMANQMGSALLAVRNSRTSMGGKKGRNDALKGRYGKKSSNRMGSNRSMNRSQMMGSSLFAPKGIGGSAFGGGRLNVPGGMGAAQSSAFLQEDNMMQMPGKGDKIPLVEIMHIKDNKVSPEAQTPEVKIEELDETDLADLAAMSIEDGDILEYKDDGYIELVLKILGLVCDGQNQILQNYLREQLDNIKSVNLIAETARFLSLVYSSISVKTIGLVTELFSTLVEFTSGNLHNQVVVFDCKICDYINFILRGASFQGCSVKEILQLKKGIGTLVTAMIEENGQVASENIVADHKQATNTSKPDGDPDRPSSVEVAETLDAESIWQVSAEAYAKSLILPNGEMKEMYLDIGFCYFHLIQRIIDITEQGASVKPKLLKTMEDQAAWDHYSNGTLSIEIVKDDKLQKIYFRCKDKNVLREDAKEKFKYEVDRSSPSNKLREFMDWSKDIMVDIKYTRKVMSFFLSKFFVKYCFFISNHPTFPRFTRLRNFCKSLRKSRDLGDEEDDDDQERDSNLEVKFFSVKTFWYMLVFAFSMLGTFFYGYFFSFHLLHMANFNQLLKRAIQAVTKNGFSLLLVFGYMLAIVFMYAQLGFAFYRELFDSVAGLHCTTMYECAVTMLHRGAAIGVSLFLVFIYGLVILFIFSLVSFAFYREIFNPVTGHYCANFYECMVTTVHRGLIDGMYNFLEVPEGKPFTYHLFKSIYDLTFFIIITTIGLNIIFGIIVDTFSELRDNKWQVDSDMHSTCFICSRNSYDFEHQGGGFEHHVKHEHNQWAYLFFFIHLDETRTNDYTALELYVSRLLERENYDFFPLNRALSLQHEEDNNELRLETLMHQVTYLVSKMKQEEADREREQEKVRQLEWERKHRNRAQSSKPRNEKP

>Ctel_215467_215466

ILSVKVYDDQAYIIFSIIDKIRARCTAAEDSTVKTIAVLLINQAISKNIQMRCTEETGNLFSFSQDLLSLTEIGSVLNHKDISLPIKHAYLTFFVGVHFRSSVLIESVFKFRRIDNETEPRGYAAQFTYLIMNTFFPFIKKFDEFFVMDPDNFPDECKIFTRLSAKIAGFIPLVGIALTRVNEVRCVLDTLQCIKERMQWGEGLPSVSIRETTLNETFQNFIKNCIAVLKIPNTVLAQIGYKGYKVYRNEYTTIGSADELPLGADFQRMVKAFVVGNNGPMNVRYRHAKMLVKQLRITASKLKKDSGGDFELELVCLKVLRAQLHNYIVILPEISSIKREKGKYHRHLQRITELQEWYTSLDIIPITVKLMACQNLDIFKECMAMIDVLLYNANVTVQDTILDCFKNTREEVFFMMIKEYLQTSIQLTKEKRLLERHYRYKQLEACSEIKHETKNMFYARNLRRYQGGDLALCSSTSNPQTGKDETSVQHYAEHAVTVTLILKVAAMMCDGQHRGLQDYLREQPDNLRTFNLVSSVTSYIGTLYTVIQSDNISLNIQGNQENRQVILDNKLIDYVNSILRINSFGNCPLKKIVELLHAIVDLLISLVEENGTNASRSAKDVAETLDLNAVLSVMDWCYEVQFNEMVKQVGCKICMVYTRIVDISPTLEATVDSKTLSISIKALEHYKQQFESVEIVKDGFLNRIYYPVDRSLEVRHEVKEKLKWNVDRTSSTNKIRDFVAWSKIIIEDIHYHRKVTKNPIINLIVKKWSVQLFLALSFMGSFTYGYFFAFHLLNIITTNELLKGVIRAVTLNGRSLLWVGILGVLMIYIYALIGFAFFRPSFDPENNLHCRSLFECFVTVLRYGITGLIDEVLILNPLESNFMQFGYLAIYQLSFFIFISTIGLNIIFGIIVDTFSELRDKKWTAGKDMRENCFICGLPCYEFEHKSKGFIHHTKFEHNMWAYIHYFIYLSNTSPNDHTALDAYVAKKRAQEAYDFFPQNKALSLASSNQTNETNKIDLLSGKIQYLIDIHKKEFIDM

>Ctel_189051_189050

AESVVMFGRAMDPMISAWAHRKTYARTLRAVFQRTDYKEPQKYLNQYDDEGDINEDFNMFAANVKKIFGGENTVEAQIGYPLKRPYTRFDSDEELPLSPEFQILVQCFIDQRPSLLLHQKYGRAMHLINYFRFSCKITKDKKLREGKENVDMKAMMVLTGLIYNEIIKLPEDWEKKQQKNKVIRKLLKSIKELQNLINSFNAIPSIMLHLSHNSSDDAVRQVLAFLSVMLFNGNPNVQKSLFDYFKSTREERFFITLKAKTQQAAMEIKGIRMVHQKYKSAILDTVESVKMLAHKAGAVVSTLKDKRQSYNRNVTVHVKKMIEVVAKETSKDKIEDCMNTFIREQPDASKVGNLGLILKIMGLMCDGQNRNLQDYFREQSDNFLSCNMVAETSLLLDVLLADVSDFTIVLLGSVLQTLVEFASGNQRNQQVLFDSKVIDFVDYVLQEKRLNECSDMLMLWVKKCIGNLIRVLTEENPSDDGDDSSKVILESLDADSVGRMVSGCFYQSRLYKYTDRQARAWYLEVGFHYFLILCRMIELCHDGNAIKRDFLNTKGNEEVWKYFSNNTMSIEIVKNDKLHRINFPVTCRGALRKKVKESFNYEVDRNPSERIKNFMEWVNEIKDDIAYTRMIYNNIISRTLINRWQYLNTTMIFLSSLQCVLFTACWDADEDDSVKRRIRFRPIDQVPEQRKEHTFIRSHFISVACLWYLVCSSHHSGISYHSNDLSPQSLPFVSVLAMTHDGFFFSYHLLHSAASNQIINRAIRSVTKNGISLLLTLFYALILIYIFTQVHFVFYREMFTDSFCNTMYECFVSVLHVGLIVGPTEGFASPENKPFTYLLGKAAVDLSFFIIISTILLNIVFGIIVDTFAELRDSKGFERHVKQEHNQWAYVFFMLHLDETSTNDYTALELYVSRKLAKEEYDFFPKDRALCLEYIEDQEERKLAILEKKMSYMVQRIKQLEAEVEFEKERSRQEQWEKENKTSKKLLSKQPSVFALIAEAPPSAVIDQPSSSLGDQRYMEALERPQTSILDSRRQANDEVITLEDAESTEEKPK

>Ctel_201920_224137

SIGNIVISMVEENSPESLQVAKEVKDTLDKEAIYRCLTACYETTQPDRAKYNANPLLAEEEPKGPLNIGVTLQSARNLAKLGTQMLKSKQQDVKVDPETKEMLDEVGFTFYLILARVYDLDPKLVKTEVNITSQQMEAFNHYRKNTMSIEILKEDHLQKINFRVKNKNVLREEVKEKLIWSVDRSSPSNKIRDLMKWSKDILKDIYYQRKILSNPLATFFTKNWMVTLLSVVINVLMLFTWNARSSVSDYKLNKTSVIPADLKEPFWNKVVILLSLIINIFMLFTWNATWSIGQADPPGFNASTIEGVDQLPKDIKDPLPNVTLAEYDVIIYGLGGAHNFFSFLVLLTYFLSNHPTLPSLSGFRKKIRSLCSHQLEKDEEDDEKDHISKLEVKFLSFTTVYYMVFLGMSIAGTIFQGYFYAFHLLNIVNNNQLLKGVIQAVTQNGKSLLWVAVLGLVVFYIYALISFALLRSSFDPDDGLYCATLWQCTITVIRYGLTAEIFDSIQTHPAENTFLKFGLLVVFHLSFFIFITTIGLNIIFGIIVDTFSELRDLKWTAERDMRDTCFICSRGSYDFEHHGTGFEHHINQEHHIWAYIFFFIHLHDTKCSDYTALELFVFKLLSQEKYDFFPLNRALSLSLMDEDSTESKVDDLLHFVMQLVDRQREEDAKKKREDEKLKQLRWKELHRSALGPAHRMDSVDTADSSNGPGALPGFHDPSSQINRQKRWKRTTKLAGVLQGLSNLGGVGIGTIQPPVPSANRFAVADSLSGDDLLEGSPSLPHDQSTTETIPLEQRHSPSPSQSDRPPYTPKNGIDDSVHLIDFEEEDEDRDDMSPSPPPPPHPMGSMFSGSDMSLPRHQSPSPPPSLPIRGGHIQDRDARSSSSLSDKPPLPL

>Lgig_123762

PSLAQRSAWKKLISTQRKRAVMACFRMIPLHSLPRHRAINLFLKAYREMWLETEELDKTILIQDLTKGDETVEEGEKNETEEECKPDSLTQLITALSRAATKEQQSSLPEDPLFMSYASIMMRKCDNLMFLVSFKLVYLLSIVHNEQEMEKQQLLSEQSRLSDRGAAEMVLLYISASRGEFNMMVENTIDLGISLLRGGNIEVQKRMLRHLKDKKDVGFFSSIAGLLEKCSVLDLDAFERTNKAEGLGMSTDTGSTDQNLHDGEFTCKIFRFLQLMCEGHNLEFQNYLRTQAGNTVTVNVVICTVDYLLRLQESMMDFYWHYSGKDVIDVSGKDHFCRASMVAKQVFCSLSEYIQGPCSLNQLALAHSRLWDAVGGFIYIFAHMQDKLSKDPDQLDLLREFMELQKEMMIMLLSMLEGNVMNGPIGKQMVDTLVESSSNVEMILKFFDIFLKMKDLTSSEAFLEFDTNQDGWISHKEFRKAMESQKVYTEEEIQYIMMCVDANHDGKVDFHEFTDRFHNPAKDIGFTMAVLLTNLSEHMPNEPRLGRLLEKAKSVLDYFEPYLGRIEIMGSANRIERVYFEITQSHIDQWEKPQIKESKRSFLHSVVNEGGDKEKLESFVNFCEDSIFEMQHATSISAEEQRIAALKSGGFGGEGGVLEPISITYRFFKDLLFSFFSFFTWTNIKKSYSAFKSMTYWQVFVALIKLTFKLSFMTICFGFNIVWTLLKFFINMMMGDKSEEEAKQRQKEKELAAAAEAAKLAPLALGPPGIPLTPIKIDPIEINRSDTDTDKKSNGLPSNGSVTNVEAGTPPAATSENKSTTTKADLETQYMELEEDEAFFQESVEENQQPLLQASTQNVYQPDMSGGQHVDKNRTLLNWIDKKQTVFLFQSLFARNFYNFKFMALVLAFILNCMLLFYKVRFFCLTVSVLFANSLLWWWNETITVSTKTEEGAAGNETAEGGDEDIAEIVTMEDDKTYLEYVMTGLAFLHCLLSLSILIAYYCLKVPLVIFKREKEIARKLEFEGLYVAEQPGDDDLKAQWDRLVLSTRSFPESYWDKFVKKKVRSRYAEQYDYGAISGLLGMEKQNVHKETTAPTKSYLPAFLSNVDFQYQIWKWGAIFTDNAFLYSAWYFLFSVLGNFNYFFFAAHLLDVAIGIKTLRTILQSVTHNGKQLVLTVMLTCVVVYIYTVIAFNFFRKFYVKEEDGQVDQKCHDMATCFVYHLHTGVRAGGGIGDEIEPADGDPYEVYRILFDVTFFFFVIVILLAIIQGLIIDAFGELRDQLEQVKEDMESKCFICGIGKEYFDKVPHGFETHVKNEHNFANYMFFLMHLINKPDTEHTGQESYVWEMYQQRCWDFFPVGECFRKQYENELGI

>Lgig_236387

LKKVKLFVENNKEETKQLLKRPPSKSDKSTLEKENMRGTLHYIFDGVMQFLQIFCRSYYQPDSMFPNEKKELSQLALDYEAFLEVIAPLVCDETQLKNLLGAMTGLITATNAISKSKMEEFHNTYGHLSGSDIHSDSRKKYEEYYAAEEEINVNLNVFVVNMELSYGGVNTVENQIGYPSDQEYSEKGEDEDLPLGQEFQNHLKCFVDSHTKDPTKRYRLAEKLVRQLLISSNLTNLSEKERLDQTQLDIKCLKLLRGLIHNEIVKLPFDWEADIQSHKKLLKTIESVQTAINSYGVVDSLCNHLSRPVDDIVRELLAFMCALLFSGNEDVQTSLYEYFTGTREETFFFAIKDRMQLSAVATREKRLLHAQHQAKVDDLIAQTRALQKAMKEGQTTNTAFLTSQLGSMLSVAKSRTSLRGSGNYRKSKLANGKSSLNGCKISLKKSTVQLPNGFKQKNGSVIMNNNKNNKVLAKNKASVVPTTDEDVDIKIDQLYEEEIAELTNYFVFIYMQLDKEELTNYFVFMYILEQLDEEEIAELTNYFNYLREQPDNIKSVNLVAETTKFLSILYSSVNNNTISLITALFDTLVEYTSGNFANQTVVFDYKICDYINHILRSEIYKGCDAEEVQTLKKSIAMLIRVLTEENPPSAVDDDNALSLVQEIFEYLDTDALSAVMVKAYNDILSVFNPSNNPSILNYVCPAGAARMELNDRTSRHNEQENQTHTDNYNLPNLTRKVGFAFHHILCRMIDLHGEETLSKICKNPEITEAWEYFSCNTLSIEIIKDDVLQKMHFQVKDKNVLREEIKDKFKYEVDRSSPSNKLRDFMDWSSDIIADIRYQRKIHSIPLARLLVKTWPVLNVSVLFLSVIIALMILITWKADGSTTSTVPDTSNYPFAFEATYILGGIHNLLTFLMIITYFLSNRPSIPTWVEIKNFFKRRGIGKKNKEENEEEIKTNHLQARLFSFLTFYYLVFLLFSILGTIFHGYFFAFHLLHMAMLNQLLKRVIQAVTRNGLSLILVGLLGLAIIYIYALAAFAFFRDVLDSNDGRQCNTMIECFITVIHHGLSDGMYNTLEGQLSGASFPRTIEVAVYDVTFFIIITTIGLNIIFGIIVDTFSELRDSKWQIDNDMKSNCFICSRENYDFERQALGFSHHVKKEHNQWAYLFFFIYLDETRPNDYSALELYVHKMRCNNSLDFFPLNRALSLQHEEDSSEKKIETLMSQVDYLVKKMKETQVEKEKDKEKQRQKEWEEKHRKSSKTSA

>Lgig_163525

ELNIKCLQVLRAIIHNEERKLPEDWETRTSEPKIQAQLKIISTIQNFYDARGSMNKSLPHLASRNDEIAKEVLAFLCIMLFNANAVVQSSMLKYFLSTREEVFFMAVRDRMALSTNSIKEKRSLQAQQDAKLKDAPTGGKKTVSTFAVSLAALKQIKAYEDALRQQRIGGWATKKKPPGKKKKQKKPKEPKKRKEPKKVKKETQANGLKKEVNFTNPLVIKEDQALIDPPEHELEIAVQIESEPEPEPAQAEETNAALEYRNDGYIELVLKVMARMCDGQNKQLQDYLREQPDNVKSFDIIAEVTRFLNVVYSNITGKNIDLVIQLFETMNEFTAGNQENRIVIYDNKIIDYINFIMRAGDFDNCITDKILELRQSIANLVMSLIEENGDGPSQVAMEVKDTLDKKAVVALMTECYEMHQPDKNRKEDMKLAMMAAGGGSTGALSTAKTFAMAGGSFLQGVVKKEKNEFAEQYMDVGFSLYLLLNRMHDTDSKLIENIKLQPLQQKAFNFYKKNSMSIEIVKEDDLQKINFRVKNKRVLREEVKEKLKWGVNRSSPSNKIRDLMAWTKDIMKDIAYQQRILSNFIAICLTKGWLFWNHGVTIISFAINILMLVTWKAKGSLETPGIMDNATGIPHAIIDPVPSIINLGQSSYEITIKTLGGAHNFFSLLVLISYFVCNHPRLPDFKSIMTSCKSKFKKKSDDDEIDKKKEHQSKLDAQFFSFTTFYYMVFLALSIGGTFTSGYFFAFHLLNIVNNNQLLSGVIKAVTQNGKSLLWVAVLGLVVFYLYGIIGFALMRSMFDPKDYLYCNTLWQCTITVVRYGLIGDLFEVMNPHDNEKTFEKFGVVVLYHVSFFIFITTIGLNIIFGIIVDTFSELRDLKWTAESDMRDICFICSRNSYDFEHHGKGFDHHVRYEHNMWAYIFFFIHLNGTKVNDYTALEMYVYKLLQKENFDFFPLDRALSLSSMGKDSTETKLDDLLYQVTTIVEKQRVEELEKKRREERMRQKIWEQKHRLGSFRRKARLPGPTEPDPLMQLQGASGQLDESNRLLQLSPLAQLAQLGQQVARRASMGGEHRADMGPTLRQHSIASSHGRHGRGGGGGGSPLRHDSTEQRYFRGSSPSRYDEEPFPRNLSPVRYDDRTRSPSRSRHGSEPDLMGLSPVHSRREPSRDYGMLSPKWVESRATSPRGASIFFPDSDSLSVHSLRDAPPSGQLPDPYLDSIETGPFLIDPPEPQRSTSRDTESAESGNSGDFTRLETRL

>Lgig_138356_138352

LDDIVRVVTHKDCIPEVKSAYINFLNHCYVDTEVEMKELFNSRHMWTLFENFLVDMAIVCNTPCDPNHSDQMMHDYVTITVMNIISTYFSSSYSDQSTSIQTNQPVFVRLLQGAFRLSHCDWLSGTQRFNVENCIKTLSDTAKSRGIAIPVDLDSQVNSLFEKSQIVLKTTNTWMKKRSNRIDSTSSTQRDYRNIIEGLQDIVSTLEDQLRPLVQAELSVLVDVLHRPELLFLPGTDARKKCESGGFISRLIKHTKTLLEEKEEKLCIKVLQTLKEMMTIDIDYSDKTVLVDKTVLVDKTVLGDKTVLVNKILLVDKTVLVDKTVLVDKTVLGDKTVLCHLDREGASDLVVDLIIENTSNRIFQETVELGIALLEGGNSIIQTSLYSRLSHDKSSETFYRTFYDRMKEAQLEIKNTVTVNTSDTTSQKLEEENLSASKNIKKGRSDAVISEGLRSQFDDASNTAGRTMGQMRQGHGHGEDSENNTPVTPPDDVTDHRPMRDKDEKKLSAQIGMMQPILRLLQLLCENHNMDLQVDNKTSYNLVCETLQFLDCICGSTTGGLGLLGLYININNVGLINQALTSLTEYCQGPCHINQNSIAMHESNGIDIIIALLLNDINPLGKQRMDLVLELKNNASKLLLAIMESRHDSENAERILSNMSPKQLVDVAKQAFHSEDTVEDDEEDDEEREASPKAVGHNIFILAHQLSQHNKELAELLKPSGTDLYGDQALEFYAKHTAQIEIVRLDRTMERIVFPIPEICEYLTEETKVKVYNSAERDEQGSKVSDFFERHEDMLREMQWQKKLRANPLLFWFSSHMLLWGSMSFNFTVIINLLVAFFYPFSNSKKELDPRLSGLVWTAMLVSLAFVITLPRPLGIRTLIVSTILRLIFSVGLEPTLWLLGVLNVINKAIFLTSLMGNRGTLTKPFRYIITDFEFMYNSIYLTMCTLGLCVHEFFYSLLLLDVVYREETLVNVIRSVTRNGRSIILTAVLAVILIYLFSIVGFIFFQDDFLMEVEMLPVEDIVKGHIRSYTCKVSSRVNETINQGLVNIITLSDGVEEDGKQRACDSLIMCILTSLNQGLRNGGGIGDVLRKPSSREPLFVLRVIYDLLFFFIVIIIVLNLIFGVIIDTFADLRSEKQQKEEI

>Lgig_168083

IHNEERKLPEDWCSRTSESKIKKQMNHIKDVQIAINNHNVINRVLPHLARRNDEIAREVLAFICIMLFNANREVQKSMLDYFLSTREEVFFMAVRDRMQISTNAIKEKRSLLAQHEARVKEALANMTNYSTTLTVGRKALQQIQVYEQKMRVERLGGWRALCRAIEPKDEKGLKRKLTWKRKSSRDKKPQPKQKPKKLSNGCPKDSEALMNEQSELELADVKVEEAKPEEEVVASMDEGVKDMLEYKDDGYIELVLKLMARICDGQNTGLQDYLREQPDNVKSFNIVGDTSQFLNVVYTTINNKTIDLVIQTFSTLNEFCSGNQENRAVIYDKKVIDYINFILRAGEIADCPVDKVIELQQIISSLITSLIEENGPGASQVAKAVKDALDKEAVYRCMTACYEMHQAEKPKLEGLMKGAEAGSYLQSAKSIAAFGGSLIQGVVKGKKRSALKETVMEVGFGFYMILARMIDIDPLLNLQLTLSPENLKAFDYYKKNSCSIEIVKEDVLQKVNFRVKNKSVVRDEVKEKLKWNVDRTSPSNKIRDLMQWTHDIMKDITYQKKILDNPIAKMFTKGWLVWNYGSILLSLAINIIMLVTWNAKASMEDCAAVFANDAANASILCPGIYDPVPAIIGIPQNQYAITLWALGGVHNLFSLFVLISYFLSNHPRMPNIAEIKAGARKLCGRSYPDDEELMKKTEKVSNLNVNLFSFTTFYYLLFLGMSIAGTLFHGYFFAFHLLNIVNNNQLLSGVIKAVTVNGMSLLWVAVLGFVVIYIYSLVGFALLRSYFSASDYLYCSTLWECTVTVIRYGLIGDMFEEIKQNPQGSSFGSFWPLVIYHVTFFIFITTIGLNIIFGIIVDTFSEMRDLKWRAESDMKDTCFICSRNSYDFEHHGRGFDYHVKNEHNMWSYVYFIIHLDDIKESDYTALELYVSKHVENESYDFFPLNQALCLTSIDIDSTETKIDDLLHQVTAIAKKQKEDEAEKKRKVEKLKQKRWQEKHRRNFFGYNQEYDDTQPLIPRAISDDRLSREPSASRDVPMILRRPADPRTASKLLSPPTDDSFQLRRRHLPPKLPHSASQEEGPEEVVSDSDTDSFQREGSYDLLDDDNDSLPPPPPPLPQPPPPLNLPSSPLARKPELPPELTDDGGDVGNIRSTDL

>Tcas_IP3R

EDVLVFYNDKASFNHFIEMMRSERHCMDEGSPLKYHVELVKLLACCTMGKNVYTEIKCHSLLPLDDIVAMVTHPDCIPEVKEAYVGFLNHCYIDTEVEMKEIYTSNHMWTLFEKSFLVDMADIATAPTDRKHPDKALENYVTNCVMNIISTFFSSPFSDQSTTVQTRQPIFVQLLQSAFRVSQCPWLSASQRFNVENCIRTLSDVAKNRGIAIPLDLESQVAAMFNKATILTRQTSKWLQASKTPKMERSQSQLMRLDRSIIEGLQDIVSLLEDQLKPLVQSELSLLVDVLYRPELLFPPGTESRKKCESGGFIRRLIKHTETLLEEKKEKLCVKVLKTLREMMAIDPEYGEKGDVLRNSLLARYFGKAFVQKHDTVELGSYQISSVMHGPGAKVLRRAGRTLHEVQTHLDREGASDLVVELVIKSVNSPSIFGEAVELGIALLEGGNPIIQKSMFNKLLGGDLSQAFFRVFYDKMKGSQQEIKSTVSVNTSDIAAKAHEDKQDGKDLDKVSKKQGGKSNGILISDELREELSRAAESTSQAYAQIRNMAVGEDGTVTSGTASAFEDLLAEKLEKHRDREDQNSLSNKVLIMQPILRSLQLLCENHNPALQNLLRNQNNKTNYNLVSETLMFLDCICGSTTGGLGLLGLYINENNVSLINQTLETLTEYCQGPCHENQNCIATHESNGLDIITALILNDINPLGKSRMDLVLELKNNASKLLLAIMESRGDSENAERILYNMNPKQLVDVACRAFHQETAEDDEEVDDGSVEDAVSPKEVGHNIYILCHQLAQHNKELAALLKPADFGSDPKTHKALIYYATHTAQIEIVRHDRTLEQIVFPIPEICEYITTDTKIKVFNTAERDDQGSKVVDFFERTESMFNEMKWQKKLRAQPVLFWVSSYMSLWSNILFNCAVLINLIVAFFYPFEDTLPKISSSISVLIWVAMLASTAIVVTLPRETGIRTLVASTILRLIFSLGPEPTLWLLGTVTILLKGIHLVSIMGNHGTLTKTYQQILTDAELVYHLAYLVFCMLGLLMHPFFYSVLLFDVVYREETLLNVIRSVTRNGRSIILTAVLALILVYMFSIIGYMFFKDDFIVSVKKKTDETICETVSDTSGNKYTHDNAQYCKIVDPGEEKKERACDSLIMCIVTTLNQGLRNGGGIGDILRAPSSEEALFVARVVYDLLFFFVVIIIVLNLIFGVIIDTFADLRSEKQQKELILKNTCFICGLNRSAFDNKTVSFEEHIKCEHNMWHYLYFIVLTKVKDPTEFTGPESYVYAMVKASNLDWFPRLRAMSLAAVEGEGEQIELRSLQAQLENTQVLVTNLSQQLMELKDQMAEQRKQKQRIGLLNSASAYLQTPNLL

>Tcas_RyaR

LTQLVTTFCRGAMTERSGALQEDPLYMSYAEIIAKSCGEEEEEGEEEESGEGEEGGASIHEQEMEKQKLLFNQARLAKRGVAEMVLLHISACKGVPSEMVMKTLELGISILRGGNFDIQMGMLNHLKEKKDVGFFTSIAGLMNSCSVLDLDAFERNTKAEGLGVGSEGAAGEKNMHDAEFTCALFRFIQLTCEGHNLEWQNYLRTQAGNTTTVNVVICTVDYLLRLQESIMDFYWHYSSKDLIDPAGKANFFKAIGVASQVFNTLTEVIQGPCTLNQQALAHSRLWDAVGGFLFLFSHMQDKLSKHSSQVDLLKELLNLQKDMITMMLSMLEGNVVNGTIGKQMVDTLVESASNVELILKYFDMFLKLKDLTSSPSFQEIDINNDGWVSPKDFKEKMEQQKSYTSEEIDFLLACCEVNHDGKVDYIGFIDRFHEPAKEIGFNLAVLLTNLSEHMPNEPRLARFLETAGSVLNYFEPFLGRIEILGGSKRIERVYFEIKESNIEQWEKPQIEESKRAFFYSIVTEGGDKEKLEAFINFCEDAIFEMQHASGLMAVEESGGGGPTRAASYSYMNVDDEDRGKDPIRRGYQSIKEGIAYGLSSLSPSNIKHKFAEMQQMTIPELFIGFFRMIIYAFYYSGFGVAIIFKYIFGVLLSLMRGPIEEPAVLEIKEEEEKVGLSRVLPALPSSEEQNTQMQAFGLDITKEDNGQYKMAAHESPTTSQPSSAEGEGETTPEEDGEHPETETMEAPLSLSDLLGGEQAKRAAQEKVEAQAAQQAVMQAIESESKQPVVTEPSAVSQINFGAYSHRAVSFLARNFYNLKYVALVLAFCINFMLLFYRVSTLGDDAEGSGSKGIPDILEGSAEGSGAGSGESGEDDDPPELVHVDEDFYYMAHVMRLAAILHSVVSLAMLIAYYNLKVPLAIFKREKEIARRLEFEGLYIAEQPEDGDLKSHWDKLVISAKSFPVNYWDKFIKKKVRQKYSETYDFDSISNLLGMEKTSFQQQESDEGRSFIYFIINIDWRYQIWKSGVAITDNSFLYSLWYFIFSILGNFNNFFFAAHLLDVAVGFKTLRTILQSVTHNGNQLVLTVMLLTIVVYIYTVIAFNFFRKFYVQEEDEEVDKKCHDMLTCFVFHLYKGVRAGGGIGDEIEPPDGDDYEVYRIMFDITFFFFVIVILLAIIQGLIIDAFGELRDQLESVKEDMESNCFICGMGKEYFDKVPHGFDTHVQQEHNLANYMFFLMHLINKPDTEYTGQETYVWNMYQQRCWDFFPVGDCFRKQYEEELGGGGG

>Lpol_IP3Rt1

SLDDIVRVVTHPDCLPEVKGAYINFLSHCFIDTEVEMKEIYTSNHIWTLFENFLIDMAMVCNAPSHDRRRADYQLEKYVLISVMNIITTFFNSPFSDQSTTVQTHQPVFVRLLQGAFRLSNCSWLKLHKRMNVETCIKTLSDIARNRGIAIPVDLDTQVSSMFSKNSVISKHTRAWLSASRTPRREHSSSSLYRNERSIIEGLQGEALRQTLLIRYFGKTNMKCGVRVSLNKPIVARNIASTGVVTHGPGGVLLSRAEMTLAEVQCHLDREGASNLVVELIMVNPNHSIFLESVELGIALLEGGNSVIQKSIYQKLTSGNNSEKFFKVFFDKMKEAQQEIKSTVTVNTADLASRSESERDSLSKEVSKDGRKMKAFKQNSVVLTQDLKEQLEDAAAATSKAYAQVRQLGCSATSSPDQDLMASGPAPHTYAALEEILAEKAEKAKEESRLPPEVAVMEPILRFLQLLCENHNAELQNYLRHQSNKNNCNLVSESLMFLDCICGSTTGGLGLLGLYINEYNVSLVNQTLETLTEYCQGPCHENQNCIAVHESNGIDIIIALILNDINPLGKKRMDLVLELKNNASKLLLAVMESRGDSENAEKILYNMSPKQLVEVACNAYHQEGDEDDEEEVEEFLQGSDGVSPKEVGHNIYILCHQLAQHNKDLAAMLKPSLSGMESKTNLALQYYASHTAQIEIVRHDRTMEQIVFPVPQICEFLTRESKVKVYNTTERDEQGSKVSDFFERTEDLFSEMKWQKKLRGQPLLYWVSRHMSDWSTVAFNLAVLVNLIVAFFYPFPEKTEISLQWSGLIWAVMLISLATIVTVPRASGIRTFIVATILRLVYSVGLQPSLWLLGAVNLVITSVHLVSIMGNHGTFNKSFRHILADFELLYHLGYFVFCVLGLCMHPFFYSVLLLDVVYQEETLLNVIKSVTRNGRSIILTAIFALILVYLFSIIGYLFFRDDFLMEVDNQQLVNPVDYKGARGISPSSKISLAERTLMRTAEVTTPSGCLWKDKKSCSCSRDILFWMYNTKDSKDAVKPQSEPVISETARPDASNKKESQKERACDYLIMCIVTTLNQGLRNGGGIGDVLRTPSAEEPLFAARVVYDMLFFFIVIVIVLNLIFGVIIDTFADLRSEKQQKEEILKNTCFICGLNRSAFDNKSVSFEEHIRCEHNLWHYLYFTVLVKVKDPTEFTGPESYVAAMIKDRNLDWFPRMRAMSLAANEAEGEQNEIRTLQAQLQNTQKLVSGLSQQLNELKEQVCNLYQMPSIIFFSMCVSVPMHIPVSLVGNLIPNVIQ

>Lpol_IP3R

YVTSSVMNIITTFFSSPFSDQSTTVQTHQPVFVRLLQGAFRLSNCSWLNLQQRMNVENCIKTLSDTAKNRGIAIPVDLDTQVASMFNKNAVIGKHTRAWLSAARGPRRDPSSSSLFRNERSIIEGLQDIVSLLEDQLHPLVQSELSVLVDVLHRPELLFPPRTEAYKKCANGGFISKLIAHTERLLEEKEEKLCIRVLQTLKEMMSLDPDYGEKIDQKLLSRREQEDQARGERLRQTLLVRYFGKSPQKKDTNSGQNKQQTTTRASVSSSSLHAPGGLMLSRAEMTLAEVQCHLDKEGASNLVIELIMRNPSQAIFVESVELGIALLEGGNPVIQKSMLYKLTQGNISEKFFKVFFDKMKESQQEIKATVTVNTAEIAARTGEDRELLGKDLGKDGRRVRALKQNGVVMTQELKQQLEDAAEATSKAYDQVRQLGTSQNLASDGDLLSPGSAPGAYAALEEILAEKAEKAKDAEESKLPAEVTVMGPILRYLQLLCENHNLELQNYLRNQNNKNNYNLVSETLMFLDCMCGSTTGGLGLLGLYINEHNVSLVNQTLETLTEYCQGPCHENQNCIAMHESNGIDIIIALILNDINPLGKKRMDLVLELKNNASKLLLAIMESRADSENAERILFNMSPKQLVDVACNAYHQEDITEEEEEGEGDDNDDDGVSPKEVGHNLYILCHQLAQHNKELAAMLKPSPNDTESKNNQALQYYASHTAQIEIVRHDRTMEQIVFPVPQICEFLTKESKLDVFHTAERDEQGSKVSDFFERSEDLFSEMKWQKKLRGQPLLFWVSRHMSIWSTVAFNLAVLINLIVAFFYPFSDPNELGPRASGLIWAAMFISLATVVTLPRPSGIRTFVGAVILRMIYSLGLQPTLWLLGTVNMIMSSVHLVSIMGNNGTFNKTFRQILTDIELLYHVTYLFFCVLGLCMHPFFYGVLLLDVVFQEETLLNVIKSVTRNGRSIILTAVLALILVYLFSIIGYIFFRDDFLMEVDERIVPEPKPKLPEFVAKHNPPVEVADLLPKPEFCSSEEVTNCDKNTTPRDNKYTAHNNLSDHLPVETNNSSVLDVLEEDSLQEPEVEGGKERACDSMIMCIVTTLNQGLRNGGGIGDVLRAPSAKEPLFAARVVYDMLYFFVVIIIVLNLIFGVIIDTFADLRSEKQQKEEIMKNTCFICGLERASFDNRTVSFEEHIHCEHNMWHYLYFIVLVKVKDSTEFTGPESYVAAMIKDRNLDWFPRMRAMSLTADDGEGEQNEMRTLQAQLDLTQKLVATLSQQLTELRDQMTEQRKQKQRLGLLGAPPIGALPIHTVSSQ

>Lpol_RyaR

TMYREIWLEEENNGHEQLIEDLTQSFEEAEEKRGQVEEEDKPDPLAQLIVAFSRNATTEHSGTITEDDLYMQYAIILGESCGGAEEEEEEEGGDDEAGPSIHKKIGLMMEQEMEKQKTLFQQARLADRGVAEMVLLYISACKGIQGPMVINSLKLGISLLRGGNIDVQMRMLKHLKEKKDVGFFTSIAGLMNSCSVLDLDAFERTQKAEGLGVGSEGTAGEKNMHDAEFTCKLFRFLQLLCEGHNLEFQNYLRTQAGNTTTVNIVISTVDYLLRLQESVMDFYWHYSSKEVIDQPGKDNFCKAITVAKQVFNTLTEFIQGPCPLNQQALAHSRLWDAVGGFMFLFSHMQDKLSKSASQLDLLTELLELQKEMVIMMLSMLEGNVMNGTIGKQMVDTLVESASNVEMILKFFDMFLKLQELTSSSAFQEIDTKRNGWITPKEFQRAMEQQKIYTQEEISYLMSCCEPNHDGLIDYREFTERFHNPAKDIGFNLAVLLTNLSEHMPNDPRLARFLETASSVLNYFEAYLGRIEIMGGSHRIERVYFEVKESTIKQWEKPQIKESKRAFFYSIVTEGGDKEKLEAFVNFCEDAIFEMQHASSISMEDTDEGPTKTSDYPFPGSEQEKPPSALEPVKQGLRYIKDGILKALSYLAPANLKKQYAKLRQKTYLELLVGFFKLSFYSVFSSLQFVYCITRFIIKFIIMLMGGQPIGQPVEEGKAEEHNRKPVFMVPMAVPLPIEAPPETSKTGDHTDMPGTIHAFGLDITKEEEGGEVRYHMAPTQAHQEVTPQSSVEFSEGSEEEAGKNQHPEKPLDGQVISDGVTSPVNHMDGDVKTKETLHASQETKLGTDSATQASIPGVQDNVLPPEMSASMAPEPQKPVEPPPVAAIDLSEYTNRFVSFLARNFYTMKYIALVLAFCINCILLFYKVTTDEVEDVIADKPEITEVLNNITEELLGGDNDLGGGEEDEEDVEEWLIIEEKFFYLEHIIRLFAFLHSSVAFCMLIAYYHLKVPLAIFKREKELARRVEFDGLFISEEPAEDDIRSHWDKLVISSSSFPVNYWDKFVKKRVREKYSETYDYDAISNLLGIDRSGSFTQDKGLGGFLSFIIDVDWKYQIWKAGVTITDNSFLYNLWYFAFSVMGNFNYFFFAAHLLDVAVGFKTLRTILQSVTHNGKQLVLTVMLLTIIVYIYTVIAFNFFRKFYVQEEEDQVDQKCHNMLTCFVFHLYKGVRAGGGIGDEIEPPDGDDYEVYRIVFDITFFFFVIVILLAIIQGLIIDAFGELRDQLQSVVDDMESNCFICGIGKDYFDKIPHGFDTHVQKEHNLANYMFFLMHLINKPDTEYTGQETYVWELYQKRCWDFFPVGDCFRKQYEDELMGGAG

>Skow_IP3Rt3

VFPIIQIFFKQICHLDVYDHRNEQEVVNNLAEVIMEFVRTTVRISMITNMTNFNILMSASRVLLDKSELDNKLAVKGELARYANQTFDTRSVAQQKYQDTYREEEHINHCLNDYANNLRLSYIGENTVDAQLKTNNNNTREYDKGGEEELPLGAEFQEHIQCFMHKTTSDNSLAHSALKNKSRKPKYQRAKKLMEGLMISCNNPKSTEQERLKQEALDIKSLQILRAMIHNEERQLPHNWKENPKKHTDRLMKIESVQNELDNHGAMLQILPLLGRTRERLSHEVLAFLSAMLFNGNVQVQTSLENYFRRSREEHFFMAARNLMHISKISIKEKRALFSQLEARNKMEEDLRMQSNTATSITNSTDKPANGLRISQNSTEGNAMKMDTINSSEVSPDIGDEKNQPKREKMNYFKKDGLIELLFRTLGQMCDGQHTGLQNYLRDQPDNMTSVNIVAETTMFLNILYANFDDTNIALLVELFTTLNEFASGNLANSMVMFDNKIIELINFILRDTNIENRISEHKQLIHELKLVMANLVMSMIEEQSREGSEFANEIMDKIDNDVIHEIMVDYYKKSFAVNTNESDKNCYTQIGFAYFHILMRLQDIDATISKERYILHELKDTDDEHSPTMNTHNQEVIRAWNFYEANSMSVEILRNGDVQKVHFRVKNTNLLRTEVKEKMKWNIKRESQIEKIQDFVHWSQDIVKDINYQNKVLSYRPAKFFILLTVKPILTASYWGAILYILFGLHNVLSACLLVSFFLSNRPRLPSGTDIQNDFRSLIGQEVSRVRQSYLDAKFFSQITFYYIVFFVFSICGTIFYGYFFCFHLLHIVVMNQLLKRVLLAVTLNGKSLLYVFLLGIIVIYIYSIAAFGFFRYDINDEETEMFFCNTTFECFISTARYGLLESFHESIGIPASTDFPTFTTRSIFDVSFYIVVVLIGLNIVFGIIVDTFSELRDSKWRTDEDMRTLCFICGRKSYDFENHGNGFRQHVKLEHNVWSYLFFFIHLNETATNDLTALELHVLNMLEVPDYGFFPVNRALCLNNIETLQH

>Skow_PI3Rt3

EVNDLLNLFANNYSLSYGGENTVSVQLKKDSDNTREYTEIGGDEELPLGEEFQEHVNCFIYKSSHNSLPIQNLKKKKKKQTESKYELASKLVEELLISGNNPRSTERERLLQEALDVKCLQLLRALIHNEERQLPEDWLNDPKNNEDQLEKIVAVQKELNEHGAMLKVLPLLARTSDSLAREVLAFLSAMLFGGNEDVQSSLIDYFLSTREEHFFMAIKNRMNLSAVATKEKRALQSQHRAKIDEALEQQKSLNKALRHGKVIAETILAGSMMMKRSTAGSKGRLGSRFGSKRGKGGMVGSQRSMNRQKFLQGSRRTLRPQNKVNDIDTAISAGTSGAANGSKKALNSKESIEMGGINNDKVPLIKVDKHDKGASLKDLGDIEFEDLPPEVQKLIEEAEADDLEFKDDGFIELVLRILGQMCDGQHSYLQNYLREQPDNIKSVNLVAETARFLDLLYSNIDDSSIALVEELFTTLNEFASGNHANAMVIFDNKIIEYINYILRDTNMASKIVLEDNKQFLHQLRLSIANLIMSMIEENSAESTLLAEEVKETIDEDAIHEIMLDYYRKAKAASDKSTREDYTVTGFAYYHILMRLQDIDPQINKENLVKVKDDDPSYSIECDKGWEFYAANSMSVELLKDGEVQKVHFRVNDTTVLREEVKEKVKWNINRESQSDKIVDFVDWSKDIMSDIKYQTKVLSYKPAKFFITLTPLWNWSLLLLSFAINIIIVATWIAPADTKTHLFGKEVHEERQSNLETKFFSFTTFYYIVFFGFSIAGTFLYGYFFCFHLLHIVIMNQLLNRVLLAVTLNGKSLLWVFLLGFIIIYIYAIVGFAVLRSHFDTDSGLYCETMFQCLVTSVRFGLIDGLQEYLAIPDTENTFLAYALRAIYDITFYILIITIGLNIIFGIIVDTFSELRDAKWRTDEDMRTLCFICGRGSYDFEHHGKGFRHHVKFEHNMWSYLFFFIHLDDTRSNDYTAIELYVSNMLKEENHEFFPVNRALCLSNDDEDYDTKIDMLMARVDQMIRRNREEDANRRRDELRQKQIQWEKEHRG

>Skow_RyaR2

IQGPCTLNQLGIAHSRLWDAIGGFLHVFANLQKKLSQDISQLELLRELLNLQKEMVIMLLSLLEGNVMNGTIGKQMFDALVESSSYLEMILNFFDMFLKVKDITCSDAFLEMDVNGDGWVSAKEFRDAMEAQKMYTTEEIEYMLSCADTNQDGKLDYAEFTERFHNPAKEIGFNLAVLLTNLSEHMSNDPRLGRLIGLAGSFLSHFEPNLGRIEIMGSAKRIERVYFEIKESNIEQWEKPQIKESKRQFLHDIVNEGGEKEKLENFVNFCEDTIFEMQLATSISGEEMGLVEALTKAVFPGGTSESRSWLIHVARAFKPMNIYRSIKDFITTTQWGVKQIRSMSLAQLFFALIKLVFIIGWKFIGLFFHLACKIVGMLVGSRIIEDAKELSGSISETLHVPSIARQANRDLKPSYLAALPGIRSGQSVSAFGIDFIKKGEDHDEAANAGADSMRMRLRRRRGMTPTSSIDLEDEDEPQTEIPTVNESNGGIVTENGAPTGMTGERRQSSVFPSLFLLPDTEKEKKEEVKEEVVEHKLSQAKQAFVSMFARNYYNLKYIALVLAFIINFILLFFKAGSTASELFAVEPDIDEINATKNGDDDDTWDELISLEEHVVSYLEPLLQLFGFLHTLSALSMLIAYCALKVPLALFKREKEVARKLEFDGMWITDEPESGDIQAMWDRLVINTRSFPVNYWDKLIKRKVRQKYSESLDDDKLCELLGMEKQDNLFSESKEEEHESILTAILRSVDWKYNLWKCGVICTDNSFLYIALYLTFSILGHVNPFFFAAHLLDVAMGFKTLRTVLQSVTHNGKQLILTVMMTCIVIWLYTVIAFNFFRKFYLKGEDDEVEFKCHNMLTCFVFHLHTGLRAGGGIADEIEAPDGDPYEFYRIIFDITFFFFVIVILLAIIQGLIIDAFGELRDQIEQVKEDMESKCFICGIGKEYFDREPHGFEKHVMKEHDFANYMFFLMHLINKPETEYTGQESYVWQLYQERCWEFFPIGNCFRKQYEEE

**Amino Acid Decarboxylases (Figure 7)**

>XP_009028665.1 hypothetical protein HELRODRAFT_186120 [Helobdella robusta]

MDANELNDKGKKMIDFVSSYWQTIRSRRALSVVEPGYLDTLLPNEAPLKAELWEDVLRDVEQLIMPGITHWHSPHFHAYF

PTGNSYPAICADILSDAIGCIGFSWISSPACTELEMKMMKWLGKLINLPSQFYTPTNAGGVIQGTASEATLVSLLAARSK

VIKDKNLSFQDINSTKMVVYCSDQAHSSVERAVMLLLMQCRKILSDSEHRMNVKALSEAIKKDLEEGLTPTMCVATLGTT

STCSFDDLTEIGKICNEQNVWMHVDAAYAGASFICPEYQYLMEGVQYADTFNFNPHKWMLINFDCSALWFKDSSFMVEAF

NVDPVYLQHVNQKVVLDYRHWQVPLGRRFRSLKLWFTMRLLGVEQLQNHIRKSIKLAKDFEKLVCQNPDFEVVNNVILGL

VCFRMKGDNSRSKKLLEKITADGKIYLVPSEINGVYFIRFAICSWFTNEGDVNEAWSTIESLSKDISMQETF

>XP_009023368.1 hypothetical protein HELRODRAFT_101612 [Helobdella robusta]

MNSSEFRQRGREMVDYIADYLENIETRRVLPEVQPGYLRDMVPKEAPYDPDSWEEVMEDVERCIMPGITHWQHPRFHAYF

PAGNSYPSILGDMLSDSIACVGFSWAASPACTELEILMLDWLAKMINLPQKFLHVSGAGGGVLQGSASDCVLVSMLVARN

KALTDLSSSFKEQPEGVLLSKLVAYCSKLAHSCVEKAGMLSLVKMRELDIDENYSLRGGTLEKAIEDDRNEGLIPFYLCA

TLGTTAVCSYDNIKELGEICEKQKIWMHVDAAYAGSAFICPEFRNKMVGIEYARSFNMNPNKWLLVNFDCSALWVEDRKE

MTNALTVDPLYLQHSQSDKAVDFRHWGIPLSRRFRSLKLWFVIRNYGVSGLQSYIRNHCTLAKLFESLVLTDGRFEVLGE

VSLGLVCFRLKGHNFYTQMLMRSINLSGKLHMVPALINDSYVIRFAVCHQRAKEADIKYAWCVVLEMAAIVLETCKENFI

EVAKVFEKIASLEIDDADEAKGDESQHSTKKNSIKKKKSDVKSSSQKFSNGDKPESNNCGTQINTKDSEKKLTLDFDGLG

NTNSNETFEETDDNDDDEVFPFDDNIPSIPSIRSLPEPAAATADATAVNSNASSSNKHGKPLASYKRRNMLLRMISDPKL

YKPQLLRSMSIERKKKMKVESSCTEELHSCSVESCDSRESCQEFATDSTGPNRLSSSYSQTNNNQEFESSKDKKKN

>XP_009023318.1 hypothetical protein HELRODRAFT_84403 [Helobdella robusta]

MDSKEFRRRGREMVDYIADYFENIEQRRVFPDVQPGYLKELVPSEPPQHPDSWDNIMNDITRCILPGITNWQHPHFHAYF

PSNNSYPSMLAEFLVDAFGAVCFSWASCPASTELEVIVMDWIANMIALPKKFLHSNGVGGGVTQSTASECILISMLVARH

KALKNLSHLHQNLEPGVLLDKLVAYRSKYAHSCAEKTGMLSLVKMRELDVDEKFSLRGNTLEKAIQEDKRNGLVPFYVFA

TLGTTSVVSFDNIKEIGEVCSREKIWLHVDAAYAGSAFVCPEFRVYMEGIENVQSFDLNPQKWMLMNAECSALWIDDKEA

LIDAMTVDPLYLQHNHSDKTEDFRHWSVTLGRRFRSLKMWAVLRSYGVTGLQNHIKNHCKMAKYFETLMLTDARFQIVGQ

VTLGLVCFKLRDNQLTEKLLSSLNSSGKMHLVPAAVNDDYIIRFAVCFQKINEDHIRKSWEIIRDTATKILNDTE

>XP_009023334.1 hypothetical protein HELRODRAFT_84539 [Helobdella robusta]

MDSKEFRQRGREMVDYIADYFENIEQRRVFPDVQPGYLKELVPSEPPQCPDSWDNIMKDIDKAILPGVTHWQHPHFHAFY

PWTQSYPSILADFLVDALSSAGFSWASCPASTELEIAVMDWLANMLGLPEKFHHCRGAGGGVTQNSAGECLIVSMLAARH

NALKHLSSIYPNEDAAVLLSKLVIYHSKLAHSCVEKAGMVLLIKTKGLDFDDKHSLRGHTLETAIEDDERKGLYPFFVLA

TLGTTPVGSFDNIQEIGEVCNRRRIWLHVDAAYAGSSFICPEMRTQMNGIKNVNSLNINLHKWMLMNSESSAIWIDDKKV

LIETMAIEPLYLQHHLSENFLSSKKNWSISLCRKFRSLKIWAVIRSYGISGLQRHIRNHCKLAKYFESLMLSDSRFQVIG

TVNLGLVCFKLKNNHLTEKLLSTLNSSGKMYLVPAAVNEDFIIRYVICYEKMNERLIEESWNIIRNLASEILNQNYEEK

>XP_009031618.1 hypothetical protein HELRODRAFT_156213 [Helobdella robusta]

MTQILDPVFEITDEPESLEELLKSVKLLLKYSTKVGHRRKLNNPSTSFDVISLAGEMMTSTTNTNMFTYEIAPVYTLIEG

LVLDQMRTMVGWPPSQGDGIFAPGASMANMYAICVARHKFIPSFKNLGMVEQRKDLIVFTTSQCNPSTPRSAAIIGVGLK

NVIFVDCNERGQMSVTDLERKIVSSLAAGSLPFFVSATSGSAMLGSFDSLTEIADVCDKYNLWLHVDASHGGGVMFSANH

IHLVRGIRRAHSVTWNCHMMMNCTLQCSAILIREKGLLEACNSTCATYLFQQDKHYDVAYDTGDKAIQCGRHNDVYKLWL

LWRSKGHTGMSDHVVRLFKMAAVFQEKLKSTPGVCMVLPEMEFVTICFWYVPEKMLKMTLKIPLNMLKFDWLEGNNNNNN

YKNINNNDDNDNNNNDWLPTFYKVVINNSATTSRDIDYVINQMKRLADEEVNTLMMMK

>XP_009011926.1 hypothetical protein HELRODRAFT_73271, partial [Helobdella robusta]

EITQNFLLHLTNILVGYIHASNNETCKVLEFHHPRLLLGRLKKVLFLESQPQNLSQTLKNCQQILKYCVKTGHPKFFNQI

SQGLDVVGLAGEWITAVTNVSIACQIEAPVYVYMEEVVLRKMCSLIGWKNGAGILTPGGTMSNLYSVSFCMQFIFPKLKF

NGLSNEKPLVLFTSDQSHFSIKRAGMLLGIGRENVVLIKSDDRGKMMPGDLRLKILSCIEANKKAFLVSATSGTTVLGAF

DPLHEIANLCEEFGLWFHVDAAWGGGLLLSKTHRHLLHGIHRLAHSVTWNPHKLMGIPLQCSAFLVREKNFLMGCNSVTR

DLGILAGDEFLDDRTMQASRHNDILKLWLSWKAKGSRLLENQIDSLMILSRYMQGQISVRPGFELVLEEPEFLNICFWYV

PQRLRHLPPGAERDDKLSTIAPKVKAVMMEKGKTMIGYQPLGDKPNFFRMIISNPASSEEDIDYLLDQIQLYGEL

>ELU12210.1 hypothetical protein CAPTEDRAFT_158583 [Capitella teleta]

MDAKEFRRRGKEMVDYVADYLENIQTRRPLSDVQPGYLRQLIPGEAPQDPESWDQLFPDIERVIMPGVTHWHSPHFHAYF

PTSNSYPAICADILSDAIGCIGFSWIASPACTELEVVMMDWLAKMLELPDQFLSGGKGGGVIHGTASEATLVALLAARSH

AVNKAKECHPDMTDAQILGKLVAYTSDQAHSSVERACLLGAVKCRLVKSDENEKMRGDALQEAIEEDKQNGLIPFFCVAT

LGTTGCLSFDPLIEIGPVCQKENVYMHVDAAYAGSSFICEEFRPLLNGVEFADSFNFNPHKWMLVNFDCSAMWFKDSSDV

VDAFNVDPLYLKHENQGAVPDYRHWQIPLGRRFRSLKIWFVLRLYGVKGIQDHIRKHVRLAHEFEELVKKNSAFEVTHEV

TLGLVCFRLKADRATNETLLQNINKDGRIHMVPSESKGKYFLRFAVCAASTESKDITFAWEVIQELADQLM

>AAO16835.1 dopa decarboxylase 56.7 kDa isoform [Drosophila melanogaster]

MSHIPISNTIPTKQTDGNGKANISPDKLDPKVSIDMEAPEFKDFAKTMVDFIAEYLENIRERRVLPEVKPGYLKPLIPDA

APEKPEKWQDVMQDIERVIMPGVTHWHSPKFHAYFPTANSYPAIVADMLSGAIACIGFTWIASPACTELEVVMMDWLGKM

LELPAEFLACSGGKGGGVIQGTASESTLVALLGAKAKKLKEVKELHPEWDEHTILGKLVGYCSDQAHSSVERAGLLGGVK

LRSVQSENHRMRGAALEKAIEQDMAEGLIPFYAVVTLGTTNSCAFDYLDECGPVGNKHNLWIHVDAAYAGSAFICPEYRH

LMKGIESADSFNFNPHKWMLVNFDCSAMWLKDPSWVVNAFNVDPLYLKHDMQGSAPDYRHWQIPLGRRFRALKLWFVLRL

YGVENLQAHIRRHCNFAKQFGDLCVADSRFELAAEINMGLVCFRLKGSNERNEALLKRINGRGHIHLVPAKIKDVYFLRM

AICSRFTQSEDMEYSWKEVSAAADEMEQEQ

>NP_001177377.1 aromatic-L-amino-acid decarboxylase [Mus musculus]

MDSREFRRRGKEMVDYIADYLDGIEGRPVYPDVEPGYLRPLIPATAPQEPETYEDIIKDIEKIIMPGVTHWHSPYFFAYF

PTASSYPAMLADMLCGAIGCIGFSWAASPACTELETVMMDWLGKMLELPEAFLAGRAGEGGGVIQGSASEATLVALLAAR

TKVIRQLQAASPEFTQAAIMEKLVAYTSDQAHSSVERAGLIGGIKLKAVPSDGNFSMRASALREALERDKAAGLIPFFVV

ATLGTTSCCSFDNLLEVGPICNQEGVWLHIDAAYAGSAFICPEFRYLLNGVEFADSFNFNPHKWLLVNFDCSAMWVKRRT

DLTGAFNMDPVYLKHSHQDSGFITDYRHWQIPLGRRFRSLKMWFVFRMYGVKGLQAYIRKHVELSHEFESLVRQDPRFEI

CTEVILGLVCFRLKGSNELNETLLQRINSAKKIHLVPCRLRDKFVLRFAVCARTVESAHVQLAWEHISDLASSVLRAEKE

>ELU11164.1 hypothetical protein CAPTEDRAFT_119245 [Capitella teleta]

MDASEFRKRGKEMVDYIADYMENIHSRRVIPEVQPGYLREMLPNKAPRKGDAWKDVMKDVERAVMPGITHWQHPRFHAYF

PAGNSYPSILADMLSDAIGCIGFSWAASPACTELETVVLDWLAKMIGLPPVFWHEHGIGGGVIQGSASECVLVCLMAARH

AAITELKNKFPFVEEGVLLSRLVAYCSKLAHSCVEKAGMISLVKMRELEPDESLSLRGSTLQRAIDEDRKMGLIPFFVCA

TLGTTAVCSFDNLNELGAVCEKENIWLHVDAAYAGSAFICPEFQHLLKGIEYANSFNFNPSKWMLVNFDCSTMWVRDRKV

LQTALTVDPLYLQHSHSDKAIDFRHWGIPLSRRFRSLKLWFVIRTYGIEGLQKYIREHCRLAKKFEGLVRKDSRCEVMGK

VQMGLVCFRLRGHNYRTQMLLRAINMSGKLHMVPALIHDDYVIRFAICAQNANDDDIIYAWNVISEMASDVINACESNNE

NEALKEIQRIDGSNPYRRRNLLLRMISDPKCYNPKVLRSLSHEKRRRSDSNKPRDEKDQNGAQV

>CAA49989.1 histidine decarboxylase [Drosophila melanogaster]

MDFKEYRQRGKEMVDYIADYLENIRERRVFPDVSPGYMRQLLPESAPIEGEPWPKIFSDVERIVMPGITHWQSPHMHAYF

PALNSMPSLLGDMLADAINCLGFTWASSPACTELEIIVMNWLGKMIGLPDAFLHLSSQSQGGGVLQTTASEATLVCLLAG

RTRAIQRFHERHPGYQDAEINARLVAYCSDQAHSSVEKAALIGLVRMRYIEADEDLAMRGKLLREAIEDDIKQGLVPFWV

CATLGTTGSCSFDNLEEIGIVCAEHHLWLHVDAAYAGSAFICPEFRTWLRGIERADSIAFNPSKWLMVHFDATALWVRDS

TAVHRTFNVEPLYLQHENSGVAVDFMHWQIPLSRRFRALKVWFVLRSYGIKGLQRHIREGVRLAQKFEALVLADHRFELP

AKRHLGLVVFRIRGDNEITEKLLKRLNHRGNLHCIPSSLKGQYVIRFTITSTHTTLDDIVKDWMEIRQVASTVLEEMNIT

ISNRVYLKETKEKNEAFGSSLLLSNSPLSPKVVNGSFAAIFDADEFLAKTYAGVRIAHQESPSMRRRVRGILMSGKQFSL

DSHMDVVVQTTLDAGNGATRTSTTNSYGHTTSAAQANSERQASIQEDNEESPEETELLSLCRTSNVPSPEHAHSLSTPSR

SCSSSSHSLTHSLTQSSARSSPVNQFRHITLCAVPSQSHLSMPLAMPLPNRNVTVSVDSLLNPVTTCNVYHGKRFLEPLE

NLAQTSASFSSSIFRLPTPMATPTRESPEDPDWPAKTFSQLLLERYSSQSQSLGNNSSTESSSLSGGATPTPTPMSSLDE

LVTPLLLSFASPSQPMLSAHGIGEGQRERGSDSDATVCSTTSSMESL

>ELU18925.1 hypothetical protein CAPTEDRAFT_180248 [Capitella teleta]

MVDYIADYLQDIRSRRVFPDVQPGYMQSLVPDACPESGEEWDAIFNDVERVIMPGMTHWQSPHMHAYFPALNSAPSLLGD

MLADAIGCLGFTWASSPAATELETIVMDWLGKMLGLPAEFLHSNTATMGGGVIQTTASDSTFVSLLAARSEAIKLYRLSN

PDLHDAEINARLVGYCSDQAHSSVEKAGLIGLVKLRLLPTDEDLSLRGDTLRNAINEDRENGLVPFYLCATLGTTGSCAF

DNLKELGVICEEEELWMHIDAAYAGTSFICPEFRHHLEGAEYGSSFAFNPSKWMMVHFDCTAMWVKNSRALHRTFNVQPL

YLKHENTGAAIDYMHWQVPLSKRFRALKLWFVLRSFGVSGLQKHVRRGVQMAKYFENLVNLDERFEIPATRHLGMVVFRL

KGEDELTELLLKRLNQTGQVHMVPASIKGKYIIRFTVTSTNTTEQDIERDWSIIQTMAGSTSADTNLSYAGRLRREGLRR

KDFGMSLILSNVPMSPKFINGSFAALFDTNDIIVDYARELGRNSVDFNGRPIRLCPRKKLREHDKQFSFDLSRMPPSRRT

THMSVFKQASLDSKIEEIFDSSFDSDSMYELDTEGFTNGDSAPAFVARADDDSKKNAEKIISSETINGLGEETCVTCHVC

GHVMTP

>NP_724489.1 tyrosine decarboxylase 2 [Drosophila melanogaster]

MDSTEFRKRGMEMVEYICNYLETLNERRVTPSVEPGYLRHLLPPEAPQEPEDWDQIMRDVEDKIMPGVTHWQHPRFHAYF

PAGNSFPSILGDMLGDGIGCIGFSWAASPACTELETIVLDWLGKAIGLPDHFLALKEGSTGGGVIQTSASECVLVTMLAA

RAQALKRLKAQHPFVEEGHLLSKLMAYCSKEAHSCVEKAAMICFVKLRILEPDDDASLRGQTIYEAMEEDELQGLVPFFV

STTLGTTGSCAFDNLPEIGKQLQRFPGVWLHVDAAYAGNSFICPELKPLLKGIEYADSFNTNPNKWLLTNFDCSTLWVRD

RIRLTSALVVDPLYLKHGYSDAAIDYRHWGVPLSRRFRSLKLWFVLRSYGISGLQHYIRHHIKLAKRFEELVLKDKRFEI

CNQVKLGLVCFRLKGSDKLNEKLLSIINESGKLHMVPASVGDRYIIRFCAVAQNATAEDIDYAWDIIVDFANELLEKEQH

DELSEIMNRKKQDTLAQKRSFFVRMVSDPKIYNPAINKAGTPKLSMELPSPVVSRGSAPIIRTQSSVDHNSWISWPLAFL

FNSNNEEKGSNVSLRFRHLDTNVRPSSSRRNSGAGSSPSPENELDYVNVQQQQMEQRSPRRSPMAVRKASSTRDNLN

>NP_476592.1 alpha methyl dopa-resistant, isoform A [Drosophila melanogaster]

MDAKEFREFGKAAIDYIADYLENIRDDDVLPNVEPGYLLDLLPTEMPEEPEAWKDVLGDISRVIKPGLTHWQSPHMHAYY

PTSTSYPSIVGEMLASGFGVIGFSWICSPACTELEVVVMDWLAKFLKLPAHFQHASDGPGGGVIQGSASEAVLVAVLAAR

EQAVANYRESHPELSESEVRGRLVAYSSDQSNSCIEKAGVLAAMPIRLLPAGEDFVLRGDTLRGAIEEDVAAGRIPVICV

ATLGTTGTCAYDDIESLSAVCEEFKVWLHVDAAYAGGAFALEECSDLRKGLDRVDSLNFNLHKFMLVNFDCSAMWLRDAN

KVVDSFNVDRIYLKHKHEGQSQIPDFRHWQIPLGRRFRALKVWITFRTLGAEGLRNHVRKHIELAKQFEQLVLKDSRFEL

VAPRALGLVCFRPKGDNEITTQLLQRLMDRKKIYMVKAEHAGRQFLRFVVCGMDTKASDIDFAWQEIESQLTDLQAEQSL

VARKSGNVGDLAQHFQIHLSTENATHEKSQ

>AAH52833.1 Histidine decarboxylase [Mus musculus]

MMEPCEYREYREYYRARGKEMVDYISQYLSTVRERQVTPNVQPGYLRAQLPASAPEEPDSWDSIFGDIERVIMPGVVHWQ

SPHMHAYYPALTSWPSLLGDMLADAINCLGFTWASSPACTELEMNIMDWLAKMLGLPEYFLHHHPSSRGGGVLQSTVSES

TLIALLAARKNKILAMKACEPDANESSLNARLVAYTSDQAHSSVEKAGLISLVKIRFLPVDDNFSLRGEALQKAIEEDKQ

QGLVPVFVCATLGTTGVCAFDRPSELGPICASEGLWLHVDAAYAGTAFLCPELRGFLEGIEYADSSTFNPSKWMMVHFDC

TGFWVKDKYKLQQTFSVNPIYLRHANSGAATDFMHWQIPLSRCFRSIKLWFVIRSFGVKNLQAHVRHGTEMAKYFESLVR

SDPSFEIPAKRHLGLVVFRLKGPNCLTESVLKEIAKAGQLFLIPATIQDKLIIRFTVTSQFTTKEDILRDWHLIQEAANL

VLSQHCTSQPSPRAKNVIPPPPGTRGLSLESVSEGGDDPAQARKIIKQPGASLARREGGSDLETMPDPFDDCFSEEAPNT

TKHKLSSFLFSYLSVQNRRKTTRSLSCNSVPMSAQKSLPADASLKNGGSFRARIFSGFPEQMMMMKKGAFKKLIKFYSVP

SFPECSSQCARQLPCCPLEAMV

>NP_610226.2 tyrosine decarboxylase 1 [Drosophila melanogaster]

MDVEEFRKYGKEVIDYICQYGTNIEERDVAPTLDPGYLKKLLPADAPQSPEPFKDVLEDFEQKIMPGVVHWNHPKFFAYF

PSGNSFPSVLGDMLSSAIGSIGFSWASCPAAAELETIVMNWYAKALGLPKAFVSDAPGSTGGGALQGSASECVLVSLITA

RARAISELKGQTSVHDSVFLPSLIAYASREAHSSVEKATKMALVKLRIIDADEHGRMRVDLLRQAIQNDVNAGLTPFFVV

ATVGTTGGCAFDDITEIGKVCRQVSSIWLHVDGAYAGNSFILPEMRVFSAGLEYADSFNTNPNKLLLTNFDASALWVRDV

MNLKSALNVNPLYLRHEHLTGVDYRHYGIPLSRRFRALKLWFVFRTYGIRGLQEYIRNHMALAKKFEMLVRKDERFEVRN

DVHLGLVCFRMRTGDEPNHMLLAQINHSGKMHMTPAKFNGRYVIRFCVTYEHATEKDILEAWTQIKCFAEEILRDHQLES
SSVPTTPEGSERTSSEPLAPVAGKPPIKKRLTRTKSLRFSFTRSISREQYQSQSEHLMDGCTPILVVDPKTLQENFQKAA
DDNDRNNSNGNVKLKDISDVDTDEASN

>NP_001246025.1 black, isoform B [Drosophila melanogaster]

MLASENFPTHHFKESIFKPYSTTSGDDLASVSPLTATAALVASTSSPADSTSTVAFEQASKMLANAANNNNNNNNNITST

KDDLSSFVASHPAAEFEGFIRACVDEIIKLAVFQGTNRSSKVVEWHEPAELRQLFDFQLREQGESQDKLRELLRETIRFS

VKTGHPYFINQLYSGVDPYALVGQWLTDALNPSVYTYEVAPLFTLMEEQVLAEMRRIVGFPNGGQGDGIFCPGGSIANGY

AISCARYRHSPESKKNGLFNAKPLIIFTSEDAHYSVEKLAMFMGFGSDHVRKIATNEVGKMRLSDLEKQVKLCLENGWQP

LMVSATAGTTVLGAFDDLAGISEVCKKYNMWMHVDAAWGGGALMSKKYRHLLNGIERADSVTWNPHKLLAASQQCSTFLT

RHQQVLAQCHSTNATYLFQKDKFYDTSFDTGDKHIQCGRRADVFKFWFMWKAKGTQGLEAHVEKVFRMAEFFTAKVRERP

GFELVLESPECTNISFWYVPPGLREMERNREFYDRLHKVAPKVKEGMIKKGSMMITYQPLRQLPNFFRLVLQNSCLEESD

MVYFLDEIESLAQNL

>ELU00176.1 hypothetical protein CAPTEDRAFT_151334 [Capitella teleta]

MMSHCLDIQDDPQNLEQVLSDCKETLKYCVKTAHPRFFNQLSTGLDIIALAGEWLTATANTNMFTYEIAPVFTLMEDIVL

TKLREHIGWPNGHGDGIFAPGGAISNLYAVAVARHTKIPDSKVKGMTGQPRLVMFTSEMSHFSIKRAGALLGIGLDNVIN

IKADDRGRMSVEDLESRIEQALAEGGVPFFVAATAGTTVLGAFDPINDIADVCEKYGLWFHVDGAWGGSALVSPTHKHLL

SGVHRVNSMTWNPHKLLGAVLQCSAILIREPGLLEATNSLKADYLFQPDKHYDVSYDTGDKAIQCGRHNDVFKLWLMWRA

KGDRGMQIHIDHLMSLSRYLQKKVRQTDGFEFVLENPEFINVCFWYVPKNIRHLPPGKERESELHKVAPRIKARMMEKGS

TMVGYQPMGKLPNFFRMINSNPASTYPDIDWLISEIERIGEEI

>AAK60398.1 cysteine sulfinic acid decarboxylase [Mus musculus]

MADSKPLRTLDGDPVAVEALLQDVFGIVVDEAILKGTSASEKVCEWKEPEELKQLLDLELQSQGESREQILERCRTVIHY

SVKTGHPRFFNQLFSGLDPHALAGRIITESLNTSQYTYEIAPVFVLMEEEVLKKLRALVGWNSGDGVFCPGGSISNMYAI

NLARFQRYPDCKQRGLRALPPLALFTSKECHYSITKGAAFLGLGTDSVRVVKADERGRMIPEDLERQIILAEAEGSVPFL

VSATSGTTVLGAFDPLDAIADVCQRHGLWFHVDAAWGGSVLLSRTHRHLLDGIQRADSVAWNPHKLLAAGLQCSALLLRD

TSNLLKRCHGSQASYLFQQDKFYDVALDTGDKVVQCGRRVDCLKLWLMWKAQGGQGLERRIDQAFALTRYLVEEIKKREG

FELVMEPEFVNVCFWFVPPSLRGKKESPDYSQRLSQVAPVLKERMVKKGTMMIGYQPHGTRANFFRMVVANPILAQADID

FLLGELELLGQDL

>Q80WP8.3 RecName: Full=Acidic amino acid decarboxylase GADL1 [Mus musculus]

MSHARDDHQGASQGSQWLSQARTLVQEGTLFNFIRCLLLFQGDSGQKEMTPGKKIPIFVDGVVLNGPQTDVKAGEKFVEE

ACRLIMEEVVLKATDVNEKVCEWQPPEQLRQLLDLEMRDTGESQDKLLKLCQDVIHFSVKTNHPRFFNQLYAGLDYYSLA

ARIITEALNPSIYTYEVSPVFLLVEEAVLKKMIECVGWKEGDGIFNPGGSVSNMCAMNLARYRHCPDIKEKGLSGLPRLI

LFTSAECHYSMKKAASFLGIGTQNVYFVETDGRGKMIPEDLEKQIWQARQEGAVPFLVCATSGTTVLGAFDPLDEIAEVC

ERHGLWLHVDASWGGSALVSRKHRRLLHGIHRADSVAWNPHKMLMAGIQCSALLVKDKSDLLKKCYSAKATYLFQQDKFY

DVSYDTGDKSIQCSRRPDAFKFWMTWKALGTSGLEERVNRAFALSRYLVDEIKKREGFKLLMEPEYTNVCFWYIPPSLRE

MEEGPEFWRKLSLVAPAIKEKMMKKGSLMLGYQPHRGKVNFFRQVVISPQVSREDMDFLLDEIDSLGRDM

>NP_032104.2 glutamate decarboxylase 2 [Mus musculus]

MASPGSGFWSFGSEDGSADPENPGTARAWCQVAQKFTGGIGNKLCALLYGDSGKPAEGGGSVTSRAATGKVACTCDQKPC

NCPKGDVNYAFLHATDLLPACDGERPTLAFLQDVMNILLQYVVKSFDRSTKVIDFHYPNELLQEYNWELADQPQNLEEIL

THCQTTLKYAIKTGHPRYFNQLSTGLDMVGLAADWLTSTANTNMFTYEIAPVFVLLEYVTLKKMREIIGWPGGSGDGIFS

PGGAISNMYAMLIARYKMFPEVKEKGMAAVPRLIAFTSEHSHFSLKKGAAALGIGTDSVILIKCDERGKMIPSDLERRIL

EVKQKGFVPFLVSATAGTTVYGAFDPLLAVADICKKYKIWMHVDAAWGGGLLMSRKHKWKLSGVERANSVTWNPHKMMGV

PLQCSALLVREEGLMQSCNQMHASYLFQQDKHYDLSYDTGDKALQCGRHVDVFKLWLMWRAKGTTGFEAHIDKCLELAEY

LYTIIKNREGYEMVFDGKPQHTNVCFWFVPPSLRTLEDNEERMSRLSKVAPVIKARMMEYGTTMVSYQPLGDKVNFFRMV

ISNPAATHQDIDFLIEEIERLGQDL

>CAA53791.1 glutamic acid decarboxylase [Drosophila melanogaster]

MSLNPNGYKLSERTGKLTAYDLMPTTVTAGPETREFLLKVIDVLLDFVKATNDRNEKVLDFHHPEDMKRLLDLDVPDRAL

PLQQLIEDCATTLKYQVKTGHPHFFNQLSNGLDLISMAGEWLTATANTNMFTYEIAPVFILMENVVLTKMREIIGWSGGD

SILAPGGSISNLYAFLAARHKMFPNYKEHGSVGLPGTLVMLTSDQCHYSIKSCAAVCGLGTDHCIVVPSDEHGKMITSEL

ERLILERKAKGDIPFFVNATAGTTVLGAFDDINTIADICQKYNCWMHIDAAWGGGLLMSRTHRHPRFTGVERADSVTWNP

HKLMGALLQCSTIHFKEDGLLISCNQMSAEYLFMTDKQYDISYDTGDKVIQCGRHNDIFKLWLQWRAKGTEGFEQQQDRL

MELVQYQLKRIREQSDRFHLILEPECVNVSFWYVPKRLRGVPHDAKKEVELGKICPIIKGRMMQKGTLMVGYQPDDRRPN

FFRSIISSAAVNEADVDFMLDEIHRLGDDL

>CAA90277.1 glutamic acid decarboxylase [Mus musculus]

MASSTPSPATSSNAGADPNTTNLRPTTYDTWCGVAHGCTRKLGLKICGFLQRTNSLEEKSRLVSAFRERQSSKNLLSCEN

SDQGARFRRTETDFSNLFAQDLLPAKNGEEQTAQFLLEVVDILLNYVRKTFDHSTKVLDFHHPHQLLEGMEGFNLELSDH

PESLEQILVDCRDTLKYGVRTGHPRFFNQLSTGLDIIGLAGEWLTSTANTNMFTYEIAPVFVLMEQITLKKMREIVGWSN

KDGDGIFSPGGAISNMYTIMAARYKYFPEVKTKGMAAVPKLVLFTSEHSHYSIKKAGAALGFGTDNVILIKCNERGKIIP

ADLEAKILDAKQKGYVPLYVNATAGTTVYGAFDPIQEIASICEKYNLWLHVDAAWGGGLLMSRKHRHKLSGIERANSVTW

NPHKMMGVLLQCSAILVKEKGILQGCNQMCAGYLFQPDKQYDVSYDTGDKAIQCGRHVDINKFWLMWKAKGTVGFENQIN

KCLELADYLYAKIKNREEFEMVFDGEPEHTNVCFWYIPQSLRGVPDSPERREKLHRVAPKIKALMMESGTTMVAYQPQGD

KANFFRMVISNPAASQSDIDFLTEEIERLGQDL

>NP_649211.1 uncharacterized protein Dmel_CG5618, isoform A [Drosophila melanogaster]

MAESVDGAETVDQIRDGLMDDWKILENVFHLLRKEDTFCVDPQKWDKQKIVPFLQPDKLKELINLKVRETENCSLAEIEE

LCQQVIHYSVKTSHGRFHNQLFGQLDPFGLAGALVTEAMNGSTYTYEVAPVFSLIETEVIATICKLAGYKEGDGIFAPGG

STSNMYGMVLARYKIAPEVKTSGMFGMRPLVLFTSDESHYSFVKAANWLGLGSYNCVSVRTNERGQMLLDDLEAKIAEAK

ARGGEPFFVNCTAGTTVLGAFDDINGAADVTERHGLWLHVDACLGGAALLSAKNRSLIAGLERANSFSWNPHKTIGAPLQ

CSLFLTRESGRLLERCNSTEAHYLFQQDKFYDVSYDTGNKSVQCGRKIDAFKFWLMLKARGYGKYGLMVDHAIHIARLLE
GKLRQRGDRFRLVIPEHEYSNVCFWFIPKAMRVSSEEETPEWWSRLYTVAPKIKEQMAHSGTLMIGYSPLKAKNLGNFFR
MVFTCFPILKSKELDFILDEIERLGEKIVL

>XP_014775469.1 PREDICTED: aromatic-L-amino-acid decarboxylase-like isoform X1 [Octopus bimaculoides]

MEKSENFDAATKQLTKYIIDYFGNIGSRNAYPAVEPGFMKTLIPAEAPETPDKWENVFNDIERVIMPGVCHWHHPKFGAY

FPARTSYAAILAAMLSDALVNVVISWKSAPAATELEVIVLDWLAKAIKLPEIFLSTSEGSGGGVIQNSSTECMLMSLFAA

RTTSINKVKTENNEMEDGIAVTKLIAYKSEETHSSFAKACKIALVKVRDIKLDDKRSMRGSSLKKAIEEDGAKGLVPFYI

CSTLGTTSVCAFDNIKEIGAVCKDEGIWLHIDAAYAGNAFICPEYRYLMNGVEYADSICMSPHKWLQVPIGCSVLWLQNS

NKLVDSIPEPGEYLQCTENSEMPNFMTWQIPCGRPFRSLKLWFVLRIFGIHRLQELIRKDIQLAHMFKDLIEKDGRFELI

GENIMSLVCFRLKGSNELNELLYKMINETREIHISNSEVNEILFLRFVVNTYHSDIQDMQYCFNVIGKLAEEVLSSCEKT

SQSLN

>XP_014775474.1 PREDICTED: aromatic-L-amino-acid decarboxylase-like isoform X2 [Octopus bimaculoides]

MDNSENFVAAVAQLAGYINDYFDYIRSRRILPSVAPGFLRHLIPEKAPQNPDKWEDVFQDIERIIMPGVCHWQHPNFGVY

FPARTSYAAILAEMLSDTLVNVVISWKSSPAATELEVIVLDWLAKAIKLPEIFLSTSEGSGGGVIQSSTTENILISLFTA

RTIAIQKIEKGHAGMKDGVAVSKLIAYKSPDSHSSVEKAFHIALVKYRDIELDEKRVMRGEQMRKAFEEDTAKGLTPFFI

CATLGTVSVCAFDNIDELGEVCREHRIWLHVDASFAGNALICPEYNYLMNGVEYADSFSMNPHKWLHIPTSCSIIWFKHS

STLIRAFDLSPLYLQCTENLDMPNYTSWQIPCGRSFRSLKLWFVLRLFGMHKLQEIIRKDIKLAHIFKNLIEKDERFELI

GDNLMTVICFRLKGSNECNKKLLKLIECSGELYMLSFKIDDILFLRFVINTSHTKPEDIQHCCNLICKLANAVLLTPNVH

D

>XP_014775466.1 PREDICTED: aromatic-L-amino-acid decarboxylase-like [Octopus bimaculoides]

MDSQEFQVASQQMIKFVADYLENSRERRVLPDVKPGFMRPLLPDHAPQDPEKWEDLFKDIERVIMPGVTHWQHPKFFAYF

PTGCSYSSIVADILSDAIACIGFSWASCPSCTELEVIVLDWLAKALKLPDCFLSTSDGPGGAVIQGTASEATLVAVLSAR

TKFLHKLKEENPGMEDGIALTKLVAYNSTQSHSSVERAFLIALVKHRSLKADDKRALRGETLRKAIKEDKAKGLIPFFVC

GTLGTTSTLSFDNIEELGLVCKFNQKGFTDCNHAESFNFNPHKWLQVNFDCSAMWIKDSRNISQAFSVDPVYLKHENEGI

MPDYRHWHVPLGRRFRALKLWFVLRLFGIKKLQEIIRRDIKLAAEFKKLIEADARFEIFGEVVMSLVCFRLKGSNEINEQ

LVKEINNDGRIHLVPSKCDDTFFLRFSICSNRTTYEDVQHAYKVITEIVEKIKQNSRVSLDSK

>XP_014775473.1 PREDICTED: aromatic-L-amino-acid decarboxylase-like isoform X1 [Octopus bimaculoides]

MENAENFEAAAVELAKYITDYFNNIRSRKVLPVCAPGFLRILITDRAPEKAEPWDDVFADIERIIMSGICHWQHPRFASF

FPSRTSYAAILGGMFSNALATDVISWKSFPAATELEVIVLDWLAKAIKLPKIFLSTSEGDGGGVIQNSSTECMLMSLFAA

RTASINKMKTENNEMEDWIAVTKLIAYKSEETHSSFVKACKIALIKVRDIELDDRRSMRGNSLKKAIEEDRAKGCVPFYI

CSTLGTTSVCAFDNIKEIGAVCKDEGIWLHIDAAYAGNAFICPEYRYLMNGVEYADSICVSPHKWLQVPTGCSVLWLQNS

NKLVDSIPEPGEYLQCTQNSEMPNFTKWQIPCGRPFTSLKLWFVLRLIGIRQLQKIIRKDIKLAHIFRELIDKDGRFELI

GENLMSLVCFRLKGSNEINEELIKSITLTREIHIGCSKIDDNVFLRFVVHAYQATIEDINYSYKLIAKLADQVLHSQPKP

RTQTSKDCLKCMISIFH

>XP_014774406.1 PREDICTED: aromatic-L-amino-acid decarboxylase-like [Octopus bimaculoides]

MDSSEFRKRGKEMVDYIADYMETLSVRKVTAQVEPGYLRRLLPNKAPYRGETWEAIMKDVDNAILPGITHWQHPNFHAYF

PSGNSYPSIIGDMLSDAIGCVGFSWAASPACTELETIVLDWVGKMIGLPKDFLHEGGKGGGVIQGSASECVLVTMLTARH

STLKKLKGSFPFVEDGTLLTKLVGYSSKLAHSCVEKAGIISLIKLRELEVDDNYSLRGHTLEAAIEEDRKLGLIPFFVCA

TLGTTAVCSFDALNEIAEVCNRERLWLHVDGAYAGNAMICPEFQHLLSGIEFAHSINFNPNKWMLVNFDCSLLWVKDSQL

LSEALTVDPLYLQHPHGDKAIDFRHWGIPLSRRFRSLKLWFVLRTYGIEGLQHYIREHVRLAKLFESKVLEEDRFEVLGE

VIMGLVCFRLKGPNTLTQKLLRQINESGRLHMVPALLNEHYIIRFAICAQNADDSDIIYAWQTISKIADELLASRERESS

VRESRRIALSDTSEEGESAEEEEVFLEFDKENIYDNQQLNFQRARMRRNLFLRMVSDPKSYSPHMMKSYSIDDSSSRLPS

DTPSETGSTNSTKRSPKTPQQATPL

>XP_014769443.1 PREDICTED: histidine decarboxylase-like [Octopus bimaculoides]

MSLQITHWQSPHMHAYFPALNSFPSLLAEMIADAINCLGFTWASSPACTELEAIVMDWLAKMIGLPSEFQHRSPQTNGGG

VIQNYVAVDSVRGCIQDGFDCFHVGYIQHNADVICIHQHGPTWPNMVYNKIRNRDPAIYAGDLPPLAHSSVEKATLIGLV

KLRLLPTDDHLSLRGNTLEMAVKKDKEAGLIPFFVCGTLGTTGACAFDNLPELGAICVAESIWMHVDAAYAGTAFICPEF

RKFMVGIEHAHSLAFNASKWMMVNFECTAMWIKDSTSLHRTFNVDPLYLKHENSGAAIDYMHWQIPLSKRFRALKLWFVI

RSFGVEGLQKHIRHGVKLAILFETLMKQDERFEIPAERHLGMVVFRLKGENELTELLLKLLNKSGKLHMVPASFKGKYVI

RFTVTSQYTTEEDIRSDFKTIQDTSSVVLRDLSLLMNKTKEDELIRDIPEIPEEKAITTSSEETKLERTLSMKRRDFGMS

LILSHVPCSPKFINGSFAAIFDNNETILEFARHLTSSDINGRPIRLSPRRGVKLREQAKQHSLDFSTLIPGRRMSTGTAR

FKQGSLDTKVDDILDSTPDNYYSNGFDETHGKKNDKEDGKENNKDEEEEQQELDAQNQQQEEQQQKQQFQVLQLQQNQLH

SVLAASQEKASSFLDKIKKLTPKRLTGETAANNNSSPVKISNGLHMCPHCGKSIV

>XP_034310760.1 cysteine sulfinic acid decarboxylase [Crassostrea gigas]

MKSIGTSLARAALRKLKIDLAKISQPRLFSSMASKDREDAFSNPDVQQMLLNLHHSMVKTLIDSETKELP

VCNFKQPEELLKALDLEIEKEPASNDTILDACKSVMDYSIKTASPRFMNQLYSGINPSCLAGSWVTEVLN

TNLHTYEVAPVFVMMEKYMMRKLSKLVGYDNGDGVLCPGGSFANMLGMHLARVQIDPDIKSKGMGNNKKM

VLFASSEAHYSIAKGASFLGFGESNVVRVETDKIGMMKPDVLDQKIQECIQQGSIPAFVMATTGSTVLGS

CDDLNAVADVCEKHGVWMHVDAAWGGGVILSEKYKHLMNGVHRSNSVAWNIHKMSTGLVQCSIFLTKSSG

MMEECNRFNAEYLFQPDKHYDVSYDIGDKTVQCGRKVDILKLWTLWKSRGDNGMAKQTDNAFENAKYLVD

QIKKREGFRLVLPEFQCPNICFWYIPSRLRNKEENADWWKEVSKIAPQIKRKIMEDGSMMIGYNPLTTKG

YVNFFRVIITNPMTTPQDMDFILDEMDRIGHAL

>XP_011449820.2 aromatic-L-amino-acid decarboxylase isoform X1 [Crassostrea gigas]

MGDMVKVRKKFNGTTEDRGRTTKKANAMDSVEFRKRGKEMVDFIANYMDTIAERRVTAEVDPGYLRNRLP

GKPPRTGDKFEDIMNDVERAIMPGITHWQHPNFHAYFPAGNSYPSILGDMLSNAIGCVGFSWAASPACTE

LETLVLDWIGKTIGLPKQFLHEEGTGGGVIQGSASECVFVMLLAARHKAMKELKKRLPYIEDGVLLSKLV

AYSSKLAHSCVEKAGMLGFVKMRQLDVDVNYSLRGHVLERAIEEDRKLGLIPFFVCGTLGTTACCSFDNV

AELSEVCSRENVWLHLDAAYAGNALICPEFRFLINGIQNVTSINFNPNKWLLVNFDCSLLWISDKNLLTS

SMTVDPLYLQHKHDDKTVDLRHWGIPLSRRFRALKIWFVLRTYGIEGLQDQIHQHIKLARLFETYVKNDA

RFEILGKVTMGLVCFRLKGPNSLTAKLLHMINESGKLHMVPALLSEIYVIRFAICSQNARDEDVEFAWKI

VSNEASSLLMERENIENGICTEKVGDGDQKNPDIDDVFPDFDDEIIFDQQKSNLHRARLRRSLFMRMVSD

PKCYNTKVLKALCVDKKRTRSTPSENSTELDYDK

>XP_011417382.2 aromatic-L-amino-acid decarboxylase [Crassostrea gigas]

MDAEEFRRFGKQMVDYVADYLENIRDRKPFPDVSPGYLKELIPDKAPDEAEQWPDVMKDIERVIMPGVTH

WHSPQFHAYFPTANSYPAIVADILSDAIGCIGFSWASSPACTELEMVVLDWLAKMLELPDCFLHSSEGHG

GGVIQGTASEATLVALLSARTQRLHQILGDKFSHSPDEGIISKMVAYCSAQAHSSVERAALIGAVKVRLL

ETDEKFSLRGETLQRAIEKDREAGLIPFFLCATLGTTSVCSFDNVLELGTVCEKEGLWMHIDAAYAGSAF

ICPEFRPLLNGVEHAMSFNFNPHKWLQVNFDCSAMWVKDSRLLSDAFNVDPLYLKHDNQGAIPDFRHWHI

PLGRRFRSLKLWFVLRLFGIKGLQERIRKDVKLAHQFEELVKADQRFEIFGEVVLGLVCFRIKGSNEVNE

RLLKTINDDRRIHLVPSKVNDTFFLRFAVCASRTESKDVKFAWEVIQELTKKISDEKK
>XP_034320494.1 histidine decarboxylase [Crassostrea gigas]

MDFEEYRLRGKEMVDFIAEYLKSIRTRRVFPDVSPGYMRTLVPEAAPQEGEKWDDIFRDIERVIMPGVTH

WQSPYMHAYFPALNSFPSLLGDMLADAIGCLGFTWASSPACTELETIVMDWLGKMIGLPSEFLHSNKETK

GGGVIQLTASDCTFITMLAARTEVFQKHRKLDPDVDEAHINARLVAYCSDQAHSSVEKAGLISLVKMRYL

TTDDDLSMRGHTLQEAISRDKEDGLIPFYVCATLGTTGACAFDNIKELGLICEKEGLWMHIDAAYAGTAF

ICPEYRSYIEGIEHANSFAFNPSKWMMVHFDCSAMWVKDCRTLHRTFNVDPLYLKHENSGAAIDYMHWQI

PLSRRFRALKLWFVIRSFGIEGLQKHVREGVRLTSKFEDLLRKDQRFEIPASRVLGMVVFRLWGENEMTE

TLLKRLNKSGKVHMVPASLKGKYVIRFTVTSQYTTDQDIERDWKIISDTATKVLHDTESEEDEAYSDEEV

SSPEVEEEKQFVRVPSIKKKEYGMSLLLSNVPMSPKLINGSFAALFDDSEAMEEVAKQISAENAEHLIPM

SPRKRKLRDQSKHQSFDQTTMADRCLSNSYKHQGSLDSKLDEIVNSSAYLETRMNGMQMDDVFEAEEERP

IDDTEDENCPMKCKDDVSYNEGSTQTETGQRRVDRTPNGQTQSMSQRKNPNIRLRIPEVNIPSMRNVCPH

CGNRFSFS

>XP_009050823.1 hypothetical protein LOTGIDRAFT_201667 [Lottia gigantea]

MDAEEFRKRGREMIDYVADYLSNIESRRPMPDVEPGYMKTLIREDAPVEPDQWEDVMKDIEPIIMKGVTHWHNPNFHAYF

PTANSYPAILGDILSDGIACIGFSWAASPACTELEVHMMNWLGKMLNLPKEFLFSPGGHGGGVIQSTASEVTLTCLLSAR

TKIINQFKKNNPSIEEGEVISKLICYTSSEAHCSVEKAGLIGAVKMRILEPDEKGSLRGVTLEKAIKEDKAKGLVPFFIC

ATLGTTGCCAFDNIKELGPICEKEDIWMHIDAAYAGSSFICPEYRPLLDGVEHAMSFNFNPHKWLQVTFDCSTLYFRVKD

SRLLAGAFVVDPLYLKHDTEGEMPDYRHWHIPLGRRFRSLKLWFVLRLFGQKKLQAQIIQDIKLAKEFEKLVLEDKRFEI

SFDVIMALVCFRLKGTNEINEKLLKLISEDGRIHIVPSNFKEKYYLRFAVCATSTTSEHVQFAWKVITEMTDKLKV

>XP_009056307.1 hypothetical protein LOTGIDRAFT_119964, partial [Lottia gigantea]

QFVFAGKEMVDYIADYLENIRSRRVFPDVKPGYMRDLLPDSAPTDGEQWDDIFQDIERVIMPGITHWQSPHMHAYFPALN

SFPSLLGDMLANGIGCLGFTWASSPACTELETIVMDWLGKMIGLPSVFLHSNEQTMGGGVLQTTASNATFVSLLAARSEA

IRRHKNSLQLDDAEINARLVAYCSDQAHSSVEKATLIGLVKLRLLNSDDDLSLRGHTLQEAVDRDREAGLIPFFVCATLG

TTGACAFDNLKELGDICDSEGLWMHIDAAYAGTAFICPEYRQYMVGIEHAQTFAFNPSKWMMVHFDCTAMWVKNSGALHR

TFNVDPLYLKHENSGAAIDYMHWQIPLSRRFRALKMWFVLRSFGVEGIQKHIREGVRLAGMFENMIRKDKRFEIPAKRIL

GMVVFRLQGDNSLTETLLKRLNRSGKIHMVPAALKEKYVIRFTVTSQYTTNSDIARDWKIIQDCASKVLADEQIVRRRRP

RIGSLRRKDFGMSLILSNVPFSPKFINGSFAALFDNSNGMVEFAKELTTGDFNGHAIRLSPRRRIKLKDASKQQSLDCAP

LSPRHESLRFKQGSLDSKIDDILESSSYESGSQTNMQNGEEALDENENDGRAAPESDTNPSNRKQKVVINLKPSMILPSN

RKVDNGVQAVNGMHLICKSCGRSFED

>XP_009048193.1 hypothetical protein LOTGIDRAFT_139922 [Lottia gigantea]

MQSEEFRKRGKEMVDYIADYMDKLSLRRVTPEVEPGYLSQLLPKEAPKKPEEFNEIMHDVDKAIMPGITHWQHPNFHAYF

PAGNSYPSILGDMLSNAFGCIGFSWAASPACTELETIVLDWMGKMIGLPKEFLHEDGVGGGVIQGSASECVLVALLVARH

LAVRQLKSRLPYTEDGILLSKLVAYSSKLAHSCVEKAGMIGFVKMRQLDVDENFALRGSVLERAIEEDRRLGLIPFFVSG

TLGTTACCSFDNLAELGEICSNERIWFHVDAAYAGNALICPEFQHLLKGIENVNSINFNTNKWLQVNFDCSLFWVKDKDT

LTTAMTVDPLYLQHKHSDRAIDFRHWGIPLSRRFRALKLWFVLRTYGVDGLQHRIREHCRLAKLFETLVATDPRFEVLGK
VTMGLVCFRLKGSNYLTQRLLKFTNESGQLHMVPALINEHYVIRFAICAESACDEDISFAWKVITKTATEMLAARLSRKD
TNISMDQKNQSSMDNSTRLRRNMFLKMVSDPKSYNPRVLKSLNFDKNSSRHRSKSLGGIGPLSLSPDFNFGSSI

>XP_009051405.1 hypothetical protein LOTGIDRAFT_181541 [Lottia gigantea]

MDVEDFRKHGHELIDFIIDCLFSQEKELPNPKVETGYMSKLIPDKAPEDPDEWKDLLKDIQPVILDGITNLHSPNYHGFY

PLRSSFPSILGELLRHCFETPTANWMANPAGIELEMNMTNWLGKMLQLPEEFLFDKKRSGGGVILTSASESVCTCLLAAR

THTIREVKKTNPNLKDGEIITKLVSYTSAEAHPSVIKAGLISCIEMRIIETDSNGGVNTSALEKAILSDIENGLIPIFVC

ANLGSTGCCGFDNLNEIGAVCNQHKIWMHIDAAYAGSAFICPEYRYLLDGVELATSFNFNAHKWLEIAPPCSVLWVKNSK
LLSEAFPVDLCAENNFDNKDKAPESPAEHIPVYRHWSLQFTRDFKSIKVWFQLRLMGIKALQEIVRTDISMAKLFESLVK
EDNRFEIVVPVTLAVVCFRLKGTKKKTKDLLELIRADRRIFMIPSALNGIFFIRFVISSSKTTPKDVHFAWKVIQEKADQ
LPQQLIEG

>XP_009052789.1 hypothetical protein LOTGIDRAFT_115956, partial [Lottia gigantea]

FLIEMTHILTDYILKENDRASKVLDFHHPHQLREMMDHCLDIDENPRDLEQILSDCKETLKYCVKTGHPRFFNQLSTGLD

IIGVTGEWLTAVSNTNMFTYEVAPVFTLMEEVILRRMRDLIGWQDGDGIMAPGGAISNFYGVLLARQKYLPQARLNGMFG

CGEPVIFTSKQSHYSIKRAAILLGIGANNVIYVDCDHRGKMMVKDLEEKIASSKRNGKLPILVNCMAGTTVLGVYDPLEE

IADVCKRNSIWLHVDGAWGGSVLLSRDHRHLLNGVERADSMTWNPHKMMGVPLQCSVILTKHKKLLAEANQSNADYLFQK
DKNYDVTYDTGDKTIQCGRHNDIFKLWLMWRAKGDQGFEEQISKNFELAQYTLEKVQNRDGFHVILDKLEAPNVCFWYLP
LKWRQQSPSDINAKELNKIAPQIKAQMMESGTMLVQYQPLGDLPNFFRVAISNPVITTRDIDFMIDEIDCIGRDL

>XP_009053632.1 hypothetical protein LOTGIDRAFT_160336 [Lottia gigantea]

MKEIVQKAGDRSQKVVEWKAPEELRELMDLDPTAVGETHEKLLMRLQDIIKYSVKTGHPRFVNQLFSSLDPYGLAGQIVT

DALNTSQYTYETAPVFTLMEETVLKTMRTCIGYTEGNGIFCPGGSLSNIMAVNCARHFMFPQTKKTGMFGLPPLVIYTSE

LAHYSIKKAGYLLGFGDDNVKLVKTDELGKIIPEDLERQIQETIDEGCTPFMIVATAGTTVFGAFDPIDKMADVAQKYGL

WYHVDGAWGGGVLMSKKHRDMMKGVDRADSVTWNPHKLLGVPQQCSCFLTKHVNILQQCHRADAKYLFQKDKFYDTSYDT
GDKTFQCGRKVDVLKFWLMWKAKGSEGFEQHIDKLFDNTRYIVEKLKQREGFRMVVNEPDCTNVCFWYVPPSLRNMPEDE
EFWNRLHTVAPKVKEGMIRDGTMMITYQPQKDLVNFFRLVLQNSATTYEDMDFFIDEIERLGKFL

>XP_009059185.1 hypothetical protein LOTGIDRAFT_192210 [Lottia gigantea]

MDTFGVAGAWLTDALNSSQYTYEVAPVFTLMEKTVLSKMLSLIGFTDGDAIFCPGGSVSNMYGINLARYKSFPDVKTNGM

QGLPNLCILTSNKGHYSMKKGAAFLGLGINNVIPVETDECGRMSPHSLETTILDVKAQGRVPIMVNATSGTTVLGSYDPL

NAIADICQNHGIWMHVDAAWGGGVLLSKKYKHLMDGIERVDSVTWNPHKMMGAPLQASAFLTRHKTLLAECHSAHAKYLF

QQDKFYDVSYDTGDKSVQCGRKVDVLKVWMMWKAKGDKKFERDINNIFYCAEYLTSQLRQKEGFRLVLDKPECTNVGFWY

IPPSMRGQTEDTDWWQRLSKVAPAIKQRMTEQGTMLIGYQPDGERVNFFRMIVSNLDTTSADMDFVIYEIDRLGNDL

>XP_009061653.1 hypothetical protein LOTGIDRAFT_127363 [Lottia gigantea]

MSIFIKRKGKKDLRLLQNHVHHDSVLDKTYEDPETLKFLQDVHNLMVEEALVKGTKRETPVIEFQHPEELKKLLNLPIGK

EGTSNADILEDCKRILKYSVKTGSLQFYNQLYSGVEPYSLAGSWVTDSLNTNLHTFEVAPVFVILEQYMVQKICKLIGFE

NGDGVFGAGGSFCNLMATHLARYHRFPDMKTKGMYDQPKLDLYASDQAHYSLLKAGGYLGFGTEHIINVKTDDKGCMIVS

DLEAKIEDSKKKAKCNTDLLFTYFQGNVPFFVMGTCGSTVLGAYDDLNAVADVCKKNNIWFHVDGAWGGGALLSSKHRHL

MKGVERADSVALNFHKMVGAQIQCSAIFVKVKGLLEKTNRAYAEYLFQPDKFYDTSYDIGDKTVQCGRKVDVTKLWLLWK
GKGDKGLEERVDNAFDNAQYLTKKINETEGFRSVLPEYQCTNICFWYIPPSLRGQKETPEWWEKLGKISPIIKERMIKEG
SLMIGFVPIKMKGLVNFFRIIVINPACEHSDMDRILSEIDRLGSDL

**Piezos (Figure 8):**

>Helobdella1 robusta XP_009020748.1 192356 P1

MCKYWIFACAALFLFISLQEAVVFRLIYMSMFLYFIISFQVSYSFWRATIKVFWWVVILYSMIVLVCIYTYQFDGFPKHW

ESFTGMRKEMLLNLGLKEIDTTELFIDLLTPTSFLIVVSIQLHYFQNDFLKMSDLNRFKSMENEPAPERRETADSALASR

RGRKDRILIFCTYLWTKFYRFLNMTSAVLWRFVEVHIFKVTVTTIFICCVYEVSALNTLLVLVVVILLPFPRLHSTLSIL

QMILSSAILLFKMIYQMNIIVDDLMTSNCTSNDSSVLIHPPFDQTLNNTNFIGIHKDKNMARYLMIYIIMILMLSLEAIV

RYHQIQHYRHPLHTPPMDGIVFMYIKRQHADQGLSQCCKFFINYSFYKFGKELCFIISMTTMVVRLDLYSVVYGVFLGVL

LMLHRRHCAFVWPVYIIFLVILLVAQYLSCVGAPPALCWVYPWSYDLSKNLKIFLYLPDYENPPNPRKLIGDYFQLLFAC

LQWNVFSIEAHKLEEFGGDDNESIIDRLNKKVKNPQSDFLSYKDSYLDYIKTVLFFYSFWVTLMVVFISGMSRISLFCLG

YLVGCFVFLWFGEGMLLKPLKKLTNMWMGLLMYCYLVLLAKCILQLLGCVYISQLNQDYCWVIQLFNIVCLKSTTYVISK

SNISKCNMSVDEAGLSWDALCFAFLLFMLRIFKTWYFQHVVNELRINNKLAARGAELINNKLIDDINLLRANEERVLQNI

KVKMDRIRTKHAACMQTRMLTKEQFDDHYTIIRSGDYYMFKDVDEEDQEMTDGDTMSEEEDSNNKEHDDWRRKRTTVAPT

TLLATAFFGGGPKEALSLIEKTSNFSRSISISSAKEYTTPEADMSPTSILDGTTSTMLGSEIDELRPFTVDDYPSMVTTS

LSTQPVTTTSSTVALPDKITTTFSTITPHTTTYSMSSYLAPTTSSYQKPLRHSDVMPSLPEVTLEMESEMSPVGSRTVCP

SADDSTVMEGKKEEEEIEEEELDDDVIDEATKKVETGLKMCKIVLIGFIDTLILRINISTRHYRSVSMKLHEEKEREKLL

SRFCKLIALFKDSMLKMPRVESDMLEEDSKDGVAVLKEDEITNRKRKENEFHNTMPRIIVLIIAISEALVARTDLLCYFA

MIFNTLMSASILSIVYPLSVFFWGMLSVPRPTKFYWVTAITYTEAGCFFYFHLFSLYSDMIFIIVIKYLFQFNFYPWNNS

TEKFAWPQIIGIEKKEMYAALDFFVLFCLFIHRTMLKKRGLWTHGLKDDAKYLRQPHSNNISNIECNRAGGDNALAGDGE

PKKLSPCQKLLNPIINFYQHITHPKYSAVNDVFAPMFACDLFTFLIVPVPFLAMLLVQFLLIIIDRALYLRKLVFGKFIF

HIILVIIVHTWMFFLLPIITDIPFVKNRAAQLWYFTKCIYFGFSCYQVRSGYPIRISGNFLTKKFGYFNLFCFKGFLAIP

FLLELRALTDWMWTDTTLALTSWLQMEDIYANVFVLKCWRKIEHDSPTPRAIPRHPVVKYGLGGLVLFLLIFIIWLPLLL

FSMSSSIYEPNPPIAVRFQLSVGGYMSSQPIFKTSINRNQLSPIDPKEYVNLKQIYMHIPRALAFLNAYREDDVYKISIG

NTSSSTWEISYPTEKKLLEDLKSMRNMSFSVDITITRDPPLNGLTEREVSVHFTRPMDPDNPKDRDALNKTFRMINGSYK

ESVTIENLFPRYLTVPSRKSVNESGLLYHGESSKLAHRSDITLSLHQNKNDKSRWWELKENCFREMREHDLSVCNSGRGS

LIVAFSERIPKGVLTYVNNFGVIGLYVSVILVIGKFVRMQSVGNIQNIMFTSLPYIERIYGLCTDIYLVRQAREFAFEEL

LFAKLNFLHRSSETMIAWTRYPKPKTD

>Helobdella2 robusta XP_009013717.1 169634 XP_009013716.1 190815 P2

MIFSGLALIGQMVFQIFILIYKSSDTLFYNCLPLELIFRQIGLQKINDSDLGNALRLILPDAVASMFSLFIYFICKCILQ

PQPSSQPSVSALVGQLQGSPSPQVRFYKSGSLLIEYLGSGIVLLLMILCGIAEPNVLNMIYFVSFLLAATLWGCGIKFGR

WFIAMEVCLLVYCGFHLVAIYLHQFPSIQELWFEREGWNETVFINADNLTNSTGILYRLLGLKTFVYTNCTHPFMLNFNV

NLKWTDILQVVAMSLVSPGQHPNYQSMENVTNSSYASPTNQVDDRLNLRIRRRQEQPNKIFEIFKTISLYLAEFMLSQSY

NAALIVMMAWSITYHSWPTFLFLVAACLIWMVPQKRDLVLKTSPVFVFYALILLSFNYIYGLDLTSDELPSPMEGKGYSM

TKIGLIRRNKHQEPFVDLAIQSFFTLVFILTLRQSRWEASKDDLDFDNLRSDFYLSRKMSGALINGILVKYWILVCSLLF

LVMALQESVLYRIIYMILFLYFVITFQVSYQFWRIWVRVFWVIVIFYSMVVLISIYVVQFDEVMVRLNTMNAKSPIILED

IGLRKYSTGELFVSLLTPTSFLIVVILHIHYFQSQFLVISDVNRYSREIDKKG

MNMRRIYLYIKRTYHELANLLWRILEVHMSKIIVIFIIVVCTYDVCALNVLLLLVALILYPLNFHHTLSILTMFYSSFII

ITRMVYQLSIFEEKTFTTECLFKQAEYDVIAYNGTVSAVAYFGYRKTNNIFFEVKLYVCLVILLALEMVISTRQKQHYNH

PRHLRPYKGVIFYGVNRGNADVTVTSWLKFFANYGFYKFGKEICFIFVVIVMTTRLDAFSLVHGLLLGCLIFLRRWACRR

VWPVYIFLLIIAVFVQYLSCLGLPKSLCIDYPWNLNGYSKVFTRNLKVFLFLPDYVLPPYALKIIGCSYLDYVKVGVFYY

SFWLTLLILFIAGTFRVNLLCLGYLLGCFFFLWHGQSLLLKPLKHIKKMANHVVLYFLCYHGQVCFAGSRPQFDREKLGF

AWDAMGFAMVLLLLRIHKSWFFQHVVNELHVDSVLASRGSELIVEMTQRRIETQRKYEMSILDKIRLKVERIRENLLKKQ

EATNQPIVQYADHYQVIRSGDYYMFEHLDLDSNSSEVKDEDILSTEAQKKMAKTPTVAELFNIVSIANMETAIKFWSDWM

RENRPKNDGEKKEGKKRDVEEEGREGDGHGVEEEDLQEGTSDGTVIVRPHHKPVVPENENLLAIIKDVTGAKNLVPSDVD

PPVKQKRSVAQTIRISFYFGYIVTWAGAEQLIVFLNSLAQDYISISDILFEEKQKIKKATILYRSNLLSEDSFHEEPADW

ASHHHHRQYTDHQHPHSPDAASPIDDDKAVLTTTTPSTTADQPPQQLSFKYKLKTFDPFTEIKKIAHLLQSSSAQDLASS

QQDQPQTPTSQPPSLPPHHHHHLALPLVIFNSCYTVFLSNSELLCYFAMVLNAIVYGSILSVVYPVTAILWAMMTCPRPS

KRFWLFAIGYTEICIIVKYVYKFVSPYTDQPDVVNKDTLFQWPYMLGLANMTHFDIFLLFCLFFHRSVLMMQPKQKLEDP

EDEMAVLAAPKPSRPAPSPSSAEQEASSPSSLPPLTGQPSTLRKRNVKKFKNKNEKGKIDDGVDGRDAEVVDGDAREKKS

DDIKLKSMRTITFDGDDDAGAAALAGHDDDDNEAGDDDDDEESGMEDELVRLCCENPLIRPLYNYCNNLRDQSFRAATDV

YVFMFLTDLITFLIAVFGFSSFGPNDASANNDGKVTTVLKSNKVFLKFIFQIFLVVLIHAWIFIGLPEVTQRRFVNNVAA

QIWYFVKCVYFVLSAYQIRSGYPTCVLGNFLTKKYNYFNLFSFQAFLAIPYLLELRALMDWMFTDTTLSLSSWLQMEDIY

ANVFCVKCNRSAEKRYKTPRGNKRAALVKYGLGGIILVALVIIIWFPLLLFSMSNAIFLTSQPSEVYVDIKIAGYEELFK

MTSQAVDPFTSADYDYFYMKNSFFMTQYQTSDICNVTIKGESGSVWSITPPALNDLVKQLERKDKVNFIFSMQVRRDPKI

GGEITYGQFKRALDRNNTEDAMIRDGLINLIRRTSNESVTLMNVFPRLILVPAKGVISSVERSFVKLGPNDTDTEQTGSL

KLTLHSPSNFTYWWSIQQYFNTSDDEFFMEDPTARANKNRATPRPIISTTIRPLMRTIPRTRQKNVSAAPTHPHPATDYG

GMPSVVDTSYSVSKDKLQLTMFNERVAPSGVLSIINNYGHAARFNTTKLRYNPHLTLPCTRHRVIGLYVSLVLVIGRFLR

MYIQGLSFKIMFVELPDPQVLLTLCQDIYMVRESKEFRLEEELFSQLIFLYRSPETLIKLAYSLAQ

>Capitella teleta ELT90834.1

MARQWVCFGLFRFIFPLAFLAATVVRFNGFSLLYGCLLLVEPLIPRPTPGSMSGACGRFLIASTLLCSLTLLAQIVFQSY

LLIVANDQEPYGTILKNCSQLETSLRQLGLQRLEPNDFGNILRVILPDIGTFFTSLFILIICKVLLSSNEPKRDRDTPSP

SSRPIESRHTSVVKQRLMRHSTVLVNLAGHFLVILFLALSGITYPCVLSSVYFVLFICVSTWASCCQGLGRRYAALRIGT

MIYSAGHLVLIYVYQFQFFQTILPPNTLIARIVGLTGIVKTDCRKPWLLDFYPIDWPQLVSPGVILLLYFTLACETRRWF

NRIREFDVNRGSKPPRRHRVSGNSERQVQQEGADQEFFQFLFCIVACFELPQNVPCSDDELVSLVEGAADAHHRYNTMET

VEAGSVAPSITSSGIQSEAGERNSQGSLLQKRGPLESIAIFVMRQSYVGTLIIMMAWSITYHSWLTFVFLLWACIIWLIP

QKRDVCLRCSPLIVLYAVCLLVICYVYGFNLTDAELPVQSENGYKFREIGLIKYDHQCIQLAVQVLFTVMFWVTLRQAVQ

EWMQRRQSAMGQGLALEVVGAPPPENEDPFRVQHRMPSNAKDGLVGHDSDAIKSVGRWMWHMFCKYWIFVCTGMFLLIAL

QEAVIYRIIYMVLFLYFVITFQSNLTRPFLSHALIGGSRPMICYRWWRLSISVFWWVVIIYSMIVLCVLYTYQFDEIPWG

WVNRTSISKEVLSDIGLEKFDTGTLFVKLLTPTSFLIVIIIQLHYFQRPFLTLSDINRFRGPRPKRKWFWQKSSREETTE

LNVEDEDEANRIHSNVSAQSDDPIAGPSHDEVTQETDLETSTQVTSISPRKQRRREQMAKFRAGLAKASLVAKGWRDRFA

RAFSHAWNTMYNMLDAISALLWRFMEVHIVKIVMLTVIIVSMSEVCALNVLFVVFVVVGVVSPSLFALMSHLCQCWSAVV

ILCKMTYQLNVISSEDVLTNCTVSQQPISTQYSTVFHSQHVSNAPFSNATHDQLPYPFNGTIDNANYIGLEKAPNLAEYL

RNYIFVVVIIGLEAVVKVHQMQYWNHIDHSKPKHGIVFPETKRADADTGLLPCAKFFMNYTFYKFGLQICFIMTMITMVV

RLDVYSVLYGIMLGVLLLLNRRTSYVVWPVYMVVLMVLLVLQYLSCLGVPPGLCWVYPWDNISNFTRNLKIYLYLPNYLD

PPNPYKLIADFFQLLFVCLQWWVFRLENSPSQAEAYGGGSNKDIVRDVEENKPNPVEDFLSYKDSYVDYLKAGLFQYSFW

VTLVIVFIAGTARISLFCLGYLVGCFFFFWFGQDLLLKPLPNLLRLWGILLGYNYFVILAKACLQMVGCVYLNDLIGGSY

CWVVQLFSIVCLRSGNYIPTSSGSTCRLDEDEAGLTWDAICFVFLLIMLRVFKSWYFQHVVMELYVENKLASRGAELINT

NLIKRVAEMKSRERDTLASIKRKMERIRLRQARLQSKAIKESEDHYIVIRSGDYYMFEDDSDEDEASSVDVQERDDQRDE

EEDAGKVGPLQILDAAFKGGTKEALEVAEGRRISQRSESEEDAECSSSRARVSRLSGPTTPPGTEPDIIEEMPEGELPSD

EDEDEEEEEETKAESACNKLVSWIKLGWAIFCGLLDYLIVVLHNISRENRSVSRILDREMEEEKRRLRAQEQNMQANADE

DDLQSSPHPSLTRKATDISIHLEQQERDISMLRQEQDIDAMKDAEKEFQRGMPRLLQLVHALIEVLLSRSDLLCYFAMIL

NVLVNASILALPYPFCVFLWALLSVPRPTKTYWVTIITYTEAIVVVKYLFQFGFFPWNDGLPQDGPFWPPRIIGIEKKSS

YAVFDLILLFCLFVHRSVMKRYGLWKDAGDIEEDFAAAEQKVQSRPNSSSTSPMDVPSITFTDADVEGAAANAAAIAAAN

AAAIVAANASDTSMEVTGSDDSSPKHKKHKKGLLRACVSPFLEFYQQMTNPKYNAVTDVYATIFLCDFVTFVIVVFGYWA

FGPAEAGGGTETVASVIQENKVPPLFLAMLLVQFLLIIVDRALYLRKQVFGKFLFQIFLVILVHVWMFFILPFVTNRKFQ

DNLPAQLWYFTKCIYFGFSAYQIRSGYPTRILGNFLTKKFNYINLFLFQGFLLIPFLLELRALMDWMWTDTTLALTSWLQ

MEDIYANIFVLKCWRRAEDTYPTPRGVKRRALVKYGVGGLLLFLLIFIIWFPLLLFSLSSTIFQPNPPIDCTVEMKLGSY

QPIFTMSAQQSNLREFTPQKYAELKTTFSGDPTATSFLSGYQANDIYEARIYGISTATWDISPPSEQALTSSLLKDPTVN

LYVTISFKREPSDSYSSGAVSNQFTKILHSDSDPDMKIRRNLAHMLNGTYESTDPIEIPHVYPPYMVVKASSKPEPADPL

LGQNASKWANSTFRLRNQPPAIQWWELESKKIEDNQVVVSENLTILTFNERVAPGAFSFFTSYGIIGLYFSFIFVIGSFT

RLHTTGLVQDIMYKHLPFVDRIHALCLDICLVRQFLEFRLEEELVMGLYISVVIVIGQFVRIFFSGTSNKIMFEQLPSVE

RLYKLCLDIYMVRETVVGLYVAVVLVIGQFLRVYTTGTYPQIAFEWLPNVDRIYKLIMEIYLVRECCEFRIEENLLAKLI

FLYRNGSMRFQYTKAKID

> Lottia gigantea XP_009059570.1 124598

LIGASGIILPSVLSAVYFVSFLTIATLWALYYSMGRKFAFYRGFLLIFSGLHILTLHLYQFQFFQDGVPHDSLVARLLGL

TGIIKTDCNKPWILSIHSDVKWEYYVNPGILLLLYWHLVDDGENQNYSSIEATNKDEPTGATPRSNDETDEEDAWSITFH

SWLTFVLLLGACILWMLPDSRRACLIISPLITIYGVCLLIAQFIFSLNLTEDELPRQTGDLRYREIGLMDLECYELALQV

FFVLFFWLTMRQYMRERQTTQNTQQGLQLHHVQRQTSTLSDFITRYVWLILCKYWIFVCVGMLLLISIQDVVVYRIIYMF

LFLYFILAFQFSYVVWRKTMFLFWWVVIIYSMAVLIMIYTYQFDAFPQYWSDGTGFTNATLSDIGLERYDTAGLFMKLLT

PTAFLIIVILQIHYFHKPFLAYSSLTRYKKDEPVADPNVTDYTTDETGNTTTDSEHHLNRRTKRNKTWAVVSAFLWRLSE

VHFFKIVVLIIMLVSVTEVSAISAIYVLLMAVFIPLSKCHIVLSHIVQFWTSLVLLAKMLYQLRLVKKDYWITNCTSPMD

VGNTSLSSMLQNGTEVDNALWVGLAKISKLDVTISYYISNYLLILLLVAFESIVRYHQIQRYRHEQIVKPKTGIVFPEIK

REDADKGLKNCIMYFFNYGFYKFGLEICYITTAATISIRADAYAVLYAIFLGILLLLSRRGNSRIWPLYTIVLAILLPLQ

YLSCVGFPLGLCLKYPWKHETTLDQNLEQWLFLPDYAYPPHPFKLLADFIQLLFVCLQWRVFRIENRQEVQDEGGDNQDI

LPEVEANTPIPALYNDFTASTLSYLDVIKYFVFGYMFWVTLAIVFLTGLTRINLFSMGYVIAVFCFMWYGQEFLLKPLRK

LLRGWNILLGYTFCVLFAKAALQLVGCVYIKHLYENQCWLIQLLGINCLMSDISINPPIHVKCVIEEDNTGLAWDVVCFV

FLLLQRRIYSSHNFRHVVDEIEAQHRLASRYVFNFF

> Crassostrea gigas 1 XP_011438607.1-

MSSKVARYISYLLFRLLLPVVLLAGCALRYNLVSFLYLLFLLGLPLIPAPSALTVRGITGVYLILLIVLGGLATLAHAIF

HIVLAAIPEPYGYSFSNCSSNEKIARLIAVERLDGVPFIHILRLVVLDVVVLAVAILVFIVCYKVFNPSRDTLSSEDVPS

LQSVTRRRRASRLHNFLLFVGKFIDVLFLAACGIIVPSLASAAYFLSFLYIATWWSLYRGLGKKFSIFRICVLIWSGLHL

LFLHLYQMQLFQEDWFPSRTSLWARILGLTAVIKTDCGKPWSVEFHEPSNWPLFVNPPLLLALYISVATETRYWRNSKRE

DVVDAETSSPKKKRRRNPKPSEREVKGGNSLVNAGENQSYETFSEEPARQRNKDDSPTMLDSDEEDGGSTRSKPKKTKPH

VKRSPVVSLFVYIMKQSYVLSLIAMMAWSITYHSWLTFVLLLAACFIWMVPQSRKACLVCSPAVVLYGEVLLIIQFVYGM

NLKELPEESSGIKLDEIGLKKFKYPCLQLALQILFTSMFWLTMRQFIRERKLSGSSDNSQVPMETLSSEGVSPGSWFKGW

IRLREMSDGEEDVWDITQSPKYIPSLDEVDGYDGQTVKRIGHSEAVRTIGVYVWNLLSKYWIFVCTGMMLLISIQEVVVY

RIIYLILFLLFMLTFQMSYTLWRASMYVFWWVIIIYSMGILIILYTYQFQDFPSYWQNNTGLSDEILSDIGLEQFNTATL

FVKLLTPTSFLILIILQVHYFHATFLRLSDIKHVKNEDKKEDQPATNTMTDTAGETTDSEPPIIKKGLRKKVKLYILTVW

HKCSEVWAEVSTVLWRVLEVHFVKVVLFTIFMVCVSELSAIGGVFVIILGVFMPITRGWTVMLHVCRIWVSIAMVAKMIY

QLSIIDKEYWNSNCTITNAGVNSSTTPAPVTNITNLTDLLPAETVINPFGNGTVNNAKWVGLDKEGVLSYYLRVSTRDDT

YCYLIN

> Crassostrea gigas 2 EKC42880.1

MGILLGVYVWNLLSKYWIFVCTGMMLLISIQEVVVYRIIYLILFLLFMLTFQMSYTLWRASMYVFWWVIIIYSMGILIIL

YTYQFQDFPSYWQNNTGLSDEILSDIGLEQFNTATLFVKLLTPTSFLILIILQVHYFHATFLRLSDIKHVKNEDKKEDQP

ATNTMTDTAGETTDSEPPIIKKGLRKKVKLYILTVWHKCSEVWAEVSTVLWRVLEVHFVKVVLFTIFMVCVSELSAIGGV

FVIILGVFMPITRGWTVMLHVCRIWVSIAMVAKMIYQLSIIDKEYWNSNCTITNAGVNSSTTPAPVTNITNLTDLLPAET

VINPFGNGTVNNAKWVGLDKEGVLSYYLRLFVAEYPWKFTTETGKNLETWLYLPSYFDPPDVNKLIGDFFLLLFVCLQWQ

VFRTEAHVHSDTSHSYGGGSNGDILEEVEANMPIPVEDFTTIHESYLDVVKSGFFSYMYWVTLYIIFLAGTTRINLFSMG

YIIGALCFMWYGQEFILKPLRKIRRSWNTFLCYTFFVIFLKASLQLLGCVYIDYLYDNQCWLIQLLGLTCLSPSKTYSPG

EYLMGAELINHILVTRVQKQREEEKQVLENIKKKMDILRHKRIQGSNKAFTEPDEHFQAIRSGDYYLFEDESEAHTEDPT

SITIGVDSEEEDGDKADPLKLISTAIESGAKAAVEQGENEDEEGNETGSEVKEDKSDSKLEKILDILKLIKMLLESLVDW

MIEKFKRVAYNHRKVAETLEEEMKAQKALIQRNKTVPVIKDTTVSLQLEDDGNEGPSGASGERVDFTDISIVDTEAPDLD

DLDGNVQSKPSTKFENKKNKVYLLLEAAYYALVSRSELVCYFLMILNQILYCSLLSLPLPLMVFLWGMLSVPRPSKMFWI

TAITYTEAVVVIKYLFQFGFFSWNNNTDIETDPFWPPRIIGIEKKDGFVTADLILLLALFIHRSILKRYGLWKDADATLA

DVDRAEEIISAPTTPIQEEGARSFESSVRNNSKEGQVGSSTSQESDGDDESGPGLQMVLGPFKKFYSQLTDPKYNATTDV

YATMFFCDFINFLILVFGYWAFGPEQTSGGDSVTEYITEDRIPVPFLIMLVTQFILIVIDRALYLRKNILGKFIFQILLV

ILIHVWLFFVLPYVTKRKFSENVPAQMFYFFKCLYFGLSAYQIRSSYPTRILGNFLTKKYNYINLFLFKGFLAIPFLLEL

RVLMDWIWTDSTLAIGSWLQMEDIYANIYVLKCWREAERNYPTPRSRKKGSLIKYGVGGLLLVLIIFIIWFPLVIFSFAN

TVYISNPPVVVTASIAIGGFQPLFKVQAQQQSIASLTTAQYNDLLTYFNEREMKSAVSNLNKYEPQDITIIKLNGKSTSV

WGISPPSKQELIQFLNNTVAAKIEFETTFTRKPTSSDASQSLTSHLMDVLDDVTRIKLIQLLKEPETSAPVEVKNLFSRF

FRLSKKTTSVLNFIPKSNVSMHLSVTNVTEWWNVNETLSTGVPCPEPQDKKCGPSATELTLITLNDRAAPAGFSFISGYG

IIGLYVTFILLIGRILRLSTTGLAERITYLELPYVDNILQLCLDVYLVREMHEFKLEEDLYAKLIFVYRSSETRIKWTRL

PKDLIITDAKKND

> Homo1 sapiens NP_001136336.2 P1

MEPHVLGAVLYWLLLPCALLAACLLRFSGLSLVYLLFLLLLPWFPGPTRCGLQGHTGRLLRALLGLSLLF

LVAHLALQICLHIVPRLDQLLGPSCSRWETLSRHIGVTRLDLKDIPNAIRLVAPDLGILVVSSVCLGICG

RLARNTRQSPHPRELDDDERDVDASPTAGLQEAATLAPTRRSRLAARFRVTAHWLLVAAGRVLAVTLLAL

AGIAHPSALSSVYLLLFLALCTWWACHFPISTRGFSRLCVAVGCFGAGHLICLYCYQMPLAQALLPPAGI

WARVLGLKDFVGPTNCSSPHALVLNTGLDWPVYASPGVLLLLCYATASLRKLRAYRPSGQRKEAAKGYEA

RELELAELDQWPQERESDQHVVPTAPDTEADNCIVHELTGQSSVLRRPVRPKRAEPREASPLHSLGHLIM

DQSYVCALIAMMVWSITYHSWLTFVLLLWACLIWTVRSRHQLAMLCSPCILLYGMTLCCLRYVWAMDLRP

ELPTTLGPVSLRQLGLEHTRYPCLDLGAMLLYTLTFWLLLRQFVKEKLLKWAESPAALTEVTVADTEPTR

TQTLLQSLGELVKGVYAKYWIYVCAGMFIVVSFAGRLVVYKIVYMFLFLLCLTLFQVYYSLWRKLLKAFW

WLVVAYTMLVLIAVYTFQFQDFPAYWRNLTGFTDEQLGDLGLEQFSVSELFSSILVPGFFLLACILQLHY

FHRPFMQLTDMEHVSLPGTRLPRWAHRQDAVSGTPLLREEQQEHQQQQQEEEEEEEDSRDEGLGVATPHQ

ATQVPEGAAKWGLVAERLLELAAGFSDVLSRVQVFLRRLLELHVFKLVALYTVWVALKEVSVMNLLLVVL

WAFALPYPRFRPMASCLSTVWTCVIIVCKMLYQLKVVNPQEYSSNCTEPFPNSTNLLPTEISQSLLYRGP

VDPANWFGVRKGFPNLGYIQNHLQVLLLLVFEAIVYRRQEHYRRQHQLAPLPAQAVFASGTRQQLDQDLL

GCLKYFINFFFYKFGLEICFLMAVNVIGQRMNFLVTLHGCWLVAILTRRHRQAIARLWPNYCLFLALFLL

YQYLLCLGMPPALCIDYPWRWSRAVPMNSALIKWLYLPDFFRAPNSTNLISDFLLLLCASQQWQVFSAER

TEEWQRMAGVNTDRLEPLRGEPNPVPNFIHCRSYLDMLKVAVFRYLFWLVLVVVFVTGATRISIFGLGYL

LACFYLLLFGTALLQRDTRARLVLWDCLILYNVTVIISKNMLSLLACVFVEQMQTGFCWVIQLFSLVCTV

KGYYDPKEMMDRDQDCLLPVEEAGIIWDSVCFFFLLLQRRVFLSHYYLHVRADLQATALLASRGFALYNA

ANLKSIDFHRRIEEKSLAQLKRQMERIRAKQEKHRQGRVDRSRPQDTLGPKDPGLEPGPDSPGGSSPPRR

QWWRPWLDHATVIHSGDYFLFESDSEEEEEAVPEDPRPSAQSAFQLAYQAWVTNAQAVLRRRQQEQEQAR

QEQAGQLPTGGGPSQEVEPAEGPEEAAAGRSHVVQRVLSTAQFLWMLGQALVDELTRWLQEFTRHHGTMS

DVLRAERYLLTQELLQGGEVHRGVLDQLYTSQAEATLPGPTEAPNAPSTVSSGLGAEEPLSSMTDDMGSP

LSTGYHTRSGSEEAVTDPGEREAGASLYQGLMRTASELLLDRRLRIPELEEAELFAEGQGRALRLLRAVY

QCVAAHSELLCYFIIILNHMVTASAGSLVLPVLVFLWAMLSIPRPSKRFWMTAIVFTEIAVVVKYLFQFG

FFPWNSHVVLRRYENKPYFPPRILGLEKTDGYIKYDLVQLMALFFHRSQLLCYGLWDHEEDSPSKEHDKS

GEEEQGAEEGPGVPAATTEDHIQVEARVGPTDGTPEPQVELRPRDTRRISLRFRRRKKEGPARKGAAAIE

AEDREEEEGEEEKEAPTGREKRPSRSGGRVRAAGRRLQGFCLSLAQGTYRPLRRFFHDILHTKYRAATDV

YALMFLADVVDFIIIIFGFWAFGKHSAATDITSSLSDDQVPEAFLVMLLIQFSTMVVDRALYLRKTVLGK

LAFQVALVLAIHLWMFFILPAVTERMFNQNVVAQLWYFVKCIYFALSAYQIRCGYPTRILGNFLTKKYNH

LNLFLFQGFRLVPFLVELRAVMDWVWTDTTLSLSSWMCVEDIYANIFIIKCSRETEKKYPQPKGQKKKKI

VKYGMGGLIILFLIAIIWFPLLFMSLVRSVVGVVNQPIDVTVTLKLGGYEPLFTMSAQQPSIIPFTAQAY

EELSRQFDPQPLAMQFISQYSPEDIVTAQIEGSSGALWRISPPSRAQMKRELYNGTADITLRFTWNFQRD

LAKGGTVEYANEKHMLALAPNSTARRQLASLLEGTSDQSVVIPNLFPKYIRAPNGPEANPVKQLQPNEEA

DYLGVRIQLRREQGAGATGFLEWWVIELQECRTDCNLLPMVIFSDKVSPPSLGFLAGYGIMGLYVSIVLV

IGKFVRGFFSEISHSIMFEELPCVDRILKLCQDIFLVRETRELELEEELYAKLIFLYRSPETMIKWTREK

E

> Homo2 sapiens NP_071351.2 P2

MASEVVCGLIFRLLLPICLAVACAFRYNGLSFVYLIYLLLIPLFSEPTKTTMQGHTGRLLKSLCFISLSF

LLLHIIFHITLVSLEAQHRIAPGYNCSTWEKTFRQIGFESLKGADAGNGIRVFVPDIGMFIASLTIWLLC

RNIVQKPVTDEAAQSNPEFENEELAEGEKIDSEEALIYEEDFNGGDGVEGELEESTKLKMFRRLASVASK

LKEFIGNMITTAGKVVVTILLGSSGMMLPSLTSSVYFFVFLGLCTWWSWCRTFDPLLFSCLCVLLAIFTA

GHLIGLYLYQFQFFQEAVPPNDYYARLFGIKSVIQTDCSSTWKIIVNPDLSWYHHANPILLLVMYYTLAT

LIRIWLQEPLVQDEGTKEEDKALACSPIQITAGRRRSLWYATHYPTDERKLLSMTQDDYKPSDGLLVTVN

GNPVDYHTIHPSLPMENGPGKADLYSTPQYRWEPSDESSEKREEEEEEKEEFEEERSREEKRSIKVHAMV

SVFQFIMKQSYICALIAMMAWSITYHSWLTFVLLIWSCTLWMIRNRRKYAMISSPFMVVYGNLLLILQYI

WSFELPEIKKVPGFLEKKEPGELASKILFTITFWLLLRQHLTEQKALQEKEALLSEVKIGSQENEEKDEE

LQDIQVEGEPKEEEEEEAKEEKQERKKVEQEEAEEEDEQDIMKVLGNLVVAMFIKYWIYVCGGMFFFVSF

EGKIVMYKIIYMVLFLFCVALYQVHYEWWRKILKYFWMSVVIYTMLVLIFIYTYQFENFPGLWQNMTGLK

KEKLEDLGLKQFTVAELFTRIFIPTSFLLVCILHLHYFHDRFLELTDLKSIPSKEDNTIYRLAHPEGSLP

DLTMMHLTASLEKPEVRKLAEPGEEKLEGYSEKAQKGDLGKDSEESEEDGEEEEESEEEEETSDLRNKWH

LVIDRLTVLFLKFLEYFHKLQVFMWWILELHIIKIVSSYIIWVSVKEVSLFNYVFLISWAFALPYAKLRR

LASSVCTVWTCVIIVCKMLYQLQTIKPENFSVNCSLPNENQTNIPFNELNKSLLYSAPIDPTEWVGLRKS

SPLLVYLRNNLLMLAILAFEVTIYRHQEYYRGRNNLTAPVSRTIFHDITRLHLDDGLINCAKYFINYFFY

KFGLETCFLMSVNVIGQRMDFYAMIHACWLIAVLYRRRRKAIAEIWPKYCCFLACIITFQYFICIGIPPA

PCRDYPWRFKGASFNDNIIKWLYFPDFIVRPNPVFLVYDFMLLLCASLQRQIFEDENKAAVRIMAGDNVE

ICMNLDAASFSQHNPVPDFIHCRSYLDMSKVIIFSYLFWFVLTIIFITGTTRISIFCMGYLVACFYFLLF

GGDLLLKPIKSILRYWDWLIAYNVFVITMKNILSIGACGYIGTLVHNSCWLIQAFSLACTVKGYQMPAAN

SPCTLPSGEAGIIWDSICFAFLLLQRRVFMSYYFLHVVADIKASQILASRGAELFQATIVKAVKARIEEE

KKSMDQLKRQMDRIKARQQKYKKGKERMLSLTQEPGEGQDMQKLSEEDDEREADKQKAKGKKKQWWRPWV

DHASMVRSGDYYLFETDSEEEEEEELKKEDEEPPRRSAFQFVYQAWITDPKTALRQRHKEKKRSAREERK

RRRKGSKEGPVEWEDREDEPIKKKSDGPDNIIKRIFNILKFTWVLFLATVDSFTTWLNSISREHIDISTV

LRIERCMLTREIKKGNVPTRESIHMYYQNHIMNLSRESGLDTIDEHPGAASGAQTAHRMDSLDSHDSISS

EPTQCTMLYSRQGTTETIEEVEAEQEEEAGSTAPEPREAKEYEATGYDVGAMGAEEASLTPEEELTQFST

LDGDVEAPPSYSKAVSFEHLSFGSQDDSAGKNRMAVSPDDSRTDKLGSSILPPLTHELTASELLLKKMFH

DDELEESEKFYVGQPRFLLLFYAMYNTLVARSEMVCYFVIILNHMVSASMITLLLPILIFLWAMLSVPRP

SRRFWMMAIVYTEVAIVVKYFFQFGFFPWNKNVEVNKDKPYHPPNIIGVEKKEGYVLYDLIQLLALFFHR

SILKCHGLWDEDDMTESGMAREESDDELSLGHGRRDSSDSLKSINLAASVESVHVTFPEQQTAVRRKRSG

SSSEPSQRSSFSSNRSQRGSTSTRNSSQKGSSVLSIKQKGKRELYMEKLQEHLIKAKAFTIKKTLEIYVP

IKQFFYNLIHPEYSAVTDVYVLMFLADTVDFIIIVFGFWAFGKHSAAADITSSLSEDQVPGPFLVMVLIQ

FGTMVVDRALYLRKTVLGKVIFQVILVFGIHFWMFFILPGVTERKFSQNLVAQLWYFVKCVYFGLSAYQI

RCGYPTRVLGNFLTKSYNYVNLFLFQGFRLVPFLTELRAVMDWVWTDTTLSLSSWICVEDIYAHIFILKC

WRESEKRYPQPRGQKKKKVVKYGMGGMIIVLLICIVWFPLLFMSLIKSVAGVINQPLDVSVTITLGGYQP

IFTMSAQQSQLKVMDQQSFNKFIQAFSRDTGAMQFLENYEKEDITVAELEGNSNSLWTISPPSKQKMIHE

LLDPNSSFSVVFSWSIQRNLSLGAKSEIATDKLSFPLKNITRKNIAKMIAGNSTESSKTPVTIEKIYPYY

VKAPSDSNSKPIKQLLSENNFMDITIILSRDNTTKYNSEWWVLNLTGNRIYNPNSQALELVVFNDKVSPP

SLGFLAGYGIMGLYASVVLVIGKFVREFFSGISHSIMFEELPNVDRILKLCTDIFLVRETGELELEEDLY

AKLIFLYRSPETMIKWTREKTN

> Mus1 musculus NP_001032375.1 P1

MEPHVLGAGLYWLLLPCTLLAASLLRFNALSLVYLLFLLLLPWLPGPSRHSIPGHTGRLLRALLCLSLLF

LVAHLAFQICLHTVPHLDQFLGQNGSLWVKVSQHIGVTRLDLKDIFNTTRLVAPDLGVLLASSLCLGLCG

RLTRKARQSRRTQELDDDDDDDDDDEDIDAAPAVGLKGAPALATKRRLWLASRFRVTAHWLLMTSGRTLV

IVLLALAGIAHPSAFSSVYLVVFLAICTWWSCHFPLSPLGFNTLCVMVSCFGAGHLICLYCYQTPFIQDM

LPPGNIWARLFGLKNFVDLPNYSSPNALVLNTKHAWPIYVSPGILLLLYYTATSLLKLHKSCPSELRKET

PREDEEHELELDHLEPEPQARDATQGEMPMTTEPDLDNCTVHVLTSQSPVRQRPVRPRLAELKEMSPLHG

LGHLIMDQSYVCALIAMMVWSIMYHSWLTFVLLLWACLIWTVRSRHQLAMLCSPCILLYGLTLCCLRYVW

AMELPELPTTLGPVSLHQLGLEHTRYPCLDLGAMLLYLLTFWLLLRQFVKEKLLKKQKVPAALLEVTVAD

TEPTQTQTLLRSLGELVTGIYVKYWIYVCAGMFIVVSFAGRLVVYKIVYMFLFLLCLTLFQVYYTLWRKL

LRVFWWLVVAYTMLVLIAVYTFQFQDFPTYWRNLTGFTDEQLGDLGLEQFSVSELFSSILIPGFFLLACI

LQLHYFHRPFMQLTDLEHVPPPGTRHPRWAHRQDAVSEAPLLEHQEEEEVFREDGQSMDGPHQATQVPEG

TASKWGLVADRLLDLAASFSAVLTRIQVFVRRLLELHVFKLVALYTVWVALKEVSVMNLLLVVLWAFALP

YPRFRPMASCLSTVWTCIIIVCKMLYQLKIVNPHEYSSNCTEPFPNNTNLQPLEINQSLLYRGPVDPANW

FGVRKGYPNLGYIQNHLQILLLLVFEAVVYRRQEHYRRQHQQAPLPAQAVCADGTRQRLDQDLLSCLKYF

INFFFYKFGLEICFLMAVNVIGQRMNFMVILHGCWLVAILTRRRREAIARLWPNYCLFLTLFLLYQYLLC

LGMPPALCIDYPWRWSKAIPMNSALIKWLYLPDFFRAPNSTNLISDFLLLLCASQQWQVFSAERTEEWQR

MAGINTDHLEPLRGEPNPIPNFIHCRSYLDMLKVAVFRYLFWLVLVVVFVAGATRISIFGLGYLLACFYL

LLFGTTLLQKDTRAQLVLWDCLILYNVTVIISKNMLSLLSCVFVEQMQSNFCWVIQLFSLVCTVKGYYDP

KEMMTRDRDCLLPVEEAGIIWDSICFFFLLLQRRIFLSHYFLHVSADLKATALQASRGFALYNAANLKSI

NFHRQIEEKSLAQLKRQMKRIRAKQEKYRQSQASRGQLQSKDPQDPSQEPGPDSPGGSSPPRRQWWRPWL

DHATVIHSGDYFLFESDSEEEEEALPEDPRPAAQSAFQMAYQAWVTNAQTVLRQRRERARQERAEQLASG

GDLNPDVEPVDVPEDEMAGRSHMMQRVLSTMQFLWVLGQATVDGLTRWLRAFTKHHRTMSDVLCAERYLL

TQELLRVGEVRRGVLDQLYVGEDEATLSGPMETRDGPSTASSGLGAEEPLSSMTDDTSSPLSTGYNTRSG

SEEIVTDAGDLQAGTSLHGSQELLANARTRMRTASELLLDRRLHIPELEEAERFEAQQGRTLRLLRAGYQ

CVAAHSELLCYFIIILNHMVTASAASLVLPVLVFLWAMLTIPRPSKRFWMTAIVFTEVMVVTKYLFQFGF

FPWNSYVVLRRYENKPYFPPRILGLEKTDSYIKYDLVQLMALFFHRSQLLCYGLWDHEEDRYPKDHCRSS

VKDREAKEEPEAKLESQSETGTGHPKEPVLAGTPRDHIQGKGSIRSKDVIQDPPEDLKPRHTRHISIRFR

RRKETPGPKGTAVMETEHEEGEGKETTERKRPRHTQEKSKFRERMKAAGRRLQSFCVSLAQSFYQPLQRF

FHDILHTKYRAATDVYALMFLADIVDIIIIIFGFWAFGKHSAATDIASSLSDDQVPQAFLFMLLVQFGTM

VIDRALYLRKTVLGKLAFQVVLVVAIHIWMFFILPAVTERMFSQNAVAQLWYFVKCIYFALSAYQIRCGY

PTRILGNFLTKKYNHLNLFLFQGFRLVPFLVELRAVMDWVWTDTTLSLSNWMCVEDIYANIFIIKCSRET

EKKYPQPKGQKKKKIVKYGMGGLIILFLIAIIWFPLLFMSLIRSVVGVVNQPIDVTVTLKLGGYEPLFTM

SAQQPSIVPFTPQAYEELSQQFDPYPLAMQFISQYSPEDIVTAQIEGSSGALWRISPPSRAQMKQELYNG

TADITLRFTWNFQRDLAKGGTVEYTNEKHTLELAPNSTARRQLAQLLEGRPDQSVVIPHLFPKYIRAPNG

PEANPVKQLQPDEEEDYLGVRIQLRREQVGTGASGEQAGTKASDFLEWWVIELQDCKADCNLLPMVIFSD

KVSPPSLGFLAGYGIVGLYVSIVLVVGKFVRGFFSEISHSIMFEELPCVDRILKLCQDIFLVRETRELEL

EEELYAKLIFLYRSPETMIKWTRERE

>Mus2 musculus NP_001034574.4 P2

MASEVVCGLIFRLLLPICLAVACAFRYNGLSFVYLIYLLLIPLFSEPTKATMQGHTGRLLQSLCITSLSF

LLLHIIFHITLASLEAQHRITPAYNCSTWEKTFRQIGFESLKGADAGNGIRVFVPDIGMFIASLTIWLVC

RTIVKKPDTEEIAQLNSECENEELAGGEKMDSEEALIYEEDLDGEEGMEGELEESTKLKILRRFASVASK

LKEFIGNMITTAGKVVVTILLGSSGMMLPSLTSAVYFFVFLGLCTWWSWCRTFDPLLFGCLCVLLAIFTA

GHLIGLYLYQFQFFQEAVPPNDYYARLFGIKSVIQTDCASTWKIIVNPDLSWYHHANPILLLVMYYTLAT

LIRIWLQEPLVQEEMAKEDEGALDCSSNQNTAERRRSLWYATQYPTDERKLLSMTQDDYKPSDGLLVTVN

GNPVDYHTIHPSLPIENGPAKTDLYTTPQYRWEPSEESSEKKEEEEDKREDSEGEGSQEEKRSVRMHAMV

AVFQFIMKQSYICALIAMMAWSITYHSWLTFVLLIWSCTLWMIRNRRKYAMISSPFMVVYANLLLVLQYI

WSFELPEIKKVPGFLEKKEPGELASKILFTITFWLLLRQHLTEQKALREKEALLSEVKIGSQELEEKEDE

ELQDVQVEGEPTEKEEEEEEEIKEERHEVKKEEEEEVEEDDDQDIMKVLGNLVVALFIKYWIYVCGGMFF

FVSFEGKIVMYKIIYMVLFLFCVALYQVHYEWWRKILKYFWMSVVIYTMLVLIFIYTYQFENFPGLWQNM

TGLKKEKLEDLGLKQFTVAELFTRIFIPTSFLLVCILHLHYFHDRFLELTDLKSIPSKEDNTIYSHAKVN

GRVYLIINRLAHPEGSLPDLAIMNMTASLDKPEVQKLAESGEERPEECVKKTEKGEAGKDSDESEEEEDE

EEESEEEESSDLRNKWHLVIDRLTVLFLKFLEYFHKLQVFMWWILELHIIKIVSSYIIWVTVKEVSLFNY

VFLISWAFALPYAKLRRAASSVCTVWTCVIIVCKMLYQLQTIKPENFSVNCSLPNENQTNIPLHELNKSL

LYSAPVDPTEWVGLRKSSPLLVYLRNNLLMLAILAFEVTVYRHQEYYRGRNNLTAPVSKTIFHDITRLHL

DDGLINCAKYFVNYFFYKFGLETCFLMSVNVIGQRMDFYAMIHACWLIGVLYRRRRKAIAEVWPKYCCFL

ACIITFQYFVCIGIPPAPCRDYPWRFKGAYFNDNIIKWLYFPDFIVRPNPVFLVYDFMLLLCASLQRQIF

EDENKAAVRIMAGDNVEICMNLDAASFSQHNPVPDFIHCRSYLDMSKVIIFSYLFWFVLTIIFITGTTRI

SIFCMGYLVACFYFLLFGGDLLLKPIKSILRYWDWLIAYNVFVITMKNILSIGACGYIGALVRNSCWLIQ

AFSLACTVKGYQMPEDDSRCKLPSGEAGIIWDSICFAFLLLQRRVFMSYYFLHVVADIKASQILASRGAE

LFQATIVKAVKARIEEEKKSMDQLKRQMDRIKARQQKYKKGKERMLSLTQESGEGQDIQKVSEEDDEREA

DKQKAKGKKKQWWRPWVDHASMVRSGDYYLFETDSEEEEEEELKKEDEEPPRKSAFQFVYQAWITDPKTA

LRQRRKEKKKLAREEQKERRKGSGDGPVEWEDREDEPVKKKSDGPDNIIKRIFNILKFTWVLFLATVDSF

TTWLNSISREHIDISTVLRIERCMLTREIKKGNVPTRESIHMYYQNHIMNLSRESGLDTIDEHSGAGSRA

QAAHRMDSLDSRDSISSCYTEATLLISRQSTLDDLDGQDPVPKTSERARPRLRKMFSLDMSSSSADSGSV

ASSEPTQCTMLYSRQGTTETIEEVEAEAEEEVVEGLEPELHDAEEKEYAAEYEAGVEEISLTPDEELPQF

STDDCDVEAPPSYSKAVSFEHLSFASQDDSGAKNHMVVSPDDSRTDKLESSILPPLTHELTASDLLMSKM

FHDDELEESEKFYVDQPRFLLLFYAMYNTLVARSEMVCYFVIILNHMTSASIITLLLPILIFLWAMLSVP

RPSRRFWMMAIVYTEVAIVVKYFFQFGFFPWNKDLEIYKERPYFPPNIIGVEKKEGYVLYDLIQLLALFF

HRSILKCHGLWDEDDIVDSNTDKEGSDDELSLDQGRRGSSDSLKSINLAASVESVHVTFPEQPAAIRRKR

SCSSSQISPRSSFSSNRSKRGSTSTRNSSQKGSSVLSLKQKSKRELYMEKLQEHLIKAKAFTIKKTLQIY

VPIRQFFYDLIHPDYSAVTDVYVLMFLADTVDFIIIVFGFWAFGKHSAAADITSSLSEDQVPGPFLVMVL

IQFGTMVVDRALYLRKTVLGKVIFQVILVFGIHFWMFFILPGVTERKFSQNLVAQLWYFVKCVYFGLSAY

QIRCGYPTRVLGNFLTKSYNYVNLFLFQGFRLVPFLTELRAVMDWVWTDTTLSLSSWICVEDIYAHIFIL

KCWRESEKRYPQPRGQKKKKAVKYGMGGMIIVLLICIVWFPLLFMSLIKSVAGVINQPLDVSVTITLGGY

QPIFTMSAQQSQLKVMDNSKYNEFLKSFGPNSGAMQFLENYEREDVTVAELEGNSNSLWTISPPSKQKMI

QELTDPNSCFSVVFSWSIQRNMTLGAKAEIATDKLSFPLAVATRNSIAKMIAGNDTESSNTPVTIEKIYP

YYVKAPSDSNSKPIKQLLSENNFMNITIILFRDNVTKSNSEWWVLNLTGSRIFNQGSQALELVVFNDKVS

PPSLGFLAGYGIMGLYASVVLVIGKFVREFFSGISHSIMFEELPNVDRILKLCTDIFLVRETGELELEED

LYAKLIFLYRSPETMIKWTREKTN

> Gallus1 gallus XP_414209.4 P1

MERRALCAGLYWLLLPLALLAACLFRFNALSLVYLLYLLLLPWFPGPADVSGEVTASRARGPSVGFGTIF

RGCAPSLLQVPVPPLWFPRGWAAPRGSLVWLIIVWRNYEFRVGMLAPLGAAWGDGCPSLAGHAGRLLKAL

LGTSLLFLLAHFVFQICLYTLPALDQLLGPSCEYPSAWCHVPPESPRWWHLGAGPHLPHAMGLLWGRCHP

NTSVLPRRQQLGGPRPAHRGHQVRLDLGDLPNSVRLVAPDVGILLVSSLCLCLCHRLVPKGSAAARRPES

QQPPEQGEEQDVERRGGTAGGDAPLSARLRVTAHWVLWAAGKGLAILLLALAGITLPSASSSVYYVLFVG

LCTWWACHLPSSRAAFDALCVLVGLYAAAHLLCLYAYQAPFVQGVFPPPTIWARVFGFKDIILYSNCSRP

NALQLNTDHPWPVYANPGILLLLYYTVATLLKLRRLDARVRGCGAACTPMAQGVWWSGCCSRGWDPPRLQ

LTLCSCREQQCQHSPQRGIWWSWRAGPRTAMAWLKTPSPCCPPALGSGARRTAPSTSWVMPRHWVSPPQC

CPIPALQWGRGSQLLFPWPGRDRPAERLPLQALWHVIMNQSYVCALIAMMVWSITYHSWLTFVLLLWSCL

IWIVRSRHHFAMLCSPFLLLYGIALCSLQYVWGMDLGPELPTRVGLMSLEQLGLVRPKYPCLDLGAKLLL

TLTFWLLVRQFIKEKLLTRTCPATPLLEVTISDTGEGPSVLLGAVPMPPTVPAVPCLAEPGQQRDVLKAL

GALVRDFYAKYWICVCAGMFIVVSFAGRLVVYKIVYMFLFLLCLTLFQVYYTLWRKVLKGFWWLVVAYTM

LVLIAVYTFQFEDFPMYWRNLTGLTNEQLGDLGLEQFSVSELFSSTLIPGFFLLACILQLHYFHRPFMHI

TDLEHIPASEPQPCHIPRPEELHGSRLLRDAVVAESARTEGGESDTASQGTARGGASLGLGMGWDFLQAG

ESIRDGSECPLCLGEYRAGLGCLLSRGXAEGXDGLGCSLKSGGYGGGLERSPKSVRGQKDMGMGLGCPLI

FGGRRNGCEYPPSQVEARRIQGRVRVSSKSGRGGYRDAPGGPSGQERDGSDYRGVLGCPEPGRVRGWIRA

SPKPGRGGGRVGVPSKPTTLCVLTAPSKWGLVLERLIVLGWTFSDTLTRGQVFVGRLLELHILKLVALYT

VWVALQEVSLMNFLLVLLWAFAMPYCRFRHMASCLSTVWTCIIIVCKMLYQLKIVDPSEYSSNCTQPLLN

GTNLSPEEMGNSTLYRGPVDPANWFGIRKGFPNLGYIQNHLLVLLLLVLEAVVYRRQEYYRKQHQLVVPI

TETIFEDVSREQLDHGLVTCAKYFLNYFYYKFGLEICFLMTVNVIGQRMNFMVILHGCWLVVILTRRRRA

AIARLWPKYCLFLVVFLLYQYLLCVGMPPALCMGKGYGEWAGGRWGGRDVVWCCSASPDYPWRWSQSLPM

NSALIKWLYLPDFFVAPKSTNLINDFVLLLCAAQQWRVFVAERTEEWLRAAGDNADRPDLEREPHNPTPN

FIHCRSYLDMAKVVVFRYLFWFVLVVVFITGATRISLFGLGYLLACFYLLLFGTAMLRKPARARLVLWDC

LILYNITVIISKNMLSLLSCVFVQQMQSNFCWVIQLFSLVCTVKGYYDPKEMGKDQDCSLPVEEAGIIWD

SICFFFLLLQRRVFLSYYFLHVMLDLQASALQASRGVALFRASLMKSMRSHRQAEKKSLAQLKRQMERIR

AKQEKYHQERLPGGEGQDGARAGESRWGGPQPLQPPSVTRAVASTRHQLLPQRPHPLLCKDRCSPAERTA

VEPPPHTPMPCTGVGAPMVAPLSPHCRSTAPSSPADPARQRERWWRPWLDHAAVLHSGEYFLFESDSEEE

EEAAVPEEPRPGRQSAFQLAYQAWMTNTRTALRQQEREQQEALGQEEEEEEEEGLVGGRQDPTSHGRGCP

EWVQSHEPLSPRPAERGNVLQRALNTLRFLWVLCQAMVDGLTQWLDACTQEHTDMSTVLRLERYVLTQRL

AKGEDVHHGVLDELYLLPPEEPQEEPQNGIASSGRNGAECEQRAEPPAAEYPLQRGSQEQVASWGSREDV

AGSRELLALPQNRSRTASELLVSRRLYFAELEESEKFYQSHNRFLKLLLAVYHCVAAHSELLCYFIIILN

NMVTASVISLFLPILVFLWAMLSIPRPSKRFWMTAIVFTEVMVVVKYLFQFGFFPWNGYAMLVRNEGKPF

FPPRILGLEKTDRYIKYDLIQLLALFFHRSLLLCYGLWDHEEDPFAKKKPEVEQAEEEEEEEERQEEAGS

PAVPAEPGALGAAVGSEPEGDARGQKEGSGDGATHLRFRRKRRRGKEPAAVVEEGEYLELGKMWSCREPG

GHPCCLSLTEDGEEEEEGEEEEEVEEEEAKQSHSQKKLKAFGLRVKLFFLTMAQNMYQPVRGFFHDILHT

QYRAATDVYAFMFLADVVDFIIIIFGFWAFGKHSAAADITSSLSDDQVPEAFLVMLLIQFTTMVIDRALY

LRKTVLGKLIFQVILVFSIHLWMFFILPAVTERLFSLNTVAQLWYFVKCIYFSLSAYQIRCGYPTRILGN

FLTKKYNHLNLFLFQGFRLVPFLVELRAVMDWVWTDTTLSLSNWMCVEDIYANIFIIKCSRETEKKYPQP

KGQKKKKIVKYGMGGLIILFLVAIIWFPLLFMSLVRSVVGVVNHPIDVTVTLKLGGYEPLFTMSAQQQSI

QPFTPQDYEALTNEFERQPVAPKPRSPLPLSPVGTPTGADPRAPFRPQVAMQFITLYGYEDIVTARIEGS

SGSLWSISPPSREQMRRELQNGSSDITLRLTWTFQRDLGKGGTVEHTFDKHTTDLQPGAPERMELAQLLQ

GTRDAPVQVPKLFPKYIRAPNGPEANPVKQLLPDGEDSYLDVEVQLKRERSGSGQGSDSFLEWWVVRLKD

APARDGNILPMVIFNDKVSPPSLGFLAGYGIMGLYVSIVLVIGKFVRGFFSEISHSIMFEELPCVDRILK

LCQDIFLVRETGELELEEELYAKLIFLYRSPETMIKWTREKE

>Gallus2 gallus XP_419138.4 P2

MSRKEKENPQEERCGRTPGSTEHRACAFRYNGLSFVYLIYLLLIPLFSEPTKTTMQGHTGRLLKSLCFTS

LSFLLLHIIFQITINSLEAGRTIEPGFNCSTWEKTLRQIGFESVKGADAGNVIRLFVPDIGMFIASLTIW

LLCRNMVQKAINEDAVHCNAQFENEEMAVREKLEPDDALMYEEDLDDDCGEAEFEETMKLKLLRRIASLA

SKLREFIGNMIITTGKVVVTILLGSAGMMVPSLTSAVYFFVFLGLCTWWSCCQAFDPLIFSCLCVLMAIF

SAGHLIGLYLYQLQFFQEVVPPKDFYARLFGVTSLSQTNCSSTWMIIKNQELHWYHHANPVLLLVMYYTL

ATLIRLWLQEPIQVPEEEKSESREDDKEIPCSPIPMTAERRRSLWYASHYTTDERKLLSMTQDEYKPSDV

LLVTVNGNPADYHTIHPSLPLENGPAKADMYSTPQYKWEPSDNMSEKEEEEVEEEEEVPQEEKENVKLHA

LVSVFQFIMKQSYICALIAMMAWSITYHSWLTFVLLIWSCTLWMIRDRRKYAMISSPFMVFYGNLLLILQ

YIWSIELKNDELPQVSGFLERKEPGELASKILFTITFWLLLRQHLTEQKALQLKEATLSEVKVGSEEDEE

KEEGELQEGEVAEEEEEEEEEQEDDDEDEQDIMKVLGKLVMAMFIKYWIYVCGGMFFFVSFEGRIVMYKI

IYMVLFLFCVALYQVHYEWWRKILKYFWMSVVVYTMLVLIFIYTYQFESFPGLWKNMTGLDENKLADLGL

KRFSVAELFTRIFIPTSFLLACILHLHYFHDRFLQLTDLKAVTSKQDNTIYRLVHQDGSLPDITMMNLTA

SSEKEEDKMLKEAGEKRMEEPDGEGGKGRAEKGKEEEEEDEEKEDDDVDDESEEEETTDLRNKWHLVIDR

LTVLFLKFLEYFHKMQVFVWWLLELHIIKIVSSYIIWVTVKEVSLFNFVFLIAWALALPYAQFRPLASSI

CTVWTCVIIVCKMLYQLTSIDPSTFSSNCTLPRENETKVDLEELKTSVLYSGPVDPAEWVGLRKSYPLLL

YLRNNLLMLAILAFEVTIYRHQEYYRCRNNLTAPVTKTIFHDITRAHLDDGLVNCVKYFINYFFYKFGLE

TCFLLSVNVIGQRMDFYAMIHAFWLIAVLYRRRRKAIAEIWPKYCCFLACIITFQYFLCIGIPPAPCKDY

PWRSGNANFNSNIIKWLYFPDFIVRPNPVFLVYDFMLLLCASLQRQTFEDENKAAVRIMAGDNVEICMNL

EAASFSQHNPVPDFIHCRSYLDMYKVIIFSYLFWFVLTIIFITGTTRISIFCMGYLVACFYFLLFGGDLL

LKPIRSILRYWDWLIAYNVFVITMKNILSIGACGYIESLIQNSCWLIQAFSLACTVKGYRIPTNNADCKL

PSGEAGIIWDSICFAFLLLQRRVFMSYYFLHVVADIKASQILASRGAELFQATIVKAVKARIEEEKKSMD

QLKRQMDRIKARQQKYKKGKERMLSMTQDSTEGPEIRKVSEEDDEGEADKEKAKGKKKLWWRPWVDHASM

VRSGNYYLFETDSEEEEEEEKKEEEEPRKKSAFQFVYQAWITDPKTALRQRRKEKKSFQKEEKRRRKVYG

DGSTDADCEDSEEEPVKKKSDGPDNIIKRIFNILKFTWVLFLATLDSFTAWLNSISREHIDISTVLRIER

CMLTREIKKGNVPTRESIHMYYQNHMMKLSKESGLDSIDKNPGQASGLQTSERMDSLDSAASRDSISSCY

TEATMLFSRQSTLDDLDGPDTVPKTSERARPRLRKMQSMDMSSSSADSGSIVSSEPTQVTMLYSRQGTTE

TIEEVEGEHEEEGAHSASRQDEEEVEDYSLGSEGAAYTPDTDVPLYSTVDSNAEAPPSYSKAVSFEHLPF

GSPDDSAGKSLMMVSPDDSRTDRLNDAILPPLTHELTASELLLNKMFHDDELEESEKFYVGQPRVLLLIY

ALYNTLVARSEMVCYFVIILNHMISASMITLVLPILIFLWAMLSVPRPSKRFWMTAIVYTEVAIVIKYFF

QFGFFPWNKYVDYTKDKPYHPPNIIGIEKKEGYVHYDLVQLLALFFHRSILKCHGLWDEDEKGDNSSNKG

DTDDELSLPGGRRDSSGSLKSVNLAASVESIHVHFPEQQTAIRRKSSSSASQLSHRSSFSSHRSKRGSTS

TRNSSQKGSSVLSIKQKSRKELLMEKFREQMIKAKAFTIKKTLQVYVPIRQFFYNLIHPDYSAVTDVYVL

MFLADTVDFIIIVFGFWAFGKHSAAADITSSLSEDQVPEAFLVMVLIQFGTMVVDRALYLKKTVMGKVIF

QVILVFGIHFWMFFILPGVTERKFSQNTVAQLWYFVKCVYFGLSAYQIRCGYPTRVLGNFLTKSYNYVNL

FLFQGFRLVPFLTELRAVMDWVWTDTTLSLSSWICVEDIYAHIFILKCWRESEKRYPQPRGQKKKKVVKY

GMGGMIIVLLICIVWFPLLFMSLIKSVAGITNKPLDVSITITLGGYQPIFTMSAQQSQLKDLNQTGFSAF

LGSYRGNTAALQFLEGYGKEDITLADLEGNSNSLWTISPPSREKMIQGLLDFSAEFTVVLSWSIQRNLTL

GAKAEIASDKLTFGLPEKTRRDIATMMSGKPLEKVTLETVYPYYIKAPSDSLAKPIKQLLTDCRWENITV

SLVKNVSEEGVREWWVLNQLGKRYKTNEESLELFIFSDKVSPPSLGFLAGYGIMGLYASVVLVIGKFVRE

FFSGISHSIMFEELPNVDRILKLCTDIFLVRETGELELEEDLYAKLIFLYRSPETMIKWTREKTN

>Danio1 rerio XP_696355.4 P1

MELQVVCGLLYCCLLPIFLLAACIFRYNALSLVYLLYLLLLPWFQWPNKHTLRGHTGCYIKALFSTSLIF

ILGHVTFQICLYTIPSLDDALGHNCSSWETLSRHVGVSRLPLEDPQGVLWLLTPDLGVFIMSLITLILCS

RLLKKRDEGSVPHMSALLHEADETEDDDEDVEGEDEGMLSCSETEDEESPSSSSAAQLAARLRATAQRFL

RNMGRILAVTLLALAGITLPSAFSAFYFLLFIGVCTWWACHFPISHLGFNALCVMVAFFTGGHLVCLYLY

QSSFAQAMFSPAGLWARLFGLKDLVTPGNCTTFEVTLNTQYDWPVYVNPGILLLLYISVTIVLKINSNSL

GDADKAREGEAVKTVQGEAGEEVELRLWEARRQSSEDDTKQTLLTTVDSTLSESHITKEPVINGSSQHDI

GPSGEATVDNPLRLVGRMVLQQSYICALIAMMVWSITYHSWLTFVLLLWSCVIWMLRTRQRFAAYCSPFI

LLYGLALCCLQYVWAMDLETELPQHIGSMSLHQLGLDRAQYPCLRLGALLLFTLTFWLLLRQSVKDTFSR

KKSLTVPLQEVTTGESTGRNESILKVFGGLVMSLYAKYWIYVCGGMFIMVSFAGKLVAYKIVYMLLFLLC

MCLYQVYYSLWRRLLKAFWWMVVAYTMVVLIAIYTFQFEDFPGYWGNFTGFTEQQLADMGLETFKLSELF

TSIVIPGFFLLACILQLHYFHKPFMKITDLENVSPIHRKRGIDNPDLVQSTEEVRPEEEELIVDQDDYPS

EADEVIPSKWGLVMDRLMVLSRKFSDTLTHVQSFIWRVLELHIVKIVAFFVVWVALLEPSAMNLVLVVLW

SFAMPYGRFRAMASCLSTIWVCVIIVCKMLYQLSVVNPAEYSNNCTAPMVNETSLEPDEVLNSTLYRASV

DPASWFGVRKDDTVLGYVKDHLLVLMLLVFEATVYRHQIHHYRQQQRSPPPIPVIFPQATRDTLDQGLLH

CIKYMLNYSFYKFGLEICFLMTVNVIGQRMNFLVIIHGCWLVAIMVRRRRANIATIWSKYCLFLVVFMIY

QYVLCVGIPPALCIDYPWRWRSTVTIHSALVKWMYLPDFYTVPNSKNLISDFLLLMCASQQWTVFDNEKK

EEWMVMGGENRDNPDPMENMLFNPAPNFINCRSYLDMAKILVFRYMFWFVLSVVFVTGATRISVFGLGYL

MACFFFLLFGTKLLTKPSRTRLVMWDCLIIYNVAVIISKNILSILACVFVMEMQKNFCWVIQLFSLVCTV

KGYYDPESVCNKDCSLPVEEAGIIWDSICFFFLLLQRRVFLSFYFLHVSADLQASARQASRGFELFRASI

IKNMHFHQQAERKSIQQLKESMKRIRGKQQKYKDGRITSDIEEQPSDPKEQKKETKLSKSKSWQYPWVDH

ATVLHSGEYYMFESDSGEEDETFQEEQKPRRQTAFQLAYQAWVTSAKDALKERQQRQRKQRAEAEKQQNA

QATDDAEEGETVFDGENSQHAEEMQEEGEDAASQATGSDMVQRILDILKFLWVLFLAMVDGFTMWLNLLT

KQYVDTSMVLSEERYLFIHNVSQRASRENMDDQISHDSEDLTLETCLDETDTDNICDTNVVESETLGGFA

KSRDFSSTTLSSELTLELDCEPFYSQQTHRPHRSRTASELLSERPFRVDELIQSREFYSSQNRLLKLLFA

LYNVLAAHSELVCYFIIVLNNLVTASVISLVLPVLVFLWAMLSVPRPSKRFWMTAIIYTEVMVVVKYLFQ

FGFFPWNTEYELKLNEDKPFFPPRILGLEKTDNYIRYDLLQLLVLFFHRSLLQRYGLWDQEGPLEEKSDL

SPAENSKNENDEKKFQHSDEEDSQAQPEPNTEVLSLTPSAETPTDAEIQTDPEPNSHTNNTKTSDTVKND

GMKKSHFLRFRKKKKAEPDAVQKEPKKKKKKCKTKHSKETSKKLLNAIEGKFKSLFLSVVKNVYRPTWDF

FQNILHAEYRASTDVYALMFLTDVVDFIIIIFGFWAFGKHSAAADIASSLSEDQVPEAFLVMLLIQFSTM

IIDRALYLRKSILGKLIFQVILVFGIHLWMFFILPAVTERMFNHNSVAQLWYFFKCIYFTLSAYQIRCGY

PTRILGNFLTKKFNHLNLFLFQGFRLVPFLVELRAVMDWVWTDTTLSLSNWMCVEDIYANIFIIKCSRET

EKKYPQPKGQKKKRIVKYGMGGLIILFLICIIWFPLLFISLVKSVVGVVNHPVDVTVTVKLGGYEPLFTM

SVQQQSIQPFTESRYNQLNNQFSKNAVAMQFITMYSYEDIVTANIEGSSGSVWRISPPSRQELIKELLSS

TGDMTLRLDWNFQRDLGKGGTVENTFDKHSISLAPKNPVRADLASLLLGTRKDPVHVPHMFPNYIRAPSG

AEAKPVMQLFDGDEEGFQDVTVSLMREASVNTTGAQEWWDISIADCKESCNVLPMIIFNDKVSPPSLGFL

AGYGIMGLYVSVVLVIGKFVRGFFSEISHSIMFEELPCADRILKLCMDIFLVRETGELELEEELYSKLIF

LYRSPETMIKWTREKNHN

> Danio2 rerio XP_009295489.1 P2

MMEVLGSSLMAMLNKYWIYICGGMFFFVSFEGRIVMYKIIYMMMFLFCVALYQLHYERWRWMLKYFWMSV

VVYTMLVLILVYTFQFESSINVWSNMTGMSREKLEDLGLEKFSVPALFTRIFIPTWFLLVCILHLHYFHE

RFLELTDIKTVADKQKSTISRLVHLDGSLVDISMIKPTFNSTNEEEVEKQEDEEDNNYHNEDEDEDERVE

LEEEDVKQTEEQKMEDSLFTCPMTADPVLEQTSGTVSLFNYVFVVSWAFALPFCQFRPLASSVCTVWTCI

IIVCKMLYQLTSISPAAYARNCSMPDNYTEAQRMDMAKSLLYIGPVDPANWIGLRKFSPLLEYLRNNLLM

LALLAFEVTIYRHQDFYRMKNNITPPVTRTIFHNITRQHLDDGIVNCAKYFINYFFYKFGLETCFLLAVN

VIGQRMDFFAMLHAFGLIAVMYQRRRKAIAHVWSKYCCFLACLLSFQYLMCIGIPPAACTDYPWRRSSSS

MDSNVIKWLYFPDFHTKPNSLFLLYDFMLLLCASLQGRVFEEECVSPVQLLAGDNVEICRDLDAATFSQH

NPVPDFIHCRSYLDMLKVMVFSYLFWFVLTIIFITGTTRISIFCMGYLVACFYFLIFGGELLLKPIKSIL

HYWDFLIAYNVFVITMKNILSIAACGYIKALVANHCWLIQLFSLACTIKGYSKPEQQANKQCELPSDEAG

IIWDSICFAFLLLQRRVFMSYYFLHVVADIRSSQLLASRGAELFQATIVKAVKARIEEEKKSMDLLKRQM

DRIKSRQQKFSEGKERMLSLAQESGERHSVLKEEEEEEEEESSKEKKKGKRKQWWRPWVDHASMVRSGNY

YLFETDSEEEEEDEDRKDEEPKRSAFQFVYHAWITDSKTAMKERRKEKRRFWQKYTESNTNKREQKEANV

TVEEGDQDGVTEDELSDSPDNMMKRVFNMVKFSWVLFLALLDSFTAWLNSICQEHIDISSVLRIERCMLT

HEAKKGNVPSRDSIRMYYQRQMCRPASTQSSLEPTEEAYSDFSPLRTDPDLPPQEHTELQTSTENLASVY

TESSIVEDSTLDEERVLKTSQRSRPKLCRISGVDVPSSADSAALKATFCSDVGEHTQGTELYSRQGTSDT

VEECGADTLWSSASDLLQTPHRDVPPSYSRATGLDCICDERKLKIQTPEDSSSGTPHTSSHRLTASELLQ

TRMFYDADLEQSERFYRSQPQLLQLCYSLYNVLVSHSQMVCYLVIILNHMISASVATLVLPVLIFLWAML

SVPRPSKRFWMTAIVYTEVTIVIKYFFQFGFFPFNQSIEVDKSKPFHPPNIIGVEKKEGYVHYDLVQLLA

LFFHRSILKCYGLWDEEEPRSVSPHTHSGAEEKAECFSEKSPVEDTYTHALSHTHLRLARKQQSSAGVRR

QSSSISQRSARSKTGSSVSRSSVRRSLSAQQKSRKELLMEKLREQLIRAKRFTVKTVLDVYIPIRQFFYS

LIHPEYSAVTDVYVLMFLADTVDFIIIVFGFWAFGKHSAAADITSSLSEDQVPEAFLVMVLIQFGTMVVD

RALYLRKTVMGKVIFQVILVFGIHFWMFFILPGVTERRFSQNTVAQLWYFVKCIYFGLSAYQIRCGYPTR

VLGNFLTKSYNYLNLFLFQGFRLVPFLTELRAVMDWVWTDTTLSLSSWICVEDIYAHIFVLKCWRESEKR

YPQPRGQKKKKVVKYGMGGMIVMLLICIVWFPLLFMSLVKSVAGVVNKPLDVSVTITLGGFQPIFTMSAQ

QNQLKELTESDFKNFINSYNYVPSAMQFLEAYRAEDVTVAELEGSSNSLWTISPPSRKNLMDVLSKEDQF

PVTMTWSIQRNLSLGAKSEFAMDKHVTYLDIKTRQELIALLNGTRNTPVLIEQVFPCFIRAPSDSNAKPI

EQLYEDGVYKSIQLDLERSANSSEDLQEWWIVDELRSAQKQQREAGLQLYVFSDKVSPPSLGFLAGYGIM

GLYASVVLVIGKFVREFFSGISHSIMFEELPCVDRILKLCTDIFLVRETGELELEEDLYAKLIFLYRSPE

TMIKWTREKNK

>Ciona1 intestinalis XP_009859039.1 P1

MDKFLHYCQQPYFLYTSVLNQQFLSITDVYVWIFLCDVINFIIIVFGYWAFGAYSSQQTNVVSDLQSNQV

PTGFLVMLLIQFSMMIIDRVIYLRKSLRGKLIFQIFLVAGVHIWLFFILPAINNRPFYQNGVAQTWYFFK

ALYFLLSSYQIRCGYPNRVTTNFLTNSHGTVNLFLFKGYRLIPFLAEATTVMNWTFTKTSMSISEWLKLW

SIHAEVFELKCWRSFEEEYPSQRGSAKAKWVQYLIGGSLVFLIFFIIWFPLLLMNIALTIGGVTNNPTQV

DVKITLGSFEPLFQVIADSDDIKIWTESQFDNFQQDFSVSQAAAAFLSGFQFDQVVTVTVDGESKTIWTI

SPPQYELLVDRLSPNNTKPVRLLFEWTLTNDPSVTNVVSSYGSNSVMLNRSSEARQHLYQLLKQRNETNT

TDSAFIMNLFPQYIHSQGTSSTARPAYNLYQGTHQSYTDVRLTLRRNASSTWWRVFDDSTSLEKPMFEIN

IFSDKVSPASLQFIVSYGLIGLYVSVVLVVGRLVRAGLVGATSTIMFLELPYVDRILQLCNDIYLVQEEK

EFELEEDLYAKLIFLYRSPTTLIKWSRPKVD

> Ciona2 intestinalis XP_009859155.1 P2

MCPVIVSAAKTVASLFIRGLEFHVWRLVFITVFCAAAYKVSAIYGVYVIFWICLLPARITHRALYVVTII

SVSIIILGNMFFQLQFISPELKYNCTFNSYGNMKVIEYEMLNWIGIFKMNQNGAYFNHVNGFMAIILMIL

FERVIMLRQQWHLKWRNIVQPTMTAFYPTVTWQELDLGIVEFLKYFVNFAFYKFGVEACFVMSVVTVWVR

ADMYAVVYALLLIIALSLKTRLSLHRYWKFYSIILLFLLILQYLICIGMPPVWCVSHNYPWLVFGSNETE

RDRLVRWMFLPDYLVLKNSTILIADFFQFLFIVSQLYVFEWEKSGWERYGGDNSYLTGAELDHVTIGTNP

RKNFMLKKARSLLDQLKSFVFKYMIWVSMAMVFIAGTTRISLFCLGYIVAFFYLLSKQQELLLGKPSRLL

KVWNVVLGYNVAVITLKISLQIASCVYIETLQENACAVVQLFSLVCSKSGAYGLQVAATSTCELSYTNSG

LTWDFICFLFLLIQRRIFSSYYFLHVTEDLKISLKLAARGAELFNDKLIEDVKERESVVQESLSALKSSM

KSIKNRYRLLRKGLPPPTTHTEATRSGDYYMFDEEEHERKEIKETKDEIMDPIKMLHKLGTVGIDETLVE

RKEDKKKLTEDSEVTHHAHEDDTHNEATHDETTHEDDHDSDDHESDHDACEDEVDGKKQKTFFESLKQCW

KLVCEFVLSGVDYLTLKLNKGNLDYRNVTLRLESEKDEISRKKFKEERSPRGSTEVAVDVNNDHNDAIDV

DGITIESNACTEETEWKDLAAIEFDKDQHWILRFFYSFWFVIISRTDVVCYFLFILNLMLDSSILSLVLP

LAVFFWASFCIPRPTTKFWNFSIVYLQLVIVVRFIFQFEFFPWNSQEEMNLHQNEPFWWPRFIGIEQSSS

WMVMDLLQLLAVVFHVSVLKVRQYIK

> Drosophila melanogaster NP_001036346.3

MVFSYACMVLQRIVVPAVLVLAALMRPVGISFVYLLMFFVSPFVPLATRRNFKGSVTAFFIILLTLSTLV

LLGHITLQILAVSLTLPIYNCSFSERLLRHIGFVSFIDLQPFAIIEWLVPEVLVFATSLGSYLTVKRVAS

QPVGAEQLENGEVVDGQAENAQTSSQPSAADANGGDVQQATVTTPLQQQQQQLRKRVSMISQHIHFEGLV

KISPLFCLATLFFAAVLRPSVPGGFYFLIFLLSGTYWATCQTLQRGFALLLRCVMVVLVLHSLSIVSYQT

PWMQSHLNHTTLTARLIGLEPLIESYCSPDIRVFLYNNKLSLDSYLNPFALFFAYFALALTTKHLIKPRL

EPKPATALGQQLDCNSSSINNTTGNKVNRQLSLLTSQTSRGRRDGSNPGGGGTTITTTTTTTSTTAIRNQ

RLSLVRQSTRKARTPQPLESGSSVAPSVTQRGNDMQLESMEQRSEQENTTTSILDQISYGFVSVGGFIYQ

NSYIFTNILMMAWSIVYHSWLTFVLLLSANVLWMIPNQRKAMMRSSPFIVLYAEALLIAQYIYGMDLNNE

ELPTSVPTAGINLQQIGFERPIENQMRPCVPLIVKTAFVLMFWVTSRQFFKEKRDRRRDSTLADFIAPLQ

ITVGSAGSSYLINDGKKTSKFLKKAGDVIKNLLVRLWIWLLVLVIFLCAITGENMTGFRICYMALFLFFL

LVFQSSSKAWVKIMYGFWLFLIFYAMSILILIYTYQFDKFDTYWSDYLNVSATLQKDIGLKRYQTKDLFL

HLVSPTIIVILTVIQVHYFHKRFIASLQQQPLAGGSAQQKPTETTALEPAPSKRRGSAGSLRKSQGPSAE

AAPGATTDFETSVRDLVRISFRKIKNKSEYIFKNFKDVFWRFLELHIMKAVYIAAFVCSVSEVCVLHIIF

VGFCVLGATSRKAVQVVISRLISFIVTVIVLSKMIYQIEYLSHSQHNVVCSDNRTANNAEWIGLTKADKV

TGGLMSLLRTYIIYMVIVTMHAVISLRQLQMRVKIGALNAPPTKLLFPNIIRADAEKDLVGLVKYLLNFG

FYKFGIEISLIALVSTITYRQDIVAVVYALWLVVLLLLRRSQCAKIWGVFQAFFAISILTQYIVLVGLPP

SSCLVFPWDEGPFGEGIQRWAMLPGALHFNHVPKLIFDFIVLVILNRQKSIFCIEQRYASNDDYPGGSNR

SVIADIAQLGRVPFDNPTHDFCSYIRNYSDILKNGVLCGFYWFTLAVVFLAGTNIADLLALGYLIGAFIF

LWQGSDFYLRPIHTIIFRWKWLLAFNVANILIKTSFQMAGCLFMTQLTKDCCWLVHMLGITCTSNVLTEQ

IMLPEEAELALKPGECPKITHQVVLLWDTICFAFIIFQLRIFKSHYFCHIITDTKANNILASRGADIIES

LRHKQIAHRHDHEKQVLHKIKRKMERIRATQQKMLRPLDKQTHFDEHGYPLPAPTVRRRKEIKLHPHATR

AGDYYMFEEMDDKFELDLIHDEIDFLEEENITESEMKMQRRKTLYDVWKDLNDAEYRRHFYMRERSYASE

PGSRILLKLDDEKEKIGPDLGLASDHDEDDDTIECDLGMRTSTLSEPLDFGRRSQTLLEVKSKDAPTGEF

PSTSKGISKERDAATASSSASPAPTRDVGDLPVIPPPSTGLGREQTSKETSDSKSKMEVDSGEVTAKDSD

EDFDTNPIIRLLEGFLVTLTIRLNRFSRNYRFVNRILAGEKKTLKESSSLNRLGLSSAAAMFHFLKSNLE

RKQSINSSPPAKGTMLSRKSDCGLPEIRIKAPSIERGAHYYHNHHSGGGSGSLSKHWSYEQVDSAGEFNL

EEENFAQRDHHIIVEVLISSWYALLANTDLICYIVVFINQVVNASLISLPLPIMVFLWGTLSLPRPTKTF

WVTLIAYTQAIVLIKCIFQFKLIWSNYHQLPNQPLTPAKIFGVENKAHYAIYDLILLLVLFLHRYLLKSQ

GLWKSGYKDTDNQFTKPTASIDERDDSDNLSQPDSRQLNDDAAQKLSLQVSQASLPGSPEFSKTGINQLE

RTKYTSSLYKFFFSLVHKSRLATDVYALMFLCDFVNFFVLLFGFTAFGTQQTESDEGVQTYLAENKVPIP

FLIMLLVQFLLIVIDRALYLRKALVNKIIFHFFSVIGIHIWMFFVVPAVTERTFNSLAPPIIFYVIKCFY

MLLSSYQIKSGYPKRILGNFFTKGFSMVNMIAFKVYMQIPFLYELRTILDWVCIDSTMTIFDWLKMEDIF

SNIYLIRCTRQSETDFPAMRAQKKASLSKLIMGGTIVLLIVICIWGPLCLFALGNAVGTSNVPFHVSLSI

RIGPYDPIYTTNNYDSIFEINPEMYSQMTNAYIKEKQALTFIAGYDATDVAAVRLAGNSPSLWNIAPPDR

QRLLNDLRNNHTLKARFSYSLTRKAPAKGLKENVGDEHAISLDESFEGRAALIHMLSETHDVEPIYSNGT

TNGTTPEVEEVVVIPGMIPKFIKVLNSGDAAVVSVLSPKHYDYRPLVIKMHRDNETNGLWWEIRDYCNDT

FYNETLSKFAYSNCTSGIVMYTFNDKKFPSTFSFLTAGGIIGLYTTFVLLASRFMKSFIGGQNRKIMFED

LPYVDRVLQLCLDIYLVREALEFALEEDLFAKLLFLYRSPETLIKWTRPKEEYVDDDGDTDSIPSRMSVR

RPEQLQPQQPQ

> Caenorhabditis elegans C10C5.1, isoform a NP_001255389.2

MTVPPLLKSCVVKLLLPAALLAAAIIRPSFLSIGYVLLALVSAVLPPIRKSLALPKLVGTFVIITFLFCL

AVALGVGSYQISEQVVHKNDRTYICNRSDTTLFRSIGLVRFHPTGTFESTRAFLPEIIATSAALLTIIIV

MFLSHRDEQLDVVGDVVTVRSESGREQRRQRKLAAIMWSAIGNSLRRLTNFVLFLFTAYVGIVKPSLSNS

IYFLAFLFISTWWSTYTPLRHGVYNQIKKFLIFYSALHFLVLYTYQIPIVHHSWLPTGSFLPRLFGLTVL

MDSSCPEWWKFPFVAPDFNDDDLIMKWPLYANPIVVLVFFYLTVAQYKFTRNGSREYIDDNEYGSSVHEE

RFVSAGTVETNVDDVGQLISISESTASAPSGRGRGNTLLLSNASSSANDDEQGRARSRSPLRNGEEQGSI

PLRKVTSQVVDRNKLSNIFNTPGDKESAASKGMIAVMTFVIFHSYSIALTAMMTWALLYHSIFGLILLIL

TCILWIFRDTRKSSFAMAPIILMYIEFLLILQYFLSMDIHAEIGDPAWMNFVGIEWTTLPVHAVIILCVQ

TLLTLPVFLLLRLARREKFYESLSDYERQRRINSYGTFGASKTGAGGVAVAKDPKSRKFAAFVEYLSNKV

SVYFIFVVSVVLLVVSTCFAPNFYNILFFALWALNLIYLKFSFRLYRGLAYAFWLTLTFYTSIVIIALYI

YQFPGVSQWIIRNTSLSQEWLNAIGLVDFRAIGESGALFLQLLAPIALFVVTMLQLKFFHGPWSRATSPR

RAENDPPTSTTEAAAVASTSGTQGRAHAAGDTLVKKLHKLANQTIELLWRFFEVHISKIVFVIIAIFIAN

NINALYIPLVILLSLAICLPSAADGIFSLFMCAYLFLVALSKMIYQLDIVPELSQIDRGVGADNCSHGNI

SMPEWFGLKKEVEGTEPIYMLFGVIVSIIALAFQSIVIYRQRHYRASLGLPESMRAKVFPDFHHSHFDRS

LKNAIQFLIDYGFYKFGLEITMIAIGIDIFNRMDALAAIQCFWLVLFALNKRVFVRRIWVFYVIYMAILY

PLQFFSYVGLPPDSCIEYPWSYWIPSYSDDARFNLSYLLNLSIYGVNWPSAYLIGDFFVLLLASCQLAVF

RREGEDNDSIYNDGNFVIKPENPQYDFIDTKKSYVDYFKSFVFHYGHWITLMSTLAAGIAGTSLFALGYI

IFTLTMLWSGNNLYVMNSTLRSFEHTLKRWNALLGYTLFTITMKVCLQIFGCVFLSWFDQSGGWGKTLCI

VRQLFSITCVNNECHVLKELEDFSKACAVETKEGNIGFDVIALSFLVFQIRIFHSWYFQHCMVEYRSEVI

LANRGAVLKNQLIEKEMKEQNEQQKAKFNDIRRRTEAIRERYQKQIERGAAERDFEPVTYGHAKRAGDYY

MFKYDPENDDLVEPVDSFVPEVDPKATAYDRLDPGQIMYAATAHDLDLAKTVQQVKKGDTIKDPDSRALI

AVSEPEARKPGGTEETDGDEDEDNKDSKVESTAKFIQKMIASALDLCSVTLNKLCREHRYVGFVLSKEKQ

KLKSGHSESLSNTSRKLTDIRSAVDLPSLQLVQSANDVEKMETAVSVDWQQKSSATRLLNAVVNCIGAHT

DILCYFFAIMTQVMTGGLITLPLPLMSLFWGNLSNPRPSKFFWVTMITYTECVIVIKFVCQFAFMPYNSI

TWRTEHQMDPMSLDKLFGVSQRDSFALWDIVLLFSLFFHRYMLRKLGLWKDANLTDTFTLKEEPRSASGS

DTGSPKKIAQEPKVVVTQSDTLEGTSGGEIVIPSDPNAVSNMEELDCEPPIPEKQSGPIGRFIHQLFHPK

FRYIRDLYPIMFGIDVICFLIMTFGYSAFGEGGSGNVLDDVKASRIPVTLVVMLVGMTLAIIIDRALYLR

KSVVGKLIYQVLMIAFLHIWVFLVLPNMTRRSAISNHVAQALYVIKSCYFLVSAWQIRNGYPELCIGNLL

THSYGMTNMIAFKVFMNIPFLFELRTAIDWTWTDTSMPLFDFFNMENFYAHIFNIKCARQFEAAYPAPRG

IPKGKLVKYMMGFPIIIGVVIFIFSPLLLWSLLNQIGTISMPEKVTLRISIEGYPPLYEMEAQGSNHDNA

ELGMIKPDQLASLNQALTDSYTTRDTNSILRSRMSVSYLKGYTYEDILIVRFRPESEIYWPISQDSRNAM

IDKLSRNTSVNFEVSLEFTRPYDPNENAALKHSKSWLVPISLDMTIRAKIQSALRGDPGHPILIPQSIPA

FIQVPNQGELTLPTSIGNTIINDGNPRINTTGMEKSDEARAWFDSLTLNLEQGKSQNEKMWIATSEHPGD

QNAKLWIKTANTTYSGRPYLQVVGFIDRAFPSFLAKVFKGGVIAVYLSVILVVGRGLVRGIFTTSPSTVM

FTELPNADHLLKICLDIYLVREAKDFMLEQDLFAKLIFLFRSPATLIEWTRMSKKKQE

>Tribolium castaneum PREDICTED: piezo-type mechanosensitive ion channel component 2 isoform X1 XP_008193839.1

MANFFLCLFIFRIVLPIVLGACIIFRPSLLSAIYLLFLLFLPCIPIPTASSMAGSTGIYIKLLIATSILT

FVAQLAFQIVLLAMPPYGHILEKCELLEQILRHVGLVRLDAMNPIAVVTWISPEIVMVVISVGTYVICKK

LLEKRTVEVVENEENLPQKAKANRKQFNLFVAVGKYAVLVALCVAAVLRPSVPGGLYFLVFLCAATWWSC

CKELGKGFAIVMRCVMAVVVVHMGALYTYQFQWPQEYLDKNSTYARYFGLTPIFWSNCTDDPRTFNWEQE

EWATYANPIALFLLYYVLALESKFLLKPQSQKKQGKFLRLEGSFTKPLNRQLSNKQLMRRATTRNKWQSA

TRKVRIPRTEYAISEHTPLIGGVSPSRRYGSGKKPPVYQDSTGSFTITGENPEDIPMDELGKGAAEEEYK

PTIIENVMYGLESILQVIIKSSYIVTNIIMMAWSITYISWITFVLLIWANLLWLVPNQRKSMLRSSPFLV

AYAWFLLISAYIYSMNLTESELPTTIEGIDLSEIGFQKVEVLPCNPLLVKCLFTAMFWITLRQFVRERIE

ERQTTALADMAAPLQVTVGAAAGVENEQDPGSKLMEKIGQYLRKILTKFWIWVVAITLFAVAITGERMTA

FRIFYMALFLFFILTFQISFRAWRKMMFGFWLTVIVFSMAILVMVYTYQFKNFDIYWRDYLHVPIQRQLD

IGLEKFETKQLFVRLVTPTFFVIITVIQLHYFHKEFMELSDPKNTSVIGVDLDQSSLQGGAPTSDKDETS

SSIKMDLTDTDSSQTRWQRLVKYFHHTSNLIFLFLELHMPKFVVLFLMLVCIYDKCALYFILVLLLVLAC

AFGRPMQIFAIYTSSVFVSVMLLARMIYQTDYINPGNWNVTCEMQNNSKIANNAKWLGFYKTSKDASLPH

LVKWNIMYILVVTLWAVILVRQFNYRVSRGKPTTRAFFMFPKITRWDADKSTSNCIKYLFNYGFFKFGVE

ICLMATVAVIGFRMDMYSIIYSVWLCVMFIAKRDTLAKIWTFYMMFIALLLPIQYVMTVGLPPTLCILFP

WDQDYWDKSEVKNAAIFRRLQEFSYLLDTEYPPSPKKLICDFILLLLVSRQAVIFRIEKRWAGREYPGGS

NDIIIHHAEEKGFVNPTPDYISSVRSYLDVIKKGVYSSFLWITLAIVFLAGTNRVNVFSIGYLIGAFVFL

WQGTDLYLRPIPTIIKSWDLFLGYNVFVILCKTALQIVGCIFIQDIPNYACWLIQLFGIGCVKKFGDLTV

QAGLADELVCKVPREYVGLVWDGLCFGFMIMQRRIFTSYNFFHLINEIKAGSILASRGAELIEELRLKRM

TEQEEQERRVLEKIKAKMDRIKANQQKIQGSSYKDSENHYVDTIFKGRPKRRTREPKSYKQAVRSGDYYM

FDDLDDEDELDFMDIPKDDEEEAKGRGQTVSELLSTAMKTDISTSVKRSETDFRRRGSMPLPTRQKSIIS

TRSQLSAPYSAPPIIEEPGPRTVTIIDERGKDEPKPGTSKDDDESTVISEQKPTVREKVSVGLLFVWAFI

QSSMISLTNFLNKYSRDYRYVIRVLAKEKKFLKEHTDYNVGLRLGSGQLWQPAASYNSLLGQSHREGSRI

DMEEYEGTEMSSYDQPPIIRLLLAIWYIIMSRSENVCYFIIFLNQIKSATFLSLPLPLMVFLWGTLTIPR

PDKTFWVTIIAYTEVIVLIKCMFQFDIIPWNMSQGITNNPFYPPRIIGIERNSNFAVWDLLLLLVVFFHR

FMLKSMGLWKSSPVPAVLLTEGDYKVNSEGKLEPVQSEMQITRRSSKHSDKTLSSKSSVPEDSDLERLVE

ESDQNEDDMRRASITKQDLQDKILSVECQRVDPMAHLPQSLKLAFLKYGESIRLFFKQLRDPTSRVAADV

YSYMFLCDFYNFFVILIGYSSFGTQQGDGGVSSYLEDDRVPVLFLLMLILQFTLIIVDRGIFLRKNILAK

IIFQFIQVFVLHIWLFIVFPIITERGFNSVLAPQMYYMVKCFYLLLSAYQIRCGYPTRILGNFLCKGYNY

VNMFLFKGFMAIPFLFELRTVMDWMWTDTSMTVFDWIKMEDIFSHIFQIKCTRHVESEYPQPRGERKPPV

IKYLMGGAILALIIAIIWFPLVFFSLGNAVGKPNPPYDVTLEIRIGPYEPVYQMSAQSNSIHQFTESNLA

QLQQAYIQQKTAATFISNYEGPDIAAVKLSLDSANIWSISPPDRERMVAEVSSNDSLKIRLEYKVSHKTS

KPEDSGVIPDNVEIQVPAAINGKPNVMRQNLLQMLKGNKSASPMILEDILPKFLKVTNRGTAKSISQLMF

LNNEDAESPNPVGKAFRTISLSLDSSNDSVTEWWQIKENCTDLNYKMFLKKLPMADCNSIIVYTFNDKIF

PSTLSVLTGGGIIGLYSTLVFVAFRFFRGFFAEQCFKIMFEDMPNIDRVLQLCLDIYLVREAGEFALEED

LFAKLVFLFRSPETMIKWTRPKEELADDEDPEGDA

>Ixodes scapularis conserved hypothetical protein, partial XP_002402212.1

MAWSITYHSWLTFILLLWSCVLWMVPRSVSRLACLRSSPALVAYAELLLLLQYLYSLALTDEELPQNLST

VNLAQVGLIKYHENSYQPLAVKILFTVMFWITLRQYIEHKTAPPEAGIELRRQMSTATSSLGMGTQLRVR

HLGRLISEWLTHYWIWVVAIMLMVISLGGTDVVLYRIAYMLLFLFFILISYQLWIKVMYGFWLTVIIYSM

LVLILIYTYQFEKFPEYWSTYLHIPQDIQKDIGLEVYQSDPGTLFLKLLTPTFFLIITIIQLHYFHKERA

VLSDLELSEQWAFPSSPAPSREEESSESRSRVSPLPGDHDVSIPVEDGPHPQDDSEDAATPATMVPSGCG

PGTTMADVLRSPKAGSPHISLERIGRFLSQAAEVGWRLLEIHTMKGVLLAVCAVHVVFVLLAVVALPFRW

LQLFLVHCCSVWASVLLLSKMIYQLNFVDKYGWSTNCTVSLFQDVIGIPSNMTGFPAPFNTTIDNRIWIG

FEKTSNLTSYCKGYIMLIVVFSAHAMVRYRQSFSRARHQLPEPRPGVVFASVNRFNADDGIRQCLMYLLN

FFFYKFGVEVCFVTMVSCIGVRLDVFALLTSLWLCAMFLLRRHSLAKVWPFYVAYLCLVLPLQYLVVVGL

PPGLCIEYPWWDPSNKTLRETALWLYLPDFQEPPLARKILVDFVQLLFACCQLYVFFLEGSQNSDFYEGG

SNKEIYDKDGLFIGQTPNPIADFVTYTKSYLSMVKVFLFFSFYWITLAVMFLAGTNRVSLFAMGYVLDFS

EETCVKDSENSPRITHGILFCRWNTLLAYNVTVIFIKCILQASTPVVGCVFLDELAKKSCWVVQLLGIAC

LKKLQPDGTAGGDGDNMCRVPVDEAGLLWDGICLAFLLVQKRLFTSYYFHHLIIEILAQQQLASRGAEMI

HEIQMKDVQEQRAAEKDIMEKIKRKMDKIRANQQKVRGGEYVEPETHFQGEPDWAPHRFHFSTLGAGHRG

RKGHAQLQGLHQVLRQGGEYSTPYSTPAGGQSLLEEEPPPGGVESPSVGQLTVSPVSAGYTAYLDSYMEP

RLSTAGQQQHVRLSSVPRPTAAHAPARNAGGQRSSHYSALQRLAALRQAGSPPCLVVFPQPSKSSSKQPA

TSSDLPSMSAEEDKTGVPEELHTEHIDPPTEQPAEELQQQLEEQPPAEDTLLEKVVNLLAFSWALLDSML

ISATAKLNSVSRDYRYVARKLADEKRIVKAQFVTGLATTLNEPALRNRKFHFHKHSEEVAPSHPSLVRFL

IACYYAIVSRSEVVCYLVIVLNQMKSASLLSLPLPLMAFLWGSLSVPRPTKAFWITIITYTEAIVVIKYL

FQFDFFDWNESGPVNRPFDPPRILGIEKRKEDSDYALYDLLLLLLVFFHRFMLKSLGLWKDTDRGASVSV

VQSPPEPALCGEVRADRQSSDHASPDPATSGFQDEASEVSTELLQYGDQLVAFRFGVLTWLGGDCLDERF

FKAVCESEFWLHSLRRKVFLEVSVSSIKIPSIARFLQGCSFCSVSRYVEPFHNFFENLLHPVYRVTTDVY

AYMFFCDFINFFIMVFGYWAFGVVWKLGGYAALVSPSQVPVPFLIMLLAQFALIVIDRALYLRKYILGKL

IFQVFIVFVIHIWMFFVLPGISQRSFVEEKNLPPKLWYFIKCIYLILSAYQIRSGYPTRILGNFFCKKYN

YINYFLFKGYMLIPFLYELRSLMDWIWTDTSMNLSNWLKMEDIFANVFLLKCQRRAEEEYPTPRGSRRSS

LTKYGLGGILLFAIILVIWFPLLLFSLGNTVGQSLLPMDCTFELSLGGYEPIFKISAQQGSLQPLPYDSW

VRLQVRSLTPLPLLPAFLATYDASDVAVVRLNGNSTAIWTVSPPSQEALIKELGGAMVPLRLRWTFARSV

DNTNADKVVGNEHTVQLVPNTTSSLAEMLNGTSKNVSVPSILPRFLLVPRKGKVEVVRALDLPDREPYRN

LTLRLRTGAFKNLSARSEWWEVQESCTDAYPYPFLRDDQGSCSNLSMVVFNEKVFPQALSQLTGYGIVGL

YTTFVLVVSRLIRGFMAGSSFIIMFDDIPNVDR

> Arabidopsis thaliana uncharacterized protein NP_182327.6

MASFLVGFLLPSLLLAAALINWSVISFLDLIAFLLVHYIAPEIGYRFQRRHWLLWPIFIFSFAVFLAQVV

YLVIWAALGQDWDTPDTGWMRVIGFMILKSWRNPTVMYFLALQLLTSLVALADIYSSRFGFARWRDTWWS

HFSGIFEHLGSHLRVASCLLLPAVQLAVGICNPSWVSLPFFIGSCAGLVDWSLTSNVSGLFRWWRVLYIY

AGFNIVLLYLYQLPINFSDMIRWIASFIGLFRISLETEGPDICSGLFLVLFYIMLSYVRSDLEDMDFIMS

TSENNLAERLLPPKYSFFIRESRAGVRHTNVLLRGAVFKTFSINFFTYGFPVSLFALSFWSFHFASLCAF

GLLAYVGYIIYAFPSLFQLHRLNGLLLVFILLWAVSTYIFNVAFSFLNTKVGKFGLGMLVALGNLVNNSV

FLYLSEESSRSSNERSYVEADEETKVLVVATIAWGLRKCSRAIMLALIFLIAMKPGFFHAVYVIFFLMYL

LSHNINRKIRKSLILLCEVHFALLYILEIDLVSNSLKQEGSASREVLFQLGLLRSESSWDFLEIALLACF

CAIHNHGFEVLFSFSAIVRHTPSPPIGFSILKAGLNKSVLLSVYSSPSSSYSQDNTTYERHIASFLSAIG

QKFLSMYRSCGTYIAFITILISVYLVKPNYVSFGYIFLLLLWITGRQLFEETKRRLWFPLKAYAVLVFMF

IYCLSSFVSLQLWLSGFIDLYFYLGYNSKAPLLDNVWESLAVLIVMQLYSYERRQSGHYIPGQSSLLHPG

VFGFFERFLAWHGQKILFAALFYASLSPISVFGFVYLLGLVICTTFPKSSSIPSKSFLIYTGFLVSAEYL

FQLWGMQAQMFPGQKYAELSFYLGLRVYEPGFWGIESGLRGKVLVVAACTLQYNVFRWLERTSGLTVIKG

KYEEPCPLFVSAEDTTASVSSSNGENPSSTDHASISMKQGEATSNSWPFFSPRGNQGAGFLHPKTGGSES

GSSRKFSFGHFWGSIKESHRWNRRRILALKKERFETQKNLLKIYLKFWIENMFNLYGLEINMIALLLASF

ALLNAISMVYIALLAACVLLRRRVIQKLWPVVVFLFASILAIEYVATWNSFLPSDQAPSETSVHCHDCWS

IAALYFKFCRECWLGVRVDDPRTLISYFVVFMLACFKLRADHISSFSESSTYHQMKSQRKNSFVWRDLSF

ETKSMWTVLDYLRLYCYVHLLDVVLILILITGTLEYDILHLGYLAFALVFARMRLEILKKKNKIFRFLRV

YNFVLIIFSLAYQSPFVGNFNDGKCETVDYIYEVIGFYKYDYGFRITARSALVEIIIFMLVSLQSYMFSS

QEFDYVSRYLEAEQIGAIVREQEKKAARKTEQLQQIREAEEKKRQRNLQVEKMKSEMLNLRVQLHRMNSD

SNFGVASPRTEGLRRRKSPYLIPDSGAASPEIDGVVHRKEEQPIDEDSQYPFEAHEFPVSTTPEALDSPE

YSFGASPCEITEVQQDLDVMSMERERKQKSEGKENPLISAVQLIGDGVSQVQFIGNQAVNNLVNFLNISP

ENSDTNEQSSVDDEVYDEMESQKRKHTPFERSTSLQSDRSSDGTSFQIGRIFRHIWSRMQSNNDIVCYCC

FIIAFLWNFSLLSMVYLAALFLYALCVHTGPTHIFWVIMLMYTEIYILLQYLYQIIIQHCGLSIDAPLLH

ELGFPTQRIKSSFVVSSLPLFLIYIFTLIQSSITVKDGDWVPSADFTSRRNARGSQKDLTRIRLSQRILD

VFKKLRDSAKLVIRSIYRYWISLTRGAESPPYFVQVTMDVHMWPEDGIQPERVECRMNQLLRLVHNERCE

KGNPDLCPYSSRVHVQSIERSTETPNEALVVLEVEYASPTNGCSSAEWYKSLTPASDVAKEIRKAQHSGL

GEGTGFPYPILSVIGGGKRDTDLYAYIFGADLIVFFLVAIFYQSVIKNKSEFIDVYQLEDQFPFDFVIIL

MVIFFLIVVDRVIYLCSFATGKVVYYLFSLILFTYAVTEYAWSIYPTQQHAAGLALRIIFLAKAMSLALQ

AIQIRYGLPHKSTLYRQFLTSEVSRINYYGYRLYRALPFLYELRCVLDWSCTATSLTMYDWLKLEDVNAS

LYLVKCDTVLNRATHKHGEKQTKMTKCCNGICLFFILLCVIWAPMLMYSSGNPTNIANPIKDASVQIDLK

TVGGKLTLYQTTLCERISGDNIDLGLDLGSQSFLPTYNKNDIQLICCQADASVLWLVPDTVVTRFIQSLD

WDTDMDITFTWVLNRDRPKGKETVKYERSVDPLDLPKRSDIQMVLNGSMDGFRVHNLYPKFFRVTGSGDV

RSFEDQTDEVSADILINHANFKWWWSFHNLKASENISACEGMDGPVAIIMSEETPPQGFLGDTLSKFSIW

GLYITFVLAVGRFIRLQCSDLRMRIPYENLPSCDRLIAICEDLYAARAEGELGVEEVLYWTLVKIYRSPH

MLLEYTKLDYDA

> Oryza brachyantha PREDICTED: uncharacterized protein LOC102715259 XP_006645917.1

MAWPTGGCAGRFLLPLVLLAASLLDWSLSSLVNMIFFFAIRFVAPRRGFRAWRLYLLYWCTIVYSVLAIL

AQVTFHIIWCIEGEEWIVAHSWWAKLVGFMRNQPGKSPSVIYFLVVQLSATVLALVEVFGSRLYQDSCWL

NFSFGIEQLGYHLRAACCFLLPAVQLVVSISHPSWISLPFFVFSCIGVVDWSLTSNFLGLFRWWRLLEIY

SVFIILLLYVYQLPVKFPYVVLAFADFIGLFKISSNSEWPEVSSGISLLVYYFMLSSAKQDILDMDSLMS

LENDGLAEELLPSRNVFLVRQSRSGRRHANVLLRGSVFRTFSINFFTYGFPVLLLALSLWSFNFTSICAF

GLLAYVGYILYAFPSLFEMHRLNGSLLVFILLWAASTYIFNVAFTFFNKRFQKDMMIWETIGLWHYSIPG

LFLLAQFCLGVFVALCNLVNNSVFLYTTEGGASSSDDHLIDEKEDTMVLIVATLAWGLRKLSRAITLMLL

FLLVVKPGFIHAVYMCFFLVFLLNHSIKKGLRQILVLFCEVHFSVLYILQLDLVSNALERSGSLTMEVLS

QLGLSNNSTTKDFMEIGSIVCFCAVHSHGFKMLFALSAVLRHTPCPPVGFTILKAGLNKSVLLSVYNTQN

SRNGQADRSTHEKKIASYLSKIGQKFLWVYRSYGTYVAFLTILLTLYLVTPNYISFGYLFFLLVWIIGRQ

LVEKTKRRLWFPLKVYATVVFIFTYCLSVSPLFAELVSKFVKLYPDLGFDSEASLLKNVWQSLAVLVVMQ

LYSYERRQNSDKNFGVSDASESGLLGFLRRLLIWHSEKILSAAVFYACLSSICLSGLIYLLGLIMFSILP

KVSRIPSKVYLVYTGLLATSEYLFQMLCEPAQMCPGQLFHDWSVFLGLKHYDSGFWGVEYGLRAKVLVIV

ACTIQYNVFHWLDLMQTSLIHEGNWEEPCQLFISGDTSSSARGENEENHSSNKFSLLFSKVQGLIGSSSS

LSQSSGNTYQTSEPVQNETSGSDEGKRYSFAKIWGMPKESHKWDKRRIIALKRERFETQKTTFKCYMRFW

MENLFKLRGLEINMIVLLLASFTLLNVISIFYITCLVVCILMNRDLIQKLWPLFVFLFASVLVLEYFALW

KEGIPWLQGTNDIEVHCRECWKNSRIFFEYCSKCWLGLIADDPRMLISYYVVFIFSSFKLRSDRLSGFSD

SDTYHQMMSQRKNALVWRDLSLETKSFWTFLDYIRLYAYCHLLDIVLALIAITGTLEYDVLHLGYLGFAL

VFFRMRLEILKKKNRIFKYLRMYNFALIVLSLAYQSPYFGQFSSGKCDQIDYIYEIIGFYKYDYGFKITS

RSAFVEIVIFLLVSIQSYIFSSGEFDYVSRYLEAEQIGAMVHEQEKKALKKTEQLQHLRRSEERKRERNM

QVERMKSEMYNLQSQLNRMNSFTPINNASHSEGLRRRRNTKLYTDNDTLLEVSAIGSPTKENKTGNTDSF

HSFEFSVEDAQKNLTDSMFRTPYDTPRSPIMGTSDELKLTDNARNSLGERSEITELGENEGKVNPNLLKP

LNVRGGVKENPLKSAVQLIGDGVSQVQSFGNQAVTNIVSFLNIDPEEQCSSEHPTEGDIYDMVESQRETH

DGQLLRTHSVTSGTGTKSSANMPIGVIFRYIWYQMRSNYDYVCYCCFILVFLWNFSLLSMVYLGALFLYA

LCVNYGPSYLFWVVVLIYTELNILSQYIYQIVIQHCGLNIHVPLLQRLGFPDDKIKASFVVSILPLFLVY

ISTLLQSSITAKDGEWVPVTEFSFLSARNSVEETHCMPYYNWRDRLKNIHLPVMNLIKMIGTGISRYWMS

LTQGAESPPYFVQVTMEVNHWPEDGIQPERIESAINRVLAIAHEERCQSNSPSSCHSCSRVRIQSIERSK

ENSSMALAVLEVVYAAPLDCQLAGWYKSLTPAANVEKEIHESQKAGLFEDVNFPYPVLSVIGGGKREIDL

YAYYFGADLAVFFLVAMFYQSVLKNKSEFLEVYQLEDQFPKEFVFILMVLFFLIVVDRIIYLWSFATGKV

IFYLFNLVLFTYSVTEYAWGMELVHRSVGGLVLRAIYLTKSISLALQALQIRYGIPNKSNLYRQFLTSKV

TQVNYFGFRLYRALPFLYELRCVLDWSCTTTSLTMYDWLKLEDIYASLFLVKCDAILNRANHQQGEKQTK

MTKFCSGICLFFVLICVIWAPMLIYSSGNPTNIANPIIDVSVKIDIKALGGRLTFFKTTACEKIPWKYMR

AYDDVDPLDYLGAYNVEDIQLICCQPDASTMWLIPAPVQSRFIQSLEETEMFFGKMELILNWDFLRARPK

GKELVKYESPVDHSPSVDDVKQVLNGTRNSFRITDAYPRYFRVTGSGEVRRLEASIDSVSGELLLNNGTP

PWWSFYDTNPSDRAGCQGLNGPMAIVVSEETPQGIIGETLSKFSIWSLYITFVLAVARFIRLQCSDLRMR

IPYENLPSCDRLLDICEGIYAARAEGELEVEEVLYWTLVNIYRSPHMLLEYTKPD

> Dictyostelium discoideum involucrin repeat-containing protein [AX4] XP_640187.1

MIGYFFVSLIYPFSLICSSVFRSNVVSYIYFIFFLLSILCLPHKSLILKNKISKTLPIITLVLSMFSLIL

QLLVNVVKVFQEQDELSVNILTAFGFYKYNSFWIVFRNVLPDVIVFVISLFTIILWFKNLVYPASINIKD

SLKKTTSLNNVDQILNSPYVHGGSGNFNSGSNNNNNNNNIVYRPAGRGLTGFSFLMLTLSSISYPSIINV

IYFVFTILIILLLASKLSIHKVMLKCYPILLITSLCHLLFVYLNQIEYFYMKYTVFKEQKWYGVLNYTEW

DVTYWPLVIGYITVLLLYISTCILFRKQQLFNRTKPRYKQKLDKLGIMSDSNNNNNNNNKSRTTKLAIIF

SKHGWTICCSQILVVCFFLTASVASAILLASGLICTLLPLKVFKKVIYIILLYFLVFISAQYIFNIPFSY

SETDLQSYGLFSFNNSKWLYIGVQIVVSLTLSLYCFYSDIKDDDLGTIKKDQQSQQSQPQPQQQQQQQQS

SQNNQIQQSPLQYQQPLPPTPISNKSLPSSPMSTKSTTVHIQNNNNGGGGGIIRPRKPLPPVPLGMIGKS

SMAMTSNSSFGSNKPLNYIQQQQLQLQQQKVIGYQTSASINQLDINDSFSIAFPSLMLLTSGIGKGYDTF

RGKYGGTFSKITSSLKAIAEITFLAIIGQSYRLALVGLFFCGLTSINLLNAGYMLFFIVFVISESLASRF

WMCLIIYAQMVLLTLYIWQLSWISSYENDLTVLIGMTNYYGSPLWVGLIWHIIIITFSIIQWNVNKLYQR

GLFSSSSSSSSSSNNNQNNNQNNQNNSYEDKFKNIPNFLLVFGDFIYRSVQQLSLPFCYLVIVIVSIFTK

ISLINIVYMATVFLCLLIHHISANGSIHIKRFWIIIILSQGVVLVARYIMQFNQVSHWLNSIFPKSNYIS

LSDIGLRNYSSSDRFIELFGCSSILVVCVFQLTVFFSIGQQQQQQQQQQQQQQQQQQQQQQQQQQQQQQQ

QQQQQQQQQQLNTSNNNNQNNNNLIIKKYTFFDSLLYIVKRICYLHGPKFVLWMVFAISIAEYNFFNFIY

LIMIVISMSFKKGTYRIGSFLLFYSQLWVLTQLAALLPTVQSFNSDKFFMDWVGLRQPNVSTPWDAVKLN

LAIILVISIQQTSYWWNKEIQNEKLEIKKKKQLKKQQQQQRKLEEHEEEYEEEEDQFGNKKNNDKLSLLS

NDSIEIILDDGNNNNNNNNNNNNNNNNNNNNNNNNNNNNNNNNNNNNNNNQSNNENNENNNNSKKENLKK

RLFWYISNFYELYGLECVFLVLAFALFWRLNILGMIYLIIIAVGLNIDKRNLHKLIYVSALLAPTILIQY

LLILVVPTKENSYPWLDHPFFLNHKTIDNLLLLSIPDRYVLVIDFLVLFFSMLLFKQRNGYYLYKDFELH

QQQQLNQQLNQQQHDSLKSIASSNSSSQKPLHPKNFFKFNSSIDGGDDDFTKEPRSWSNELRYMIIRYSS

QVILIVIFLAGTAECDILSCFYVFFSVYVLFSGNAHSRKWSYLWKSLHIYNWLVLMAQIIFQVAVILYFQ

FKFNSNQMFEGHNHNHNHNHSSSSSSSSSGSIIDILSSQSSAIGIGGGNSSGSDSSYEVIENSLPTELYN

IAVVFGFKIETGPLSISTISDVIIMVLLAYQKMIFQSRDFHILEEHLKAKRDLNYETAREFYKIRRNARI

EQLNSIQDKITQRRSRLQHLKLKRINRRKNRHNHYYNNNPNNNYNNNNNNNNSNSSNSNNNNNDDDSNEP

LSLGDNSFVPPKNTTNQNATNSTYSPFANSTMHMPPYENNNNNNNNNNNFNNNPLSNSSSTVSSFGVIEK

PLEKKNWVKIYLNKILDWLDPLPDILIENYKKQQLQQQQKLEMNQSLLFGDQLQQEQQQQQEQQQQLNPQ

QQQSQSSKELPPILEQFEHNDDDFEISLNQAPGDYRNSIFIDSEQMRNAMQQMEQRRQQRLQQSIDASQL

LQQQQQQQQASSSNTNTNSNNNNYNNNNNNNNNNNNNNNNNNNNNNNNNNNNNNNNNNNNNEIPKPNQTS

SSTITLEPIEDEEEFLYKEKTQYFKRIIRGFSRIARDESKWLVFMACIANGVFYNSIISLVYLLAVFLYG

RLFESPRPSKNFWRFMIGYSSLIICLKYVFQIPKNYYNCNENYHNSNNGTILMTSTSVYNTNNNINNNNQ

LNNNENYEWWQCPNTLLSEQNLLLTLPYVFGLYIIDGHFISGAFWDLAILLCCLWHRHVYRSKGLWNFQE

KDFYVDQKQQSPLNLFNLDQQHFDQQQIDQIQNQQDLNNSPISLNSSNNNNNNNNNNNNNNNNNNNNNNN

NNNNNNNNNDQTLIDINNNNNNNNNNNNNNNNNNNNNNNNNNNNNNNNNNNNNNNNNNNIIIDQILVDCS

NEYDEEDQQQFNEFYDEEFDDRNEQEKENDEQEIQVIKKSSKSIAKIIIYPFKWLFVSIIEYVWLAIRTD

EKPGRDYYMPLLFTDFACLFFLVIFPQNFTGIPSSDIAEFLEQNVIPRQYIVILLAQFGVIILDRIIYLY

KSVKAKFVLQIVLTVLYHVFLFFYFPDLIVKPFSFGYTWPLVVFYLMKCIYLYYSALQICYGYPILSQNR

FLMDGYSDFHNIGYALYKAIPFVYELRTLLDWIATDTTMLFYDWLKFEDLYSTIFSVKCRLEWIKRQGRQ

KGHKQPKFEKFVTGVTFFIGLVILLWFPLIILSSGLPGSNIEPVNNIQIEVSVVGWNPFLKINQDLSLES

GDGDSTMDQNSFNNLKEDYSFLTTDDRQGIQNISINTFSEEIWNLSPPAKAQLINYLLTNTSLQIEVSYT

LTRSGGVNSVIVGSNSVQLKPDQELNFYNILTRVQSNNSNNSNNPNENSSSGSDDNNNNSNNNIGSNSFI

VEGLFYKFIKLPGVSGNPIYPTDNNGNVLALDALFTMNSTNFNNSNQPPQYFWQVNAYDSTKTYNISTLE

SIQFYTISSKLPNGITSTLVSAGIIGLYVSVVLSVGRFLRLSITQISIKIQLENLDSCDEILKMIDDVFI

AREYGDLVLEEELYHELIQVFRQPQLLYSLTTFKNNQLNLPTTPTINSTLNNQNNQNNNNNNNNNHEKIN

>Tetrahymena1 thermophila hypothetical protein TTHERM_00031690 (macronuclear) XP_976967.1

MLFKSKLTKREAMMAKDYEQQQKLMTKRLESETVHYIKEFYKLLNTSYERIQEWQQIQLQESNCLEMSEQ

INEMKKNIKQFNKQRKNNRDINYHLSNKIINAEIHFELTHTLNLLTSITYKCKTLTQVLMEKIQNNNKEQ

NYLDVSTNQNQQQSLEEKSIQSQFFQPLCATGLLTYQVLKAIKIIIKNNQIISLSMNQQEEFIDNLLLIM

LLWNGWKGKIEKKIFKNSVIVIESCLVSKRFAFNASILKNINLFIEKIQLKEMSLFLRVLALLVLEVNDK

QSYKNISKGGGMNYTNNYLLMNHSLLVKQPIFLSKVFEIAESMIKVLQCLMQCESEIQLHRTVQMLNKSK

YLEQSGICISKLVEKLNRKCQQQSNVLLNKKSIIQYCLQNSSLMELLFILQNLLSGSKKIIVQQQLNELD

FIEKILTPFYDIFFHQEFNLDQIVCEDDEDTEDVCFTARIQLLRIIIQFIQRDEDNTNIISKFISTTEHF

YLQNVEIPLLLHLHHNSQSAINGQENKNKSQEVDGILKYRNLFLETNGKDILENEDYLQQIKSEEMKKKI

QDLQYPSQISEKTIVQCRKDRLSYIEDKGLMHKIIESFKSVNQNCQLRFWLGSCLEIFLKGSNWYHQVFV

FETSLLYTLIRQYILNQLSTTNNIQITLDVLGELVKFNHLNVIYLNLILNQEGWITKIEQKMFSNIVDTN

VFLRNLYLTILCESNNFRTLVELIKSVNEDNLNQENLCCINSALVILIFSYQKGHFKQILEQIFKYFKGQ

QTLSLFFQVINVWQNYHQVKISFIVLNQQENNSNYYENQVEKPQIDKANKLMADQSNKKLPQRENSLKFT

YTPKAHNENYCIQGNSECSDFQNIIQCIKDQNILKDKKINEREQNQAGKCNILTNIRYSQGKQIQDLNQD

SQQNDGESAECVDCFESQENQKQNTTQEIQQENKCIYNHLQFDLGKNPQRTEEQIINTKGFKAKKQPALI

KNMKKWRIKQNLKNKSINQEEQQQNQQSFKQLCDTTKQIVNIQDNKSNSQKYQAVTVDNSLSKFINKIEK

YQKNNTNNHINQVFKQQYAEKSISPIDSSKEDSIQNDKNNILSEIENELQKPEIINLALKCTEQQKHSTQ

NQNVKILQSQQINGTPFQLIKQKFLQEKSLVRNPSLQKNQFNQISSNSTLRNVEQILLSSSQSNADTQAK

NNRSQNLNNNKFSKKRNISVQMVNSSNNALKANQSQNQAQEYLQNYINTAETNHLLLSNLNKGIIQPPPQ

AQQQETLFIPKVMTRNASFLKKGNNLIQNITNFQNSSHQFIKQIDNKYNQNKVNCKENTSVPLSPSLNKQ

NHEVFQFNPFNISNSQRSLQTSQNFNFQKSLKSYLFYQNKFMGQKQHEFLMNNFKCLPKLNSLLNQPQPN

INNEANNQIFNNFSKGLISKLNQSQSEKFISNKQNEQFQHQLQQDVSPKRVLVCSFTNQYSCQQLQKQKL

NEIKQKEVIKEDDNINQEEAGIKQITQESQQANFNELSNYYKQKYEQDLNNKNSIPVQFNNQKGQKLDNI

PQNRILRKKNQSQDLRYQYNENEDAKFSELKQRQASQNLTNKMIIPDYSNNNRNNQQQREDDQGNLNQEN

IIRQSRRIGTYARQQSNRNQIIDGENQQNQSFLTEEQLQKRKELKQSILRGIICFLIPLCMFGLYAIFLK

SYFTAFYILFAGLLIIFTLESFYVSPQMPQTDNKNDNEISLLNEEKVQLLDFRIYLWIIGTFATASVLIF

KLFMITYYNVHNEMPFKASYNIMYDLEIYIVDKGGQNWSNILKMFLPTSLVLINCIIVFIFRVPRKSLKF

QDIKLHKKRNNLINFCKTLVWTCLVAVQSFNVTLQAYIFYFIFFLSLSSLFFSSKIKNFLDEYIKHLIKL

ICLGVLMLQFLCSTKSFRDSIQGQEWSFELFGLNYISDFKDELTYTPLYFISMWCLLIITVLCANLIKFE

RLVLKVREISEEYQKKYGGNLPELSYEHSSSNEIVILSSDNEVNSNQLKSNDVDPAQSSSSDSDSSSDEE

DDTAQQLQQQQDIRNQSKRVESIQIRQERGISLQPQQQQVAFDGQEIQVQPENQLEQQLLQQEQNIQQEP

TIEQHQKDSQLKEENSSKTEKAVKKMKTLGNKIMLKLLKFKRNFKSDTFMLRILQIVCIFLVQRYFTYYS

LGLMTWVLLTGFTINHRFTIVTSFVFAIPSLVLIYVFTVFSNISNNPFIPSWANSQLGFQTCYTPWLEVI

ILNSGLFLYFFYCQQALQSFKQKKKEKEKKKTGVKFGTKYIKPHVILVGLFLIFFSNMNTTIQIPRQKKS

DSKKGTSDRRSQSGEQVMPTPRGIRTFDVTPQNVNQAETLQNPFNILDSDRTNKLKSDNVDASDFNLTKA

GRGSQTVIPRSKKFKQVTFGRRYWILMDQFAEDSFCYSLFNFIGISQPYNFPIHLGLSHQDPADESIVWI

LFYFIILQYDSYKSKIYMKYSHKITEIFAKKALIKKVFPNFISYVSSFYIVFYSILIWVSFIVISGVLIL

VDFSFINTYLLVYILLLVIYFVKKNANKKLNYVVFQRTWYFFTLNLIIVSFTRYFLQLTSVQFVSDEIIN

ENNSWFKTFQRNRSVYGFYSDNPDDNLRMKFLPGIINIFFAVLCLDYFRLISEGYKFSRELERKRIANQG

AQDRIRENSVSSYGGAASSRKNSDISIASSMFDDGEIIESRIEKQKALETLKMYQDYVQVDILLKYKWMT

IPAKWISLLFTDVLCFLVIIFCIYFKISLSMLYFLAIYLFFYFKTHSHILNYIEANRFFEIISEKMLLFQ

EKYLRINKAPDNILNDKQQAEGTPIQQQKEDLKESNEYIQKQNRSFTQKQSEEDQLDEEFVKKINRISLI

SLEIKIREKEKVWNYSFYGSMIFLMITYFSQFLLTSFALTNVGTQICYWIQWGMFIFGTQITVIDDIENT

WRNIYFYVLLFWLNVLDLKCLRYLQSIIFEKENEIQRETNDQDNQQNAVQVETDKLLEQNADNQQEQEEV

KDQSEYTPSSKGNSYIFIYHKLNIQQIQLDDISLKKVVSQYLGNKKELEEISEEIEEEQANFIDKKLVRS

NKVARMSLEIFAQDFEIDLNESEFKERMAEINKIHFYTQGAESENLFKYAITQQNKEQKDPFKETYSINN

KSFSEETIIEYAEYINIAYYFKSKKNFIEMRIFYSTLFIIQRIYVLILLFDSNSKQNAFSWIYLILAAYF

WVRTPKSSTTSLIPIPSYLWNLKNMSVIDNILSDTQIQSYLAFDNSDNVALLLNALIIFLIEIYFMYFVF

ICKFIMQRIRDRRSLSSLISQKVWINFRAWKSSYISFITTVYEVVYTVLKVPQIDNFINTDDTNLSYFRQ

LLKQEVISFHISEKVFILFFIQLVFDIMLSKDFKEFLKTYQLTNQVKQRLRRKCIAYQFNNEKLKKIEKR

LQENKELIRNVQEVQNQLDNWHATIDMKSNKPNGKINSTMYEPDEKRSTSSLNLNEQVVKAKQIEAAKKK

EESQSNLDTFFTPEELEREKEKKSIPQFKRYLRKFATLFKGMRNEFIFQDELGMLDFVIKNNPYLLECIK

INLIEYLTGDLVAIQAILDFLDNLYANIFLKIRTQSIDEFNPDKLKRNLQRAASANPDILSDFQFLKRNN

SMKPNEIELFRKESEIYSNYDRVLLFDSNQRFKFFNLDEERWFKEKNKGRDTYITCILKYLGQILISGWS

YICYIFFIFYYFKNNGLTSMFFPLFVFLYFMVEEKIGEMLIWEISFFYISILIIFKFVNSLSGLIQQSDQ

SSPSDYNITVFFDSNDSLFFEGFLYFLIYIQLNLIRRMGLDKKMINQEENTHMSYVRLKVNKFIKTEIQP

KIQRKDLNDDFFEINEEDSNLEYDTDLQDAEAESRKSSNFVLLQKSLPESLLQVQVDNFEQGEKDESRLI

EQEDLKKIQEENIKSQTDQSNTSESDFSINSIESEFTQEGFYKQLEEEISEKPDLIDPEVKTNFFFRLFS

KYTKKSGVDLYPSIALIQVILLIYIFLFYNEMQNGQQDLSSSLKYNQFSGSMVIFMIIQIVFMVWERYIT

LYTSEEVKDKEVERIKLIVKARSQGLAYSKNLKNLKALQKEITELERLKKEEEQFQKDLQSSNIRDQKQL

QDLKKGQKGNQDQSEEDKKKEKGKQFKFSQILMKYIQLFMIVVLFTYFIFFFIPQPFFKTISGSIDWTHY

QTNGYLGGLYFFFGIYLVLSCFQIKIGYNQFKIRNTILTKKNILTGICAKSFRAIPFIQELKVVMDWTFT

ATTLDLFQWFKIEDIHFYLFCAKVDANDNKKKPVGMPQNWIMKFFMGWVFLLAILLLLFGPMILFSSLNP

STQLNNITNASFEMGLQINGQSGNYYQFYQNGNIGNLKSVASSSTNMDIYQNITAISQASPSQFQQFSLY

SSSDVNWQLSVPSITEIQQTLENQIQGRTNIQIQFSVTSLSSQDNTFLYPINTQNIYDKSTSLTQEQVAQ

LLVVMDDCNSQAMELPNFYISLLRLDTNGDSFEARTFSNLSPSQQIAYFKSAYLNLKCKLNPDQTYDENI

RYWVLSLDQNGQNGLIFYVLCDKYSSVLQGLSIITIYTSVILVIGKLIKSVFSGDIQLLMFTDQPHSDKL

IKICDAIAYSRTERDLIKENLLYWELIDLMRSPEVVKMLTGSYSKAIINKRKKELEEAEREEERKLKKEQ

> Tetrahymena2 thermophila hypothetical protein TTHERM_00151710 (macronuclear) XP_001021704.1

MVVAAINDKVKPGIVPLFFYQPSMLKQNSDENKSLQAETQPTSKSNEQSLQNTQQYRILIQIKGQSIYIQ

MELKVQKTQIIGKKEIKKEGKDKKNENKDGLEKQVSKWYDGKVVQWIFCTFIPFAFLAAYPIIILSYYTI

AHTVMAQVFVQIALINFYIKPSKSSLDSKEQQLKEIFIKKQTIQLKDYRNIFFKFVMILESLNLLSKIIF

VIIYYSKEDLQIENIKNVLLDLEFFFVSDQAKQSASRIYKNHSEVRIEELVHPSNQKAQGLLNFLVLVSL

ASCQIFNPSIMAFGFLGLSLISQIVSLISSSQRKMINAQMIIQSLIQFYCFAQFFLNMFSATNTFLENNY

FESQFIGVNSIAEFSNGLNKLNYEKSRWSFIPVYQQIEKEKKKNRLKKALVKLLSLKKQNSNILETKSEL

LPRFDQDSNNQIIHYHTINDQDRVQIELQTYNDIRTEQQILDKNQDQSQDNADALNHLIIQDIMVDEIIK

NPNTQGQKANRKQKMTNSLKLGTAQVNFGNDSKGEIQQDDNDNNQNQHTILSKIRKNNKSTTYTPGLFLK

KQQNNQQRGSFKVNQHEQDEQIQDKVQKYAPSLQEQKEKGIEIIRQQLIKIERGEEDNIDNFLTLKKQDI

LYEESIQPESYLIANQKTSKDKFSAFKTKLYNYILDLSSRSFIYIKSQFEDSIIPIRFLQCCSILIIMRY

PCYYSLGLISWCLLSSIFKLEIKIKFLFITLVPIPCIIIIYAFNLYGSIQLNYDLYPKHGFVYQRFFWLE

SIIFNTIIFLFFTYLLILRRLKQNLSSIVQSQKEKSQEKIEENSNNKQNLDAQQNDNSKSMIEQNIKQIG

EQNNDFIQQRNSEKQESVSKKNSSPYNQDQVRFTSKSDPIKGEINLRFSKQSQSSVAKLKADQENENEQN

QRIQSNSYTKSDKQENDNKNSQKIDYSQQKPVSLMTIFFALILKYSQTLALFLLYWIGMNYMGEQNSDNN

QDSYLATIINFIGINYEYDMPFSLSLSKDDYAGLSIIWIAFMFATLQEDTFRSETYQKYSNKIIFLIQDQ

PIIKKYCPNFLFYTNKITILAFCYYCRSYQYSITYLSTRTLKPLNIKNYIINPNSQAYQTFKDNRAIYGF

YPELGVNDNLRYTFFPIIFVLYFAIFVQNYLKMIYNAVKHHNKGQIRDKSESNCSVISDNQKRLLSEREN

ITIDIINKNYQKARREENRQIYINYVLFDIYSEVSSGVYQILKGLQFIMIDIQNMLVFLFAMVFRLSISM

LIFIIIYLKFYYRYHNQLETFLEKSYMKKSINYFFKSFQKFYSFPQKNLGWKINLDPKPPTSNKNGKKQQ

EIQQQDEVIISSNQLKNLQVEKANDTKLKEQDNAENEQNKIEQEQSQKQNSLSQNKNLQALRRQSKKDLI

ECKIQEKIEDFITIQQKLKIDEQIGIWPYSFWFSYICISITYGAQFLATDFAKSNISNYVINFIQWAFFI

FGTSCNTLSLSSIFRNIYFYLIMFWMNIIIFVLIRMKKQHEQNVMNQKQELQSQSQLNKNSQIQNRDSSA

KKGESNQNNPLNYDDFRASKSPAINSEFPRNSSSYSQQLNRLTYLHLNQAQVADVNIQEGEQKLAFESER

LKRVQKSEKEVGVVYYQEGGLLPQRITWCKLIYKHFKLTEEESEDEDNNEAFIQKTQFIEGLSPNYNHDN

IFKQQNKDKTDSIIEKNYDDAFKNEIENSIEDEGSINQIKNKLDGYAFNVQSPNILKDQYEQKQEQSDEE

KKQELQDEILIKKRIQYFYQMKSCYVKQKFKSGLLLALYPFYSVILMHVSEQKHDVFSYIYLLVAFLGNS

IKAINRNTIFLVLIQYILLLVNINQQTSPLQIDQYLLDFKSMSLIQQYITDKELLQYLAFNSSNQSSAAT

ISFILNCLQIAPISIYKIDQAQKKQQENQNGSVNNPDSDQIVHDQILKNQKFQLSNNNLSTYSIEFTDRS

DSKYEYQYDEDGQLINPIQPLWINYQNWKSQTFQFVNLMYEISISFGYIICAVLIMIISCQYLDIFNFII

LAYNMIIFFIIEFVVPFPQMLNQRKKIQNLLRVLQFYLYAVFALYSTMQIPYVRNNISATGQFLSYQFLN

LKIIDKIYIMFCIMCTQLLISSQDFKTITFSLSQSYILNANLSHKALSIHQNDQTISELLKNEKERRSLR

EVVRKVTQTLKNWHKSQSYIQPSIVQQQNNQNKNLNTSLQINQFQSSKNLQNTLPARSQSAHNVPLSCFS

NQMNTRSDSKDSILYEQQGLISNQIQRGFQNKSKSKNIIKNNPLEAQTYQKDKFQQTPINKQNQETVQDF

QKNDQNSPTILINLASNEKQEQNNNQLLQNEEFSKKPDQQHITKEKALQKKDVEIDELDYSSDESIGNID

EVNHANSGIQIKKYDFPRISQIKINNLENLQQQQFNKQDQLNNSSLKRMKISRVSSVDHLEQNLLFDQQS

ALKKSFNINQQKDTDSSYTTRKVRNQSKEKSVCFQKQKVVKTSRKKSLKDKKQEKANKYHNVENIQAVKK

YQKDSDSEDSEDSQVSLFNSSEIVDEEDNDLMDQIKQQFDNKIQKQKEEEEALLQGSKQAKRETSHKILI

KICKLIESQVNPLLFMDTVQISDYILKNNCQIFSKIYINIQDLLLGNLTSLMEYSHLLSIVYYCIYQTIQ

EAGVEFAFNNLFAIIQRATECDEISKIIYDSLIETSSQYPTIPYYYHLRKEQEKNITDKKIIQREQRNNL

DHICIYHPQIPMYIYNVKSTNSLPFNLLTKFGLLKRLLKSFFKMVLSNWQFVCFFFFLMYYFSNTGLMSM

PFPCLVFLNFLIEIKFVSLQIWMISFIYVTFLLIFKFIIQINGIYMDTQLQNTFFNDNLSFSFEGYLIFL

IFVAQIMTKYIGMDKEDVYNSENIYQSYIRKCVNDLEFRQDFIENCLQPTNEDNMLKNIDQISPIYKKTQ

DKPNLFHIRQMSQQQFDGDNQLFRDENLNKNTQNLLNKQMKIERDQSTENQNDFDNIGQLKKQPSLNKQG

SLQHKNTFKQNPQLFLEDQTLSEAEENNKIQIKSNQDILLKSFNQGKNNLLSISEQERSISMHEQMLKDL

KQKPDLLVNDTFKKENFFKRVFRWTQITLKVAFMVFERYLALRRVSEKTKKEKPKKKKEKNSSSSDNSND

SDLSTSNNLSQTQIIDEEDSLIQKKQKANKAFDSFDEKAYSRQNQPRSQSQIKRKPTFKKEVNIKHLRSS

TKISQEYDTYKNFQEMQSLVLNQEDDEEIDNKKQNKILKDTYKDALKQQDHYEEKQIDNEKQQNKKGYDQ

KDFEIVNDTPQNRVRSFASHTRNSKEYDEDVFKLRQQHRLSLVLREEIIFANLEKEQINANLQNFKQYGN

LITKNKDSNNLDSSLSSSDDQKSFLQDSSVNLSQGKNINKKKSNQNTPQNKKGQTRDLENQIEETKKSSN

QKEKNQMVSYPTDNIIKYQDETEANQNAINKQKKYNDPVILPFEQQQGIQSSQDEDNQDFKSDASASDNV

SINKNKKEKPKESFVLTTELLKFIQLTVFLILFSWFVFFYVPQSFQKNTISDQKVVVNYSTNGYLIAFVF

IVMIYLLLSTLQLRYGYKKLKLRNTILQFKNTVAGFCAVVVRAIPFLQELKVILDWTVTKTSLTLFQWFL

LEDIHYSLYVAKLDSKDVRSTKVDAPQSKLVKIFLGLLQVIVILLLIFGPMLLFSTLNPISDYNGITSGE

IEVGIQFKQLGQINYYPIFTNSHTNNLQTVQRDCTYDPQKNVNNQDASQQECYWDEYKFRDVNIIYTTDS

QQFQKFNFYSYSENSWDLSFPSKKDIESKFEDILNNQNTSQTAQLVIKLVLNRKVSQYNTAPYISYYPNP

QIQNSSYLNKQIIQGFYDTLTDCQKPGVILPNFYIFLLSSTISNQSYISSPINHEDNNNSAYFAKDSYIQ

LKCQDIQNPQTQKYWEISIDQAGTSGLSYFVVTDKYSQATFGFSIITLYTSVILVIGKFIRDAFGGELEK

IIIKCMPNPDELLRICEAISNARNEKDLLKERILYFELMDLIRSPEVLKKLTGSYSFVLNQKHKLDLQQE

EIDKAFKKRKRLIKKKLANKKKKEKQRLQKLLDENKETQNKEETQHDEQNKNFQIEEQAEEEEEYDQITI

NQFLKK

> Tetrahymena4 thermophila hypothetical protein TTHERM_00340180 (macronuclear) XP_001017682.1

MVFIQINKQEDIFLGKGEDFRSSTRKTVNNQNTFQTLVAKYEWSQTLITISIVILVIATVLIDGVFGVIY

SIILTSWLLTKLKNKGNELQLQIDIDKKYLTFLKYFSLLTCYCCILWKLNVFLYSTFYYFFHNGTTAVYA

ISLCLRFAFSNQFITYICNKAQIREKQTKLLNYKNLFDIQLFWYCNRFMLHLVQYHSIPLDVHTSTNLAD

WQCEQKTFVIAIFNIPFIFKYVTGSLLFDLLGLHSYNEEENGVDGFLDRMAYLSLYLITIGSIYYITLLK

IYDKESQKEHYHLLAQQQALHQSILSSHQSQQHSISLNQPSELTLPPQRADDDNFVDINQHQDQINQHSR

HLESDLRSSQLRQGESVNESQLTQQNNNLKKQKKKTKSKFKLALSAFWKELKRTSLLHLEKLNVFLIFAV

SIYKIDVIHSIYMVAFISLVFSHMKYWRNILRAIIAYSYAVIILHYIFTVLYEAKAIKQVSQTWQIIGVN

EKPTAETFLTVQTFEIVIVLIFTYCQANIVYVTDTYNIYRQQSIELEEEATTLQRKQQIIKQMDFFSRFF

ISYGVWVCYINLLSVGLIPPANILSLGYLIFLQLFISFTMYENHERRALSKIMKGWPLLVFISSFILIVR

YLSLFSALNNILQSILPSKKVIEDIGLSNEMNTLQICGNAFILFILNAQKRLIDAKIYEEEQSDIEQQDN

QSNDLDKLVLFYGLLLSILHSSFAGVLACFAQMLIFLWGRDTYWQRVWIPLWLISFVFLLSQYLFQLHII

GDHLSDAEKSWAGVFSINSSRYWVFLILYIVQQILCVFMRKLQKFEERLSSSSYQNFVQDYQTVKQSYQI

EKKRIDEENDKELMELLDQEEREKYQQEISFKIQQQSLSLSNKKRKKDINSDSLNSPLNKLSKSMIRRHK

QSGDEQSELQKSQNKINYQTYLKWNPYKKGIDLEYELKEFFKYFGYEISIFILIVAAYFEMNAVSLIHII

LAVFFALFSNVINSQSMYNPKALLFVNYIWRFINYLMVLDITRKYIISIWFPQDWKVNKPWNSWVFTCNK

QGKDSYSPYLNHGVEDKSDMISDYDSCTHDWRYWLSLEEESNLHLILDIINFQIMVIYDRYFREIYQHNG

PKRCHSDIKSQDQLDFTRKNDIFSYLKLFIFGYFYQIPIMIYFIIGCLSNISSYTDIISIVYVFTSIFML

FKTKMLLREKNSIWKYLLLFNTLVMVILCLYQSPFFQCPIVYPDDRYYLNSEECLYLQNSKKGQLYILEV

LNRKVDSIDDLYILLSHIFGFNKSHHFHFGFTKEVFMIIFVLFGLLQKNLWNHPYTQFYIEPYFKEEKEK

QKENAINIVEALHVARISSNFQQEMELEVYDSIQSRVENKVVIWEKMVSSGDESNNPKLQTEEGQHLLEQ

EVNKEEAQISQAEEQQEEGQQKLSEKDQEIIMHKAEKIVLESNIPIHLKDVMKICTQYSKDQEKLEIEIE

NLKTNSLQMIIEMVYDNQYIGINQFKDQMILLHQTRRKQIESIALEKKDKDQLKQEQEQDQGKDEVQESL

NRSAQEVQQEENKLQELVEQVESVSPSNQGENAQKESEQEMKQKQKNFKIIILKIKDLFKAIKVLLINMM

CSMLSELSKDTINLYKKQDSLSLIFIVFMQRKFQLVCGFAFSLNAINNADIVSVIYPIIYLTYCLVESPF

PTRSFWKLIMYYTILIITLKFLYQLPVFCGTPPYTFFLLSDGQDCSTSNITSEQARTRIDFIIGIRKYTG

PASYPRDQGFFSGIFWDYIILLCLFVQRYLLQLEGRWTYVFISKDECYIPQFRDPQGEYSKYHKLPLDQQ

MKHSFSLDGITDATINYSEYESQEQQQEDYIEDQVLSNQNLNQEQVADVNQHTINPSNQGEENLDLNQRQ

RDQVVNRNILQDLNQSLEEEKDQVASLHQQQSNNSYDELIQQDNEIKEEEDNQYYRRINFLMLFIYRLFP

QYMQNFYKKKIKNEINFNKQKQQLLSTEEEKEAAQILHDAKIESLEMKDEDIQGTKPGRNFYIHQFVSSF

VVLIYIILFYNKMASQSTRDITNETRFSRSVVIIILICITIMVMDRILYKLRRIKSYFIDIHESSEQVEE

RKQDSTQHAAIIKLIIHILITICSHIYLFFILPDQTYSRFHENSYLIVCYMLILVYLYFSALQIKFGYPQ

MSVGSIFSRDTSLVYRLSFIVYRALPFLYELRSVIDWTFTSTSLDLFQWFKLEDAYANLYSVKAEMNVRK

ETHKFGEKRDFVEKCTNGFLFLIFLLVIILLPIFLFSNLNPAVELNNLTSGQVKLEFQVLLTGKEYHVRI

YQKNGIEVKDFTDQQFSTFSDLFYAVDDLWQDRLQVIKMDKFSDKYQEFNENMIKDLEKSFNNPDMQMGV

LIDFTFNRPKPEGKEQVRAKQVLNFDKDQMSKMKQILTDMRVDMQSQKKEKYKEYKISLYEFIPFFYQIP

DGQNAVDRAFEPQHKDELDKYNKSLPQDMLQDPNFDPNVLKYLDAELLFIMEAGEYQWQIKRRSSPYFDK

IVPNVDDRIAFITLSEKTFSSILRSMSDNLSVVGIYLTVVLTVGRFLRLYFNNVSMRCIWEEMPDTNDLF

DLCQSIYIARLEQNLMKEERNYELLIRILRSPEILMRLTGKSLYDI

>Hydra2 vulgaris PREDICTED: piezo-type mechanosensitive ion channel component 2-like XP_002160109.2

MRKRVPKTFLYMLLTQFLLMLFDRFLYIRKYAFVKFIYLAILVLLFHIFLFVVVPGITQREFVYNIPAIF

LYIFQCMYFGLSAYQVRCGYPTRILVNFLTKNYTITSAILFQGIQAIPFLLELRSVLDWVCTKTTLSLNH

WLKMEDIYANTFILKCWRNSEKYYPHPRGLHYWTTSKWLIGGLLVALLIGVIWFPLLFMSFINSSYIYSP

PSEATFTLTLGGYQPLFKVTTQQQFLQTLSQESINAITKSNHSDNWDASKVEHGFEDYKMTGNIMSVEVL

SNSSSIWTISPPSRAFLINDLKSNSSISLRFQYVFNRESNNTEVTLVQSTVSWVRAISLPPGDPIRNSIA

EVIKSGKGEIKIPRLFATFVQVPARGSVKPVDWLGDGSYVDCTIVLRNGSIPDFEGYAAQVEWWEIIQNK

PKPMFSHDAGNLEIIVLNDFVPPLHLSFFANTGIIGLYVGFVWLIGKFVRLFFTSISYRIMFDEVPNVDR

ILKLCLEIYMVRESKELKLEEDLFAKLMFLYRSPETLIKWTKYKRQ

> Hydra1 vulgaris PREDICTED: piezo-type mechanosensitive ion channel component 1-like, partial XP_004212490.1

LLDIGLRSLEKPTLFIELLIPTLFSMSIVVQLHFFHTPLVKNINQILNNRKFLKTNKLTAPIASCSTEAS

TCARTSTELDKFDEIADNVDKAPKFKKWYDDFTSILWLFAELHMPKIVCFTAMIVAIQQVSAINAVFIIM

IGLSMPSKTLQYIISFIMIVWSSLVVLVYMVYQLEFVKENTLERNCTNSSLPVLLQVHHFSSNAWWLGLH

KSTSVSLDVR

> Nematostella vectensis 248469 predicted protein XP_001622392.1

MFDVHTLCVQDINVASKHFLVADCHGSKSQIIKKQQKVLNAVETINWPEEKKESEGYRVS

DVEDSGESLWNKICRSVKAFLNRIYDPETWFQGRGQKYWMLSKWTVGGLLVALLVIVIWF

PLLFMSYINSVYTSNPPQDATFTLSIGGYQPQRQAQFVKIVEENVVPKTFLYMLLSQFFL

IIIDRCLYLRKYVFAKLFFLLFLTVSFHVFMFIVVPYVTKRPFFKNIPAIFMYIFMCMYF

GLSAYQVRSGYPTRILGNFLTKSYTLTSGVLFQGFQAIPFLLELRSVLDWVCTDTTLTLY

HWLKMEDIYANIYVLKCYRESEKTWFQGRGQKYWMLSKWTVGGLLVALLVIVIWFPLLFM

SYINSVYTSNPPQDATFTLSIGGYQPLFRVSAQEQFLERLNNTKIDRIKTQHPGLHQGAD

TQPPLQVAIIFNCVLKIQIDTFLADYHDRGTVVLVKITGNSSSIWQISPPSRQLMMHDLL

GEKNVTLRFKYEFTRIIGLYVGFVWLVGKFVRLFFTSISYRIMFDEMPNVDKVLKLCLDI

FMVRESKSFKLEEDLFAKLMFLYRSPETLIKWTKYKRA

>Trichoplax adhaerens hypothetical protein TRIADDRAFT_55613 XP_002112008.1

MASIFVATLLFRWFLTLSLLAASILRINGFSALYFGFFLVMPFLPNPSHQSFFKGPIRKYLLSICALSLG

LCVAHIIFQIVLAANPPYGSIIIDCFWQERLARQIGFNKLGHNVWNDIRLLAPDAIIFIISVVTFLYCRK

LANKQVDQIMHGSDDDRRHVYDKLDSLATLTYSKYGHSLKNIKILGLTGYFKTNCSSPSLPILRHGIPWY

SYVCPGLIILTFWTFATHYSFSLKWNSYGKFPNLATHMYFLMWWTSKKRSETKQRSPSTQLQTENPLQVN

VDAHQTSTLNQDVTSQNTVATNNDQRTEAENNVLLEPVPTAVETDENLHIHNIILQYSQQKFYAFLQYVE

MNIHLLALIAMLTWSISYHSWPAFVLLIFACLFFIHPRTYEVMMTTAPLLVIYGEIMIIATYIYGLNLHK

ELPEVVNGFNLQQVGLQRFSNQCLHLGLQSIFLAIFWLTLRHKQNRARNSERELYRVIGITIQTLLAQYW

ILACLGALLIVSLHGQPTIYQIIYMFFFLLLTVTYKIYFKTFLDYTYNVFVTGTETHIVKAVMLTIFIVC

VAEVTALNGIFAIFVILQLPYQRLQKVLSYIMTLLIAVSLLSKLTFQLSVIDTKNLRSNCSYAIPEWNGT

MFITFYLNRTLNDIEWFGLKKVSNVSSYVGGYIAIICLITMEKVINHRQRQMYELTNTRRPPSGILFPNI

DETKIDTSLKSRIKYVIDNFCSLWGIELCIFTEIIVIGARGDVYSAIYSVVLGLQLFLPRKQLSTLWPIY

IAFLTISIIAQYLLLLGLPTYLCYGDFFMLMFACCQMKAYLSGKRDSHLTVRIHAEDGKMTNPGNFLNNR

CWLDTIQIFIFQYFLWITYAVIFIVGTSQINIFCFGFLLADFFFLWHGQSIVKRLKFDDNGKMVKTWWKL

FIAYTYTVILLKTSFQVVVCAWPNIIPCIVSRIFNVVCLRAFVGEDPVERMNCVTLPKKYGVGIDGVCLM

FLLIQQLIFNSNQIYYVAEELFETNQLSKKVAKFLRDCRQERIKLEIESREKIRKRIKDSVEVLKSRRNS

KLFANLSHEQIIAAGDYYLFRDLYQKEKLVGQTVDTQYTDTVSDDRSTQDKSQIDDLDPSSKEKIQNKQQ

IANEHHEDVQSSEAASNDLVYLKQDTDFECHDVSQRAEPGITDNPLYGRHSSIYVSKSQTGGPASTQYRI

ADGESSSSTLQEHTKHSHNQFIESASNAASTDTKRAFKTQENISDLPAPQNNLFLNKQLNVQEVTLKDIT

TDSLPSRLDSLDGKVTSKEEKKKGAFRRFINYLYCNIWITYTDKLTRWLDTFGIEYRAAAKKIRQERANL

SKVHSNERAVTTNDVHDSAENTVVAAAVITANADLDLDEPLESKELQRLQSSLAYKTESNDGWQGNMSRF

DHFIFALYYSLCANTDNICYFLILLNLIVHGTVLSLILPLSTFLWAFLSIPRPHRYFWIFAFTYTEIVVL

IKYTFQFSFWTFNRPDGNNDPFWAPRIIGIVQEDNFGRIVAFDLILLLSLFFHCAILKSFGLWNDDDSNN

DHDEMTESEEIWPNIKFIQQLLRHDSKPGTIDVYTSMFACTFIAFLIIIFGWSSFGENASQNDNLSTFIA

ENNIPLPFLIAMLTQFLLMLCDRAIYLRRSLLARLIVYIIQVIALHTWLFFFLPFLTRRSFKYNAAAIIL

YITYCIYFRLSCFQLCQGYPIRVLRRFLTDIFNNYTYYFVLCVNAIPFFLEFRTLMDWTCTDTTLTLWHW

LKMEDIFYNIFYTKCRRDYENSYPQKKGDPKSPLFKVALGGFLIFLLGAIIWFPLLFMSLAKTSGIADLP

TEGQFSLSIVGYEPLYSYTAEQRSITMLSQSDYNHLFNVHKYNNPSARSFLSQYDRENVVRVLLNENSSP

IWEISPPVRQKLINDLSSNQTITLRFSWSFNRKPNSALVSDMVTGNNYFNLYDPNLRDGLSQMLMFNDNQ

TSMVTIPKLYPSFVLVPATGASTVVKALDNGSYVDCGVELLKGPTYDDVLKSMVEWWRIVQKSPFHINNT

KSSRSNSLEMIVFSDKVIPPGLSFLAGYGIIGLYVSLVLVVGRFIRIFVSSVSYRIMFDEIPDPNAIHEL

CEEIYMVRESHEFELEEELFAKLIFLYRSPETIIRLTRLASLLIHYACIDPTDTKIISYKSGCIALNGNN

L

>Amphimedon queenslandica PREDICTED: piezo-type mechanosensitive ion channel component 2-like XP_003384616.2

MIWCQEGKYRGYPLLLSVPVPIWFQGDSFLILEDSYLKDLWIKDYSYFIPVLPEMGGESFVPPRYGATGN

TPVNETSLTVGDGHSIASIESRLNSIKTLWDKFVIRVKELAHVLMSVNYVVSLAAMMAWGVIYPTWVALV

LLLWACGMCLIPKIDLKRSFYLISPLLLLYSVALVLLQYVNSLNLSLSVNVAVVGECDTQSTPWALQNCR

ALVLTVKSLLMLPFCASVYQFISTWNLVKENSEEAPPAAAPNGDKEEQISTVALLQSQLTVILITYWILV

VVGAFLLVILAGDASILKSVYVVFFFILLLFYQIFPKWWIYIMYPFWCLLVAYTTLHLILVYTFPFPSIN

DLWMSAYNSSNHGWNFTAEEFYDALGLLQYTTSGNTSRSLFYVLVPLAILIIVLALQIRYFTPWATFCSP

VAQEELDGSTDVSSRNAEMLFHRMTNLVSTREATDTKREREVEEKEEEDEEEIHVEEENVEDKDHSDFSP

ELTLYKVANYVLFQLRRGIVAIWYLSWRFLELHIHKAIVLSLFGLCLYEVSASYLILVGLVLLVAALPII

NGLAYPAATLYLGLLSLGKFLFQISLIESFNIDVDEDCVDPGLSSRSLLFNYTYSPTSNDSSWIGLNKAA

SDTEYVAGPLLVSLLIALSYSTRRHQAYSIENKRLTIERWEIGSLFLGVKQELAHHSWTDAIKYILNHFF

EMYGVESIVIAVRIDIYGVFAAILLGAMLITPRRFLRIFWMLYLTIQGILILVQYLALLGAPTSAICDNG

YPWASKFNDTYHTSYLCMALQMKYFVRNPLTGLPAVTDYPFMYDTDKFLDYLKILLFQYYFWLAIFFVFV

AGAFGLNVFGLIFLLLCFIFLYRGQLLLSDERTKRKKWWMTLRAFIYFILLIRIMIELPGCVFIPEISFG

GRCGFISVINLHCSEPLYYGIPSSLDCDNNPHSSTGLWFDVLTFILVTIQVILLDTQYADQVAREINEKE

SSAVEFSKILFAVLDENEKKSTEKQKEELEELKERAGKLKLTEGKIAHLLNVGTRVPPDFSLTVSPTHSS

KKDSSPTDIEEKEEEEEEEVKHKSKAEDVAEEISKWKKVGSFLWKVWLEVVDYIINFLEDSSANYMEVLK

RVAKARHESHAPAADSPHSEEGRGLEETGTKPPGIAVQAEVTESGATPPVSETKFTHSSSVRKSPSFPPP

SMKRLRSSGYNFDKSGDIIQTLRIAPSSDHEEQAKAVHERLEKLSTEYTRRPKRLLRALYYWTLSHFNYV

VIFMAILAIIRSGSFISFFYAAIIFMWGLLYVPWPTKRYWNILLFFTMFVLVVKYIYYFVYFAIGNDGET

GDNAFLGLNGPASWFFGIETTSSYFSNSFVHLLLLMSIIFHRGLLKQHGLWQNISTLSKRTGILRSVFYR

FIGFFKTLFSKTTIAGAKDLYNWMLFFDCLCFITIALGYSSFSVSTGSGDVPEVATYIQSNNIPLTFLTM

LFLQFFFMLVDRAIYLRHSIIAKFVFYVTLLILWHIWMFLILPVTTEKAFIDNPAAQAIYFFKSMYFIVS

ALQMISGYPERLLKNFAAKNFSPPVGFIFLGYRAIPIIPELREVMDWVFTDTSLTVINWLRVQEIWAQLY

QIKVRRVREKLLPRDLGDKQSFVVKLFLGGAFLVGLVLLIWGPLLVISLVNETSTPNPPIYATVDLDLEG

YQLLQIQAQSSFIYELNQDQYSTLRNKITTTTIRRDFNDLFPKENFRRILFPGGSSLLWTASQPSQDQLK

DLLALSAKAVHKLVFSWSFRRSPGTGVASEVISGSKNIEINTEVARQFLDNLNGNLSLINLTSLYPLYVS

APAAEMSTEYTSISNYISGNTVVDCSALLNRTETLNNVTINQWWTLTQSSSYVLATGNESPGSLEMIVHS

DPVVPVVFSFVSNLGIIGLYVSVVLVISRFLRSFINSLMFEIIIDEILYPDPLLELCDDIFAVREAKDFV

LEEVLVGKLFYIFRSPERLLKITELPKTKQD

**TRP Channels (Figure 9):**

>XP_006511116.1 short transient receptor potential channel 1 isoform X1 [Mus musculus]

MGAPPPSPGLPPSWAAMMAALYPSTDLSGVSSSSLPSSPSSSSPNEVMALKDVREVKEENTLNEKLFLLA

CDKGDYYMVKKILEENSSGDLNINCVDVLGRNAVTITIENESLDILQLLLDYGCQSADALLVAIDSEVVG

AVDILLNHRPKRSSRPTIVKLMERIQNPEYSTTMDVAPVILAAHRNNYEILTMLLKQDVSLPKPHAVGCE

CTLCSAKNKKDSLRHSRCLASPALIMLTEEDPILRAFELSADLKELSLVEVEFRNDYEELARQCKMFAKD

LLAQARNSRELEVILNHTSSDEPLDKRGLLEERMNLSRLKLAIKYNQKEFVSQSNCQQFLNTVWFGQMSG

YRRKPTCKKIMTVLTVGIFWPVLSLCYLIAPKSQFGRIIHTPFMKFIIHGASYFTFLLLLNLYSLVYNED

KKNTMGPALERIDYLLILWIIGMIWSDIKRLWYEGLEDFLEESRNQLSFVMNSLYLATFALKVVAHNKFH

DFADRKDWDAFHPTLVAEGLFAFANVLSYLRLFFMYTTSSILGPLQISMGQMLQDFGKFLGMFLLVLFSF

TIGLTQLYDKGYTSKEQKDCVGIFCEQQSNDTFHSFIGTCFALFWYIFSLAHVAIFVTRFSYGEELQSFV

GAVIVGTYNVVVVIVLTKLLVAMLHKSFQLIANHEDKEWKFARAKLWLSYFDDKCTLPPPFNIIPSPKTI

CYMISSLSKWICSHTSKGKVKRQNSLKEWRNLKQKRDENYQKVMCCLVHRYLTSMRQKMQSTDQATVENL

NELRQDLSKFRNEIRDLLGFRTSKYAMFYPRN

>NP_001103367.1 short transient receptor potential channel 2 [Mus musculus]

MDPLSPQPNWTEIVNKKLKFPPTLLRAIQEGQLGLVQQLLESSSDASGAGPGGPLRNVEESEDRSWREAL

NLAIRLGHEVITDVLLANVKFDFRQIHEALLVAVDTNQPAVVRRLLARLEREKGRKVDTKSFSLAFFDSS

IDGSRFAPGVTPLTLACQKDLYEIAQLLMDQGHTIARPHPVSCACLECSNARRYDLLKFSLSRINTYRGI

ASRAHLSLASEDAMLAAFQLSRELRRLARKEPEFKPQYIALESLCQDYGFELLGMCRNQSEVTAVLNDLG

EDSETEPEAEGLGQAFEEGIPNLARLRLAVNYNQKQFVAHPICQQVLSSIWCGNLAGWRGSTTIWKLFVA

FLIFLTMPFLCIGYWLAPKSQLGRLLKIPVLKFLLHSASYLWFLIFLLGESLVMETQLSTFKGRSQSVWE

TSLHMIWVTGFLWFECKEVWIEGLRSYLLDWWNFLDVVILSLYLASFALRLLLAGLAYMHCRDASDSTTC

RYFTTAERSEWRTEDPQFLAEVLFAVTSMLSFTRLAYILPAHESLGTLQISIGKMIDDMIRFMFILMIIL

TAFLCGLNNIYVPYQESEKLGNFNETFQFLFWTMFGMEEHTVVDMPQFLVPEFVGRAMYGIFTIVMVIVL

LNMLIAMITNSFQKIEDDADVEWKFARSKLYLSYFREGLTLPVPFNILPSPKAAFYLVRRIFRFLCCGSS

CCKAKKSDYPPIGTFTNPGARAGSAGEGERVSYRLRVIKALVQRYIETARREFEETRRKDLGNRLTELTK

TVSRLQSEVASVQKNLAAGGAPRPPDGASILSRYITRVRNSFQNLGPPTSDTPAELTMPGIVETEVSLGD

GLDGTGEAGAPAPGEPGSSSSAHVLVHREQEAEGSGDLLLEGDLETKGES

>NP_062383.2 short transient receptor potential channel 3 [Mus musculus]

MSTKVKKCREPARVTLPAPEEEEDGEAEGGESQRRRRGWRGVNGGLEPPCPRAPPSPGPDASSEGSPSRW

RTAGMRDKGRRQAVRGPAFMFGARGPSLTAEEERFLDAAEYGNIPVVRKMLEESRTLNVNCVDYMGQNAL

QLAVGNEHLEVTELLLKKENLARIGDALLLAISKGYVRIVEAILGHPGFAASRRLTLSPCEQELRDDDFY

AYDEDGTRFSPDITPIILAAHCHKYEVVHLLLLKGARIERPHDYFCRCSDCAEKQRLDAFSHSRSRINAY

KGLASPAYLSLSSEDPVLTALELSNELAKLANIEKEFKNDYRKLSMQCKDFVVGVLDLCRDSEEVEAILN

GDLESAEPLERHGHKASLSRVKLAIKYEVKKFVAHPNCQQQLLTIWYENLSGLREQTIAIKCLVVLVVAL

GLPFLAIGYWIAPCSRLGKILRSPFMKFVAHAASFIIFLGLLVFNASDRFEGITTLPNITVIDYPKQIFR

VKTTQFTWTEMLIMVWVLGMMWSECKELWLEGPREYIVQLWNVLDFGMLSIFIAAFTARFLAFLQATKAQ

QYVDSHVQESDLSEVTLPPEVQYFTYARDKWLPSDPQIISEGLYAIAVVLSFSRIAYILPANESFGPLQI

SLGRTVKDIFKFMVLFIMVFLAFMIGMFILYSYYLGAKVNPAFTTVEESFKTLFWSIFGLSEVTSVVLKY

DHKFIENIGYVLYGIYNVTMVVVLLNMLIAMINSSYQEIEDDSDVEWKFARSKLWLSYFDDGKTLPPPFS

LVPSPKSFVYFIMRITNFSKCRRRRLQKDLELGMGNSKSRLNLFTQSNSRVFESHSFNSILNQPTRYQQI

MKRLIKRYVLKAQVDKENDEVNEGELKEIKQDISSLRYELLEDKSQATEELAILIHKLSEKLNPSVLRCE

>XP_006501359.1 short transient receptor potential channel 4 isoform X1 [Mus musculus]

MAQFYYKRNVNAPYRDRIPLRIVRAESELSPSEKAYLNAVEKGDYASVKKSLEEAEIYFKININCIDPLG

RTALLIAIENENLELIELLLSFNVYVGDALLHAIRKEVVGAVELLLNHKKPSGEKQVPPILLDKQFSEFT

PDITPIILAAHTNNYEIIKLLVQKGVSVPRPHEVRCNCVECVSSSDVDSLRHSRSRLNIYKALASPSLIA

LSSEDPFLTAFQLSWELQELSKVENEFKSEYEELSRQCKQFAKDLLDQTRSSRELEIILNYRDDNSLIEE

QSGNDLARLKLAIKYRQKEFVAQPNCQQLLASRWYDEFPGWRRRHWAVKMVTCFIIGLLFPVFSVCYLIA

PKSPLGLFIRKPFIKFICHTASYLTFLFLLLLASQHIDRSDLNRQGPPPTIVEWMILPWVLGFIWGEIKQ

MWDGGLQDYIHDWWNLMDFVMNSLYLATISLKIVAFVKYSALNPRESWDMWHPTLVAEALFAIANIFSSL

RLISLFTANSHLGPLQISLGRMLLDILKFLFIYCLVLLAFANGLNQLYFYYEETKGLSCKGIRCEKQNNA

FSTLFETLQSLFWSIFGLINLYVTNVKAQHEFTEFVGATMFGTYNVISLVVLLNMLIAMMNNSYQLIADH

ADIEWKFARTKLWMSYFEEGGTLPTPFNVIPSPKSLWYLVKWIWTHLCKKKMRRKPESFGTIGRRAADNL

RRHHQYQEVMRNLVKRYVAAMIREAKTEEGLTEENVKELKQDISSFRFEVLGLLRGSKLSTIQSANAASS

ADSDEKSQSEGNGKDKRKNLSLFDLTTLIHPRSAAIASERHNLSNGSALVVQEPPREKQRKVNFVADIKN

FGLFHRRSKQNAAEQNANQIFSVSEEITRQQAAGALERNIELESKGLASRGDRSIPGLNEQCVLVDHRER

NTDTLGLQVGKRVCSTFKSEKVVVEDTVPIIPKEKHAHEEDSSIDYDLSPTDTAAHEDYVTTRL

>NP_033454.1 short transient receptor potential channel 5 [Mus musculus]

MAQLYYKKVNYSPYRDRIPLQIVRAETELSAEEKAFLSAVEKGDYATVKQALQEAEIYYNVNINCMDPLG

RSALLIAIENENLEIMELLLNHSVYVGDALLYAIRKEVVGAVELLLSYRKPSGEKQVPTLMMDTQFSEFT

PDITPIMLAAHTNNYEIIKLLVQKRVTIPRPHQIRCNCVECVSSSEVDSLRHSRSRLNIYKALASPSLIA

LSSEDPILTAFRLGWELKELSKVENEFKAEYEELSQQCKLFAKDLLDQARSSRELEIILNHRDDHSEELD

PQKYHDLAKLKVAIKYHQKEFVAQPNCQQLLATLWYDGFPGWRRKHWVVKLLTCMTIGFLFPMLSIAYLI

SPRSNLGLFIKKPFIKFICHTASYLTFLFMLLLASQHIVRTDLHVQGPPPTVVEWMILPWVLGFIWGEIK

EMWDGGFTEYIHDWWNLMDFAMNSLYLATISLKIVAYVKYNGSRPREEWEMWHPTLIAEALFAISNILSS

LRLISLFTANSHLGPLQISLGRMLLDILKFLFIYCLVLLAFANGLNQLYFYYETRAIDEPNNCKGIRCEK

QNNAFSTLFETLQSLFWSVFGLLNLYVTNVKARHEFTEFVGATMFGTYNVISLVVLLNMLIAMMNNSYQL

IADHADIEWKFARTKLWMSYFDEGGTLPPPFNIIPSPKSFLYLGNWFNNTFCPKRDPDGRRRRHNLRSFT

ERHADSLIQNQHYQEVIRNLVKRYVAAMIRNSKTNEGLTEENFKELKQDISSFRYEVLDLLGNRKHPRRS

LSTSSADFSQRDDTNDGSGGARAKSKSVSFNVGCKKKACHGAPLIRTVPRASGAQGKPKSESSSKRSFMG

PSFKKLGLFFSKFNGQTSEPTSEPMYTISDGIAQQHCMWQDIRYSQMEKGKAEACSQSQMNLGEVELGEI

RGAAARSSECPLACSSSLHCASGICSSNSKLLDSSEDVFETWGEACDLLMHKWGDGQEEQVTTRL

>NP_038866.2 short transient receptor potential channel 6 isoform 1 [Mus musculus]

MSQSPRFVTRRGGSLKAAPGAGTRRNESQDYLLMDELGDDGYPQLPLPPYGYYPSFRGNENRLTHRRQTI

LREKGRRLANRGPAYMFNDHSTSLSIEEERFLDAAEYGNIPVVRKMLEECHSLNVNCVDYMGQNALQLAV

ANEHLEITELLLKKENLSRVGDALLLAISKGYVRIVEAILNHPAFAEGKRLATSPSQSELQQDDFYAYDE

DGTRFSHDVTPIILAAHCQEYEIVHTLLRKGARIERPHDYFCKCTECSQKQKHDSFSHSRSRINAYKGLA

SPAYLSLSSEDPVMTALELSNELAVLANIEKEFKNDYRKLSMQCKDFVVGLLDLCRNTEEVEAILNGDAE

TRQPGDFGRPNLSRLKLAIKYEVKKFVAHPNCQQQLLSIWYENLSGLRQQTMAVKFLVVLAVAIGLPFLA

LIYWCAPCSKMGKILRGPFMKFVAHAASFTIFLGLLVMNAADRFEGTKLLPNETSTDNARQLFRMKTSCF

SWMEMLIISWVIGMIWAECKEIWTQGPKEYLFELWNMLDFGMLAIFAASFIARFMAFWHASKAQSIIDAN

DTLKDLTKVTLGDNVKYYNLARIKWDPTDPQIISEGLYAIAVVLSFSRIAYILPANESFGPLQISLGRTV

KDIFKFMVIFIMVFVAFMIGMFNLYSYYIGAKQNEAFTTVEESFKTLFWAIFGLSEVKSVVINYNHKFIE

NIGYVLYGVYNVTMVIVLLNMLIAMINSSFQEIEDDADVEWKFARAKLWFSYFEEGRTLPVPFNLVPSPK

SLLYLLLKFKKWMCELIQGQKQGFQEDAEMNKRNEEKKFGISGSHEDLSKFSLDKNQLAHNKQSSTRSSE

DYHLNSFSNPPRQYQKIMKRLIKRYVLQAQIDKESDEVNEGELKEIKQDISSLRYELLEEKSQNTEDLAE

LIRKLGERLSLEPKLEESRR

>XP_006517325.1 short transient receptor potential channel 7 isoform X1 [Mus musculus]

MPVSHTDASPSHSLGNFPQIRKRTGRQVGHKRRVFTIPHKGKQSAPPHNRNSALWSRSRLQHHGERHLRL

GHPWGARSRRLRSGFGSSRGGTSLRILLSMLGSNTFKNMQRRHTTLREKGRRQAIRGPAYMFNEKGTSLT

PEEERFLDSAEYGNIPVVRKMLEESKTLNFNCVDYMGQNALQLAVGNEHLEVTELLLKKENLARVGDALL

LAISKGYVRIVEAILSHPAFAQGQRLTLSPLEQELRDDDFYAYDEDGTRFSHDITPIILAAHCQEYEIVH

ILLLKGARIERPHDYFCKCNECTEKQRKDSFSHSRSRMNAYKGLASAAYLSLSSEDPVLTALELSNELAR

LANIETEFKNDYRKLSMQCKDFVVGVLDLCRDTEEVEAILNGDVNLQVWSDHHRPSLSRIKLAIKYEVKK

LGQTLRSPFMKFVAHAVSFTIFLGLLVVNASDRFEGVKTLPNETFTDYPKQIFRVKTTQFSWTEMLIMKW

VLGMIWSECKEIWEEGPREYVLHLWNLLDFGMLSIFVASFTARFMAFLKASEAQLYVDQYVQDVTLHNVS

LPPEVAYFTYARDKWWPSDPQIISEGLYAIAVVLSFSRIAYILPANESFGPLQISLGRTVKDIFKFMVIF

IMVFVAFMIGMFNLYSYYRGAKYNPAFTTVEESFKTLFWSIFGLSEVISVVLKYDHKFIENIGYVLYGVY

NVTMVVVLLNMLIAMINNSYQEIEEDADVEWKFARAKLWLSYFDEGRTLPAPFNLVPSPKSFYYLIMRIK

MCLIELCQSKAKRCENDLEMGMLNSKFRKTRYQAGMRNSENLTANSTFSKPTRYQKIMKRLIKRYVLKAQ

VDRENDEVNEGELKEIKQDISSLRYELLEEKSQATGELADLIQQLSEKFGKNLNKDHLRVNQGKDI

>NP_808449.1 transient receptor potential cation channel subfamily A member 1 isoform 1 [Mus musculus]

MKRGLRRILLPEERKEVQGVVYRGVGEDMDCSKESFKVDIEGDMCRLEDFIKNRRKLSKYEDENLCPLHH

AAAEGQVELMELIINGSSCEVLNIMDGYGNTPLHCAAEKNQVESVKFLLSQGANPNLRNRNMMSPLHIAV

HGMYNEVIKVLTEHKATNINLEGENGNTALMSTCAKDNSEALQILLEKGAKLCKSNKWGDYPVHQAAFSG

AKKCMELILAYGEKNGYSRETHINFVNHKKASPLHLAVQSGDLDMIKMCLDNGAHIDMMENAKCMALHFA

ATQGATDIVKLMISSYTGSSDIVNAVDGNQETLLHRASLFDHHDLAEYLISVGADINSTDSEGRSPLILA

TASASWNIVNLLLCKGAKVDIKDHLGRNFLHLTVQQPYGLRNLRPEFMQMQHIKELVMDEDNDGCTPLHY

ACRQGVPVSVNNLLGFNVSIHSKSKDKKSPLHFAASYGRINTCQRLLQDISDTRLLNEGDLHGMTPLHLA

AKNGHDKVVQLLLKKGALFLSDHNGWTALHHASMGGYTQTMKVILDTNLKCTDRLDEEGNTALHFAAREG

HAKAVAMLLSYNADILLNKKQASFLHIALHNKRKEVVLTTIRNKRWDECLQVFTHNSPSNRCPIMEMVEY

LPECMKVLLDFCMIPSTEDKSCQDYHIEYNFKYLQCPLSMTKKVAPTQDVVYEPLTILNVMVQHNRIELL

NHPVCREYLLMKWCAYGFRAHMMNLGSYCLGLIPMTLLVVKIQPGMAFNSTGIINGTSSTHEERIDTLNS

FPIKICMILVFLSSIFGYCKEVIQIFQQKRNYFLDYNNALEWVIYTTSIIFVLPLFLNIPAYMQWQCGAI

AIFFYWMNFLLYLQRFENCGIFIVMLEVIFKTLLRSTGVFIFLLLAFGLSFYVLLNFQDAFSTPLLSLIQ

TFSMMLGDINYRDAFLEPLFRNELAYPVLTFGQLIAFTMFVPIVLMNLLIGLAVGDIAEVQKHASLKRIA

MQVELHTNLEKKLPLWYLRKVDQRSTIVYPNRPRHGRMLRFFHYFLNMQETRQEVPNIDTCLEMEILKQK

YRLKDLTSLLEKQHELIKLIIQKMEIISETEDEDNHCSFQDRFKKERLEQMHSKWNFVLNAVKTKTHCSI

SHPDF

>XP_017177501.1 transient receptor potential cation channel subfamily M member 1 isoform X1 [Mus musculus]

MVWAVLMKRQKMAVFLWQRGEECMAKALVACKLYKAMAHESSESELVDDISQDLDNNSKDFGQLAVELLD

QSYKHDEQVAMKLLTYELKNWSNSTCLKLAVAAKHRDFIAHTCSQMLLTDMWMGRLRMRKNPGLKVIMGI

LIPPTILFLEFRTYDDFSYQTSKENEDGKEKEEENVISYLGYLLLFNYVILVRMDGWPSPQEWIVISYIV

SLALEKIREILMSEPGKLSQKIKVWLQEYWNITDLVAISMFMVGAILRLQSQPYMGYGRVIYCVDIILWY

IRVLDIFGVNKYLGPYVMMIGKMMIDMLYFVVIMLVVLMSFGVARQAILHPEEKPSWKLARNIFYMPYWM

IYGEVFADQIDLYAMEINPPCGENLYDEEGKRLPPCIPGAWLTPALMACYLLVANILLVNLLIAVFNNTF

FEVKSISNQVWKFQRYQLIMTFHDRPVLPPPMIILSHIYIIIMRLSGRCRKKREGDQEERDRGLKLFLSD

EELKKLHEFEEQCVQEHFREKEDEQQSSSDERIRVTSERVENMSMRLEEINERENFMKTSLQTVDLRLSQ

LEELSGRMVSALENLAGIDRSDLIQARSRASSECEATYLLRQSSINSADGYSLYRYHFNGEELLFEEPAL

STSPGTAFRKKTYSFRVKDEDAKSHLDQPSNLHHTPGPSPPATPGRSRLALEGPLSTELRPGSDPGISAG

EFDPRADFKSTEAAPSLNAAGVTGTQLTVESTDSHPLRESKLVRYYPGDPNTYKTMKSRSFVYTEGRKLV

RGLSNWSAEYSSIMDQAWNATEWRCQVQRITRSRSTDIPYIVSEAASQDELEDEHRGSLLDPQISRSALT

VSDRPEKENLLSVKPHQTLGFPCLRSRSLHGRPRSAEPAPSKLDRAGHASSTSNLAVMSVVPEGQNTQQE

KRSAETEC

>XP_006513793.1 transient receptor potential cation channel subfamily M member 2 isoform X1 [Mus musculus]

MESLDRRRTGSEQEEGFGVQSRRATDLGMVPNLRRSNSSLCKSRRFLCSFSSEKQENLSSWIPENIKKKE

CVYFVESSKLSDAGKVVCACGYTHEQHLEVAIKPHTFQGKEWDPKKHVQEMPTDAFGDIVFTDLSQKVGK

YVRVSQDTPSSVIYQLMTQHWGLDVPNLLISVTGGAKNFNMKLRLKSIFRRGLVKVAQTTGAWIITGGSH

TGVMKQVGEAVRDFSLSSSCKEGEVITIGVATWGTIHNREGLIHPMGGFPAEYMLDEEGQGNLTCLDSNH

SHFILVDDGTHGQYGVEIPLRTKLEKFISEQTKERGGVAIKIPIVCVVLEGGPGTLHTIYNAINNGTPCV

IVEGSGRVADVIAQVATLPVSEITISLIQQKLSIFFQEMFETFTENQIVEWTKKIQDIVRRRQLLTIFRE

GKDGQQDVDVAILQALLKASRSQDHFGHENWDHQLKLAVAWNRVDIARSEIFTDEWQWKPADLHPMMTAA

LISNKPEFVRLFLENGVRLKEFVTWDTLLCLYENLEPSCLFHSKLQKVLAEEQRLAYASATPRLHMHHVA

QVLRELLGDSTQLLYPRPRYTDRPRLSMTVPHIKLNVQGVSLRSLYKRSTGHVTFTIDPVRDLLIWAVIQ

NHRELAGIIWAQSQDCTAAALACSKILKELSKEEEDTDSSEEMLALADEFEHRAIGVFTECYRKDEERAQ

KLLVRVSEAWGKTTCLQLALEAKDMKFVSHGGIQAFLTKVWWGQLCVDNGLWRIILCMLAFPLLFTGFIS

FREKRLQALCRPARVRAFFNAPVVIFHMNILSYFAFLCLFAYVLMVDFQPSPSWCEYLIYLWLFSLVCEE

TRQLFYDPDGCGLMKMASLYFSDFWNKLDVGAILLFIVGLTCRLIPATLYPGRIILSLDFIMFCLRLMHI

FTISKTLGPKIIIVKRMMKDVFFFLFLLAVWVVSFGVAKQAILIHNESRVDWIFRGVVYHSYLTIFGQIP

TYIDGVNFSMDQCSPNGTDPYKPKCPESDWTGQAPAFPEWLTVTLLCLYLLFANILLLNLLIAMFKLDCC

VPQTF

>NP_001030316.1 transient receptor potential cation channel subfamily M member 3 isoform a [Mus musculus]

MGKKWRDAGELERGCSDREDSAESRRRSRSASRGRFAESWKRLSSKQGSTKRSGLPAQQTPAQKSWIERA

FYKRECVHIIPSTKDPHRCCCGRLIGQHVGLTPSISVLQNEKNESRLSRNDIQSEKWSISKHTQLSPTDA

FGTIEFQGGGHSNKAMYVRVSFDTKPDLLLHLMTKEWQLELPKLLISVHGGLQNFELQPKLKQVFGKGLI

KAAMTTGAWIFTGGVNTGVIRHVGDALKDHASKSRGKICTIGIAPWGIVENQEDLIGRDVVRPYQTMSNP

MSKLTVLNSMHSHFILADNGTTGKYGAEVKLRRQLEKHISLQKINTRIGQGVPVVALIVEGGPNVISIVL

EYLRDTPPVPVVVCDGSGRASDILAFGHKYSEEGGLINESLRDQLLVTIQKTFTYTRTQAQHLFIILMEC

MKKKELITVFRMGSEGHQDIDLAILTALLKGANASAPDQLSLALAWNRVDIARSQIFIYGQQWPVGSLEQ

AMLDALVLDRVDFVKLLIENGVSMHRFLTISRLEELYNTRHGPSNTLYHLVRDVKKGNLPPDYRISLIDI

GLVIEYLMGGAYRCNYTRKRFRTLYHNLFGPKRPKALKLLGMEDDIPLRRGRKTTKKREEEVDIDLDDPE

INHFPFPFHELMVWAVLMKRQKMALFFWQHGEEAMAKALVACKLCKAMAHEASENDMVDDISQELNHNSR

DFGQLAVELLDQSYKQDEQLAMKLLTYELKNWSNATCLQLAVAAKHRDFIAHTCSQMLLTDMWMGRLRMR

KNSGLKVILGILLPPSILSLEFKNKDDMPYMTQAQEIHLQEKEPEEPEKPTKEKDEEDMELTAMLGRSNG

ESSRKKDEEEVQSRHRLIPVGRKIYEFYNAPIVKFWFYTLAYIGYLMLFNYIVLVKMERWPSTQEWIVIS

YIFTLGIEKMREILMSEPGKLLQKVKVWLQEYWNVTDLIAILLFSVGMILRLQDQPFRSDGRVIYCVNII

YWYIRLLDIFGVNKYLGPYVMMIGKMMIDMMYFVIIMLVVLMSFGVARQAILFPNEEPSWKLAKNIFYMP

YWMIYGEVFADQIDRKQVYDSHTPKSAPCGQNETREDGKTIQLPPCKTGAWIVPAIMACYLLVANILLVN

LLIAVFNNTFFEVKSISNQVWKFQRYQLIMTFHERPVLPPPLIIFSHMTMIFQHVCCRWRKHESDQDERD

YGLKLFITDDELKKVHDFEEQCIEEYFREKDDRFNSSNDERIRVTSERVENMSMRLEEVNEREHSMKASL

QTVDIRLAQLEDLIGRMATALERLTGLERAESNKIRSRTSSDCTDAAYIVRQSSFNSQEGNTFKLQESID

PAGEETISPTSPTLMPRMRSHSFYSVNVKDKGGIEKLESIFKERSLSLHRATSSHSVAKEPKAPAAPANT

LAIVPDSRRPSSCIDIYVSAMDELHCDIEPLDNSMNILGLGEPSFSALAPSTTPSSSAYATLAPTDRPPS

RSIDFEDLTSMDTRSFSSDYTHLPECQNPWDTDPPTYHTIERSKSSRYLATTPFLLEEAPIVKSHSFMFS

PSRSYYANFGVPVKTAEYTSITDCIDTRCVNAPQAIADRATFPGGLGDKVEDLSCCHPEREAELSHPSSD

SEENEARGQRAANPISSQEAENADRTLSNNITVPKIERANSYSAEEPNVPYAHTRKSFSISDKLDRQRNT

ASLRNPFQRSKSSKPEGRGDSLSMRRLSRTSAFHSFESKHN

>NP_780339.2 transient receptor potential cation channel subfamily M member 4 [Mus musculus]

MVGPEKEQSWIPKIFRKKVCTTFIVDLSDDAGGTLCQCGQPRDAHPSVAVEDAFGAAVVTEWNSDEHTTE

KPTDAYGDLDFTYSGRKHSNFLRLSDRTDPATVYSLVTRSWGFRAPNLVVSVLGGSGGPVLQTWLQDLLR

RGLVRAAQSTGAWIVTGGLHTGIGRHVGVAVRDHQTASTGSSKVVAMGVAPWGVVRNRDMLINPKGSFPA

RYRWRGDPEDGVEFPLDYNYSAFFLVDDGTYGRLGGENRFRLRFESYVAQQKTGVGGTGIDIPVLLLLID

GDEKMLKRIEDATQAQLPCLLVAGSGGAADCLVETLEDTLAPGSGGLRRGEARDRIRRYFPKGDPEVLQA

QVERIMTRKELLTVYSSEDGSEEFETIVLRALVKACGSSEASAYLDELRLAVAWNRVDIAQSELFRGDIQ

WRSFHLEASLMDALLNDRPEFVRLLISHGLSLGHFLTPVRLAQLYSAVSPNSLIRNLLDQASHASSSKSP

PVNGTVELRPPNVGQVLRTLLGETCAPRYPARNTRDSYLGQDHRENDSLLMDWANKQPSTDASFEQAPWS

DLLIWALLLNRAQMAIYFWEKGSNSVASALGACLLLRVMARLESEAEEAARRKDLAATFESMSVDLFGEC

YHNSEERAARLLLRRCPLWGEATCLQLAMQADARAFFAQDGVQSLLTQKWWGEMDSTTPIWALLLAFFCP

PLIYTNLIVFRKSEEEPTQKDLDFDMDSSINGAGPPGTVEPSAKVALERRQRRRPGRALCCGKFSKRWSD

FWGAPVTAFLGNVVSYLLFLLLFAHVLLVDFQPTKPSVSELLLYFWAFTLLCEELRQGLGGGWGSLASGG

RGPDRAPLRHRLHLYLSDTWNQCDLLALTCFLLGVGCRLTPGLFDLGRTVLCLDFMIFTLRLLHIFTVNK

QLGPKIVIVSKMMKDVFFFLFFLCVWLVAYGVATEGILRPQDRSLPSILRRVFYRPYLQIFGQIPQEEMD

VALMIPGNCSMERGSWAHPEGPVAGSCVSQYANWLVVLLLIVFLLVANILLLNLLIAMFSYTFSKVHGNS

DLYWKAQRYSLIREFHSRPALAPPLIIISHVRLLIKWLRRCRRCRRANLPASPVFEHFRVCLSKEAERKL

LTWESVHKENFLLAQARDKRDSDSERLKRTSQKVDTALKQLGQIREYDRRLRGLEREVQHCSRVLTWMAE

ALSHSALLPPGAPPPPSPTGSKD

>NP_064673.2 transient receptor potential cation channel subfamily M member 5 [Mus musculus]

MQTTQSSCPGSPPDTEDGWEPILCRGEINFGGSGKKRGKFVKVPSSVAPSVLFELLLTEWHLPAPNLVVS

LVGEERPLAMKSWLRDVLRKGLVKAAQSTGAWILTSALHVGLARHVGQAVRDHSLASTSTKIRVVAIGMA

SLDRILHRQLLDGVHQKEDTPIHYPADEGNIQGPLCPLDSNLSHFILVESGALGSGNDGLTELQLSLEKH

ISQQRTGYGGTSCIQIPVLCLLVNGDPNTLERISRAVEQAAPWLILAGSGGIADVLAALVSQPHLLVPQV

AEKQFREKFPSECFSWEAIVHWTELLQNIAAHPHLLTVYDFEQEGSEDLDTVILKALVKACKSHSQEAQD

YLDELKLAVAWDRVDIAKSEIFNGDVEWKSCDLEEVMTDALVSNKPDFVRLFVDSGADMAEFLTYGRLQQ

LYHSVSPKSLLFELLQRKHEEGRLTLAGLGAQQARELPIGLPAFSLHEVSRVLKDFLHDACRGFYQDGRR

MEERGPPKRPAGQKWLPDLSRKSEDPWRDLFLWAVLQNRYEMATYFWAMGREGVAAALAACKIIKEMSHL

EKEAEVARTMREAKYEQLALDLFSECYGNSEDRAFALLVRRNHSWSRTTCLHLATEADAKAFFAHDGVQA

FLTKIWWGDMATGTPILRLLGAFTCPALIYTNLISFSEDAPQRMDLEDLQEPDSLDMEKSFLCSRGGQLE

KLTEAPRAPGDLGPQAAFLLTRWRKFWGAPVTVFLGNVVMYFAFLFLFTYVLLVDFRPPPQGPSGSEVTL

YFWVFTLVLEEIRQGFFTDEDTHLVKKFTLYVEDNWNKCDMVAIFLFIVGVTCRMVPSVFEAGRTVLAID

FMVFTLRLIHIFAIHKQLGPKIIIVERMMKDVFFFLFFLSVWLVAYGVTTQALLHPHDGRLEWIFRRVLY

RPYLQIFGQIPLDEIDEARVNCSLHPLLLESSASCPNLYANWLVILLLVTFLLVTNVLLMNLLIAMFSYT

FQVVQGNADMFWKFQRYHLIVEYHGRPALAPPFILLSHLSLVLKQVFRKEAQHKRQHLERDLPDPLDQKI

ITWETVQKENFLSTMEKRRRDSEGEVLRKTAHRVDLIAKYIGGLREQEKRIKCLESQANYCMLLLSSMTD

TLAPGGTYSSSQNCGCRSQPASARDREYLESGLPPSDT

>NP_700466.1 transient receptor potential cation channel subfamily M member 6 [Mus musculus]

MQVKKSWIEGVFYKRECNKFIPSSKDPHRCTPGCQICHNLVRCYCGRLIEEHHGLDRAWNLSVTEGHGDE

QWSVEKHTVKSPTDTFGTINFQDGEHIHHSKYIRTSWDTKSDHLLHLMLKEWNMELPKLVISVHGGLQNF

KISSKLKETFSQGLVKAAETTGAWIITEGINSGVSKHVGDALKAHSSKSLRKIWTVGIPPWGVIENQREL

VGKDVVCMYQTLGNPLSKLTTLNCMHSHFILCDDGTVGMYGNEEKLRRNLEKHLSMQKIHTCSRQGVPVV

GLVMEGGPNVILWVWETVKNKEPVVVCEGTGRAADLLAFTYKHLEDGGILRPQVKEELFCLIQNMFNFSL

RQSKHLFQILMECMVHKDSITIFDADSEEHQDLDLAILTALLKGTSLSISEQLNLAMAWDRMDIAKKHIL

TYGQHWKPGALEQAMLDALVMDRVDFVKLLIENGVNLHRFLTIPRLEELYNTKQGPTNKFLRHLVQDVKQ

HTLLSSYRITLIDIGLVIEYLIGGAYRSSYTRKSFRILYNNLYRKHKSVSSFAQGLSQHSLHQRHSLRNR

KESSESTLHSQFFRTAQPYKSKEKPEDSQKSKKKSKERQSLSEEPEAAGFIYPYNDLLVWAVLMKRQNMA

MFFWQHGEEATVKAVIASILYRAMAREAKESNMVDDTSEELKNYSEQFGQLALDVLEKAFKQNEPMAMKL

LTYELKNWSNSTCLKLAVSGGLRPFVSHSCTQMLLTDMWMGRLKMRKNSWLKIIISILLPPMILTLEFKS

KAEMSHVPQSQDFQFTWNYSDQGLSNTKESACVKDYDLERGPDEKPDEPLHLDLRNVPQSLPWTRRVYEF

YSAPFVKFWFYTMAYLAFLMLFTYTVLVEMQPQPSVHEWLVIIYIFTNAIEKVREICISEPSKFKQKVKM

WLSEYWNLMETVAIGLFAVGFGLRWGHPPLQTAGRLIYCIDIIFWFSRLMDFFAVNQHAGPYVTMIAKMA

ANMFYIVIIMAIVLLSFGVARKAILSPKEPPSWRLARDIVFEPYWMMYGEVYASDIDVCSNETSCPPGSF

LTPFLQAVYLFVQYIIMVNLLIACFNNIYLDIKSISNKLWKYNRYRYIMTYHQKPWLPPPFILLNHLCLL

LRGLCCRPAPQDQEEGDGGLKLYLTKDDLKKLHDFEEQCVEKYFHEKTEGLNCSFEEQIRMTSERVSEMF

FQLKEMNEKVSFIKDSLLSLDSQVGHLQDLSAITVDTLKVLSAVDTLQEDEILLANRKHSTCRKRPHSWT

NVICAKVLSDMESCGKKKLQYYSMPPSLLRSLARSQLPPSVQRGALVEVTHSKREASHVREEQEEREMEQ

RTTASGISHVRQAHSKYGQFLLVPSSGKQVPLSLETPPHLFRSSEEAGIDGLVLEHIHQSDLTTHLPQQT

PAASHQALVAEHKDQHEAVTQMSDKPAKAEQDLLAFSGTPAPMTVTSLPSRAISMQDEGGYVNWAFSEND

ETGVFSFKKKWKTCLASTCNSDSNPGGDYFLHTGGRSGLDNSRRLAQSCECPAGPWTQARRSFWINPLCR

DKALIKSHSFRFHKEEKLRKTWKNNSHSKSLETRSTWLKAKLLTKTRSLSKKKRKTQGLQVPVITVNACY

QSDQLNAEPGETNTTEEFSKKWLSVSNFSQIGLEPYIYQKMKMKEIKRHTTQASDHLRQPQENRDKTPTW

NSGSTSLSRSFLTRSPNEVHKISTSLKSPQEPHHHYSAIERNNLMRLSQTIPFTPIQLFTGEEVTIYKLE

ESSPLTLDKSMSSWSQHGRAAMIQVLSQEEMDGGLRKAMRVISTWSEDDVLKPGQVFIVKSFLPEVVQTW

YKIFQESTVLHLCLREIQQQRAAQKLIYTFNQVKPQTIPYTPRFLEVSLVYCHSANQWLTIEKYMTGEFR

KYNNNNGDEIAPTNTLEELMLAFSHWTYEYTRGELLVLDLQGVGENLTDPSVIKPEDKQSRGMVFGPANL

GEDAIRSFIAKHRCNSCCGKLRLPDLKRNDYSLSRTHCNLGFGQTIEPTEELPERDKNRSSLEDHTRL

>NP_067425.2 transient receptor potential cation channel subfamily M member 7 isoform 1 [Mus musculus]

MSQKSWIESTLTKRECVYIIPSSKDPHRCLPGCQICQQLVRCFCGRLVKQHACFTASLAMKYSDVKLGEH

FNQAIEEWSVEKHTEQSPTDAYGVINFQGGSHSYRAKYVRLSYDTKPEIILQLLLKEWQMELPKLVISVH

GGMQKFELHPRIKQLLGKGLIKAAVTTGAWILTGGVNTGVAKHVGDALKEHASRSSRKICTIGIAPWGVI

ENRNDLVGRDVVAPYQTLLNPLSKLNVLNNLHSHFILVDDGTVGKYGAEVRLRRELEKTINQQRIHARIG

QGVPVVALIFEGGPNVILTVLEYLQESPPVPVVVCEGTGRAADLLAYIHKQTEEGGNLPDAAEPDIISTI

KKTFNFGQSEAVHLFQTMMECMKKKELITVFHIGSEDHQDIDVAILTALLKGTNASAFDQLILTLAWDRV

DIAKNHVFVYGQQWLVGSLEQAMLDALVMDRVSFVKLLIENGVSMHKFLTIPRLEELYNTKQGPTNPMLF

HLIRDVKQGNLPPGYKITLIDIGLVIEYLMGGTYRCTYTRKRFRLIYNSLGGNNRRSGRNTSSSTPQLRK

SHETFGNRADKKEKMRHNHFIKTAQPYRPKMDASMEEGKKKRTKDEIVDIDDPETKRFPYPLNELLIWAC

LMKRQVMARFLWQHGEESMAKALVACKIYRSMAYEAKQSDLVDDTSEELKQYSNDFGQLAVELLEQSFRQ

DETMAMKLLTYELKNWSNSTCLKLAVSSRLRPFVAHTCTQMLLSDMWMGRLNMRKNSWYKVILSILVPPA

ILMLEYKTKAEMSHIPQSQDAHQMTMEDSENNFHNITEEIPMEVFKEVKILDSSDGKNEMEIHIKSKKLP

ITRKFYAFYHAPIVKFWFNTLAYLGFLMLYTFVVLVKMEQLPSVQEWIVIAYIFTYAIEKVREVFMSEAG

KISQKIKVWFSDYFNVSDTIAIISFFVGFGLRFGAKWNYINAYDNHVFVAGRLIYCLNIIFWYVRLLDFL

AVNQQAGPYVMMIGKMVANMFYIVVIMALVLLSFGVPRKAILYPHEEPSWSLAKDIVFHPYWMIFGEVYA

YEIDVCANDSTLPTICGPGTWLTPFLQAVYLFVQYIIMVNLLIAFFNNVYLQVKAISNIVWKYQRYHFIM

AYHEKPVLPPPLIILSHIVSLFCCVCKRRKKDKTSDGPKLFLTEEDQKKLHDFEEQCVEMYFDEKDDKFN

SGSEERIRVTFERVEQMSIQIKEVGDRVNYIKRSLQSLDSQIGHLQDLSALTVDTLKTLTAQKASEASKV

HNEITRELSISKHLAQNLIDDVPVRPLWKKPSAVNTLSSSLPQGDRESNNPFLCNIFMKDEKDPQYNLFG

QDLPVIPQRKEFNIPEAGSSCGALFPSAVSPPELRQRRHGVEMLKIFNKNQKLGSSPNSSPHMSSPPTKF

SVSTPSQPSCKSHLESTTKDQEPIFYKAAEGDNIEFGAFVGHRDSMDLQRFKETSNKIRELLSNDTPENT

LKHVGAAGYSECCKTSTSLHSVQAESCSRRASTEDSPEVDSKAALLPDWLRDRPSNREMPSEGGTLNGLA

SPFKPVLDTNYYYSAVERNNLMRLSQSIPFVPVPPRGEPVTVYRLEESSPSILNNSMSSWSQLGLCAKIE

FLSKEEMGGGLRRAVKVLCTWSEHDILKSGHLYIIKSFLPEVINTWSSIYKEDTVLHLCLREIQQQRAAQ

KLTFAFNQMKPKSIPYSPRFLEVFLLYCHSAGQWFAVEECMTGEFRKYNNNNGDEIIPTNTLEEIMLAFS

HWTYEYTRGELLVLDLQGVGENLTDPSVIKAEEKRSCDMVFGPANLGEDAIKNFRAKHHCNSCCRKLKLP

DLKRNDYTPDKIIFPQDESSDLNLQSGNSTKESEATNSVRLML

>NP_599013.1 transient receptor potential cation channel subfamily M member 8 [Mus musculus]

MSFEGARLSMRSRRNGTMGSTRTLYSSVSRSTDVSYSDSDLVNFIQANFKKRECVFFTRDSKAMENICKC

GYAQSQHIEGTQINQNEKWNYKKHTKEFPTDAFGDIQFETLGKKGKYLRLSCDTDSETLYELLTQHWHLK

TPNLVISVTGGAKNFALKPRMRKIFSRLIYIAQSKGAWILTGGTHYGLMKYIGEVVRDNTISRNSEENIV

AIGIAAWGMVSNRDTLIRSCDDEGHFSAQYIMDDFTRDPLYILDNNHTHLLLVDNGCHGHPTVEAKLRNQ

LEKYISERTSQDSNYGGKIPIVCFAQGGGRETLKAINTSVKSKIPCVVVEGSGQIADVIASLVEVEDVLT

SSMVKEKLVRFLPRTVSRLPEEEIESWIKWLKEILESSHLLTVIKMEEAGDEIVSNAISYALYKAFSTNE

QDKDNWNGQLKLLLEWNQLDLASDEIFTNDRRWESADLQEVMFTALIKDRPKFVRLFLENGLNLQKFLTN

EVLTELFSTHFSTLVYRNLQIAKNSYNDALLTFVWKLVANFRRSFWKEDRSSREDLDVELHDASLTTRHP

LQALFIWAILQNKKELSKVIWEQTKGCTLAALGASKLLKTLAKVKNDINAAGESEELANEYETRAVELFT

ECYSNDEDLAEQLLVYSCEAWGGSNCLELAVEATDQHFIAQPGVQNFLSKQWYGEISRDTKNWKIILCLF

IIPLVGCGLVSFRKKPIDKHKKLLWYYVAFFTSPFVVFSWNVVFYIAFLLLFAYVLLMDFHSVPHTPELI

LYALVFVLFCDEVRQWYMNGVNYFTDLWNVMDTLGLFYFIAGIVFRLHSSNKSSLYSGRVIFCLDYIIFT

LRLIHIFTVSRNLGPKIIMLQRMLIDVFFFLFLFAVWMVAFGVARQGILRQNEQRWRWIFRSVIYEPYLA

MFGQVPSDVDSTTYDFSHCTFSGNESKPLCVELDEHNLPRFPEWITIPLVCIYMLSTNILLVNLLVAMFG

YTVGIVQENNDQVWKFQRYFLVQEYCNRLNIPFPFVVFAYFYMVVKKCFKCCCKEKNMESNACCFRNEDN

ETLAWEGVMKENYLVKINTKANDNSEEMRHRFRQLDSKLNDLKSLLKEIANNIK

>NP_444407.1 mucolipin-1 [Mus musculus]

MATPAGRRASETERLLTPNPGYGTQVGTSPAPTTPTEEEDLRRRLKYFFMSPCDKFRAKGRKPCKLMLQV

VKILVVTVQLILFGLSNQLVVTFREENTIAFRHLFLLGYSDGSDDTFAAYTQEQLYQAIFYAVDQYLILP

EISLGRYAYVRGGGGPWANGSALALCQRYYHRGHVDPANDTFDIDPRVVTDCIQVDPPDRPPDIPSEDLD

FLDGSASYKNLTLKFHKLINVTIHFQLKTINLQSLINNEIPDCYTFSILITFDNKAHSGRIPIRLETKTH

IQECKHPSVSRHGDNSFRLLFDVVVILTCSLSFLLCARSLLRGFLLQNEFVVFMWRRRGREISLWERLEF

VNGWYILLVTSDVLTISGTVMKIGIEAKNLASYDVCSILLGTSTLLVWVGVIRYLTFFHKYNILIATLRV

ALPSVMRFCCCVAVIYLGYCFCGWIVLGPYHVKFRSLSMVSECLFSLINGDDMFVTFAAMQAQQGHSSLV

WLFSQLYLYSFISLFIYMVLSLFIALITGAYDTIKHPGGTGTEKSELQAYIEQCQDSPTSGKFRRGSGSA

CSLFCCCGRDSPEDHSLLVN

>XP_006502032.1 mucolipin-2 isoform X1 [Mus musculus]

MPGDEETLDLPAWNRVPDLTWGPHHRSAMASLDSEVREECLREDLKFYFMSPCEKYRARRQIPWKLGLQI

LKIVMVTTQLVRFGLSNQLVVAFKEDNTVAFKHLFLKGFSGVDEDDYSCSIYTQENTYESIFFAIKQYRH

LKNISLATLGYGESEDNRTGLKVCKQHYKTGAMFSSNETLNIDSDIETDCIHLDLQVLTTEPEDWAQTSF

FRLDFYRLVQVDISFALKGIDLQAVHSREIPDCYLFQNTITFDNTAHSGKIKIYLNSEANIEECKNMNIS

GSTQRSTHYLLVFDVFVIMICLASLILCTRSIVLALRLRKRFLNFFLEKYKQRVCGADQWEFVNGWYVLV

TISDLMTIIGSILKMEIKAKKLTNYDVCSILLGTSTLFVWVGVIRYLGYFQTYNVLILTMQASLPKVLRF

CACAGMIYLGYTFCGWIVLGPYHEKFENLNIVAECLFSLVNGDDMFATFAQIQQKSILVWLFSRLYLYSF

ISLFIYMVLSLFIALITDSYHTIKYQQHGFPETDLQKFLKESGSKDGYQKQPSALLSCLCCLRRRRSNDH

LILID

>NP_598921.1 mucolipin-3 [Mus musculus]

MANPEVLVSSCRARQDESPCTFHPSSSPSEQLLLEDQMRRKLKFFFMNPCEKFWARGRKPWKLAIQILKI

AMVTIQLVLFGLSNQMVVAFKEENTIAFKHLFLKGYMDRMDDTYAVYTQSEVYDQIIFAVTQYLQLQNIS

VGNHAYENKGTKQSAMAICQHFYRQGTICPGNDTFDIDPEVETECFLVEPDEASHLGTPGENKLNLSLDF

HRLLTVELQFKLKAINLQTVRHQELPDCYDFTLTITFDNKAHSGRIKISLDNDISIKECKDWHVSGSIQK

NTHYMMIFDAFVILTCLASLVLCARSVIRGLQLQQEFVNFFLLHYKKEVSASDQMEFINGWYIMIIISDI

LTIVGSVLKMEIQAKSLTSYDVCSILLGTSTMLVWLGVIRYLGFFAKYNLLILTLQAALPNVMRFCCCAA

MIYLGYCFCGWIVLGPYHEKFRSLNRVSECLFSLINGDDMFSTFAKMQQKSYLVWLFSRVYLYSFISLFI

YMILSLFIALITDTYETIKHYQQDGFPETELRKFIAECKDLPNSGKYRLEDDPPGSLLCCCKK

>NP_852087.2 polycystic kidney disease 2-like 1 protein [Mus musculus]

MNSMESPKNQELQTLGNRAWDNPAYSDPPSPNRTLRICTVSSVALPETQPKKPEVRCQEKTQRTLVSSCC

LHICRSIRGLWGTTLTENTAENRELYVKTTLRELVVYIVFLVDICLLTYGMTSSSAYYYTKVMSELFLHT

PSDSGVSFQTISSMSDFWDFAQGPLLDSLYWTKWYNNQSLGRGSHSFIYYENLLLGAPRLRQLRVRNDSC

VVHEDFREDILNCYDVYSPDKEDQLPFGPQNGTAWTYHSQNELGGSSHWGRLTSYSGGGYYLDLPGSRQA

SAEALQGLQEGLWLDRGTRVVFIDFSVYNANINLFCILRLVVEFPATGGTIPSWQIRTVKLIRYVNNWDF

FIVGCEVVFCVFIFYYVVEEILEIHLHRLRYLSSVWNILDLVVILLSIVAVGFHIFRTLEVNRLMGKLLQ

QPDTYADFEFLAFWQTQYNNMNAVNLFFAWIKIFKYISFNKTMTQLSSTLARCAKDILGFAIMFFIVFFA

YAQLGYLLFGTQVENFSTFVKCIFTQFRIILGDFDYNAIDNANRILGPVYFVTYVFFVFFVLLNMFLAII

NDTYSEVKEELAGQKDQLQLSDFLKQSYNKTLLRLRLRKERVSDVQKVLKGGEPEIQFEDFTSTLRELGH

EEHEITAAFTRFDQDGDHILDEEEQEQMRQGLEEERVTLNAEIENLGRSVGHSPPGELGAEAARGQSWVS

GEEFDMLTRRVLQLQCVLEGVVSQIDAVGSKLKMLERKGELAPSPGMGEPAVWENLYNPS

>NP_001156476.1 polycystic kidney disease 2-like 2 protein [Mus musculus]

MSEATWWYRGGTSKHDLHYRREAEVNTTLEELLLYFIFLINLCILTFGMVNPHMYYLNKVMSSLFVDTSL

PDDERSSFRSIRSITEFWKFMEGPLIDGLYWDSWYGNKQLYSVKNSSRIYYENVLLGIPRVRQLRVRNNT

CKVYPAFQSLVSDCYSKYTVENEDFSDFGLKRNPEWTHTPSSRTAPWHWGFVGVYRDGGYIVTLSKSKSE

TKAKFVDLRLNNWISRGTRAVFIDFSLYNANVNLFCIIRLLAEFPATGGLLTSWQFYSVKLLRYVSYYDY

FIASCEVIFCIFLFVFIIQELRKVNEFKSAYFRSVWNWLEMLLLLLCFLAVSFYAYCNMQSFLLLGQLLK

NTDSYPDFYFLAYWHIYYNNVIAITIFFAWIKIFKFISFNETMSQLSSTLSRCMKDIVGFAIMFFIIFSA

YAQLGFLVFGSQVDDFSTFQNSIFAQFRIVLGDFNFAGIQQANWILGPIYFITFIFFVFFVLLNMFLAII

NDTYSEVKADYSIGRRPDFELGKIIQKSCFNVLEKLRLKKAQAKEEKKMQTTDLAQRARRDGFDESEIQE

AEQMKRWKERLEKKYYSTEIQDDYQPVTQQEFRELFLYAVELEKELHYVSLKLNQLMRKLH

>NP_001001445.1 transient receptor potential cation channel subfamily V member 1 [Mus musculus]

MEKWASLDSDESEPPAQENSCPDPPDRDPNSKPPPAKPHIFATRSRTRLFGKGDSEEASPMDCPYEEGGL

ASCPIITVSSVVTLQRSVDGPTCLRQTSQDSVSTGVETPPRLYDRRSIFDAVAQSNCQELESLLSFLQKS

KKRLTDSEFKDPETGKTCLLKAMLNLHNGQNDTIALLLDIARKTDSLKQFVNASYTDSYYKGQTALHIAI

ERRNMALVTLLVENGADVQAAANGDFFKKTKGRPGFYFGELPLSLAACTNQLAIVKFLLQNSWQPADISA

RDSVGNTVLHALVEVADNTADNTKFVTNMYNEILILGAKLHPTLKLEELTNKKGLTPLALAASSGKIGVL

AYILQREIHEPECRHLSRKFTEWAYGPVHSSLYDLSCIDTCEKNSVLEVIAYSSSETPNRHDMLLVEPLN

RLLQDKWDRFVKRIFYFNFFVYCLYMIIFTTAAYYRPVEGLPPYKLNNTVGDYFRVTGEILSVSGGVYFF

FRGIQYFLQRRPSLKSLFVDSYSEILFFVQSLFMLVSVVLYFSHRKEYVASMVFSLAMGWTNMLYYTRGF

QQMGIYAVMIEKMILRDLCRFMFVYLVFLFGFSTAVVTLIEDGKNNSLPVESPPHKCRGSACRPGNSYNS

LYSTCLELFKFTIGMGDLEFTENYDFKAVFIILLLAYVILTYILLLNMLIALMGETVNKIAQESKNIWKL

QRAITILDTEKSFLKCMRKAFRSGKLLQVGFTPDGKDDFRWCFRVDEVNWTTWNTNVGIINEDPGNCEGV

KRTLSFSLRSGRVSGRNWKNFALVPLLRDASTRDRHSTQPEEVQLKHYTGSLKPEDAEVFKDSMAPGEK

>NP_035836.2 transient receptor potential cation channel subfamily V member 2 isoform 1 [Mus musculus]

MTSASNPPAFRLETSDGDEEGSAEVNKGKNEPPPMESPFQGEDRNFSPQIKVNLNYRKGLGPSQQDPNRF

DRDRLFSVVSRGVPEELTGLLEYLRRTSKYLTDSAYTEGSTGKTCLMKAVLNLQDGVNACILPLLQIDRD

SGNPQPLVNAQCTDEFYRGHSALHIAIEKRSLWCVKLLVENGANVHIRACGRFFQKHQGTCFYFGELPLS

LAACTKQWDVVTYLLENPHQPASLEATDSLGNTVLHALVMIADNSPENSALVIHMYDSLLQMGARLCPTV

QLEDICNHQGLTPLKLAAKEGKIEIFRHILQREFSGLYQPLSRKFTEWCYGPVRVSLYDLSSVDSWEKNS

VLEIIAFHCKSPHRHRMVVLEPLNKLLQEKWDRLIPRFFFNFACYLVYMIIFTIVAYHQPSLEQPAIPSS

KATFGDSMLLLGHILILLGGIYLLLGQLWYFWRRRLFIWISFMDSYFEILFLVQALLTVLSQVLRFVETE

WYLPLLVSSLVLGWLNLLYYTRGFQHTGIYSVMIQKVILRDLLRFLLVYLVFLFGFAVALVSLSREARSP

KAPEDSNTTVTEKPTLGQEEEPVPYGGILDASLELFKFTIGMGELAFQEQLRFRGVVLLLLLAYVLLTYV

LLLNMLIALMSETVNSVATDSWSIWKLQKAISVLEMENGYWWCRRKRHRAGRLLKVGTKGDGIPDERWCF

RVEEVNWAAWEKTLPTLSEDPSGAGITGYKKNPTSKPGKNSASEEDHLPLQVLQSH

>NP_659567.2 transient receptor potential cation channel subfamily V member 3 isoform 1 [Mus musculus]

MNAHSKEMAPLMGKRTTAPGGNPVVLTEKRPADLTPTKKSAHFFLEIEGFEPNPTVTKTSPPIFSKPMDS

NIRQCLSGNCDDMDSPQSPQDDVTETPSNPNSPSANLAKEEQRQKKKRLKKRIFAAVSEGCVEELRELLQ

DLQDLCRRRRGLDVPDFLMHKLTASDTGKTCLMKALLNINPNTKEIVRILLAFAEENDILDRFINAEYTE

EAYEGQTALNIAIERRQGDITAVLIAAGADVNAHAKGVFFNPKYQHEGFYFGETPLALAACTNQPEIVQL

LMENEQTDITSQDSRGNNILHALVTVAEDFKTQNDFVKRMYDMILLRSGNWELETMRNNDGLTPLQLAAK

MGKAEILKYILSREIKEKPLRSLSRKFTDWAYGPVSSSLYDLTNVDTTTDNSVLEIIVYNTNIDNRHEML

TLEPLHTLLHTKWKKFAKYMFFLSFCFYFFYNITLTLVSYYRPREDEDLPHPLALTHKMSWLQLLGRMFV

LIWATCISVKEGIAIFLLRPSDLQSILSDAWFHFVFFVQAVLVILSVFLYLFAYKEYLACLVLAMALGWA

NMLYYTRGFQSMGMYSVMIQKVILHDVLKFLFVYILFLLGFGVALASLIEKCSKDKKDCSSYGSFSDAVL

ELFKLTIGLGDLNIQQNSTYPILFLFLLITYVILTFVLLLNMLIALMGETVENVSKESERIWRLQRARTI

LEFEKMLPEWLRSRFRMGELCKVADEDFRLCLRINEVKWTEWKTHVSFLNEDPGPIRRTADLNKIQDSSR

SNSKTTLYAFDELDEFPETSV

>XP_006530495.2 transient receptor potential cation channel subfamily V member 4 isoform X1 [Mus musculus]

MADPGDGPRAAPGEVAEPPGDESGTSGGEAFPLSSLANLFEGEEGSSSLSPVDASRPAGPGDGRPNLRMK

FQGAFRKGVPNPIDLLESTLYESSVVPGPKKAPMDSLFDYGTYRHHPSDNKRWRRKVVEKQPQSPKAPAP

QPPPILKVFNRPILFDIVSRGSTADLDGLLSFLLTHKKRLTDEEFREPSTGKTCLPKALLNLSNGRNDTI

PVLLDIAERTGNMREFINSPFRDIYYRGQTSLHIAIERRCKHYVELLVAQGADVHAQARGRFFQPKDEGG

YFYFGELPLSLAACTNQPHIVNYLTENPHKKADMRRQDSRGNTVLHALVAIADNTRENTKFVTKMYDLLL

LKCSRLFPDSNLETVLNNDGLSPLMMAAKTGKIGVFQHIIRREVTDEDTRHLSRKFKDWAYGPVYSSLYD

LSSLDTCGEEVSVLEILVYNSKIENRHEMLAVEPINELLRDKWRKFGAVSFYINVVSYLCAMVIFTLTAY

YQPLEGTPPYPYRTTVDYLRLAGEVITLFTGVLFFFTSIKDLFTKKCPGVNSLFVDGSFQLLYFIYSVLV

VVSAALYLAGIEAYLAVMVFALVLGWMNALYFTRGLKLTGTYSIMIQKILFKDLFRFLLVYLLFMIGYAS

ALVTLLNPCTNMKVCDEDQSNCTVPTYPACRDSETFSAFLLDLFKLTIGMGDLEMLSSAKYPVVFILLLV

TYIILTFVLLLNMLIALMGETVGQVSKESKHIWKLQWATTILDIERSFPVFLRKAFRSGEMVTVGKSSDG

TPDRRWCFRVDEVNWSHWNQNLGIINEDPGKSEIYQYYGFSHTVGRLRRDRWSSVVPRVVELNKNSSADE

VVVPLDNLGNPNCDGHQQGYAPKWRTDDAPL

>NP_001007573.1 transient receptor potential cation channel subfamily V member 5 [Mus musculus]

MGAKTPWIQLQKLLNWWVRDQDWNQHVDQLHMLQQKSIWESPLLRAAKENDMCTLKKLQHDQNCDFRQRG

ALGETALHVAALYDNLDAAIMLMEAAPYLVTESTLCEPFVGQTALHIAVMNQNVNLVRALLARGASASAR

ATGSAFHRSSHNLIYYGEHPLSFAACVGSEEIVRLLIEHGADIRAQDSLGNTVLHILVLQPNKTFACQMY

NLLLSYDGGDHLKSLELVPNNQGLTPFKLAGVEGNTVMFQHLMQKRKRIQWSFGPLTSSLYDLTEIDSWG

EELSFLELVVSSKKKEARQILEQTPVKELVSLKWKKYGQPYFCLLGALYIFYMVCFTTCCVYRPLKFRDA

NRTHVRDNTIMEQKSLQEAYVTYQDKIRLVGELVTVIGAVIILLLEIPDIFRVGASRYFGQTVLGGPFHV

IIITYASLVLLTMAMRLTNVNGEVVPMSMALVLGWCSVMYFARGFQMLGPFTIMIQKMIFGDLLRFCWLM

AMVILGFASAFYIIFQTEDPDNLGEFSDYPTAMFSTFELFLTIIDGPANYRVDLPFMYSVTYATFAIIAT

LLMLNLFIAMMGDTHWRVAQERDELWRAQVVATTVMLERKMPRFLWPRSGICGCEYGLGDRWFLRVEHHQ

EQNPYRVLRYVEAFKSSDKEEVQEQLSEKQPSGTETGTLARGSVVLQTPPLSRTTSLSSNSHRGWEILRR

NTLGHLNLGLDPGEGDGEEIYQF

>NP_071858.3 transient receptor potential cation channel subfamily V member 6 [Mus musculus]

MGPLQREDRPALGGANVAPGSSPVGVWHQPQPPKEPAFHPMGWSLPKEKGLILCLWNKFCRWFHRQESWA

QSRDEQNLLQQKRIWESPLLLAAKENDVQALSKLLKFEGCEVHQRGAMGETALHIAALYDNLEAAMVLME

AAPELVFEPMTSELYEGQTALHIAVINQNVNLVRALLARGASVSARATGSVFHYRPHNLIYYGEHPLSFA

ACVGSEEIVRLLIEHGADIRAQDSLGNTVLHILILQPNKTFACQMYNLLLSYDGGDHLKSLELVPNNQGL

TPFKLAGVEGNIVMFQHLMQKRKHIQWTYGPLTSTLYDLTEIDSSGDDQSLLELIVTTKKREARQILDQT

PVKELVSLKWKRYGRPYFCVLGAIYVLYIICFTMCCVYRPLKPRITNRTNPRDNTLMQQKLLQEAYVTPK

DDLRLVGELVSIVGAVIILLVEIPDIFRLGVTRFFGQTILGGPFHVIIITYAFMVLVTMVMRLTNVDGEV

VPMSFALVLGWCNVMYFARGFQMLGPFTIMIQKMIFGDLMRFCWLMAVVILGFASAFYIIFQTEDPDELG

HFYDYPMALFSTFELFLTIIDGPANYDVDLPFMYSVTYAAFAIIATLLMLNLLIAMMGDTHWRVAHERDE

LWRAQVVATTVMLERKLPRCLWPRSGICGREYGLGDRWFLRVEDRQDLNRQRIRRYAQAFQQQDGLYSED

LEKDSGEKLETARPFGAYLSFPTPSVSRSTSRSSTNWERLRQGALRKDLRGIINRGLEDGEGWEYQI

>NP_001261600.1 transient receptor potential cation channel A1, isoform G [Drosophila melanogaster]

MPKLYNGVYSGQCGALSPPDLMEAQPKLLPKPRSNSSGSTGRNSKYWIFSMIIERSAGPKRIEIDGDDAD

TPLEAILPAEPPAEVCLLRDSPFRILRAAESGNLDDFKRLFMADNSRIALKDAKGRTAAHQAAARNRVNI

LRYIRDQNGDFNAKDNAGNTPLHIAVESDAYDALDYLLSIPVDTGVLNEKKQAPVHLATELNKVKSLRVM

GQYRNVIDIQQGGEHGRTALHLAAIYDHEECARILITEFDACPRKPCNNGYYPIHEAAKNASSKTMEVFF

QWGEQRGCTREEMISFYDSEGNVPLHSAVHGGDIKAVELCLKSGAKISTQQHDLSTPVHLACAQGAIDIV

KLMFEMQPMEKRLCLSCTDVQKMTPLHCASMFDHPDIVSYLVAEGADINALDKEHRSPLLLAASRSGWKT

VHLLIRLGACISVKDAAARNVLHFVIMNGGRLTDFAEQVANCQTQAQLKLLLNEKDSMGCSPLHYASRDG

HIRSLENLIRLGACINLKNNNNESPLHFAARYGRYNTVRQLLDSEKGSFIINESDGAGMTPLHISSQQGH

TRVVQLLLNRGALLHRDHTGRNPLQLAAMSGYTETIELLHSVHSHLLDQVDKDGNTALHLATMENKPHAI

SVLMSMGCKLVYNVLDMSAIDYAIYYKYPEAALAMVTHEERANEVMALRSDKHPCVTLALIASMPKVFEA

VQDKCITKANCKKDSKSFYIKYSFAFLQCPFMFAKIDEKTGESITTASPIPLPALNTMVTHGRVELLAHP

LSQKYLQMKWNSYGKYFHLANLLIYSIFLVFVTIYSSLMMNNIELKAGDNKTMSQYCNMGWEQLTMNLSQ

NPSVASQIRLDSCEERINRTTAILFCAVVIVVYILLNSMRELIQIYQQKLHYILETVNLISWVLYISALV

MVTPAFQPDGGINTIHYSAASIAVFLSWFRLLLFLQRFDQVGIYVVMFLEILQTLIKVLMVFSILIIAFG

LAFYILLSKIIDPQPNHLSFSNIPMSLLRTFSMMLGELDFVGTYVNTYYRDQLKVPMTSFLILSVFMILM

PILLMNLLIGLAVGDIESVRRNAQLKRLAMQVVLHTELERKLPHVWLQRVDKMELIEYPNETKCKLGFCD

FILRKWFSNPFTEDSSMDVISFDNNDDYINAELERQRRKLRDISRMLEQQHHLVRLIVQKMEIKTEADDV

DEGISPNELRSVVGLRSAGGNRWNSPRVRNKLRAALSFNKSM

>NP_611979.1 painless, isoform A [Drosophila melanogaster]

MDFNNCGFIDPQAQLAGALAKQDIRQFVAALDSGALADLQDDRHTSIYEKALSTPGCRDFIEACIDHGSQ

VNYINKKLDKAAISYAADSRDPGNLAALLKYRPGNKVQVDRKYGQLTPLNSLAKNLTDENAPDVYSCMQL

LLDYGASPNIVDQGEFTPLHHVLRKSKVKAGKKELIQLFLDHPELDIDSYRNGEVRRLLQAQFPELKLPE

ERHTGPEIDIQTLQRTLRDGDETLFEQQFAEYLQNLKGGADNQLNAHQEEYFGLLQESIKRGRQRAFDVI

LSTGMDINSRPGRANEANLVETAVIYGNWQALERLLKEPNLRLTPDSKLLNAVIGRLDEPPYDGSSHQRC

FELLINSDRVDINEADSGRLVPLFFAVKYRNTSAMQKLLKNGAYIGSKSAFGTLPIKDMPPEVLEEHFDS

CITTNGERPGDQNFEIIIDYKNLMRQERDSGLNQLQDEMAPIAFIAESKEMRHLLQHPLISSFLFLKWHR

LSVIFYLNFLIYSLFTASIITYTLLKFHESDQRALTAFFGLLSWLGISYLILRECIQWIMSPVRYFWSIT

NIMEVALITLSIFTCMESSFDKETQRVLAVFTILLVSMEFCLLVGSLPVLSISTHMLMLREVSNSFLKSF

TLYSIFVLTFSLCFYILFGKSVEEDQSKSATPCPPLGKKEGKDEEQGFNTFTKPIEAVIKTIVMLTGEFD

AGSIQFTSIYTYLIFLLFVIFMTIVLFNLLNGLAVSDTQVIKAQAELNGAICRTNVLSRYEQVLTGHGRA

GFLLGNHLFRSICQRLMNIYPNYLSLRQISVLPNDGNKVLIPMSDPFEMRTLKKASFQQLPLSAAVPQKK

LLDPPLRLLPCCCSLLTGKCSQMSGRVVKRALEVIDQKNAAEQRRKQEQINDSRLKLIEYKLEQLIQLVQ

DRK

>NP_612015.1 pyrexia, isoform A [Drosophila melanogaster]

MENVRFSIIENDLKWNSESDPTADVDLLLDKRSISSEANSAGVFSDEAHEIFARQQQNQILWDLDEVKET

LTESPGGKHVVDMVQSGCFLELMTDSADCNLALICCSVFGSVENTLFLLKHYNADPNVADSRGRTPLHFA

CCRANAPIAKVLLDFGADPNRWDARKEVTSLHCAASSKSVECILLLLRRKASINIGIEKRSALHYAIDVN

AVDCVEILLKYGADPNTPQVYTETPLHTASAAGFAKCVQLLLSHNADVRSQFGEGKVTALHLAAENDYVE

CARLLLEHRAEVDCRNASHQTPLHLACLSQSIGTVDLLISYGANVNAVYRDGRTALHAAIVKQSRSLDCC

NALLKAGADVNKADNYGYTPLHIAALNEFSSCVYTFIEHGADITARTDGRVSALSFIVRRTPEIIPKLMQ

KLDSSIKANDQEIGDVDCQIKLDFRLLVPSSSMDRGETELLLSLIEVGQKRILMHPLCETFLFLKWRRIR

KFFLMSLAYHTLFVILFTFYVIWVYVRCCKKEELCVAPGYVSTIGYLVIILNLILLGKEVFQMAHGLRGY

AKYWENWLQWTIGTGVLLCVTPETVRTDDLTAVPVWQHHVAAIVILLVWLELMMLVGRFPIFGVYVQMFT

KVAVNFAKFLLAYICLLVAFGLSFAVLFNDYPAFENITWSFLKSITMMSGELEFEDIFYGDYAVKFPVTA

HIIFLSFVLLVTVILTNLMVGLAVSDIQGLQVSATLDRLVRQAELVSRLESLFFSRLLRSAPTNLIQLCK

RSALLRTSRDKLQFTIRPNDPRDNQLPEDIKLNVYKLVAERRDRNQSLRRRQFENNYNIFSRSLQRQQQP

LHTDFLKPEPATGTTKKTPQNLFHMHELLRPRSATNVPQQFRQEAEGTVQMKNQANVLSAVLAEVQAIKT

QLVDLVAKFERFSENATRKLNYSTDELCRLRQQGQSVASSHIRRHR

>NP_731193.1 water witch, isoform A [Drosophila melanogaster]

MENLGYKEGSTKPRRMTRSISVVTKTEVEQPLTENNANNRFKSIPPNLMVRWRNNTDAMIEAQYPTAGEF

EYMECGPSPPAESAPSMYDSFEEPTSELSVQICNDTLRISLIDQMKSAAGRVKLFEDIEQSNVVAEGIRT

HFESASKLEKNLCYLWAAYLKRWDLIESLLEAGADLHFCDQNGISALHLSAFSGCLATLGLLVAKGLNVN

LQSKCYTPLHCAAFGNAAEAAKLLINNGADISKDTSKPNCEESLLHCAVRSNALECLQIFIAEGADVNSL

KPNGTNAIHLAADLGNIQCLEALLNAPNADANVRICIREKESTALHLAADEGNVECVDLLLAKGADAKLK

NHRGFTPLHLAARTSSLDCVESLLRNGNADANAEDFDHRTPLHAAVGKSENAYDIMETLIQWGANVNHKD

IYGFTALHLAALDGLVQCVEMLIFHGADVTTKSKKGTSALNVITRKTPASVAMIRQKLDAAITLHHSQDP

VNREVELELDFRQLLQHCHPREISYLNTFVDEGQKEILEHPLCSSFLYIKWGKIRKYYIGRLIFCFSFVL

FLTLYVLTALAHNCYNGSKNDNTTIPAQELCQKQSILGDMLRNNPFVMEMQWWVLVAITIVEIFRKLYGI

TGYSSFRHYVTQVENIMEWFVITSVFVISYIYTNKTYTFQNHIGAFAVLLGWTNLMLMIGQLPVFDVYVA

MYTRVQGEFAKLFMAYSCMLIGFTISFCVIFPSSSSFANPFMGFITVLVMMIGEQDLSLLINDPEGKDPP

FLLEVSAQITFVLFLLFVTIILMNLLVGIAVHDIQGLKKTAGLSKLVRQTKLISYIESALFNGYLPTWLR

NLLHYTALVSPQAYRVVLCVKPLNPSEKRLPREILMKAYEVGKMRKHFGHTISSKNSAENYLSYKNKYNN

NNGATTGYVLPDSDPDAGQFTTLTTKIDDNADRIEFLTQEIQELKQALISQQQQASKVIDKLLIVISNQQ

KQNLRK

>NP_609802.1 transient receptor potential cation channel gamma, isoform A [Drosophila melanogaster]

MMEEENTIRPHQEIRQLTLEEKKFLLAVERGDMAGTRRMLQKAQDTEYINVNCVDPLGRTALLMAIDNEN

LEMVELLINYNVDTKDALLHSISEEFVEAVEVLLDHENVTFHSEGNHSWESASEDTSTFTPDITPLILAA

HRDNYEIIKILLDRGAVLPMPHDVRCGCDECVQSRQEDSLRHSRSRINAYRALASPSLIALSSKDPILTA

FELSWELRRLSFLEHEFKNEYQELRKQCQDFATALLDHTRTSHELEILLNHDPTGPVYEHGERMHLNRLK

LAIKLRQKKFVAHSNVQQLLASIWYEGLPGFRRKNMALQAVDIIRIGIMFPIFSLAYILAPYSSIGQTMR

KPFIKFICHSASYFTFLFLLMLASQRIETFIGGWFFADSSGMLNTMEELPTKRGAKPTFIEWLILAWVSG

LIWSEVKQLWDVGLQEYLNDMWNVIDFVTNSLYVATVALRVVSFFQVQKEMIYNSHATDLPRERWDAWDP

MLISEGLFSAANIFSSLKLVYIFSVNPHLGPLQVSLSRMVMDIMKFFFLYVLVLFAFGSGLNQLLWYYAD

LEKKRCPEVSPMSALLNMNGTNDPNACIVWRRFSNLFETTQTLFWAVFGLIDLDSFELDGIKIFTRFWGM

LMFGTYSVINIVVLLNLLIAMMNHSYQLISERADVEWKFARSKLWISYFEEGGTCPPPFNIIPTPKSIWY

AIKWMRRVFCSGSSAARREHLKTIRRKAQQASDRDFKYQQIMRNLVRRYVTVEQRKAESQGVTEDDVNEI

KQDISAFRCELVEILKNSGMDTNVTAGQGGGGGGKKNRQKERRLMKGFNIAPPGSTGSLAPVAEFSTSLD

NYDNQHEILSSTLSTLFTPNFMHKRQQSQAGSGGGGSESPTTPTAPQGTQGAAMTASSQVTKYNKSALKP

YNKRIAGHKKRWGTLIEAAKVGNVSKMLGRSKSEDSVCNSSHTSTPVHGQMRVTYAQNSPQQEYGYHGET

SSTTISTPTPTISVVSNSPAAHAGVGSHFFHTTSGLTAIAALKRKRKKFSSSKNICPVTESVAAANAAEI

LNDKTLKRVSSYPAAEAGVQHNPAQLVKPRRHEQTQSQHDSVETNSTFTLSIDPSNTSVNSREPLISTSC

VSTTGAIG

>NP_476895.1 transient receptor potential-like, isoform A [Drosophila melanogaster]

MGRKKKLPTGVSSGVSHASSAPKSVGGCCVPLGLPQPLLLEEKKFLLAVERGDMPNVRRILQKALRHQHI

NINCMDPLGRRALTLAIDNENLEMVELLVVMGVETKDALLHAINAEFVEAVELLLEHEELIYKEGEPYSW

QKVDINTAMFAPDITPLMLAAHKNNFEILRILLDRGAAVPVPHDIRCGCEECVRLTAEDSLRHSLSRVNI

YRALCSPSLICLTSNDPIITAFQLSWELRNLALTEQECKSEYMDLRRQCQKFAVDLLDQTRTSNELAIIL

NYDPQMSSYEPGDRMSLTRLVQAISYKQKKFVAHSNIQQLLSSIWYDGLPGFRRKSIVDKVICIAQVAVL

FPLYCLIYMCAPNCRTGQLMRKPFMKFLIHASSYLFFLFILILVSQRADDDFVRIFGTTRMKKELAEQEL

RQRGQTPSKLELIVVMYVIGFVWEEVQEIFAVGMKSYLRNMWNFIDFLRNSLYVSVMCLRAFAYIQQATE

IARDPQMAYIPREKWHDFDPQLIAEGLFAAANVFSALKLVHLFSINPHLGPLQISLGRMVIDIVKFFFIY

TLVLFAFACGLNQLLWYFAALEKSKCYVLPGGEADWGSHGDSCMKWRRFGNLFESSQSLFWASFGMVGLD

DFELSGIKSYTRFWGLLMFGSYSVINVIVLLNLLIAMMSNSYAMIDEHSDTEWKFARTKLWMSYFEDSAT

LPPPFNVLPSVKWVIRIFRKSSKTIDRQRSKKRKEQEQFSEYDNIMRSLVWRYVAAMHRKFENNPVSEDD

INEVKSEINTMRYEMLEIFENSGMDVSSANKKERQPRPRRIKVWERRLMKGFQVAPVQNGCELDAFGNVN

GQGEMQEIKVESIPSKPAKETAKERFQRVARTVLLQSTTHKWNVVLRAAKDSQIGRCTKNERKSLQNLGR

AIEEAKRLIMLNPGCPSGRESPIRIEFEDEKTSTLLELLNQISAEISDSEKPKIRPIWRPPLKTVPARAM

AANNTRSLTAPELKISRKSSPAPTPTPTPGVSHTALSQFRNRELPLCPSKLIANSAPSAPTAPPKKSAPT

APTPTYKPTTHAPFSVEGGNRENTRASDGVRSDNSNFDIHVVDLDEKGGHLGRDNVSDISSIASTSPQRP

KHRN

>NP_476768.1 transient receptor potential, isoform A [Drosophila melanogaster]

MGSNTESDAEKALGSRLDYDLMMAEEYILSDVEKNFILSCERGDLPGVKKILEEYQGTDKFNINCTDPMN

RSALISAIENENFDLMVILLEHNIEVGDALLHAISEEYVEAVEELLQWEETNHKEGQPYSWEAVDRSKST

FTVDITPLILAAHRNNYEILKILLDRGATLPMPHDVKCGCDECVTSQMTDSLRHSQSRINAYRALSASSL

IALSSRDPVLTAFQLSWELKRLQAMESEFRAEYTEMRQMVQDFGTSLLDHARTSMELEVMLNFNHEPSHD

IWCLGQRQTLERLKLAIRYKQKTFVAHPNVQQLLAAIWYDGLPGFRRKQASQQLMDVVKLGCSFPIYSLK

YILAPDSEGAKFMRKPFVKFITHSCSYMFFLMLLGAASLRVVQITFELLAFPWMLTMLEDWRKHERGSLP

GPIELAIITYIMALIFEELKSLYSDGLFEYIMDLWNIVDYISNMFYVTWILCRATAWVIVHRDLWFRGID

PYFPREHWHPFDPMLLSEGAFAAGMVFSYLKLVHIFSINPHLGPLQVSLGRMIIDIIKFFFIYTLVLFAF

GCGLNQLLWYYAELEKNKCYHLHPDVADFDDQEKACTIWRRFSNLFETSQSLFWASFGLVDLVSFDLAGI

KSFTRFWALLMFGSYSVINIIVLLNMLIAMMSNSYQIISERADTEWKFARSQLWMSYFEDGGTIPPPFNL

CPNMKMLRKTLGRKRPSRTKSFMRKSMERAQTLHDKVMKLLVRRYITAEQRRRDDYGITEDDIIEVRQDI

SSLRFELLEIFTNNNWDVPDIEKKSQGVARTTKGKVMERRILKDFQIGFVENLKQEMSESESGRDIFSSL

AKVIGRKKTQKGDKDWNAIARKNTFASDPIGSKRSSMQRHSQRSLRRKIIEQANEGLQMNQTQLIEFNPN

LGDVTRATRVAYVKFMRKKMAADEVSLADDEGAPNGEGEKKPLDASGSKKSITSGGTGGGASMLAAAALR

ASVKNVDEKSGADGKPGTMGKPTDDKKAGDDKDKQQPPKDSKPSAGGPKPGDQKPTPGAGAPKPQAAGTI

SKPGESQKKDAPAPPTKPGDTKPAAPKPGESAKPEAAAKKEESSKTEASKPAATNGAAKSAAPSAPSDAK

PDSKLKPGAAGAPEATKATNGASKPDEKKSGPEEPKKAAGDSKPGDDAKDKDKKPGDDKDKKPGDDKDKK

PADNNDKKPADDKDKKPGDDKDKKPGDDKDKKPSDDKDKKPADDKDKKPAAAPLKPAIKVGQSSAAAGGE

RGKSTVTGRMISGWL

>NP_001137672.2 transient receptor potential cation channel, subfamily M, isoform A [Drosophila melanogaster]

MLGKFTSNHVQVYHQYSRPVPVKKPVKHQPRSWIETNFQKRECIKFIPCPKDDTKCCCGQAQITHQTIPG

IESGSPGDLWLPTKHTRPQPTDAYGTIEFQGGAHPTKAQYVRLSFDTRPELLVQLFTKEWNLELPKLLIT

VQGGKANFDLQAKLKKEIRKGLLKAAKTTGAWIFTGGTNTGVTKQVGDALLLEGQQRTGRVVSIGIAPWG

IVERNHELLGHNREVPCHSISSPRSKLAVLNNRHAYFLLVDNGTQAKYGAELILRRKLEKFISNLKLHPF

THSSTPVVCLVIEGGTNTIRAVLEYVTDSPPVPVVVCDGSGRAADLLAFVHKYASDGEEQPVLESMRDYL

IGTIQKTFEVGLDQSEKLYQELLQCTRNKNLITVFRIQEKPEGEAQELDQTILTALFKSQHLSPPEQLSL

ALTWNRVDIARSEIFVYGQEWPNGALDEAMMQALEHDRIDFVKLLLENGVSMKKFLTIPRLEELYNTKHG

PANTLGYILRDVRPHIPKGYIYTLHDIGLVINKLMGGAYRSYYTRRKFRPIYAKVMNSYANACRKSSTYQ

YQRYAGANSLSLVTGLLPFTSEMALFEFPFNELLIWAVLTKRQQMALLMWTHGEEALAKSLVSCKLYKAM

AHEAAEDDLDTEIYEELRSYAKEFESKGNKLLDFSYRQDAEKAQRLLTCELHSWSNQSCLSLAVAANHRA

LLAHPCSQVILADLWMGGLRTRKNTNFKVILGLAMPFYIRQLDFKSKEELQQMPQTEEEHLENQNLDNDD

SDRSQPDAEALLADTYSVRDTKVHENGKVSLTDSDTAQFREFFNLSEYNEVKQHQPLRLKKKFYEFYTAP

ITKFWADSIAYMFFLIMFSFTVLVKMEQMPRWQEWYSIAYITTLGFEKVREIISSEPVAITHKFSVWAWN

MWNPCDGAAIILFVIGLAFRFRENTMDIGRVIYCVDSIYWYLRILNILGVNKYLGPLVTMMGKMVKNMIY

FVVLLAVVLMSFGVSRQAILYPNKQPTWSLIKEVTFQPYFMLYGEVFAGDIDPPCGEDPSQPGCVTGHWV

TPITMSMYLLIANILLINLLIAVFNNIFNEVNSVSHQVWMFQRFTVVMEYQQKPVLPPPFIALCHFYSLL

KYCVRKAKGLEVQRDNGLKLFLEKDDLERLYDFEEECVEGFFHEQEIILNQSTDERVKNTTERVETMSQK

IEDINQKENIQTATVQNIEFRLRKMEESSEQILSHLAVIHRFMSTHTAGADDLRGSTINIPGEMQRMRTI

SISDTEGGSGPGGNGGGGGGGGAIVPLGLGAGLNLNSLQVTTRRRFNRSLTEVRPDAYIFDEGTHFEVVP

LPEEPDEVVKSREALNEQVVRKASMQSEADSDIYIPVSQRPSTCETVKRTPYVTVRQDTGASTESKDTLT

PMGNNDDDQTLVGGDNSDDATPDINFEAARHRALRQRTVSLCRRNSETYSLTGADINRSHISLNQLASLS

RRQMSLTQSEPDSDKDAPIAQGSAHPGKSVLHAKPSRNILLKLHSEYTSITDELESVCHMIASPTVSLPS

NKASLDRPKTEMSRAEAAALLEKKHLKECEENDYMILEGLIESRGSIDASAQGFEIGVSIDYSHRYPLRR

ETAVELSPSKPSVDGDLMGGGEGGGAGGGDSSDTSGAGSCGAMVGISSGFQLKNERPWQRNSSMEQQTYP

SPLVPTRATSDFLNPPYEGRLFKKSSESLQKNSSTETDYSAHPYRFIKQSSNETNTSLTGSYNVDTPSLT

AEPSLDAGDSHSATGISISVGAVGGTATARYQPIRTASVGAADGRRLREESSSSLDLSSSGPVTMQAAPA

PPVRPMLLKKQFSVDQGKPSQTAAEAVPQTPEAAQAGQAKLISTLKPQPFASKLGMNVLKESSSSTDESV

GSSAKSSNPALSIPQISTHLVQDEIAKLSSNIKSSTESEKDPPFNETMC

>NP_649145.1 transient receptor potential cation channel, mucolipin [Drosophila melanogaster]

MQSYGPGAQTAPAVKRRTDSYEAAQQQQQSPESDEEYVNTRILRRQVQLQSTPVAPVVPMPISAGSGTAP

PSVDGREEQPEFPGSSAASYQEERMRRKLQFFFMNPIEKWQAKRKFPYKFVVQIVKIFLVTMQLCLFAHS

RYNHINYTGDNRFAFSHLFLRGWDSSREVESYPPAVGPFALYLKSEFFDTVQYAVNGYANVSRSIGPYDY

PTPNNTMPPLKLCLQNYREGTIFGFNESYIFDPHIDEVCERLPPNVTTIGVENYLRQRDVEVNFASLVSA

QLTFKIKTVNFKANGGPLSAPDCFRFDISITFNNRDHDGQMLLSLDAEATRLKCHGATDFISDANFDSML

RSVLNIFVLLTCALSFALCTRALWRAYLLRCTTVNFFRSQFGKELSFDGRLEFVNFWYIMIIFNDVLLII

GSALKEQIEGRYLVVDQWDTCSLFLGIGNLLVWFGVLRYLGFFKTYNVVILTLKKAAPKILRFLIAALLI

YAGFVFCGWLILGPYHMKFRSLATTSECLFALINGDDMFATFATLSSKATWLWWFCQIYLYSFISLYIYV

VLSLFIAVIMDAYDTIKAYYKDGFPTTDLKAFVGTRTAEDISSGVFMTDLDDFDQTSFLDVVKSICCCGR

CGRHQEPAQPNSGYTSLSSIMK

>NP_001097089.2 no mechanoreceptor potential C, isoform D [Drosophila melanogaster]

MSQPRGGRGGGRGGGVGRKTPSSLTGPPDESATPSERATPASKADSDPKDDSSSNGDKKDMDLFPAPKPP

SAGASIRDTANKVLGLAMKSEWTPIEAELKKLEKYVANVGEDGNHIPLAGVHDMNTGMTPLMYATKDNKT

AIMDRMIELGADVGARNNDNYNVLHIAAMYSREDVVKLLLTKRGVDPFSTGGSRSQTAVHLVSSRQTGTA

TNILRALLAAAGKDIRLKADGRGKIPLLLAVESGNQSMCRELLAAQTAEQLKATTANGDTALHLAARRRD

VDMVRILVDYGTNVDTQNGEGQTPLHIAAAEGDEALLKYFYGVRASASIADNQDRTPMHLAAENGHAHVI

EILADKFKASIFERTKDGSTLMHIASLNGHAECATMLFKKGVYLHMPNKDGARSIHTAAAYGHTGIINTL

LQKGEKVDVTTNDNYTALHIAVESAKPAVVETLLGFGADVHVRGGKLRETPLHIAARVKDGDRCALMLLK

SGASPNLTTDDCLTPVHVAARHGNLATLMQLLEDEGDPLYKSNTGETPLHMACRACHPDIVRHLIETVKE

KHGPDKATTYINSVNEDGATALHYTCQITKEEVKIPESDKQIVRMLLENGADVTLQTKTALETAFHYCAV

AGNNDVLMEMISHMNPTDIQKAMNRQSSVGWTPLLIACHRGHMELVNNLLANHARVDVFDTEGRSALHLA

AERGYLHVCDALLTNKAFINSKSRVGRTALHLAAMNGFTHLVKFLIKDHNAVIDILTLRKQTPLHLAAAS

GQMEVCQLLLELGANIDATDDLGQKPIHVAAQNNYSEVAKLFLQQHPSLVNATSKDGNTCAHIAAMQGSV

KVIEELMKFDRSGVISARNKLTDATPLQLAAEGGHADVVKALVRAGASCTEENKAGFTAVHLAAQNGHGQ

VLDVLKSTNSLRINSKKLGLTPLHVAAYYGQADTVRELLTSVPATVKSETPTGQSLFGDLGTESGMTPLH

LAAFSGNENVVRLLLNSAGVQVDAATIENGYNPLHLACFGGHMSVVGLLLSRSAELLQSQDRNGRTGLHI

AAMHGHIQMVEILLGQGAEINATDRNGWTPLHCAAKAGHLEVVKLLCEAGASPKSETNYGCAAIWFAASE

GHNEVLRYLMNKEHDTYGLMEDKRFVYNLMVVSKNHNNKPIQEFVLVSPAPVDTAAKLSNIYIVLSTKEK

ERAKDLVAAGKQCEAMATELLALAAGSDSAGKILQATDKRNVEFLDVLIENEQKEVIAHTVVQRYLQELW

HGSLTWASWKILLLLVAFIVCPPVWIGFTFPMGHKFNKVPIIKFMSYLTSHIYLMIHLSIVGITPIYPVL

RLSLVPYWYEVGLLIWLSGLLLFELTNPSDKSGLGSIKVLVLLLGMAGVGVHVSAFLFVSKEYWPTLVYC

RNQCFALAFLLACVQILDFLSFHHLFGPWAIIIGDLLKDLARFLAVLAIFVFGFSMHIVALNQSFANFSP

EDLRSFEKKNRNRGYFSDGPMTPFLAFERLFFAVFGQTTTLDINPMRHLRPEWTEVLFKFVFGIYLLVSV

VVLINLLIAMMSDTYQRIQAQSDIEWKFGLSKLIRNMHRTTTAPSPLNLVTTWFMWIVEKVKARMKKKKR

PSLVQMMGIRQASPRTKAGAKWLSKIKKDSVALSQVHLSPLGSQASFSQANQNRIENVADWEAIAKKYRA

LVGDEEGGSLKDSDAESGSQEGSGGQQPPAQVGRRAIKATLADTTK

>NP_609561.2 polycystic kidney disease 2 [Drosophila melanogaster]

MAQQGQSGRSPSGPPGPPPPRPPKTPPGASPRGTPTADSTPGDVTPARGPPPPPATKRVSIGVGSTRSAP

PPATDPTGPPPTAGKPVRDTGPSTSPRPSLRDPGQSTSDKPSTSDKPSTSDKPSTSARTTVREPGPSSSP

SRPVSTRESGRITFQEAAPSTSAKVVKLAASEPRPAKPPEKEGFSLFKRKKKSVGSSTPPSPSRGHRAPS

IIAASALQNRAKTNRILYTTDEEVREALVEFSVFIIFLILTSLVVLSVRHTYMFYFNDTMKKLFTNREMV

VAPSVTVGFEKLITVPDWWDYLKYNFLVTLHGDLTFMDDAQANLTSMPPEGSEQGSEQGSEQGSEQSPEE

VVGEAPEEAPEMDRLQEDFKYNGNPYIDLRGHRRAKRQSGVDGNSEDGSEDVSGNYLKDDSDSIQANMSF

HHLEGRVFLYENLLLGPPRLRQIRVRKESCYVNDAFIRYFNTCYAAYSSGAEDRKPMHKGSPFRTMHDLD

STPIWTVLAFYRTGGYTVNLDYDKDRNVKIINDLKDIHWLDRGSRLCLVEFNLFNENTDIFQSIKLIAEI

PPTGGVIPQAHLQTVKMYSFFTDRSMLMTVIYIFWYIMVIYYTIYEITEIRKSGIKIYFCSMLNILDCAI

LLGCYLALVYNIWHSFKVMSLTARAHSDVTYQSLDVLCFWNIIYVDMMAILAFLVWIKIFKFISFNKTLV

QFTTTLKRCSKDLAGFSLMFGIVFLAYAQLGLLLFGTKHPDFRNFITSILTMIRMILGDFQYNLIEQANR

VLGPIYFLTYILLVFFILLNMFLAIIMETYNTVKGEITQGRSHLGSYIYRKLSGMLYWITHCGRKRRHHP

QASETEDKDAEHDVGAAHDETHEIRKNMTPAEQQYFKDIPQGENQDMVRLNNRVGLLEEILEKLINNMDD

ILKRVEKDYHNKKK

>NP_648696.2 nanchung, isoform A [Drosophila melanogaster]

MGNTESNVTSGVKKQAGVSTQALYKFVNLKGGGLLVDMMKRACQTKQFAEIDHAIKTKVEPFLYNKGAGR

YFPISKLVLLRNRDRPRTRQLPEIRALENPDDDFNIHDYCPEVSEAEYISNPTAYRFVCWDLNMRGAVGE

TILHLCLLNASSLHADLAKRLLKFYPKLILDIYMSDEYYGESVLHIAIVNEDPAMVKYLLDANADVQERC

CGAFMSAEDTKFSRTDSPDHEYVALCPMTNYDGYVYWGEYPLSFAACLSQEECFRLVLARGADPDFQDTN

GNTVLHMLVIYEKIEMFDVGYEVGTNIHIKNIQNLTPLTLAAKLGRVEMFFHVMSIEREIYWQLGSITCA

AYPLLMIDTINEETGNINKDSVLNFVVFGDKLEHLELLDGVVIDLLKTKWDTFCKSRFYKQFYMFALYFL

ISLFSFILRPGPDAKDEDEDGANSTTAKSDLYRQNGSDSYHLHSKRATMTTEYKTFWLNFTEYYDPSEVE

VLPAWWESYAQCPLMNLESDLAKLRIMAELLNFVGAILYLLVALREARFLGLKMFIENLMTAPSRVMFLF

SCALMMTIPWLRVSCLTEIDDHVTVVIMLTTAPYFLFFCRGFKTVGPFVVMIYRMVMGDLLRFVSIYLVF

VMGFSQAFYIIFLTFDNPSSPEDQDAESNPMPSPMESIVAMFLMSLTNFGDYYGAMVSTQHEYEAKILFF

LFMVIVSVLLVNMLIAMMGNTYQKIAEIRNEWQRQWARIVLVVERSVPPAERLKNFMQYSQSMSDGRRAL

VLRLNMTEEKEEMKEVQEMKRIHQRFAKKRQMEREARALRRQQEYEKFFGTAPKSECSDNNNF

>NP_572353.1 inactive [Drosophila melanogaster]

MKFLLKKCLRKKAPEMKPGAILDAVISQSSATACKCLLYKLADYKRGGDLIDAINSGGLIAVEQLIREQF

GVFMYNDGKGQVINRAEFLRWKYRDHTEVTIPIEASLSIHDPLGKWEDHKACWQMQYRGALGESLLHVLI

ICDSKVHTKLARVLLRVFPNLALDVMEGEEYLGASALHLSIAYSNNELVADLIEAGADIHQRAIGSFFLP

RDQQRANPAKSTDYEGLAYMGEYPLAWAACCANESVYNLLVDCGSDPDAQDSFGNMILHMVVVCDKLDMF

GYALRHPKTPAKNGIVNQTGLTPLTLACKLGRAEVFREMLELSAREFWRYSNITCSGYPLNALDTLLPDG

RTNWNSALFIILNGTKPEHLDMLDGGIIQRLLEEKWKTFAQNQFLKRLLILSTHLLCLSVSVYLRPAHDG

EAEDEDSEGSDASAAALLDIQSDEGDSGGGDYNAQTVARYCAEFATLVGVLSYVIFQQGDEIKNQGLSAF

LKQLSHAPAKAIFLFSNLLILACIPFRLIGDTDTEEAILIFAVPGSWFLLMFFAGAIRLTGPFVTMIYSM

ITGDMFTFGIIYCIVLCGFSQAFYFLYKGHPQVQSTMFNTYTSTWMALFQTTLGDYNYPDLNQTTYPNLS

KTVFVIFMIFVPILLLNMLIAMMGNTYVTVIEQSEKEWMKQWAKIVVTLERAVPQADAKGYLEAYSIPLG

PSDDSGFEVRGVMVIKSKSKTRAKQRKGAVSNWKRVGRVTLTALKKRGMTGEEMRRLMWGRASISSPVKV

TKQKLKDPYNLHTDSDFTNAMDMLTFASNPASSNGVTLRSVTAPPPAPPAPDPFRELIMMSDQRPETHDP

HYFAGLQQLANKAFDLVEQTMKTQPQAPVAKKVDPLPVASVAKASPAAPATQATATAAAASDLMAMPLPI

SNLSNLFQDPKDIVDPKKLEEFMAMLAEVETEESDSGGPILGKLSLAKRTHNALSKAEIRRDQQGFEGHS

HGQFQPMSSVWAPPGLDVDTGFHFDEAVAEEVLTIEQEAEVETEDGNGGQDSEDIPTAEEVHATMKQFHL

RKCQPAQDEAARRAKSARVRRRNKVSPEQSDDPDERSQRGRSAYTRRTQSPPDPLEPWSTRELQDINKIL

ARK

>QDR51043.1 TRPA5 [Manduca sexta]

MCTRGLISTLFPRDVETGITEEGEDDENRRPLLGDGDGSSISRARPPRPRHLGLVNTTQNARQLPKLTLP

DMTPSSVSSTVHNTPVGESASTMSKPWRRGDRRLRRLNTELLEAIEGHDVEQVEKLLNSGANPNATCRLD

LVSACHMAALIGGDALGLLVKFGAEKLRIDRLGRTPLHLAALAGNARQVAILLDFPEDMQDRVGDESMSS

ETEDDVRKLCPYIREMTNVRCDLGEVNAALPKTWKDNIDHECRDIKGSLSEMFQPGWTPLHVASSCIRRH

CTRLLLAAGADPNICDAEGRTALDVAGSAFYYNKEISPKHLPEVIKMLIKAGGKYNTMNKRAPAFMDTPL

HTAVELENLEAIRELLDAGASINRLNRAGLTPLHVCVKKQLEEHLQLLANYDYINMDPMAAVVDVKDKEG

HTVLQAAVEAAWVPGVCIALEAGADVTLKANDGETPIHSAAALGNIDVLNEILSVAKKKDALDYQNEEGE

TALYKAIIHGRLECVKRILDEGASMKITLPGDVNVLHVAADYGELEILKVLLEYDDAMTDAMINALSADD

RRGFGPIHFAVSNNHAECVKLLLSKKADVRLRTTCNPHKASTPLHIAAVKNHVNIAKILVQFDKTIIHEV

NSLGWFPIHTASHHGCRDVITLLLKEGADLSGYTDGPKKFKRTAIDMIINNLPKPTDFMEEVFDSYISSN

GQDLQDPSCVITVDFSILMPNVCEMEQMKVIEALLKTGNRYSQKRLLVHPLVESFLYLKWKALLPFFYAI

IGLYGFFVTSLTVFTVSVFFYRDTNDKTPDWLNSTTWSYVVYVTILLILLQELLYMNVKSSRYFLQLETW

VKFGSLGLAVILPYAVVVVPLNEADWPRHVATLALLLSWLEMMFLLSRFPNWGYYVLMFGKVASNVVKIL

LTFAFLVIGFSLSFMIQFRSQIPFEGPWAALVKTIVMMTSEFDYVALFDEEHSKELATSLIVVRLIFLIF

LILAAIVLMNLLVGVAVNDINDLEVLGNIRRLAKQVEFLSTLDTLVYNKLFNKILPKKVNSRLKNKRKVN

NIMTLSPGKPRWKYSKVLPSRLKDAIFNTAQFQKKQMDDEKDFQRFNNMMDEIHEIIVKEKKDGDKQMET

PDLIKDRLVKRGDDITKRLGEIDDAVVEIKNQTKSYVEESKSSVEMLNVKVDQMSLEIEVIKQLLSRIES

KIGR

>QDR51038.1 transient receptor potential cation channel A1 [Manduca sexta]

MLGRSSVDNTLIEEMFKAAESGNVEDFTRLYLSEPGRLGVRDGRGRTATHQAAAKNNTNILHYIKNYGGD

LNALDNSKNTPLHVAVENEALGAIEFLLQNRVETGLLNEKSQAPIHLATELNKVEVLQIFAKYKNDFNVN

LGGEHGRTALHLAAIHDHEICAKILITDLGAEWKKPCNNGYYPIHEAAKNASSRTMEVFLQWGESKGCTR

ESMISLYDNEGNVPLHSAVHGGDIKAVELCLRSGAKISKQQHDLSTPVHLACAQGALDIVKLMFNMQPSE

KMACLTSCDVQKMTPIHCAAMFDHPDIVNYLISEGSDINPLDKEKRSPLLLAASRAGWRTVHTLIRLGAD

IQLKDINSRNVLHLVVINGGRLEEFAANCKDRCEKSLAQLLNEKDNTGCSPLHYASREGHIRSLENLIRL

GACINLKNNNNESPLHFAARYGRYNTACQLLDSDKGTFIINESDGEGLTPLHIASREGHTRVVHLLLNRG

ALLHRDHNGRNPLHLAAMSGYTQTIELLHSVHSHLLDQVDKDGNTPLHLATMENKPSSIALLLSMGCRLR

YNKQDMSAIDYAIYYKFPESALAMVTHEQRAQEVMNLRSDRYPCVTLALIANMPRVFEAVQDKCITKANC

KKDSKSFYIKYSFDALCPQLKDDDGANVPQSQKIPLPALNIKYSFMYYQYSKLELEALRQAQNDPTYRPD

PLSVINAMVVHGRVELLAHPLSQKYLQMKWNSYGKYFHLANLLFYCIFLIFVTVYTYLLMTSVDVAVMTP

KKQKNNLNPTTLGPEFNESIGNITANITAANVEFESKVAMYTSTVAILVYNLLCLMREAYNLKQQKWHYL

VDPSNLVSWTLYISSTLTVLPTIYGNYDNLQYSAAAITVFLAWFELLLLLQRFDQVGIYVVMFLEILQTL

IKVLMVFSILIIAFGLAFYILLSKVGGYHLSFNSIPLSLMRTFAMMLGELDFVGTYVQPYYKADSDTTLP

FPVTTFFILGTFMVLMPILLMNLLIGLAVGDIESVRRNAQLKRLAMQVVLHTELERKLPAFILEKVDKNE

LIEYPNNKNCKLGFLDLILRKWFCNPFSDDVGLDLVLESNEDYITAELDKQKRRLREMSQLLEQQHTLVR

LIVQKMEIKTEADDVDEGVSLADTRVVPRWSSPRTRNNLRTAQSVNKGG

>QDR51040.1 pain trpa [Manduca sexta]

MAHNSYEMKSNRLSRGGSLFGADPQVQLKLALQNNDFATFKKLVSYGAVDFEYVYQYPDHKTCLEMAVSE

PNRQEFVKLLLQHEVEVNKVNETYAAAPIHFAVENGNKEALATLLEDDRIDVNIKSKSNTALLMAIKKIE

ELDDDRENDLVTYEDMVELLLKAGCNPNSPDLKGVTPVYSAAKQGLERVITLILDYSRYPVDIDTYKDRR

GKTARHYIKEAFPHLEAKFESNTADTEIVDADILFSFLSRHEEDNFIRNFTKLANKNDHRSVLAANNGFH

TMLQFATEKGFDRAIRVLLDHGADPNATCSSNTSRPVAIACQNGYFIILKMFIDNESTLFEPVNSESLLQ

VTIKGMRSYTESPKVNFKECLKLLLKHPKININVNHLDMKDNTALHYAARNGDNDTVLELLRKGACVGLR

NKFNEPPLADINAKTLETYLDECITTNSERPSDDDYEIHMKYSFLVYPNNSLENEISKVPLMDNTNNNVK

EYDAILAPETDALLYMTRNEDLRPLLKHPVITSFLYLKWQRISCLFYANITFYSFLWLCLILYIILGYGV

EKKQADSIEALNIITHVGAVIGLILLIFREVFQLLVSPTRYLQSIENWMEIALIFVTTWIVTHDSAAEST

KQQLSAVAILLSSAELVLLIGQFPTLSTNIVMLKTVSWNFFKFLLWYCILIIAFALSFYTLFRQEENEDQ

KVPDPNNTGKEEEEEDFFEDPGRSLFKTIVMLTGEFDAGSIKFSTFPVTSHVIFIVFVFMIPIVLFNLLN

GLAVSDTQEIRADAELVGHISRVKLISYFESVLIGNARAFTKPSRGWSWLPSNLQNLNIIKPKMFFIKPF

AKRISLFPHFLPKYRILVKPNQSNKIEIPHAEPLGKFGDDYEDVEGGGCCFERCQNYKLDRMIVKRAKLV

ISKRTNISDIVEIKEQLCQVEMKIEGLESTLKKVLQALESDRKY

>QDR51046.1 Pyrexia trpa [Manduca sexta]

MIRTHAQRSRNVSHGDVPKNEKKKKVYREGSARWYAGISELAEDAEDDAEAGYISSDESAHGVDLAERLR

VKPSKDIWELDEIQYTLRQLPHFEEILSLMDTGHYDEVISYATNTNGGVGLRSAALWACWLGRSTLLTKL

LKLGIDPDETDDAGRTCLHLSCLVGSEVCAKVLLDHGAHPNRWDSSTDTKATPLHCAASAKSLACVKVLI

AHGADVNAGLSDRSPLHYAVLSDAPEVVKELLEAGACPDTPQVFTETPLHVAASLGSASCTKLLLDAGAD

VRAAVGPGRSTALHLAAEDGHAECARLLLEHGAHIDWPNFRGQTPLHLATLAQSLEVVEMLVSKCANVRA

RDADGRTPLHGAIVRGARACDVARLLLSAGADPNAPDNFGYTPLHIAALNEFSACVLLLLDYGGDVTLRT

NGGVSALSFIVRRVPDVIPRYLCKFDDAVHVSEHELGDVDCELTIDFRPLVPCLTRGEAELLLAFVEVGR

KDVLKHPVAETFLFLKWRRIRKFFVLSFIYHALFITLYSLYILQIFLCEDVTCNVPSYLRPMQYLILLLN

LCFMLKEVFQACQDWSAYIRQWENWLQCFIIIGVFLCTIPYWDIDNGVIRTDTTDWQHDVAAITIFFCWL

ELMMIIGRFPTFGLYVQMFTTVTVNFATFLLAYSCLLIAFGLAFSVLFSNYPAFHLPAGLVKTVIMMSGE

LEYEDIFYSNSSSSQIQYPLTAHGMFLIFVVLVTVILTNLLVGLAVSDIQALQESAGLDRLVRQTELIAH

LEGMLFSSLLSCAPKQLLAVLRWGALLTASQMRTLNIRPNDPRESRIPKELISSIYKMVASRKDTKRSIR

KNNNYEFRLMKCKDEEDKEQKQDVVKRKSNLYDRYSVESQNVERRKRTTSGSNADKNRPPSLTINAIQEI

CMVDIKTQLLELTKKVNKLVEITDARLNQIESKLDVQPP

>QDR51041.1 water witch trpa [Manduca sexta]

MLHSSNYLSLIDIAIACHLKCNVRARPIFSRHDVLIVHQVCRQLNSRRCARGHKWRTIAASHQSAGVSRV

SRVCAARMPAPRLLPARWWRRREHEAPRSLPHLPHPPHPPRDMERRPRASRVPSSPARQHRPHHPPRDDD

ELERAYPSMGQLEYVLAGSSPPAESAPNMYDSFEEPPLDLTAHICGDSLRQSAHEQMRAVGGRLRLLDEL

ETGAITADTAANTFATASEAEKNVCLFWAAFLRLANLLPILVDAGADPLYFDALGLSPLHVAAFSGSIEC

ANYLLSCGADPNYMARCFAPLHCAAFGNSVQVANLLISRGASVHAAVKYVNCEGGLLHCAVRANSVECLK

LFISHGVDVNLIEPGGTNAIHLAADLGMIQCLAILLETPGADANVRTRVGDRESTALHLAADGGFVECVD

LLLSKGADASLKNHRGFTALHLAARSSSLECVESLLRKGNAEPNAMDFDKRTPLHAAIGKSDSACDIIET

LISWGANVNQKDEYGFTPLHLAALDGLSACVETLIYHGADVTTRSKKGNSALNVIARKTPASLAMITRKL

DCAITLHHSQTSNREVELELDFRSILQHCYPREISYLNTFVDEGQKEVLLHPLCSAFLYIKWEKIRKYYV

ARLFLSFIFVLCLTLYVLTALAHNCYNGSKDMEETIQEQELCQKQSILGDLLRKNPFVIEMQWWVLAGIT

IFEIFRKVYGIAGYSTVKQYLMQSENIIEWFVIVSVFLISYIYTNITYAWQNHVGAFAVLAGWTNLMMMI

GQLPVFGTYVAMYQKVQKEFAKLLMAYSCILIGFTISFCVIFPDSSSFANPFMGFITVLTMMIGELNLDL

LLNEPDGNDPPVLLEISAQITYVLFLMFVTVVLMNLLVGIAVHDIQGLRKTAGLSKLVRQTKLISYMELA

LFNGYLPKCLLKILHSSALVSPQAYRVVLSVKPLNPSEKRLPRDIMMAAYDIAKMRKQYGHTISSSGSTT

GAYSCFKKYENNNDSGYREYGYSGLGSLHARLDETSENVRQLTQEVRELKKLISAQQLVIQQALAGAMDR

>QDR51042.1 water witch 2 trpa, partial [Manduca sexta]

TEISHVSAHSNKTDKITPCTTSPLSRDLTMDNFGYREMGWPSPLQTPAQTPTRSRLTSSVRSRVREPEEY

PLNSAYRHVKNIDLLESAREPGKAKEYLFVGPSPPAEDAPNMYHSFDVLAPDPEARLTEEAVRRGLCERA

LTLGAARFFDDVECGLITAENIEEHISSAPEVVVNLTLLWAAFLTRDELLPAILEAGADIHYSESPGLTA

LHMAAFSGAGRAAIFLISRGADVDFAPKYFTPLHCAAFGNSLEVAEILIANEASLHSVVQRAGCEDNLVH

CAVRADAVECMEMFIERGVDPSYVTSGGLNALHLAAEIGAQRCLSYLLKETKISVNGVTKQRDKECTALH

LAAARGYAECVELLLSEGAKANTKNYRGFTALHLASRCSSIECVEVLLRDGNADPNAEDYDKRTPLHAAI

CRADRACDIIDMLVSWGAQVNKKDEYGYSALHLAAMDGLTQCVETLIFLGADVTSKSKKGHTALSVIARK

TPKSLAILKYNLDCGISLSRSTEGNEEVQIEFDFGKLLKFSYPREITYLNSLIDEGQKDILLHPLCSAFL

FMKWRKIRKFYLARLIFCFLFVSFLSIYVLTAVVKTCKGKYSKKYGVPNELCQAQSLLGIMLENNPIEFE

RWVLMAITAFEIVRKLTGITGYSSLYQYFTTFENLMEWFVLLSVFSLYNIHRDYSWQNHVGGYAVLGAWT

NLMLMMGQLPMFGDYVAMYQKVLTEFLKLLLAYICLLLGFTICFCVVFPNEEMFSNPLMGFISTLSMMVG

ELNLNILINDPMQDDPPLIFELSSQIIFILFLLFVTVILMNLLVGIAVHDIQGLRKTAGLSKLVRQTKLI

LFVEMGMFSAWLPKCLHKYVYRTALVSPEAGKVILSVKPLNPREKRLPTDIMMAAYELAQINKVKSGRSV

KEILYKNKFSSKLKNEGQSNDHNFNIEIRGMQEKIDQATFNLKKIDQEMRHLNTLLMEQQTMLQNVFKYS

EVVPYRSSYATTPIYPESPVFFSSNTSESNK

>QDR51030.1 transient receptor potential [Manduca sexta]

MNRGSREELLGSSQEALRNSQHDLRHVFERPLTREEKTYLLHADRGDYVTVKRLIEQFAGRSDVLDINCV

DPLNRSALIAAIENENIELIKLLLGSGIKVKDALLHAIKEEYVEAVELLLQWEEDNHVPGEPYSWESVDP

AGATFTPDITPLILAAHRNHYEILKILLDRGATLPVPHDVKCGCDECVKSSQEDSLRHSQARINAYRALS

SPSLIALSSADPLLTAFQLSWELGRLSRMETEFRVEYKALRQQCQEFATSLLDHTRTSKELEIILNYNPW

DVDCWEPGERQTLGRLKLAIKYKQKMFVAHPNVQQLLGAIWYEGLPGFKRKNIIGQCFQVAKLGVMFPVY

CTIYMLVPNSEYGRFMKKPFVKFICHSSSYMLFLSLLSLASQRAEYVVLEWLGIRWLQDLVDYWKEHERG

SLPGPIEFSVIIYIASLIWAEIRALWTGGITEYISDLWNIVDFITNMFYIAWISLRISSWYIVQRDHKNG

LDPWYPRERWDSYDPMLLSEGAFAAGMIFSFLKLVHIFSINPYLGPLQVSLGRMILDILKFFFVYMLVLF

AFGCGLNQLMWYYAELEKDKCYHLPNGMPDFDGQERACTIWRRYANLFETSQSLFWASFGLVDLTTFELT

GIKSFTRFWALLTFGSYSVINIIVLLNMLIAMMSNSYQIISERSDTEWKFARSNLWMSYFEDGDTVPPPF

NIIPTPKHLSCWIKHCFTKRDRGSIINKSREKARIEHEAVMRVLVRRYVTAEQRARDEVGVTEDDVMEIR

QDISTLRYELIDILHNNGMKTPRVSLQDATSTXLSGKKGKVMERRILKDFQIGLIEGIIKDVISRENKPK

DVFSQIAKAIVRRGSTDSKKRDWNALVRTNTVRRDPIGTAAEAEIRRSRQSLRAHILENVEARTIDPQKL

LEYNPKLSEVTPTTRVAYAKFMRSKIKSDFAERERNRKISDEISLEDEEFESPKKRECTHRKMTGRTFRS

RTPIPETSDDEGLKDNEIKVLAYIEDKGSEVSSEQTRPLKSSSETSEPRTPDFDPVNDRKLPPPAEFHVQ

IEPDTPTHSSAEESSVQDLITPTGSLKSHKEQILSATALDSQYKQMISPSPDFDMKQSASVFLESEKSSP

VKSSYLEPFDSSKSPSSSIKSTKTDSKFVSASLETQMAPSRLSVASESRSPDKRLSTSSSPKSTSSRSSR

VSPSSPTPSMGAVDRGKSKVTGKFVSGWL

>QDR51033.1 transient receptor potential-like protein [Manduca sexta]

MAEKKDLEAGDPELECVRKPMKLPELPKPLTFEEKKYLLAVERGDMGNVKRILQKAHRKKHIEINCVDSL

GRGALTLAIDGENLEMVELLVIMGVDTKDALLQAINAEFVEAVELLLEHEELIHKEGEPYSWQKVDPNTA

MFTPDITPLMLAAHKNNYEIIKILLDRGATLPNPHDIRCPCEECIRESTEDSLRHSLARLNEYRALASPS

LIALSSTDPILTAFQLSWELRNLAFAEQESKAEYLELRRQAQLFAVDLLDQSRSSQELAIILNHDPDEPA

FVDGDHMKLARLELAIDFKQKKFVAHPNIQQLLASIWYEGVPGFRRKTTMEKIMIICRVALLFPFYCTLY

MIAPNCATGKLMRKPFMKFLIHASSYLFFLLILILVSQRAEVQAIQLFGPEWMVKELEKELLKQRGNGPT

YLELVVVVYVLGFIWEETQEIYIEGIRSYLRNMWNFIDFTRNSLYVAVAVLRFAAYIQQTSEIRRDQQTK

FIPRESWDAFDPQLIAEGLFAAANIFSALKLVHLFSINPHLGPLQISLGRMVIDIVKFFFIYSLVLFAFA

CGLNQLLWYFADLEKRKCYVLPGGLPDWDNAGDSCMKWRSFGNVFEASQSLFWASFGMVGLENFELAGIK

SYTRFWGLLMFGSYSVINVIVLLNLLIAMMSNSYAMIDEHSDVEWKFARTRLWMSYFEESATLPPPFNIM

PTPKLLLKMLGLRKKDKLRKMKLKEQKEKEHDVRYTAVMRALVWRYVSAMHRKMDDEPVTEDDVNELKGD

VSALRYELLEVFERNGMDVSFTDKKEKTVLGSKQKVWERRLMKDFHIAVAGLEEDKPANEDGLSRFRRIA

KMAIANTTNAKWNETLVRSGVATQIGRCRSRESFKNQQNLQRAMEEARKLVMRSPLPGSSRAASPIEMPV

TPGHTLLNLIKDISTEVGEPVDISVTPGVMSPAKPPLVEGVAGLATSALLSAASAVPIRPISPKPRSPSP

APPKPASKAGTPLPPSRGASPVPKPSSPVGDGTTSPQPEPSPLKAKRVPGESPPKVIKRKAPGPATQADD

QAKDAAPEGDKDGKPSEDIAVARPVAPEIKPSEGAIPPPPAPVKPKAPVAPSMEVTPSTPPGHAPKTAPT

PPPPAPPAAAKPPSPPKAASPKPASPKAEAPQPSTSAAPRPPTPSSPPNRLTSTSSTEQLIPPSSPEPLR

PIKKIDEVKTIKRQPKTGWL

>QDR51034.1 transient receptor potential melastatin [Manduca sexta]

MSIQKCLLDNSSRMIKAMSTAKSNNNEAKTKTTRSWIETTFQKRECKCFVASAKDEHVCWCGLSRTAHGA

NIATATPGEAWVPARHTQPAPTDAYGTVEFQGGPHPTKAQYVRLSHDTRPELIVQLLTREWALDRPKLLI

TIQGGKANFDLQPKLKKVLRKGLLKAAKTTGAWIFTGGTNTGVTRQVGDALQLERSQRAGRVVSIGIAPW

GIVEGANELIGKGRDVPYHAIASPRSKLAVLNNRHAYFLLVDNGSVGRYGAEIVLRRKLEKYIAAQKLHP

YTHSSTPVVCLVIEGGTNTVRAVLEYVTDTPPVPVVVCDGSGRAADLIAFVHKYASESGEQTVLESMRDY

LIGTIQKTFEVGLQQAECLYSELLQCTRKKNLITVFRIQERAEGGEVQELDQVILTALFRAQHLTPSEQL

SLALTWNRVDIARSEIFVYGQEWPPGALDEAMMQALEHDRIDFVKLLLENGVSMRKFLTIPRLEELYNTK

SGPSNTLRYILRDVRPHLPKGYVYTLHDIGLVINKLIGGAYRCYYTRRKFRPIYAKVMNKSVNVHRNSAS

FTRHTAGGLSLITGFMPVTSEMALFDYPFNELLMWAVLTKRHQMALLMWTHGEEALAKSLIACKLYKAMA

HEAAEDDMETEVYEELRHYGKEFENKALELLDYCYRQDDDQAQQLLTCELQNWSGQTCLSLAVTANHRAL

LAHPCSQIILADLWMGGLRTRKNTNLKVILSLLCPLYILRLEFKSKEELQLMPQTEEEHLENESIEDDRS

TKDPTDAEVGVVKRRXXSIKKIIPXGQQNKNKALIGSGAECETRVDQNGKVGKVGWEPSYRELPEHHAPE

IKRMRPLRLRKKIYEFFTAPITKFWADSXSTIVLVLMFTYTVLVKMDPTPSWPEIYSICYILTFLCEKIR

EIVTSEPVAIRHKFSVWAWNMWNTYDAGFIIFFLVGLTLRLRLGSMDVGRVIYCVDIIYWYLRILNILGV

NKYLGPLVTMMGKMVKNMIYFVVLLLVVLMSFGVARQAILYPEQEASWYLIRDVFYQPYFMLYGEVFASD

IDPECGKEPGAKKCVTGRWITPIAMTVYLLVANILLINLLIAVFNNKFNEVNEVSHQVWMFQRFTVVMEY

EQKPVLPPPLIIFCHIYAVGKWVTRNVSHKRFQYDNGLKLFLEKEDMERLYDFEEECVEGYFREQEVKLS

NSIEERVRNTTDRVEHITTKMEDLNQKANCQIQSVQSVEFRLRKLEDIAEQTMNHLAVIHRFMATHPSLV

NRGSTDTLGPPPPTFQAGGNRLRTASDRSEVLSDSDTGAARFTVRPVQEEDERASQPSDDELPQSGPEGG

PEKEPEADSISTSSAGSAVGPDVTVVRPQPSITPARQRSSDSNRTEPSDEKKDLSQAAPSTIDDAFLPEV

REMPTDLQSLRPRTTSAGTRRTANTRRRSEGGNDCGMGGHYSSQHLAPRRQHSQTHSEPDNSAVDAGSVH

NSVTGRQSGWEATGGATGIPSGRSRVGGAPRSLLLAMHEYTSITDELETVYGLFSPPHTPRTPITSRLLS

PARAASPSIRRRHASEMSNPEFALFMEKEHLRGAEEDDYIIMENLIQRRLELGSVLDRYDEEEAGDGAAD

DRGEVSGISISVCVNNSGTDEPMPPQTTTLLTVSDRRLPHQRHSLRRSSAIDSRELPSPLQPLLGEDSCG

EVLLPVPRQPSGDTNASSELSLSHLLSESLPPRPSIVLDSSQLPRQRSAEQPLKPQPPAPARPETMC

>QDR51039.1 Transient receptor potential cation channel, mucolipin [Manduca sexta]

MEGITPSPEDTTCSEGEEEQKIRVNLPTRENGQGYSQQSTSYNTLHQMEEKMRRKLQFFFMNPIEKWQAK

RKFPYKFVVQVIKIVLVTFQLCLFAHNRYNHVNYTWDNRISFSHLFLQGWDSTREINAYPPGAGPLAVYK

IDEFYSTLDFAYAGYSNLSNAIGPYSYNDESNNMPDPVFCQYNYKQGDIFGFNESYQFNSEIVETCINFT

RKGNEVFDSELFIKEAGLDINFSALIRAKLMFSIKTINFRAAGPITPPDCYRFDVEIIFDNEDHDGQMSL

ILEAEPYKLECKGDTAYITDNKIDQILRSTLNILVILICAVSFVLCSRAIYRAQLLKELTVQFFQRTYNK

DLSLDGRLEFLNVWYIMIIVNDLLIIMGSAIKEQIERNQFTNDQWNVCSLFLGTGNLLVWFGVLRYLGFF

KTYNVVILTLKKAAPKIFRFSICALLLYAGFMFCGWLILGPYHMKFRSLATTSECLFSLINGDDMFATFS

IMSKKSPMLWWFSRVYLYSFISLYIYVVLSLFISVIMDAYDTIKQYYKDGFPKSDLQQFIGEANIEEVSS

GLYRTQSSTSLNAIMNSLCCCNVYRSAYAKIGSGGSNINLL

>QDR51035.1 no mechanoreceptor potential C [Manduca sexta]

MSEGGRDQPRSANLNISSGVRSPPANQQKDIQKLELSFIELNKERAKQKEWKKLIRDLVFQRGKIPLLLA

VEAGNQSMVRELLSAQTAEQLRASTPAGDTALHLAARRRDVDMARILVDYGAVVDATNGAGQTALHIAAA

EGDEPLVKYFYGVRANAAIADNEDRTPMHLAAENGHAAIIELLADKFKASIFERTKDGSTLMHIASLNGH

ADCAMMLFKKGVYLHMPNKDGARSIHTAARYGHVGIINTLLQKGESVDVTTNDNYTALHIAVESCKPAVV

ETLLGYGADVHIRGGKQRETPLHIAARIPDGDKCALMLLKSGAGPNKATEDGMTPVHVAAKFGNLATLIL

LLEDGGDPLRKTKSGETPLHMACRSCKPDVVRHLIEFIKEHKGENIAIAYIDAVDDDGASALHFACKITK

EEVKIPTADRQVVKCLIENGADVSLQTRHNHETAFHFCAVAGNNDVMTEMITHISTADVSRALNRQNSIG

WTPLLIACNRGHMELVSTLLSNHARVDVFDVEGRSALHLAAEHGYLQVCDALLTNKAFINSKARNGRTAL

HLAAMNGFAHLVKFLIRDHNAVIDVLTLKKQTPLHLAAASGQMEVCKLLLELGANIDATDELGQKPIHAA

AQSNYSEVVQLFLQQHPNLVMATTKDGNTCAHIAAIQGSVKVIEELMKFDRTGVISARNKLNDSTPLQLA

AEGGHADVVKVLVRAGASCTEENRAGLTAVHLAAEHGHTNVLDVMRSTNTLRISSKKLGLTPLHIAAYYG

QAETVRELLSHVPGTVKSEAPSGVSLVPELGAESGLTPLHLAAYNGNENVVRLLLNSAGVQVDAASNENG

YNPLHLACFGGHMSIVGLLLSRSAELLQSTDRHGKTGLHIASTHGHYQMVEVLLGQGAEINATDKNGWTP

LHCAAKAGHLNVVKLLCESGASPKSETNLNCAPIWFAASENHNDVLEYLLHKEHDTQSLMDDKRFVYNLM

VCSKNHNNIPIEEFVLVSPAPVDTAAKLSNIYINLSTKEKERAKDLIAAGKQCEAMATELLALAAGANSA

GHILTATDNRNIEFLDVLIENEQKEVIAHTVVQRYLQELWRGSLKWTGIKIMFLFVAFIICPPVWVVFSL

PLGHKYNKIPIIKFMSYLTAHIYLMILLALVAITPIYNSIFRESLVPRWYEWMLLISLSGLLLFELTNPS

DKSGLGWIKIAVLLIGMIGVATHVIGWIFVLPKYWPTLMYCRNQCFALSFLLACVQIMDFLSFHHLFGPW

AIIIGDLMKDLGRFLAVLAIFVFGFSMHIVALNQPFRNINKAEDNKYARQARRKLFSDVTMNPLYSFELL

FFAVFGQTTTEQTRVHRNDSNTQPAWTNYLFKIVFGIYMLVSVVVLINLLIAMMSDTYQRIQAQSDIEWK

YGLSKLIRNMHRTNTAPSPLNLVTTWLMWLIARCRERLSKKKRPSLVHMIGLQRQDQMSARSKAGAKWLS

KVKRGQVVPKDSTRLSVVHLSPLGSQLSFNNATRIENVVDWEVIAKKYRALTRDEPEEPQAVENEADTAD

DVSEIVPNNISAAPP

>jgi|Helro1|172113|fgenesh4_pg.C_scaffold_22000277(130200)

MAQNVSNCNPSDRQTHRKSLSNQITRYTKFPYNKNIINNNNSKNINNNKINNNFFDKALSTLTDLNISNL

ENSSSYNRRALPRSSSSTARGCLFTNFEDVQLTDEERVYLNAAALGDVPILRQSLEDIDVPLNLNCVDYM

GRSALHLAVDNDNMESIELLLDRLQFECLEESLLHAISKGSTKIVRTIIEHPSYIAGEESLKKRHKDPFF

RTDEKSQFSPDITPLILAAHYNNHEIIQMFLSRNHTIVKPHHISCRCSECVTKRGYDSLKRSRSRLNAYR

ALASPAYMALSSPDPIMTTFELRQEMKKLAQVEKEFKSEYLSLVEQCMDFACELMDLCRGTQEVEAVLSE

GSQENGSRDPLARLKMAMQYEEKKFVAHPNCQQHLTSIWYGPETGFMQSLTLWKKSLMWVVCVPLVPVFC

FVYILSPDNKIGNAMRCPASKFITHTLSHVCFLILLAAATFRLDEKFLPIPSTRSSTSDWFENRSLGQFG

DQSRFLNGEMSHAEIVESLLKGTFRPANILITNVQICLMFWVLGLLWMECKQMHTSGLRIYLLDDYNIID

FIVLSLYLASYTLRFLVDHRIKSVDKWYNQGTSRARHALLANDTKLYDSIKNEIFSDPLSYFMNASRFYW

KPNDPEIVSDVLFAVANVVSIARTTYLMPAFEVLGPLQISLGRMIGDITRFLVLFTLVLLAFMVGIHNLY

WYYGSQTFLVNKDGEQKLMHTSEAFQGMPRTLLSLFWSMFGLVSVDSVEIKHPSKEKGEKFVGSFPPGSH

ATSIVEGVGHFLFALYHVAIIIVLTNMLIAMMSHSFEAIQGDCDVEWKFARTRLWVNYIDEGNTLPVPFN

MIPTPKSLAYIWQWVKELFKSQRELMLKELGEAAKKQTFIKSRREMVCQSLSIESPDTTYSDILQRLVRR

YLFKLERLKEEGKDKAGIGSEAAQPQSADMFNQPEPAFADEADESDLSPFSAESLARESNQDTYPKPTLT

ASTTTTQLSACGSNQQLQNANPPRYETHSNYQQPQEHDASQIRSPAANSPQPINDFRLSLCQPSTATASL

TTTSATTTPATTKQPTSILRSRYSNQCRMRRTSLLPGSNQPPSGQNPGAPTANTVVQLISLPQIDAIQRN

LKLLDVRLQHVQASAKQEDHSRQDIDHIRMVMSENQKALSTVVTVLSSIQEEVRTLSVAFNRQQQQHIQF

GLQFPLLTKTKSTQEKQCGNANTANFGNTDKTVPDNQTSYTAPVHTSSSSSSSLFYFSNPFSTTATAAQN

NTSLTNRPPSLAVTSTSGKLNFPREKRRASLRAEKTLM

>Helro_190762

MSYVTLRNLLIAGKDGIELPSIAQGLLNDPVPEHVDRRLFEYIVKNFEKVECGLFRSTAQENVCECGNIF

ANHTEEAKAAGKLFSSSPSNSLQLETKVSSPTDAFGEINFGGRTNSSYVRLSSSTEASAVLELMRKYWTM

DKPNLLLSVTGGAQNFTMKPRLKEVFRQGFIKAAQSTGAWIITGGMNAGVMKHVGEAVRDYTLKHGRIAP

WGCILNRESLMSSTGTGCWPANYSSVASSVTKECPLDPNHSHFILVDDSTSGKYGSEITLRAELERAVSN

SPTYLEFDSDESVAIPIVSIVLEGGPNSIETVKNAIACGTPAIIVQGSGRVADILAHAYNNAVEVEIVAE

DQLGKKHKQLISIVEQWLEKELMDKIKSELKTKSDEQAKQILENVKICVSNRDFMTVFDLDGKDCGSDID

ITILKALLKSNKGKKIDQLKLALAWNRADVAREQIFTDDAIFEIRKAVAAPLPMDDASLLGFFNGVIYTS

LLAPPEDLYDLMYKALIQNKVEFVKLLISRGVSLKEFATVPRILSLLNEPLTRRNCKHLFNISESTLVSL

KHVGQLVQDLMGHYYEHSFCKPPYKDIEASDYVKTSNIHAKKDNPLKSLKIPFNTLRSGKSDKRLPLPEF

EDPSNVLFLWCVLVHMKDMSLVFLDIGKEDIAMALMAQHISKSLLQKEQDSDFAVNIQNQIEYFGNLALG

VLNECYSVDQSKATLLLVRQMSSFGETTVLKMAVQADHKEFVAHPACQNLLTNIWTDQLSDDNNYIWIIL

ALLMPFLSPILLNFRQKEIRLNNAEDAKEKALTRMFLNKKRHNSDARREKNVAATHILIQQSKEKTQQKA

QERNFVLEYWDKLESFFKAPFTTFCYNCIAYFAFLLLFGVILLTNFFPVYGKQSNQVIPIEEIILIAWVS

TFLLEEIRQFIVTESSHPGQKLFNYISDPWNVSDIISIATFYIGLTLRFIPTSTCERCFYAGRIVLAFNL

MMFFLRVLHMFSVHQQLGPKLVMIKQMMVDLFYFVIIIMVFISSYGITSQVILYPNQELNIKLARDVLSD

AWWNVYGQLDIDMVSDGKCSSKPDVCDFNSTTGKCKALDCAVYGYESDKCYGKEDNCVANDWVVPILLAI

YALITNVMMLNLLIAMFSNTFAAVQGQSNVVWKMQRYHLIQEFESRPIWPPPLIIIGHVYQFIRYMVFDR

CCRNQKSTKIGFRMKLNKVEEKELINWENIYSEEYLLNNETTAESSMEFKINETFKKSDDVMFKLDQLSE

SSLFSQTTSLKDAAQTKLPPLLEKRFAQIEEQLKQNAQALTLLMDHLKISREAPEEVKLADVTGKEDVER

RQVEIKKKKELETERLKEELKQHKLSRSAVYPVMFEAVVRFPVPDNKVSWKVAYPSYKPPSYTSDEVLDG

NYDEENMDHISFNAVDQMRRYDRRSCTGRYDIVDGFPLNPRGRTGLKGRGSLNRWGPNHIYKLLLTRWAD

SEPTSIKPKKYLQFLAVEFLAPGHKIIIPELLYPETSNPTMECLQDLPDAVELKRWLREEGDHVTHLFED

RQGSRDYYDDPRNTDNAWLEVSADIYHDEDVIVLTNEQLGDPQTIKDDKKFTWVRIHRGMDVYAADLPIL

KTAVNKLKASF

>Helro_191443

MAHFNPNAEDELLTDEERVYLLAASQGDMAIVRQSVVGEIDVDEKLGSSDDLGKNEKEFIKFVNNKRSVK

GLKKRNLDVNCVDYMGRSALHFAVDGENIEMIDFLMEFLSLELIDEALLHAVSKDLAKIVRQIIEHPTYE

SRNTNYPLRKFHNSFKKLHLRAEKSGQFSPDVSPLILAAHRNNHEIIQLFLSKQQTIAKPHSISCHCCDC

TAKQNCDSLKRSISRLNAYKALASPAYMALSSQDPIMTTFQLRQEMKKLAQVEKEFKNEYMGLVEQCMNF

ACELMDLCRGTQEVEAVLNEGCDDRGDPLIRLKMAVEYEEKKFVAHANCQQHLTTIWYGSDMGFLQSMEN

WKKVIFLCTLIPIVPFACLLYIISPNSRQLLKQDEYPDLTSCIKLCSLIQLSKMLRCPATKFLTHTLSHA

CFLVLLAVATFRLEERNPYNLSNITDSQCFRHHRATNWNKSKFITQNKCNHSSRYHQTFSKALRNVSFHY

FVDSRHMNQSMKNPVINVDVNGLLWIECKQLHHRGIRVYMLDYYNWIDFFILSLYLGSYVLRFAVDKWMK

EADHFFNGTTKAREALNRHDCKRFHQIKKEIYEGNKTSMSYFMKASRFDWRPDDPEIVSDVLFAAANVLS

IARTTSMMPAFEVLGPLQISLGRMLGDISRFLVLFTLVLFAFMVGLHNLYWYYGFQTYVGKEGDNKIHYS

TVAFQGLPRTLQSLFWSMFGLVPVESVEVKYPGKWESSEYHNNFPGGNHTTSLVEQVGMYLFALYHVTVI

IVLTNMLIAMMSHSFEAIQGDCDVEWKFARTKLWMNYIDHGSTLPVPFNVVPTIKTLKYFIGWSSRIKAY

LLDYRCRKKKNLEVERVNDSCTSDDDSTGIYLNTYSDILQRLVRRYLFKLERMKEENKEVSAVICDPVSQ

FTNEAYTGQLKDPDKDGLLPGNKVMRLSSSTSKSSDRVTNNAPIGRRLSLKPSQMQNKQRQNKTRQNKLG

RWRQLYTNSSFRTNQQMHVHVGFVQLVTLPQLDSVQKILKNLDVRFQRLHERAKNEQRTRQDIKHILTVI

LENQKAVRSYNIVLPSFKREI

>jgi|Helro1|162222|fgenesh4_pg.C_scaffold_4000200(66730)

MEDNPRFNFLNYVNIKNNKTPTSKSLQDNDRSLRRMMSEDGEWNSLGVGVVRKEVDLTPLEKQFLYAVER

GDIPTVQKLLNDNANKLNFNSNCVDALGRTALAISIEYDNIEMLDVLLKGRLDLGGDSLLQAINEDNVEA

TQMILNHEKKLQKQVQPLFNKKNNKNNNNNNDNDDNNNVSNFQMTSSTESQHSIAPVLETVENAIKEKAT

KITARWRKLTVKKWASMDSGGDDNLQDITGNNSCFTPDITPIILAAQRDNYEITKILLDRGDSISKPHDI

RCSCDICVTKTAEDSLNHSMSRINAYRALASPSLIALSSKDPILTAFELSWELRRLSKLENELKIEYEGL

AKQCQNFAVDLLDQVRGSKELEVLLNYDKYAPVRDFGDRMALARLKLAIKYKQKKFVSHANCQQLLASIW

YEGLLGFRRRHIIFKVLLTIIVSLLFPILSLIYIISPKSQAGQFMRRPFIKFICHSASYMTFLSLLILAS

QRIKSIISLAKEADDKSTKMIREVRGAPPTSIEWMILAYVAGLVWAEIKQLWDQGLKSYVHDMWNILDFT

TNSLYIATYTLKLVAYLKVEEEKRNNYPSAYADRKSWDAYDPTLISEGLFASANIFSCLKLIYIFTVNPH

LGPLQISLGRMVIDILKFLFIALLVCFSYSCGVNQLYWYYAEVKSRECSVCEANKVNITSLLNANINKSV

LGSNNSVVDKMATPIPDCHLVCDRSIANLFEIIQTLYFAIYGLVELNKFELKEKHIFTQFVGKLMFGTYL

SIMTVVLINMLIAMLSSSFQKISDDADTEWKFARSRLWMSYFEEGGTVPTPFNIIPTPKTFWYLGKWMFD

RLCRFSTKAKTNKWHSLKRAVKRMQERDVVYQSVIFDLVKRYVMKRQKKTSEGVTEDNVNELKQDLSSFR

YELLDILRSNGMNVPKPSLNSSGNQCPSSRLRRKNSRLSSLNKFSSQESWVTEQTYQVDQSETSQQHLLR

QSASVQQCMHHQLLSRQMSEAKDFNSFPKLSKKCQKQSMMQQLKQNIMFQQQQLALLQQQSEYSDPDSDV

DHENDVVISSFCSDTFTNAYKKPVKKCISSPMSFSSISSTPSITDLNSNNRNNAKIPLTRQKSVETIMMQ

LDTLQECVDEEDCSNKEMTDKLPQPPNIHFKSKIHEITTNNKIEPTNESLTLAITNMPGESTINAPVNCS

NNSTNKKNKNDNIFNENSNNTEKMQVSFQENVTNYKAVEGRTDPYTNCEQFK

>Hirudo_comp26132_c1

ACFKAEIMSSLLNGTDKFGCTPLHYASKQGFIRTIANLLQMGALVVLKNNKQQSALHFAASCGRINTCKELLRGISSSQIVNETDSSGQTPLHLAAAGGHDKVVTLLLKLGAGICKDINGNTPLHLAAVSGYTRTMLAILSVHNHLVDAANKAKNTCLHLAVLESQTGTISLLLNLGAKMQTNIDGLYPMDLAISKELKDSCVAFIAHDRWREAMDLKSNLFGCNMVGMIEHLPEVCQMVLDRCRTPSGADQRDPEFHYIYDFRYLQVDPLYIKESKKRGITILPLISLNLMVKHNRVECLYHPLCLAFLHSKWLSYGVWFYLVTMLLYGISLGCLTTFVVGFDFKVEVLRNSRQALYNASANYSANYSANSSANISTRTYTPYIADDDNDDRFRFDNLTMTQRICVIVVIISSVMCLFKEVVKACQQKLDYCKDANNMREICSCVLCAALTIPLLMDICVDIQWQIGALAVFLAWFNVLVYLQGFDVVGIYVVMFLEIMQTLLQVILVFSVLIIAFGLAFYISLNAGSSPSFETPQVSLVTSTVMMLGAIDYFQTWVEPDSRGELPNQLLNYFLLLLFILLMPILLINLMIGLAVGDIGAVLQNAKLKKIAMQVTLYTGIEMKLPAQLIKMIDKPYLKVYTNSRCSKFKEMAMSMFTVNSDEDDNGRKDALEHQSFLYRELHQQKMRLKQIQKDTSKNYDLLKLIIQKMEIKTEMEDMDEETSDLSLTRKVDGSKDFIGWSSIALKHSMLKQVFTNWSSRHKATAQVDDEETQ

>Helro_191728

MISTTTTKFVAHPNCQQHLTSVWYGKEMAFLQSSSHWKKVTCFLYFCFVSYAYEAFVGLEKLASHLLETL

IQKLLFVLMIIRKLLQQFCCKPVDKMLMWLVTIPAVPLLCIAYLIYPQSKVCLRLFTCLFVCCETGRIRL

KASADVLKLLSTCRFIGYFEGSVGAEVRTGHVTSDGGVVVTDHGSVDDKLNDGLNSGENVMNLSDDEVDG

LLKNTFRPCNVHYSLIIYILGMMWVECKQLYKEGMRSYLADSYNCIDFIVLSLYISSYVLRIVVQFHLSR

ANESFGDLAAKAEKYLRSENFEAFKNLVASTCEEPLSREKYFMRTSRFDWQMNDPEIVSDMLFGLANVWS

IARTTYLMPAFEVLGPLQIALGRMMGDIAKVLVLFTLVLMAFMVGLHNLYCYYGSQTYRLFNNETFRTIH

ASETFQNLWRTLLNLFWSMFGVVPLDSIAIKHPSQIYGSLPQHSFSSTSLSAPSSSLSAPQTFITVASAF

ATLTQTTLTPHPTLRSLSSSSVDILSSSPSLSSFSPSPFSSSPLYLPFKPPSPPSLFTSAHANATPPAPS

IPPLPLSLPLLSPSFQSSSFSKSSKSFSKSSSSSSSSSSSWSSSSSLSWSSPSDASLASHPGSTIIVEGV

GLFLFALYHVTVIIVLVNMLIAMMSHSFEAIQGDCDVEWKFARTKLWMSYIIVEDTLPIPFNMLPTLDAL

KSCCGGFLSHIRGFNGNIVKMLQVICQKKLILRTKSELIQTLNCHVYKLVVGSALSHAVIRAWLYSNAAC

LKLENYHAPWRHGKLHHTTNADILQRLVRRYLFKLERMKDEINNHHHVHLGDHVHHYDHNHHHDHHHHDH

HHDHHDHHNHYGQADRDPTRSTSEMTSSITYVPDYAKDRKESDAKRKTTQSSFGSNQSLVFNFSPSSLVF

TQQHGGGSSKPQRTRPPSIRRRRQQHVSCQQLYQLNLQEYQESSEQEQPQQQQHQPQHHLSQQLQQQLHQ

QKHYSQEQHREQNDLPKQPQQQYTHSTGNNISSPDNNIISRYGNKCSLNGNHNTTITDNNNISTNHNNST

TNSNNSANNNGDSITKIRSSGTSLSSQPPTEKTKIQIEAIIKNINLFQVRLSHVRAITTRRDVLRDDIKH

VKRLLLENCKALLSVLSVLESIRVEVKCLETIARLKRENMMKK

>jgi|Helro1|171704|fgenesh4_pg.C_scaffold_21000221_Helro_77785

MFKSSNYFDVEASWRLQNREQLDNPIYRYINLQKEGRLADAFNRCGVEGLEQLVRDELPNFMYNAGAGAV

ITKNEYLDWVNVIESKMQNKPSKKEKTGQSFRSLKSGLSISRKFKRNHLDRVGPLNVEHLTNNDGSLWID

SNLSNNYYFFESTGNVEQNNSCNAVTELYKFTQSEETCNVVNKLRSKHKHDELSIDNFISNGIRNHVKLN

NNIGILNNSDDKNDCDTVPVKPFVKMFKEHEGCWQLRKRGSVGESIIHLAVLYSKSSHIYKEITKVLLNIFPKLALDLYEGEEYYGMSGLHIAIVNGDLELVKLFVEKGSDISQRATGKFFLPDDQKNGPSTESTNYYGRAYYGEYPLAFAACFGYESIYDYLMEQGADPDQADSFGNTVLHMMVICNQTAMLSAAMRHQFKRPNLCLANQQNLTPLTLASKLGRMEIFSEIIELQSYEIWRYSNITCSVHPLGPLDTIGQNGAFNPNAALVYITNGVSENHLEMLDDGIIKQLLLDKWKNFAKRRFLERVLLAVFHLLVLSVAIYLRDDVVFVDYIFPPNVTDIIRYFAEVVVLAYCLVTVVIQVFEMKTQGLSAYVQRETPAKCVFNVSCVFIIICLPVRCLHYQTAEKILLALAAPCAWANLLFYARLDIFFCFFIGVFVVVFYQMLRGDLARFGIIYIIFFIGFGQSFSFLFKTTKPATPDCKASQLPFETIGGTIMKLFEMTLGDYQYESFSRTANPILTKLIFILFAIMVPILLLSMLIAMFVHTYEQIIKRSEKEWKRQWAKIILVLERTFSPKQLVHFQENYSVCIGGNTNPIRGLMVIKNTFKTKARQRKDALANWKVRFLRKLFFKFLKYFINVSKETVQNGKKK

VSFHSTLTCWARKFK

>Helro_188397

MVGQIKNEIAANRSDGSTNGRVNKPCWIEETFQKRECVKFQRDRVDKTRCMCGRSEAFHLDKRDKFSRGNPQYSDQWWDVTTHTESSPTDAYGVVEFQGGSYTTKAQYIRLSSDSRPEQILQLLSTQWGLHLPRVLISIH

GNSANSSFCLQPQFQKFDNEEHFWTGNGRSSGAWIISGLAKVDGDTGDDSPIEHRPNVVSICIAPWGLVH

KREDLIGRNINVPYFAQPGSDNAALTKHHSYFLLVDDGTEGKFGCEMILRKKFENYISQLRTNNHKSTKD

NCIPVVCLLMEGNICFVRKVLECVTDKPPIPAVLAVDGRGGAADLLSFACEQVNFDGTMPEISKRKLLSL

IAHNFNYEQSETIFSELLLCAMRKNFITFFKLDEEKQDIGRAILYALIKSRKQNPFYHLNLIMTWDRIDV

ARNYLFNGGVNWPDGSLDNVLMDALIDEKVEFVQLFIEKGINMQTWLTVQKLEDLYNQVLNAPRPVLAKY

VHKSSNNEKLDASSTLKHLLAELRGNTPGNRTTLYEIGQVVNKLMGGCFQSTYSRRKFLVLHSAAKSKSR

KVSTASRISTISGTSASDDEYKQLLSDTFRYPYSELIVWAVLLRRNDVAMFMWQRGEEPLAKCLVAAKLY

KSMAREAEKDEYEVDTALEIRKYHNKFSELATKLLDQCFRTNWKLSQLLLTYELLNWNNQTCLSLAVSGR

NRFFLAHTCCQLLLTEMWKGGLRIRKYSSLKVLFGILCFPSILFFKYRSQEELQLMPQQQDVHADDSDGE

TSSSDSESIENENFMILQTLRQQQQQLHQFSNVVIDSSSILEQQRPTISTVAIAALATAGATNSLTNHAA

TNEASQIISPFQRQIYRKRRFSKPFRHPAKNSNLRRGSKHSLGKTASPEEIIGRLNEHHKQSQLHAKSKI

LKFYTAPITKFWMHLIAHLIFLLLYTYMILQTTPADPSTIEWFVVAYMCGLGVEKIRELMTCEVARIGHK

ILSFLSDVWSIIDILSVGLFFIGFAIRFTPALVSSARAFYCLTVMLSIIKLIEFFVISPRLGPFGPMISQ

MLKESISIVAFVFVLIWSFGTFRESLWFPDHDFEWTSVREVFLKPYIMLFGEVYKEFIWPDCVANVTSPD

DFKCVPGRWLVPAIFSMFMAIGYVVLFNVLIALFNRIYMQCQEHFDIVWKFQRYELVIKHVSKPILPPPF

IIISHFISLLLFIRQRCSKKWNRCDRGLKIFLHENDLEKLHCFEKECLEDYQEAKMTSPFRTVVCLSDV

>Helro_159882

MAYKNTALHYACSNGHVEAVQLLLEKKADVSLTNTYNNSALDLAIDNLHQDVVMIFDYQYIDPGPDDASS

QRNRYLALNTMVHHGRDVLLSHPLCQNLLFQKWVRFGRSMFIINLLTYIGYLTCLTALVVIKYQDCPPSH

TNISTTNCKLEETEMGESLPVYRSGSDDNSSKCLQSPLTCLSRPHPQYATQRILTVFLYVFVAVFFVRDF

IKILSQRWRYILNIYNTIVWLLMLATVSFLFDSQFFDRWRAAWVAMFLAWINFIMYLRRIDFLGIYVIMF

VSVLTSLVKGSFEDIFTSIISVLIMTLGEVNYLELKQALTIFRVDGDVLIIIFVFLMPIALMNLMIGVAV

GDIEKIQRNAYLKRIGLQVDLMYNIENNMPRLLQKKFHIRRTAIKPNEEPEHTNLTCFGQRRKVQFIEAE

KKDKTTSRLESLIEKTQAQQQSIKVLTMTLQQQNILLHEIARKFEVSYPHTGITSHLGFGGGGGPHGGAG

GFVYNRPTSYLWDSQSLYPSYKLGNNASNPVTPEPSVAGQPSFATTIQPPHQPKMLIPESVSVYQNVNLD

GVDRWRKRSIRPTKNVVIVGDFVCPTFDDDKPKTYI

>jgi|Helro1|116296|estExt_Genewise1Plus.C_790001

MRQKHCAAMPANPNCYGSFGQPSVNQNFVYVGNVEKDEDGSVELSTIYGPWTLEEDIFRRRLKYFFMNPY

EKFQARSRIPWKMFIQILKIVIFTYQLIDFGSLRSLHIEFMETTNLALKHLYLKDWTTSYETMPYPPSMG

KMACYTDQEFFQSVDFILNRYNETESIAIGNFSPAQENGTTPPVEMCVHKYKEGVIWAFNQTFIFDSRVI

KDCFKVMPVFDPPSNLSVYNIEKFLESVNRTINFDSLIRITLHFNLNTVHLKSLHRSSDPDCFLLNVRIL

FDNSEHSGQMLLLLETYVNDVTCKGDVLFDQGVDISEVFSWIFNGIVLIICFVSSILCLRSFHRAIVLKK

DTVKFFETNYPSVIEVTWSDKMDFVDMWYILIIINDLLTGAGCICKIFIQMKMNTNYEICGILLGTGSLF

AWFGVLRYLAMFKSFNVLIVTLKRAALSVFQFFICAFFIFIAFSICGWIVLGPYHIKFRELTTSLECLYS

LINGDDIYTTFATMNQVSTGVWLFSRLYLYMFISLFIYVVLSMFITIIADTYDSIKHPDSHEYLKSRLQR

FADECCTRASQLYSRPEFQHLHSRRNFIFAKLLKLFGHKGRSARSSLVVNESRDEEQSLLTSS

>Helro_72856

MGNKLSSNVSDAVKQQATGGSGQVVIYQLADVKGGGQLIEIMKEAKKTKNYEKADSLIREKLKAFVYKNG

EGARVSSSTLNKIRIGLLSHVLEFDQYISYRENIQKEIGTNNNICSEGNSYKKVCWRLDERGSLGETPLH

VCFLNATPTHAELAKRLLAIYPNLINDVYVNDEFYGESVLHMAIVNEDPAMVKYLLDRGSDMHQRCSGTF

FLPDDVKGSCVASETIIQLSKNNHIRNNRHTYWGEYPLCFAVCLGQEECVRLLVAKGADVNKQDSFGNTA

LHIVVIHYYKDMYDLLVSMHANINLKNKQGLTPLTLAAKLGHIEMYQHIMQTDREVYWTYGNVTCAGYPL

DHVDSISETGHINSKSVLYLVVFGKKPEHLDMINGALEQLLNEKWKTFAKARFFRRLIFFLLYFTVATVA

FSLRPGTVIPSSPIKSNSSQISKYHSDYENEVTSRRVDVSFCHLNKLDSYLAMEYEGGVDEKAMRMVTIH

YVDPCYQIQIQSSVDIARLVFEVLTVIGSLIYIIFALRELHFQGCKNFFTSQKTSPTKTLFLLSNMIIIA

MVPFTGACSYRATDCMAVLALLSTGPYFLFFCRGSKIVGQFVVMIYWMIRKDLLRFFSIYLILLVGFSQS

MFIMFRGYDNPAFSAPLESIVGMFRMSIGAFAEMYSKFETNHFLNEWNQVIFIVFMILVALLLINMLVAM

MGHTYDCVNQSQREWMRQWAKIILVIEETITPESRRKQQIKYSQEMRDGRRALAIRWNDKVGGSNTN

>Helro_85683

LTDEERVYLNAAALGDAPIVQKSSKKRPKTPRLNVNCLDYMGRSALHLAVDSDSMETVELLLDRIDFDCI

EEALLHAISKGATKTVRVIIEHPNYIAGSIIIVLGAEQAKRSRKDPFFRTDEKSQFSPDITPLILAAHYN

NHEIIQMFLSRNHSLDRPHPISCQCGDCVTKQNYDSLKRSRSRLNAYRALASPAYMALSSPDPIMSTFEL

RQEMKKLAQVEKEFKIGRALRCPASKFITHTFSHICFLVLLAAATFRLDNQTYPMTFNLTNDLFAPANVL

MTNVQICLMFWIFGLLWMECKAMYNSGLRVYLLDSYNMIDFVVLSVYLSSYVLRFLVDHRIKAADRFQNT

HTNTHAHTHTHKHTQTHTHARTHTHTARFEWKPNDPEIVSDVLFAVANVLSIARTTYLMPAFEVLGPLQI

SLGRMIGDITRFLVLFTLVLFAFMVGLHNLYWYYGSQTYKKEGTEKLVHTSEAFQGLFWSMFGLVNIESV

EIKYPNKEKGSDFAYSFPGGNHATSVVEGVGMFLFALYHVAIIIVLINMLIAMMSHSFEDIQTDCDVEWK

FARTGLWMNYIDDGGTLPVPFNMFPTPKSFIYLYKLAFVHSFVRSFVHSFVHSFIHSFVRSFVRSFIYSF

IHSFIHSFIHSFIHSFIHSLVASIDHTYDNNNNNNNDDNINF

>Helro_167034

MKRFFLGITRQTDNSKVAHEKPLSVARSYSDDDELDGRFKVKLISSPNESASNNSSTLTVNDCHTNSMSE

RTNSVMRRRIRRQRPVSNLVAKARQELSTAIDFDDVKQFEAVLLRYESSGLNLESRELVDYYSGKSFFHE

ALEKDKRDICDYIAANCSISYLLGDYPDIVRRESKRRMKSSLVFRRKTCLHLMVEKGNKKTLDTVMNRLK

KACVNVDDNTNASWPKLPSFGFCKTQKDLDTFLNATVTFETKDRRRRTGTAIHLAVVNGRWQMLELLVSY

GLSIDERDSEQETALMLAVQQSQIESLVELVRLGADINAQNKQDCAALHMSVGKDNQMVKVLIDEESTDL

NIERIKNPRWAIEMAAKTNNAQVVGWLIDRKKGKGAVFRANVHQALLIAASNGFADVLSTILERKPKLLN

KPDDFGNTPVMLAADSGKLEAVQLLVEMPDIDLDRRNNLGQSVFDLAMKLEDESVLSFLLGRVRFNEALD

CFYVHTAVSNGKIERLKMLLLDDDHHERLHLLEESTENSVYHTAAAFNSSEVIDLFLDENDDIKDLQNKN

YETALHVAVRRNHVESVRSLLNKRVRVDILDSEGQNALHAAVTTRATGIEIVEMLIEAFEKVDPMLVNEQ

ESNGNTALHLAAMFNKPEVIRILAMCDPEIENKDGETALHVAARAGSAESVRTLLELFYGDRKANIDRRD

ELGFSALYRSAENGDREKYELMLLYGADMAITCESGPSILHRLVECCKADPNKAEKYVSIYKSTVEWAVV

WYCRKKGLRCPLANTNQFYLVQLDAVRLLTSDTQYDGCNVLQYAARKGESKMLKVILETANVYKFTKCSP

LKIERTQESMSHNVKSSILSIMSNSNSKNDNPHMGQNNTNNGELAKFLEDKNALPTNAVLYDVTFLIPGH

IPDSTIRREHRDSTNSEAEQRRRRSSAPPKSNVSCLEYLVESGQESDINEILSTEPFHSLVYNYWTLCRR

GYELLMWTHVTYMVLLTYFVIPDSSWIQSRFHNLSENIYRNGTLISNGEVIVGSLFSVRIYSFFLIWPAL

IFIYELITALHFIVQLRYNKNNMSLRYELLGRLKIGEITSGLMRIPVGLMLFIFDNLSHISSLAFALSCV

AWYVTCVYASSVQPYLECLALHLVFGWLHTINYVKGFKDWNALASILKTILIKDITRFIYIYIFVLISFG

YAIHVLIQANIHSRPELASSTSTIYLTFYQMLSPGNVLDVTKSPNGNGTDNAPGASVLRGVYATYSILSS

VVLMNMLIAMMNSTYSDVKSVRSTAWRFESLRMALWIERNFKLVRKLRLIRFKARFDKWQNRWFLKRTPA

GELKINSDILLGKIESDTPDKDRDVQTRRKSEDFTRKLEDMRSEIDRLNEALMMYEDKTARVFNGLNMEL

KHIHELCLESYQLARVSNRK

>Helro_169550_169548

MRAVMALTLIIYNTRLSSHDDAPGHVSTSTTSSLFFFLFLLILCPSSLFIFLRILHTKSCKEEDLLEAAESGNLTKMRWLLDSCKPGELNIEYTDQLGRTPLHLAVVNEHKEVVRFLLDLVSHHCLYKVLLAAINIGNEEIAEIIIEHPKYANISSDLKSQNISSDVRGSSTNITDDNQFFEEETSRDELKFARSRLNAYRGMSSEAYISLSSDDPILTSFKLRKEIQQAEYSALANQLSEYAVKLLDKVHGNEELNAILNARDGQQEDGDETSGGELARLQLAIKYKEKKVPLCVNMATSLQATTSAARPIIKNIVDQFVAHPSCQKKLTSLWFHDLNNNNLMSRSSFLHALIIFVIILFFPILSLVYILFPNLKVSQLMKTPCMKFVVNAASYIFFLFLIVIQGHVEQGKLCQERLGQHDQLANHIKTFSSSFII

RNSTVFENLKSATNHLNAYVDGSINRPANENECMVLKRSFHCRNVSEICIRNYRPAAMEQVYRTGCRDHF

INVYNAMDFFMLGFYMASFTLRRITEQLIWKSLTFYKNAASWMKLKDDDPAAVYWFYWLDADRYYWDSWD

PVHVCEALFAVGNIINFARLFHLLSINEHLGPMLISLERMIKDVMKFMVTFVPIFLAFMVGMHNLYWYYD

PRVRSVVEMKDHKIETNAQEGFGNLELTFRTTFWALFGLKDARTVELGRGFESRFTESVGYIVFGVYNWG

AVIVLLNMLIAMMTRSFDKIATDQDIEWKYVRSKLYLDYINHGSSLPVPFNILLAPKFIFKHLKCAVTWC

FEGSFCDEKKEKKKNFGPISRSLRKKMQTEVGRNDWTLEKALNAVGKIYENISIGGTNNTITANNSNCTA

NNNNSTVNQHYHNNDDDNDFICVDDNNERNNDNNNEYCINCNFYAKNEMSGNKKFLEKKANTNKFNYNKY

PLITADRTSSVANNNINKNDQIKIKNINNRNILRYNNDTDNNNFQQRLAKHNNNNNGSHKIYGSSKKSNN

SVNSDNNKTNNIYNYCNTNNNNNISNNNDVNDDNDNVRGNNFTRGMRRNDSVTTTVNKDVSDNVIKNIVR

RYIFDRLLASVGNSEGSNTTSLLTQQLDSMQENFQLATNRLSDQLAKLSRDLTLQHSSTFHNNNSNNNNN

NNNNINVSHVSNVINNPNIQKSSKFRSRIQNILHKHHSFNIPSMVSDSLDNNISEENSSSVNQNATCTFN

CAASNHLYTNKSCTDHNVTSNGFDSAATGMSRRNVSHITDDNSPKRFGGENIKLEHVNQYVLKKKSFSNW

TKNHGQTNSAHLHCGDIYKDSYNSNSNVISSSTCESNNSDNNNPNSNYINNNNPNSNYINNNNNPNSNYI

NNNPNGDDNTCSTNNNHSTNIRSLADGTPLANIKNTPNLIGTTRQQHINSSNVDFKTADDNYLTKSNFLC

KFITTRSADNNTFY

>Helro_163554

MSAKLLHHKLSFSGFGQEASKNVPYFIVPVNKNEKKPANHNTNIVHNNSNSSNNKNNTSDVSEAIKLAAR

SVWKHMYDARNGKLPKLIISVTGGAKRFEMSKKHLESFKTGLMKAATTSEAWIITGGTKNGVMEFVGDAV

REYMLENGSYEQNLVVIGVATLGAVVHCEELIKECKPPFNTDNHLPYKYTYPEQALVKQALLDPHHTHFI

LVDDGSRGSFGREIEFRAAFEQHDFSYVEEMTLLQMTLPTAITSTTTEAATVTDSTDTEMESISMLPVVL

MVLEGGINTLETAVEAIKKNTPIIVLQGSGRASDFLVKCMDYIALKDMKKMTDEMIKEAVIYFPDQPNVP

EKAANYAKILLDPSKKHLFTIFYQKDDTSGDDLAEVILYALLRTSISTSENANLTPESTNRFLDLALSFN

RCDIARKEILKYEKKTGLLQPSQLNGNFMSALKQNKPEFIQMYLELGVEVGSFFTYRMLLDLYKYVFEIS

KDSTPTAGVFKSLVQDLDVRFRISLFVFEFIAHPDSLLEKPYREIFFWCTLFGHHDMARIFWRSGLNYIG

QWRLDGQSDHLYFINLSKYEEDAKGILDECYTFDCEISKNLLVRSLGTWGDKTLFDLANDGTMMNFAEHD

CCQAKLNSIWCGKLRSYTSSWTIFLGLFFPFIMVNFIQFRGSDANNQIAYLIFLAIFSYFVLLELHGGSI

TVIEVLTWIWMFAMAADEIRQMFTQDAPTILLKFYKYYYYDRWNLYEQLIIVMMIVAVILRFTLYSSDTY

SARVMYALTLLLSILKLLKQFYVHQYIGPKVIMIQKVIFDLMFFIFIFGVFLMGYGITVQALLYPDPPHS

WNVLSNIFYQPYFTLFGQFNLMELMGQVQSCTTNHTLYLSDPLSNPLCSQKSWVAITLFAMYAITVCIML

VNLLVAIFSFTFNSIQESAVKTWQFYRIGTISEFFKKPALFPPLILIGHVILLVDVIINYCCSRKDEYGR

RIKSPVRNDFRMELDEKESKRLGAFEKKGMLSYFIKQKDAMRQDTNHKIDVINSRQEMSIDLESIRGAIN

RAKKSKVFDVGFDDAGDVTSRRMHNNVNVASNNASSRQVDEQMKELEERMKGQFKNFENSINRIEQYLKN

INNNYSNNNNNNNNNNNNNNNNNNNNNNNNNNNNNNNSSSNSRISNSTSNSRANNNSGKFSDTSSNASSR

KGRICIQIANTFDPWEFKICGLRAVRSLPTPGLCRR

>Helro_63393

MDSSNDKHLNHLEKVLLCAVEKGDKHLVVRCLHAEKPVDINCSDFLGRSPIQIAVEKRNISLIKILLEQD

GIQIGNTLLYAICEGTYEIVEMLIDHHSVTADMIGSDWGFNMKNDFNISDEFFPEISPIVLAAHCNHFEI

IQLLISRGATINKPHPLSCSCSKCRKETFEDSLRYSLQRIQIFRALSSPAWISLTSFDPVLTAFRLSWEL

EHLAMKENEFRDLYANLSEQCKQYACNLLDQCRSSHEVIAVLNKSVDSAYDKWTTEWSDINKLTLDRLKL

ALKYRQKQFVAHPHCQQLLASVWFEGIPGWRRRNFITKVVIIITLICFLPIMALIYLVFPRSRIGRLVRT

PFMKFMYHSVSFGVFLFLLILVSTNFGAADGEYTQRQHQRGPPPTNLECLIVAYVMGFVWGECKQLWDEG

LKAYLRQWWNWLDFIMLSLYLCTLSLRLIAYFHSQHPDIYGLSLAPRNQWPETDPTFVSEGVFAIANVFS

FARIIYLFQANPLLGPLQISLGCMLIDIGKFFFIFFLVITSFACGLNQLYYFYSSSNSSHEIETNHQMTS

QEMQNTNYENAFNPSYVTLFWSLFGLTQLQTVRDGQLKAITKAVGECLLMFYHTMAVIVLINMLIAMMSN

SYREIEDHADREWKFARTKLWMGYFDDGSTLPPPFNILISPKSIYYFLRSIKLLVYKILYCCHIKPSTKK

MMRKSMKSKEKLLEDNRRQYTVRSIYLKNGLTTMLTLMCMYLFKILFVIYQQDVMRKLVNRFIHQWKIQL

RQESINEDHISEIKQDISSLRRELQNDFKKDEFQSSFMFEKIKKDIARLARRNSNPPFKSEAAFTSSTKK

KSKSMERRFDSTTDNDILPRKFSGTYNNFNSYPLADDEMKNKNAIISYELDYLQQIQKQHGNSSPGYKQQ

NKTECDVNLLKNQIIGSLEAEVGKIITNALSPVIDYLKRHTSEASSLHLQFLKLVRK
